# Assessing the impact of long‐term storage on the quality and integrity of biological specimens in a reproductive biobank

**DOI:** 10.1002/btm2.10692

**Published:** 2024-06-26

**Authors:** Zhao Wang, Changming Zhang, Xin Zhang, Yuehong Bian, Yongzhi Cao

**Affiliations:** ^1^ State Key Laboratory of Reproductive Medicine and Offspring Health, Center for Reproductive Medicine Institute of Women, Children and Reproductive Health, Shandong University Jinan Shandong China; ^2^ National Research Center for Assisted Reproductive Technology and Reproductive Genetics Shandong University Jinan Shandong China; ^3^ Key Laboratory of Reproductive Endocrinology (Shandong University), Ministry of Education Jinan Shandong China; ^4^ Shandong Technology Innovation Center for Reproductive Health Jinan Shandong China; ^5^ Shandong Provincial Clinical Research Center for Reproductive Health Jinan Shandong China; ^6^ Model Animal Research Center Shandong University Jinan Shandong China

**Keywords:** biobank, DNA integrity, long‐term storage, quality control, RNA stabilization, serum marker

## Abstract

Biobanks hold a pivotal role in facilitating translational and clinical research endeavors. However, the effects of prolonged storage on frozen blood samples analytes are not well defined yet. The aim of this study was to investigate the long‐term stability of the quality of DNA, RNA, and endocrine markers within blood samples amassed from the biobank over the past 11 years. The results show that the overall quality and integrity of DNA remained not significantly influenced. However, RNA integrity and purity displayed substantial deterioration as storage duration increased, to ensure high‐quality RNA for downstream analyses, advised to prioritize using blood samples stored within 3 years. Furthermore, the study examined the influence of storage time on endocrine markers. Through repeated measures ANOVA and linear regression analyses, it was evident that storage duration significantly influenced the levels of endocrine markers. This insight aids researchers in selecting appropriate markers for their investigations and augments the precision and dependability of results when dealing with long‐term stored samples.

**Translational Impact Statement**

Ensuring proper quality control of these biosamples is crucial, as it helps maintain the stability and reliability of biological markers and ensures the validity and accuracy of research findings. This was done by comparing these findings with the initial data collected before storage, thereby establishing a foundation for quality control of the stored samples, with implications for the broader field of biobanking and translational research.

## 1 INTRODUCTION

Biobanks stand as repositories that amass, preserve, handle, and distribute human biological samples alongside correlated data, dedicated to the domains of research and diagnostics.^1^ Beyond their conventional function of uncovering and substantiating biomarkers, they play a pivotal role in propelling the genesis of novel pharmaceuticals.^2^ In this capacity, biobanks serve as a conduit bridging the realms of academic research and the pharmaceutical/biotechnology sector. With the aim of delving into the origins of infertility, the inception of the Reproductive Biobank back to 2006 at Shandong University. This initiative was driven by the purpose of amassing reproductive ailment specimens augmented by comprehensive clinical and lifestyle data.

The success of translational and clinical research heavily depends on access to high‐quality biosamples.^3^ Ensuring proper quality control of these samples is crucial, as it helps maintain the stability and reliability of biological markers and ensures the validity and accuracy of research findings. Prolonged storage of blood samples, along with repeated freezing and thawing cycles, can cause cell damage, reduce nucleic acid yield, and compromise the integrity of these molecules, thereby affecting downstream molecular applications.^4^, ^5^ Despite these challenges, there is still a lack of standardized quality management protocols for the long‐term storage of blood samples. To address this issue, we have selected specific biomarkers, including reproductive endocrine and metabolic indicators, to assess serum preservation quality. Additionally, the purity and integrity of nucleic acids were used to evaluate the preservation quality of blood samples. This approach aims to enhance the reliability of long‐term stored biological samples.

The prolonged preservation of samples inevitably influences their quality. Samples stored at −80°C tend to uphold their quality, with DNA maintaining its integrity over extended periods.^4^, ^6^, ^7^ However, it is essential to note that RNA samples tend to undergo degradation even at low‐temperature storage, a degradation level that varies due to distinct handling approaches and storage conditions.^8^, ^9^, ^10^ Furthermore, the stability of specific analytes within serum or plasma samples can also be compromised by prolonged storage. The extent of this impact hinges on variables such as the analyte method (e.g., hormones^11^, ^12^, ^13^, ^14^, ^15^, ^16^ and proteins^17^, ^18^, ^19^), storage conditions and duration.

In this study, we assessed the variation in quality of DNA, RNA, and endocrine marker within samples retrieved from the biobank over the past decade. This was done by comparing these findings with the initial data collected before storage, thereby establishing a foundation for quality control of the stored samples.

## 2 MATERIALS AND METHODS

### 2.1 Collection and handling of blood samples

**FIGURE 1:**
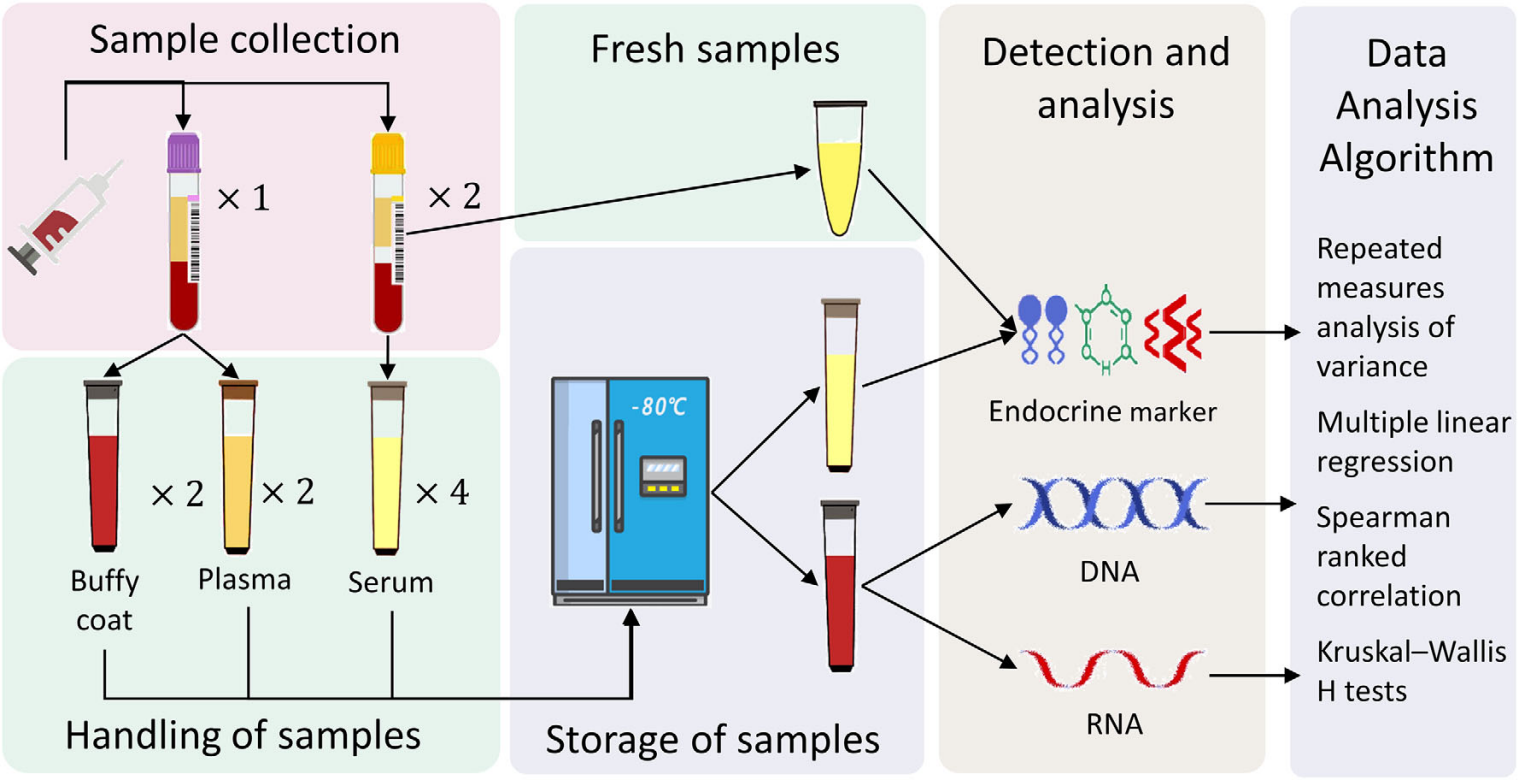
Experimental workflow of sample collection, sample processing, and data analysis.

For context, since 2010, the Shandong University Reproductive Biobank has collected biological samples from women of reproductive age who provided informed consent,^20^, ^21^, ^22^, ^23^, ^24^ with samples including serum, plasma, buffy coat, and lymphocytes. The workflow of sample collection, sample processing, and data analysis are shown in Figure 1. We examined 50 cases per year from the compiled samples from women ranging in age from 22 to 40 years, taken between 2012 and 2022 (a total of 550 cases). Blood was drawn by venipuncture into one 5 mL standard K2‐EDTA tube (GD050EK2; GONGDONG), and two serum tubes with a gel separation plug (GD050SG; GONGDONG). Blood collection tubes were centrifuged at 2000*g* for 10 min at room temperature so that the blood separated into serum, plasma, buffy coat, and red blood cells. One of the serum tubes was analyzed immediately after collection, and the remaining plasma, serum, and buffy coat materials were aliquoted into 400 μL fractions into two‐dimensional barcoded microtubes (MP52519L; Mironic) and placed at −80°C until analysis.

DNA was extracted from a 200 μL buffy coat of the microtubes using a DNA Extraction Kit (Whole Blood Genomic DNA Extraction Kit for Pre‐NAT II, EUROIMMUN, Germany) on an automated workstation (Perkin Elmer Pre‐NAT II Automated Workstation, EUROIMMUN, Germany). RNA was extracted from the remaining 200 μL of the microtubes using a Total RNA Extraction Kit (Tianmo #TR205‐200, Tianmo biotech, China).

The DNA and RNA quality and purity were initially quantified using a NanoDrop One instrument. Gel electrophoresis was utilized to resolve DNA for 550 samples on a 0.8% agarose gel (Biowest), stained with GelRedTM, at 8 V/cm for 45 min against the 1 kb Plus DNA Ladder (Tiangen). Subsequently, an Agilent 2100 Bioanalyzer was employed to measure RNA Integrity Number (RIN) values and to conduct electrophoretic analysis.

We obtained 550 serum samples (50 per year from 2012 to 2022), 500 μL of which had been tested for relevant endocrine marker (FSH, E_2_, LH, GLU, PRL, T, TG, TC, HDL, and LDL) at the collection time (i.e., fresh samples) Following storage, serum assays were conducted to compare the levels of FSH, E_2_, LH, GLU, PRL, T, TG, TC, HDL, and LDL to those from fresh samples to assess the quality of serum preservation. PRL, FSH, LH, T, E_2_, and TSH levels were assessed/measured in blood samples using a chemiluminescence assay with a Cobas E 601 Immunoassay System (Roche, Switzerland). This was carried out employing commercially available kits (Access Prolactin, hFSH, hLH, Testosterone, Estradiol, and Access TSH, Roche, Switzerland) in strict adherence to both the manufacturer's and supplier's guidelines. GLU, TC, TG, LDL‐C, and HDL‐C levels were assessed using a Cobas C311 modular analyzer and appropriate kits (Glucose HK, Gen.3 [GLUC3], Triglycerides [TRIGL], Cholesterol Gen.2 [CHOL2], Diagnostics Gmb, Germany; Cholestest LDL, Cholestest HDL, Sekisui Medical Co., Ltd. Japan). These measurements were conducted meticulously, following the explicit instructions provided by the manufacturer.

### 2.2 Statistical analysi**s**

The serum samples obtained from the biobank underwent at least one thawing cycle before being used in research initiatives. To mitigate the potential impact of sample thawing, the samples cryopreserved in 2022 were specifically designated as the baseline measurements for the years 2012–2021. Calculation of the Correction Factor: A correction factor was calculated by manipulating data proportions from baseline period, predicated on the discrepancy ratio observed between the fresh samples from the baseline period and samples that underwent storage. This corrected data guaranteed that the median of fresh samples and stored samples from the baseline period stood at 1. This computation was executed with the following formula: corrected data = CX/C0, where C0 is the median of the 2022 sample, and CX is the experimental sample. Statistical analyses were conducted using SPSS Statistics for Windows version 27.0. (IBM Corp.).

Spearman ranked correlation analysis was performed to assess impacts of storage duration (years) on the total DNA and RNA yields and on the RIN value (for RNA) for each sample. Kruskal–Wallis *H* tests, using multiple group comparisons, were conducted to assess differences over storage for DNA and RNA yields, *A*_260/280_ ratios, and RIN values (for RNA). Effect quantification analysis was conducted to determine the magnitude of differences. We utilized repeated measures analysis of variance (ANOVA) to assess the effects of the sample storage on the outcome of serum endocrine hormones and related biochemical indices.

The sample was comprised of 11 groups, classified according to the number of storage years of each of the samples. Multiple linear regression models predicting the concentrations of endocrine markers were developed for the stored serum samples. Relationships between the dependent variable and independent variables were assessed using linear regression with the ordinary least squares method. The model's goodness of fit was evaluated using statistical measures, including the coefficient of determination (*R* ^2^) and adjusted *R* ^2^. Hypothesis examination was conducted to determine the statistical significance of the given regression coefficients.

## 3 RESULTS

### 3.1 Influence of storage duration on DNA yield and quality

**FIGURE 2:**
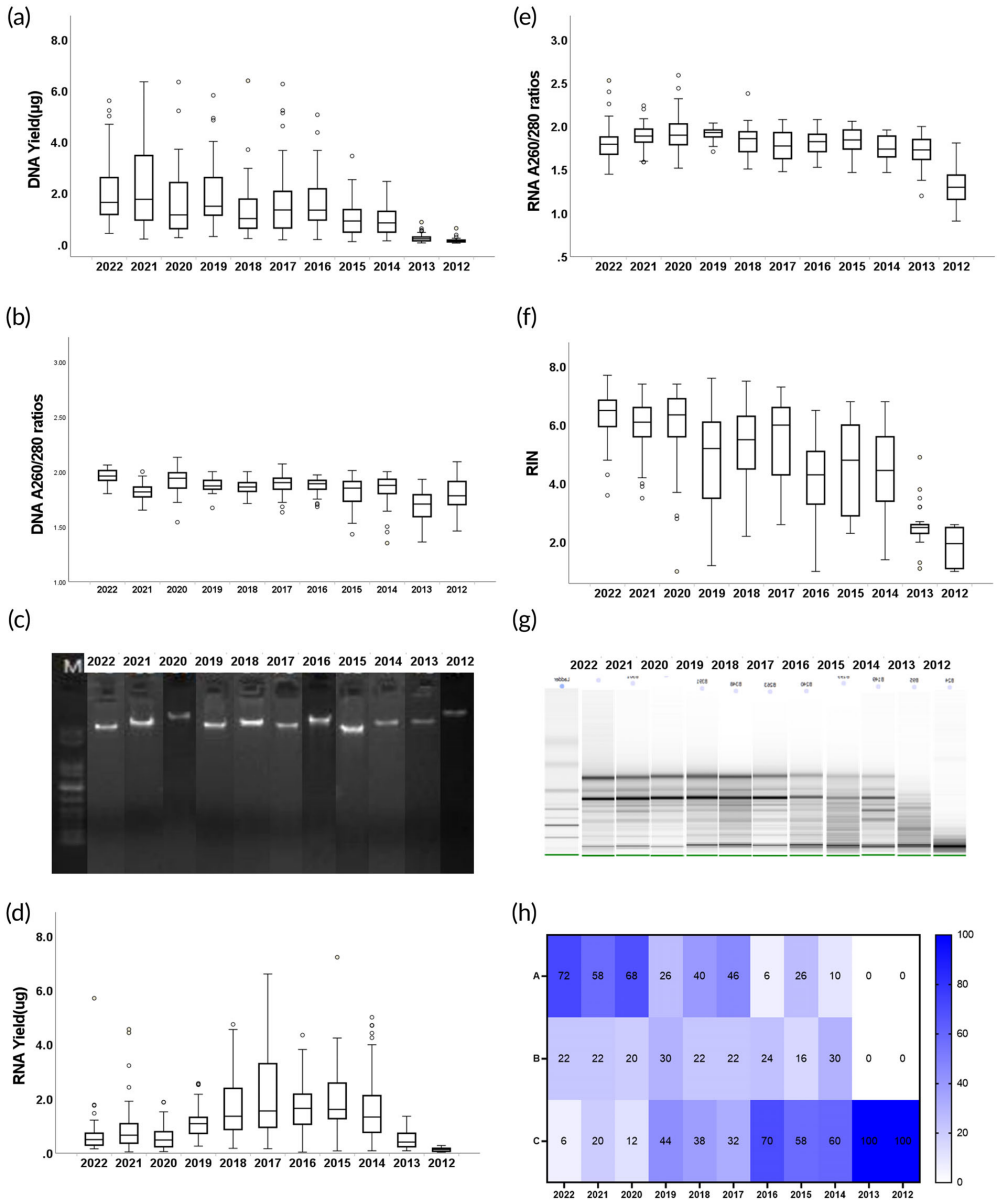
The quality control results and analysis of DNA and RNA. (a) Yield of DNA extraction from preserved samples across 11 groups (50 cases per year). (b) *A*_260_/*A*_280_ ratio of DNA extracted from 11 groups of preserved samples (50 cases per year). (c) DNA electrophoresis outcomes for 11 groups (50 cases per year) of preserved samples; additional electrophoresis results are detailed in the accompanying table. (d) Yield of RNA extraction from preserved samples across 11 groups (50 cases per year). (e) *A*_260_/*A*_280_ ratio of RNA extracted from 11 groups of preserved samples (50 cases per year). (f) The RIN (RNA integrity number) values for 11 groups (50 cases per year) of RNA extracted from stored samples using an Agilent 2100. (g) RNA electrophoresis findings for 11 groups (50 cases per year) of preserved samples; additional electrophoresis results are outlined in the attached table. (h) Based on RNA yield, RIN value, and electrophoresis results, a heatmap was generated to categorize RNA into three levels (A, B, and C). Grade A: RIN ≥ 6 and 28S/18S ratio ≥ 0.7, total yield ≥0.2 μg; Grade B: RIN = 5.0–6.0, or RIN ≥ 6 but 28S/18S ratio < 0.7, total yield ≥0.1 μg; Grade C: RIN < 5.0, total yield <0.1 μg.

DNA yield decreased as storage duration increased. The median yield of DNA from the 550 samples was 1.04 μg (interquartile ranges [IQR] 0.41–1.8), while the median yield of DNA from the samples stored for more than 9 years (*n* = 100) was 0.23 μg (IQR 0.15–0.305) (Figure 2a). The *A*_260/280_ and *A*_260/230_ ratios exhibited median values of 1.84 (IQR 1.79–1.86) and 1.36 (IQR 0.82–1.66), respectively (Figure 2b). Considered in terms of published quality control standards (*A*_260/280_ between 1.6 and 2.1, concentration ≥25 ng/μL, total yield ≥0.5 μg, and lack of degradation fragments),^11^, ^12^, ^13^, ^14^, ^15^, ^16^ all samples held for up to 9 years had yields above 0.5 μg, with 95.8% (*n* = 527) of these samples possessing *A*_260/280_ ratios within the acceptable range of 1.6–2.1.

Genomic DNA derived from 550 samples was assessed for potential degradation based on agarose gel electrophoresis. High‐molecular‐weight staining signals were evident for all 550 samples; Figure 2c presents a representative gel electrophoresis image for one sample for each year. We used Spearman ranked correlation to explore potential relationships between overall DNA yields and the duration of sample storage. Following Bonferroni correction, a robust inverse correlation was detected between blood sample storage duration and total DNA yields. This correlation was statistically significant, denoted by *r*s = −0.591, *p* < 0.0001. Thus, the extended storage period of blood samples directly correlates with diminished DNA yield during extraction. A Kruskal–Wallis *H* test indicated that statistically significant differences were present in the total DNA yield of the groups (*p* < 0.0001). Moreover, statistically significant differences were present in the *A*_260/280_ ratio of the groups (*p* < 0.0001).

### 3.2 Impact of storage time on RNA yield and integrity

Based on our analysis using a NanoDrop One instrument, the median total RNA yield was 0.86 μg (IQR 0.37–1.65). Variability in total RNA yields across different groups (i.e., sample years) was examined, with the 2012 samples having a significantly lower yield (0.13 μg) than other years (Figure 2d). RNA integrity and purity have been used in previous studies for assessing the effects of prolonged blood storage on RNA.^10^, ^25^ RNA with OD_260/280_ > 1.8 is generally acknowledged as being pure and suitable for gene expression analysis.^10^, ^26^, ^27^ In samples with a storage duration of fewer than 7 years, the OD_260/280_ for RNA was within an acceptable range. For samples stored for more than 7 years, there was a pronounced drop in the OD_260/280_ ratio, indicating adverse impacts of long‐term storage on RNA (Figure 2e).

The effect of prolonged storage on RNA integrity can be evaluated using RIN values: RIN values span from 10 (fully intact RNA) to 1 (totally degraded RNA),^28^ and RIN values above 5 suggest an acceptable RNA quality, with values above 8 considered ideal for downstream applications.^10^, ^28^ The median RIN value across the 11 groups of samples is presented in Figure 2f. RIN values decreased with increasing storage duration. We found that samples stored for 3 years (2020–2022) had a median RIN value above 6, indicating high‐quality RNA. Samples stored for 4–6 years (2017–2019) had a median RIN value over 5, indicating acceptable RNA quality. Samples stored for more than 9 years (2012 and 2013) had a median RIN value below 3, suggesting low RNA quality, unsuitable for subsequent experiments.

RNA electrophoresis is commonly used to detect RNA integrity, with an electrophoresis pattern of intact, undegraded RNA products appearing as three bands representing 18S rRNA, 28S rRNA, and 5S rRNA.^29^ Our RNA electrophoresis data analysis suggested that the integrity of RNA was compromised under prolonged storage (Figure 2g). RNA samples stored over 5 years had three distinct bands, yet the bands corresponding to 28S rRNA and 18S rRNA appeared fuzzy and weak, indicating degradation of RNA over the course of sample storage. In samples stored for over 9 years, the bands corresponding to 28S rRNA and 18S rRNA were undetectable, suggesting severe RNA degradation.

We used Spearman ranked correlation to assess potential relationships among RNA yields, the RIN value, and the period of sample storage. Following Bonferroni correction, a robust inverse correlation was detected between the duration of blood sample storage and the RIN value. This association was statistically significant (*r*s = −0.668, *p* < 0.0001). There was no evidence for correlation between blood sample storage duration and total RNA yields. A Kruskal–Wallis *H* test indicated that statistically significant differences were present in the RIN value of the groups (*p* < 0.0001). Moreover, statistically significant differences were apparent in the *A*_260/280_ values of the groups (*p* < 0.0001).

Based on the yield, RIN value, and electrophoresis results, RNA samples were divided into three grades: A, B, and C (Figure 2h). For samples stored for fewer than 3 years (2020–2022), the percentage of samples categorized as grade A exceeded 58%, while the proportion of samples classified as grade C is below 20%. For samples stored between 3 and 6 years (2017–2019), the proportion of samples categorized as grade B or higher exceeded 60%. For samples stored over 6 years, the proportion of samples classified as grade C exceeded 58%. For samples stored over 9 years, 100% of the samples were categorized as grade C.

### 3.3 Impact of storage time on serum markers

**FIGURE 3:**
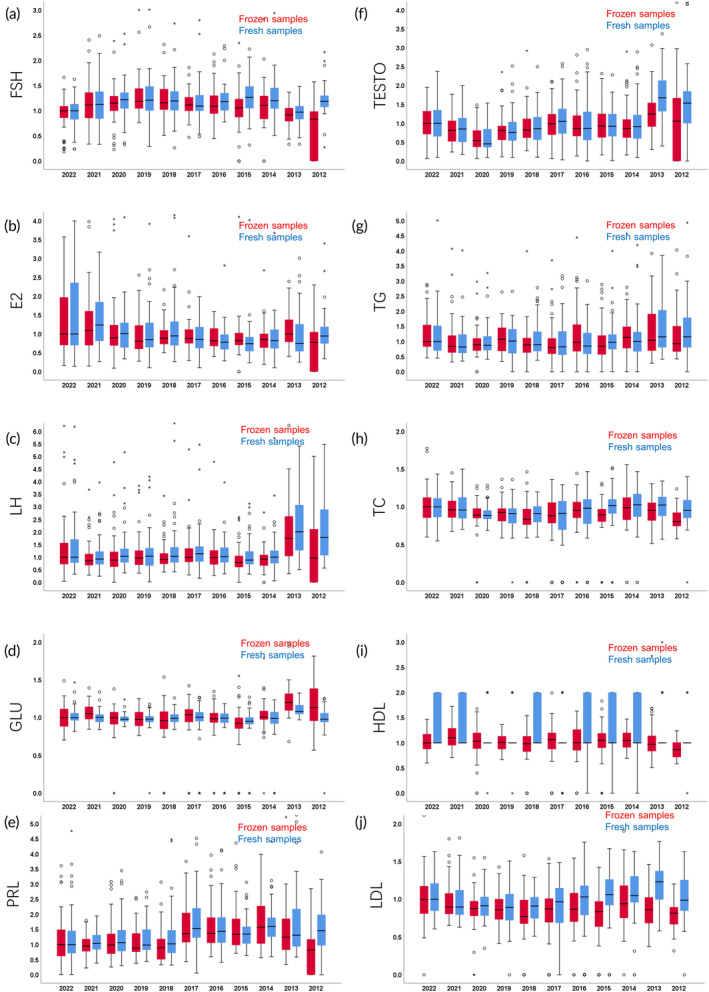
Effects of serum storage duration on FSH, E_2_ (B), LH (C), GLU (D), PRL (E), TESTO (F), TG(G), TC (H), HDL (I), and LDL (J), along with a comparison of test outcomes between varying storage durations of frozen samples and fresh samples.

Boxplots were generated to visually depict the distribution of data across the 11 groups classified by year, supporting trend detection for central tendency, spread, and skewness of the data derived from both the fresh and frozen samples (Figure 3). Comparing the median values of FSH, LH, TG, TC, HDL, and LDL for the fresh and frozen samples each year, there was a decreasing trend with increasing storage time; there was an increasing trend with time for values of the E_2_, GLU, and T levels in the frozen samples. Calculating the length of the box in each boxplot, within the same group, consistent box lengths indicates that the variability in the measurements is relatively stable between fresh and frozen samples.

We conducted repeated measures ANOVA to investigate the within‐subject effects of the storage duration across the two measurement occasions (i.e., once using fresh samples and once using frozen samples, as outlined in Table 1). This allowed us to compare the measurements between fresh and frozen samples to assess the consistency over time. Prior to the repeated measures analysis, we assessed the sphericity assumption using Mauchly's test. The results of the Mauchly's test indicated no violation of the sphericity assumption (*p* > 0.05), suggesting that the variances of the differences between all possible pairs of time points were equivalent. This allowed us to proceed with the repeated measures analysis without the application of corrections for sphericity violations. Within‐Subject Effects Analysis was performed to determine the impact of storage time on serum endocrine hormones. We detected a significant effect of storage duration on E_2_ (*p* = 0.003), LH (*p* = 0.001), PRL (*p* < 0.001), GLU (*p* < 0.001), TC (*p* < 0.001), TG (*p* = 0.017), HDL (*p* = 0.002), and LDL (*p* < 0.001) levels.

**TABLE 1:** Repeated measures variance for the impact of storage time on serum components.

	SSQ	df	MS	*F*	*p*
FSH	1.103	10	1.11	0.945	0.49
E2	246.944	10	24.694	2.686	0.003**
LH	97.745	10	9.774	6.046	0.000***
GLU	7.27	10	0.727	12.211	0.000***
PRL	59.868	10	5.987	5.66	0.000***
TESTO	52.494	10	5.249	1.021	0.423
TG	21.253	10	2.125	2.179	0.017*
TC	4.534	10	0.453	6.34	0.000***
HDL	5.18	10	0.518	2.836	0.002**
LDL	6.043	10	0.604	5.881	0.000***

*Note*: The presented table illustrates the outcomes of the analysis on within‐group effects, with data demonstrating sphericity without requiring correction. Mauchly's test revealed no sphericity violation (*p* > 0.05), indicating equivalent variances of differences between all‐time point pairs, enabling us to conduct repeated measures analysis without sphericity corrections. ***, **, and * represent significance levels of 0.1%, 1%, and 5%, respectively.

Abbreviations: df, degrees of freedom; MS, mean squares; SSQ, sum of squares.

### 3.4 Linear regression analysis

A linear regression analysis was performed to assess the potential influence of storage duration on sample detection outcomes (as outlined in Table 2); The results of an *F*‐test (*p* < 0.05) suggest that the independent variables have a statistically significant effect on the dependent variable, signifying that the overall model (including storage time and other factors) significantly explains sample detection outcomes. Analysis of collinearity showed that the variance inflation factor (VIF) was less than 10, indicating that no multicollinearity is present within the model. Therefore, the linear regression formula can be used to predict the changes in endocrine indicators within samples over storage time. The formulas for constructing the model are presented in Table 3.

**TABLE 2:** Results of linear regression analysis for the impact of storage time on serum components.

Years	Unstandardized coefficients		Standardized coefficients beta	*t*	*p*	VIF	*R* ^2^	*F*
	*B*	SE						
FSH	−0.131	0.037	−0.065	−3.566	0.000***	1.007	0.824	*F* = 1235.51, *p* = 0.000***
E2	3.603	2.867	0.053	1.257	0.209	1.012	0.061	*F* = 16.974, *p* = 0.000***
LH	−0.042	0.065	−0.017	−0.641	0.522	1.009	0.613	*F* = 418.641, *p* = 0.000***
GLU	0.059	0.01	0.218	5.667	0.000***	1.014	0.276	*F* = 94.977, *p* = 0.000***
PRL	0.178	0.133	0.042	1.335	0.182	1.041	0.498	*F* = 262.132, *p* = 0.000***
TESTO	1.317	0.213	0.259	6.168	0.000***	1.009	0.078	*F* = 22.208, *p* = 0.000***
TG	−0.023	0.011	−0.072	−2.115	0.035**	1.016	0.433	*F* = 189.536, *p* = 0.000***
TC	−0.049	0.007	−0.198	−6.781	0.000***	1.013	0.585	*F* = 347.131, *p* = 0.000***
HDL	−0.004	0.003	−0.043	−1.247	0.213	1.004	0.416	*F* = 176.201, *p* = 0.000***
LDL	−0.063	0.007	−0.32	−9.753	0.000***	1.063	0.5	*F* = 246.281, *p* = 0.000***

*Note*: Linear regression modeling utilized storage time (years), test value at collection, and test value in September 2022 to predict the impact of preservation years on endocrine data. Refer to Figure 3 for the linear regression fitting plots for each indicator. A linear regression model assumes that the overall regression coefficients are not equal to 0, indicating a regression relationship between variables. *R* ^2^ represents the goodness of fit of the regression curve. VIF values indicate the severity of multicollinearity, used to test whether the model exhibits collinearity, meaning there is a high correlation among the explanatory variables (VIF should be less than 10 or even 5, strictly speaking). *B* represents the coefficient under the constant condition, the standard error is *B*/*t*‐value, standardized coefficients are the coefficients obtained after data standardization, the *F*‐test is used to determine the presence of a significant linear relationship, and *R* ^2^ is used to evaluate the goodness of fit of the regression line to this linear model. ***, **, and * represent significance levels of 0.1%, 1%, and 5%, respectively.

**TABLE 3:** The formulas of constructing the model.

	Constant	Coefficient for storage time (years)	Coefficient for fresh sample
FSH	0.923	−0.131	0.932
E2	28.582	3.603	0.561
LH	1.001	−0.042	0.915
PRL	3.047	0.178	0.802
T	21.778	1.317	0.015
GLU	0.702	0.059	0.706
TC	1.339	−0.049	0.759
TG	0.36	−0.023	0.797
HDL	0.744	−0.004	0.425
LDL	0.975	−0.063	0.634

*Note*: The formulas of constructing the model: predicted value = constant + coefficient for storage time × YEARS + coefficient for fresh sample × fresh sample test value.

The results of the regression analysis suggest that the storage time had significant effect on the measured levels of FSH, T, GLU, TC, TG, and HDL (*p* < 0.01). In contrast, storage time did not exhibit a statistically significant impact on the measurements of E_2_, LH, PRL, and HDL.

## 4 DISCUSSION

Biobanks, serving as the foundational infrastructure for the collection and preservation of research materials, can enable advances in both basic research and translational health research. The quality of samples stored in biobanks directly impacts the reliability of subsequent experiments. Factors known to influence sample storage quality include; storage time, storage temperature, and freeze–thaw cycles. To control storage temperature and freeze–thaw cycles, equipment such as −80°C freezers and liquid nitrogen containers can be employed, along with increased aliquoting. This study aimed to investigate the impacts of long‐term storage on buffy coat and serum samples in terms of DNA, RNA, and endocrine indicator measurements. The findings of the study enhance the precision and reliability of research in fertility studies, reproductive disorders, as well as large‐scale epidemiological studies in reproductive health.

The yield and quality of the DNA extracted from extended storage samples and their suitability for downstream research has been investigated previously, including reports that samples stored at −80°C for >10 years generated high‐quality DNA for genetic analysis.^7^, ^30^ In our study, we determined that prolonged storage has minimal impact on the quality and integrity of DNA of blood samples, although we did note that prolonged storage can result in reduced DNA yields: the median yield of DNA from samples with a storage duration exceeding 6 years was lower compared to samples stored for 6 years or fewer. We therefore recommend increasing the quantity of blood samples used for DNA extraction when working with samples that have been stored for an extended period (over 6 years). This could help compensate for the potential degradation over time, and may assist in improving the ability of researchers to obtain sufficient DNA yield for downstream analyses.

Biobanks have been established to accumulate and house various biological samples for future use. RNA is a highly labile biological molecule, and we performed RNA extraction using frozen samples and evaluated the quality of samples obtained over a period of 1–10 years, assessing the purity and yield. Our findings revealed a progressive reduction in RNA integrity and purity with increasing storage duration. Notably, samples stored for over 9 years exhibited severe RNA degradation (RIN < 4.0, total yield <0.1 μg), rendering them unsuitable for meaningful experimentation. We found that RNA extracted from blood samples stored in a regular −80°C freezer remained relatively stable with respect to quantity and quality over a 5‐year frozen storage period. This finding differs from prior reports on RNA stability, for example RNA extracted from PAXgene blood RNA tubes was reported as stable for up to 6 years while being frozen.^10^, ^31^ Despite this discrepancy, our sample repository's use of blood samples beyond RNA extraction led us to choose the more common and cost‐effective −80°C storage method.

Notably, while standard −80°C storage is widely used, RNA stability in standard −80°C storage is apparently less stable than when using RNA protectants (e.g., PAXgene blood RNA tubes, RNAlater™). Our study did not find significant degradation or quality decreases in the RNA from blood samples stored for 5 years. We recommend researchers prioritize the use of blood samples within a 3‐year timeframe, as this ensures high‐quality RNA for downstream analyses, as evidenced by a median RIN value exceeding 6 in samples stored for this duration.

In summary, our study outlines the relative stability of RNA in blood samples stored in a standard −80°C method. We must carefully consider factors, including storage duration and RNA stability, when selecting samples and designing studies to ensure the reliability and validity of our experimental results. By strictly adhering to these practices and performing routine quality control assessments, we will be able to safeguard the integrity and usability of our stored samples.

The endocrine markers we investigate encompass protein hormones (FSH, LH, and PRL), steroid hormones (E_2_ and T), blood glucose, and lipid profiles (TC, TG, HDL, and LDL). No consistent pattern of level variation with storage time was found for any of the hormones or associated binding‐proteins. Some hormone and protein levels decreased and some increased with increasing storage time. However, the changes did not exceed the inter assay variations of the enzyme‐immunological tests, and the levels remained within the clinically normal range.

Previously reported findings indicated that glycoprotein hormones remained stable under short‐term storage at −70°C (about 9 months). However, under long‐term storage conditions (exceeding 2 years), variations in the stability of these hormones were observed.^32^, ^33^, ^34^, ^35^ Additionally, discrepancies emerged in the results when comparing tests conducted by different manufacturers.^32^, ^33^ On the other hand, for sex steroid hormones, there have been reports indicating that the dissociation rates of E_2_ in serum appear to increase with time in frozen serum samples.^36^, ^37^ This factor may impact measurements of the distribution of these steroids in serum. Similarly, other have shown that glucose concentrations of stored samples increased from 11.8% to 14.0% in human serum after freezing/thawing,^38^ and HDL stored at −80°C for 1 year, can significantly increase in HDL‐C levels (*p* < 0.01). Contrastingly, some reports showed the stability of serum cholesterol and triglyceride concentrations during storage in the freezer and after undergoing freeze–thaw cycles.^39^, ^40^ Therefore it this is a topic of controversy,^41^, ^42^ and more controlled studies are needed to identify a consistent trend.

Additionally, the impact of storage time on the measurement of specific endocrine markers may vary depending on the assay method.^32^, ^33^ Different antibodies may bind to different epitopes, potentially affecting the performance of older samples, such as the potential loss of terminal amino acids. In endocrine markers studies, attention should be given not only to the measurement method but also to the factor of storage time. Taking measures to control for these factors is essential for ensuring the reliability and accuracy of the research.

The Shandong University Reproductive Biobank has been in continuous operation for 16 years, providing uninterrupted samples and services for reproductive research. To ensure sample quality, we consistently adhere to the following principles. (1) standardized sample acquisition and uniform preparation of all aliquots, (2) aliquoting samples into reliable airtight tubes to prevent freeze‐drying effects during long‐term storage, (3) dual backups of samples to different devices, with regular monitoring of storage temperatures, (4) periodic quality control measures to ensure research quality.

## AUTHOR CONTRIBUTIONS

**Zhao Wang:** Data curation; formal analysis; investigation; methodology; visualization; writing – original draft. **Changming Zhang:** Investigation; resources; validation. **Xin Zhang:** Investigation; resources; validation. **Yuehong Bian:** Formal analysis; methodology; supervision; writing – review and editing. **Yongzhi Cao:** Conceptualization; funding acquisition; project administration; writing – review and editing.

## FUNDING INFORMATION

This research was supported by the National Key Research & Development Program of China (2023YFC2706405, 2021YFC2700402), National Natural Science Foundation of China (82071610).

## CONFLICT OF INTEREST STATEMENT

The authors declare no conflicts of interest.

## CONSENT FOR PUBLICATION

Written informed consent for publication of identifying images or other personal or clinical details was obtained from all of the participants. There was no participant under the age of 18.

## Supporting information


**Appendix S1:** Supporting information.

**Appendix S2:** Supporting information.

**Appendix S3:** Supporting information.

## Data Availability

All data supporting the findings of this study are available within the manuscript except for the raw sequence data. Any data providing genotype information considered to be personal property by Chinese law, hence the submission to public achieves is prohibited. The raw sequence data can be acquired upon reasonable request from the authors (yzcao@sdu.edu.cn), if approval could be granted from the Ethics Committee of Reproductive Medicine of Shandong University.

## APPENDIX A

### A.1

Tables A1, A2, A3, A4, A5, A6, A7, A8, A9, A10

**TABLE A1:**

	Unstandardized coefficients		Standardized coefficients	*t*	*p*	VIF	*R* ^2^	Adjusted *R* ^2^	*F*
	*B*	SE	Beta						
Constant	0.923	0.268	–	3.446	0.001***	–	0.824	0.823	*F* = 1235.51, *p* = 0.000***
Years	−0.131	0.037	−0.065	−3.566	0.000***	1.007			
FSH (fresh)	0.932	0.019	0.9	49.122	0.000***	1.007			

*Note*: Dependent variable: FSH (*n* = 532). *** represent significance levels of 1% respectively.

**TABLE A2:**

	Unstandardized coefficients		Standardized coefficients	*t*	*p*	VIF	*R* ^2^	Adjusted *R* ^2^	*F*
	*B*	SE	Beta						
Constant	28.582	18.97	–	1.507	0.132	–	0.061	0.057	*F* = 16.974, *p* = 0.000***
Years	3.603	2.867	0.053	1.257	0.209	1.012			
E2 (fresh)	0.561	0.097	0.246	5.793	0.000***	1.012			

*Note*: Dependent variable: E2 (*n* = 530). *** represent significance levels of 1% respectively.

**TABLE A3:**

	Unstandardized coefficients		Standardized coefficients	*t*	*p*	VIF	*R* ^2^	Adjusted *R* ^2^	*F*
	*B*	SE	Beta						
Constant	1.001	0.438	–	2.284	0.023**	–	0.613	0.611	*F* = 418.641, *p* = 0.000***
Years	−0.042	0.065	−0.017	−0.641	0.522	1.009			
LH (fresh)	0.915	0.032	0.784	28.862	0.000***	1.009			

*Note*: Dependent variable: LH (*n* = 532). ***, ** represent significance levels of 1% and 5%, respectively.

**TABLE A4:**

	Unstandardized coefficients		Standardized coefficients	*t*	*p*	VIF	*R* ^2^	Adjusted *R* ^2^	*F*
	*B*	SE	Beta						
Constant	3.047	0.908	–	3.355	0.001***	–	0.498	0.496	*F* = 262.132, *p* = 0.000***
Years	0.178	0.133	0.042	1.335	0.182	1.041			
PRL (fresh)	0.802	0.036	0.696	22.142	0.000***	1.041			

*Note*: Dependent variable: PRL (*n* = 531). *** represent significance levels of 1% respectively.

**TABLE A5:**

	Unstandardized coefficients		Standardized coefficients	*t*	*p*	VIF	*R* ^2^	Adjusted *R* ^2^	*F*
	*B*	SE	Beta						
Constant	21.778	1.292	–	16.852	0.000***	–	0.078	0.074	*F* = 22.208, *p* = 0.000***
Years	1.317	0.213	0.259	6.168	0.000***	1.009			
TESTO (fresh)	0.015	0.008	0.081	1.917	0.056*	1.009			

*Note*: Dependent variable: TESTO (*n* = 531). ***, * represent significance levels of 1% and 10%, respectively.

**TABLE A6:**

	Unstandardized coefficients		Standardized coefficients	*t*	*p*	VIF	*R* ^2^	Adjusted *R* ^2^	*F*
	*B*	SE	Beta						
Constant	0.702	0.323	–	2.176	0.030**	–	0.276	0.273	*F* = 94.977, *p* = 0.000***
Years	0.059	0.01	0.218	5.667	0.000***	1.014			
GLU (fresh)	0.706	0.06	0.453	11.804	0.000***	1.014			

*Note*: Dependent variable: GLU (*n* = 501). ***, ** represent significance levels of 1% and 5%, respectively.

**TABLE A7:**

	Unstandardized coefficients		Standardized coefficients	*t*	*p*	VIF	*R* ^2^	Adjusted *R* ^2^	*F*
	*B*	SE	Beta						
Constant	1.339	0.128	–	10.443	0.000***	–	0.585	0.583	*F* = 347.131, *p* = 0.000***
Years	−0.049	0.007	−0.198	−6.781	0.000***	1.013			
TC (fresh)	0.759	0.029	0.761	26.06	0.000***	1.013			

*Note*: Dependent variable: TC (*n* = 496). *** represent significance levels of 1% respectively.

**TABLE A8:**

	Unstandardized coefficients		Standardized coefficients	*t*	*p*	VIF	*R* ^2^	Adjusted *R* ^2^	*F*
	*B*	SE	Beta						
Constant	0.36	0.079	–	4.567	0.000***	–	0.433	0.431	*F* = 189.536, *p* = 0.000***
Years	−0.023	0.011	−0.072	−2.115	0.035**	1.016			
TG (fresh)	0.797	0.041	0.663	19.467	0.000***	1.016			

*Note*: Dependent variable: TG (*n* = 499). ***, ** represent significance levels of 1% and 5%, respectively.

**TABLE A9:**

	Unstandardized coefficients		Standardized coefficients	*t*	*p*	VIF	*R* ^2^	Adjusted *R* ^2^	*F*
	*B*	SE	Beta						
Constant	0.744	0.036	–	20.883	0.000***	–	0.416	0.414	*F* = 176.201, *p* = 0.000***
Years	−0.004	0.003	−0.043	−1.247	0.213	1.004			
HDL (fresh)	0.425	0.023	0.641	18.607	0.000***	1.004			

*Note*: Dependent variable: HDL (*n* = 498). *** represent significance levels of 1% respectively.

**TABLE A10:**

	Unstandardized coefficients		Standardized coefficients	*t*	*p*	VIF	*R* ^2^	Adjusted *R* ^2^	*F*
	*B*	SE	Beta						
Constant	0.975	0.082	–	11.913	0.000***	–	0.5	0.498	*F* = 246.281, *p* = 0.000***
Years	−0.063	0.007	−0.32	−9.753	0.000***	1.063			
LDL (fresh)	0.634	0.029	0.713	21.71	0.000***	1.063			

*Note*: Dependent variable: LDL (*n* = 496). *** represent significance levels of 1% respectively.
