# Supplementary material for: Assessing the impact of long‐term storage on the quality and integrity of biological specimens in a reproductive biobank
Source: Bioeng Transl Med. 2024 Jun 26;9(6):e10692. doi: 10.1002/btm2.10692 (PMC11558192; doi:10.1002/btm2.10692)
Supplement: Supplementary file 1 — Appendix S1: Supporting information. [file BTM2-9-e10692-s001.docx]

**DNA Sample Quality Inspection Report**

| **Number** | 549 | **SAMPLE TYPES** | DNA |
| --- | --- | --- | --- |
| **Quantitative Method** | **Nanodrop one** | | |
| **Quality Inspection Method** | **Agarose Electrophoresis** | | |

| **The name of the reagent** | / | **Batch Number** | / |
| --- | --- | --- | --- |
| \| \| **order** \| **Name** \| **concentration (ng/μL)** \| **volume (μL)** \| **total (μg)** \| **A260/ A280** \| **A260/ A230** \| **Conclusion** \| \| **Remarks** \| \| --- \| --- \| --- \| --- \| --- \| --- \| --- \| --- \| --- \| --- \| \| **Quality** \| **total** \| \| 1-1 \| B1 \| 10.8 \| 20 \| 0.22 \| 1.78 \| 0.59 \| A \| / \| / \| \| 1-2 \| B2 \| 31.8 \| 20 \| 0.64 \| 1.78 \| 1.10 \| A \| / \| / \| \| 1-3 \| B3 \| 8.0 \| 20 \| 0.16 \| 1.94 \| 0.54 \| A \| / \| / \| \| 1-4 \| B4 \| 9.2 \| 20 \| 0.18 \| 1.73 \| 0.50 \| A \| / \| / \| \| 1-5 \| B5 \| 5.3 \| 20 \| 0.11 \| 1.93 \| 0.40 \| A \| / \| / \| \| 1-6 \| B6 \| 11.6 \| 20 \| 0.23 \| 1.70 \| 0.60 \| A \| / \| / \| \| 1-7 \| B7 \| 8.0 \| 20 \| 0.16 \| 1.67 \| 0.53 \| A \| / \| / \| \| 1-8 \| B8 \| 6.4 \| 20 \| 0.13 \| 1.71 \| 0.47 \| A \| / \| / \| \| 1-9 \| B9 \| 5.0 \| 20 \| 0.10 \| 2.02 \| 0.31 \| A \| / \| / \| \| 1-10 \| B10 \| 8.2 \| 20 \| 0.16 \| 1.77 \| 0.51 \| A \| / \| / \| \| 1-11 \| B11 \| 5.4 \| 20 \| 0.11 \| 1.65 \| 0.33 \| A \| / \| / \| \| 1-12 \| B12 \| 7.9 \| 20 \| 0.16 \| 1.64 \| 0.58 \| A \| / \| / \| \| 1-13 \| B13 \| 3.5 \| 20 \| 0.07 \| 2.09 \| 0.26 \| A \| / \| / \| \| 1-14 \| B14 \| 3.8 \| 20 \| 0.08 \| 1.63 \| 0.23 \| A \| / \| / \| \| 1-15 \| B15 \| 8.3 \| 20 \| 0.17 \| 1.76 \| 0.49 \| A \| / \| / \| \| 1-16 \| B16 \| 6.5 \| 20 \| 0.13 \| 1.76 \| 0.46 \| A \| / \| / \| \| 1-17 \| B17 \| 7.8 \| 20 \| 0.16 \| 1.84 \| 0.55 \| A \| / \| / \| \| 1-18 \| B18 \| 5.8 \| 20 \| 0.12 \| 1.73 \| 0.36 \| A \| / \| / \| \| 1-19 \| B19 \| 6.0 \| 20 \| 0.12 \| 1.61 \| 0.45 \| A \| / \| / \| \| 1-20 \| B20 \| 6.3 \| 20 \| 0.13 \| 1.85 \| 0.42 \| A \| / \| / \| \| 1-21 \| B21 \| 5.1 \| 20 \| 0.10 \| 1.97 \| 0.37 \| A \| / \| / \| \| 1-22 \| B22 \| 3.5 \| 20 \| 0.07 \| 1.82 \| 0.24 \| A \| / \| / \| \| 1-23 \| B23 \| 8.9 \| 20 \| 0.18 \| 1.80 \| 0.49 \| A \| / \| / \| \| 1-24 \| B24 \| 6.0 \| 20 \| 0.12 \| 1.98 \| 0.39 \| A \| / \| / \| \| 1-25 \| B25 \| 18.8 \| 20 \| 0.38 \| 1.82 \| 0.93 \| A \| / \| / \| \| 1-26 \| B26 \| 5.5 \| 20 \| 0.11 \| 1.63 \| 0.35 \| A \| / \| / \| \| 1-27 \| B27 \| 9.1 \| 20 \| 0.18 \| 2.00 \| 0.68 \| A \| / \| / \| \| 1-28 \| B28 \| 3.1 \| 20 \| 0.06 \| 1.89 \| 0.27 \| A \| / \| / \| \| 1-29 \| B29 \| 9.3 \| 20 \| 0.19 \| 2.07 \| 0.57 \| A \| / \| / \| \| 1-30 \| B30 \| 3.4 \| 20 \| 0.07 \| 2.02 \| 0.22 \| A \| / \| / \| \| 1-31 \| B31 \| 4.4 \| 20 \| 0.09 \| 1.91 \| 0.29 \| A \| / \| / \| \| 1-32 \| B32 \| 4.6 \| 20 \| 0.09 \| 1.87 \| 0.39 \| A \| / \| / \| \| 1-33 \| B33 \| 5.9 \| 20 \| 0.12 \| 1.68 \| 0.40 \| A \| / \| / \| \| 1-34 \| B34 \| 10.6 \| 20 \| 0.21 \| 1.83 \| 0.55 \| A \| / \| / \| \| 1-35 \| B35 \| 11.1 \| 20 \| 0.22 \| 1.78 \| 0.66 \| A \| / \| / \| \| 1-36 \| B36 \| 8.3 \| 20 \| 0.17 \| 1.86 \| 0.57 \| A \| / \| / \| \| 1-37 \| B37 \| 3.9 \| 20 \| 0.08 \| 1.88 \| 0.29 \| A \| / \| / \| \| 1-38 \| B38 \| 8.2 \| 20 \| 0.16 \| 1.86 \| 0.65 \| A \| / \| / \| \| 1-39 \| B39 \| 8.0 \| 20 \| 0.16 \| 1.64 \| 0.51 \| A \| / \| / \| \| 1-40 \| B40 \| 4.5 \| 20 \| 0.09 \| 1.46 \| 0.26 \| A \| / \| / \| \| 1-41 \| B41 \| 8.5 \| 20 \| 0.17 \| 1.66 \| 0.45 \| A \| / \| / \| \| 1-42 \| B42 \| 14.8 \| 20 \| 0.30 \| 1.78 \| 0.71 \| A \| / \| / \| \| 1-43 \| B43 \| 9.9 \| 20 \| 0.20 \| 1.97 \| 0.62 \| A \| / \| / \| \| 1-44 \| B44 \| 7.0 \| 20 \| 0.14 \| 2.04 \| 0.50 \| A \| / \| / \| \| 1-45 \| B45 \| 6.5 \| 20 \| 0.13 \| 1.78 \| 0.47 \| A \| / \| / \| \| 1-46 \| B46 \| 10.0 \| 20 \| 0.20 \| 1.70 \| 0.54 \| A \| / \| / \| \| 1-47 \| B47 \| 5.1 \| 20 \| 0.10 \| 1.64 \| 0.40 \| A \| / \| / \| \| 1-48 \| B48 \| 8.3 \| 20 \| 0.17 \| 1.60 \| 0.47 \| A \| / \| / \| \| 1-49 \| B49 \| 12.4 \| 20 \| 0.25 \| 1.72 \| 0.52 \| A \| / \| / \| \| 1-50 \| B50 \| 6.4 \| 20 \| 0.13 \| 1.92 \| 0.43 \| A \| / \| / \| \| 1-51 \| B51 \| 11.3 \| 20 \| 0.23 \| 1.82 \| 0.66 \| A \| / \| / \| \| 1-52 \| B52 \| 18.2 \| 20 \| 0.36 \| 1.65 \| 0.61 \| A \| / \| / \| \| 1-53 \| B53 \| 17.8 \| 20 \| 0.36 \| 1.77 \| 0.95 \| A \| / \| / \| \| 1-54 \| B54 \| 11.9 \| 20 \| 0.24 \| 1.74 \| 0.68 \| A \| / \| / \| \| 1-55 \| B55 \| 14.5 \| 20 \| 0.29 \| 1.82 \| 0.80 \| A \| / \| / \| \| 1-56 \| B56 \| 6.4 \| 20 \| 0.13 \| 1.56 \| 0.37 \| A \| / \| / \| \| 1-57 \| B57 \| 13.1 \| 20 \| 0.26 \| 1.78 \| 0.67 \| A \| / \| / \| \| 1-58 \| B58 \| 31.4 \| 20 \| 0.63 \| 1.93 \| 1.03 \| A \| / \| / \| \| 1-59 \| B59 \| 20.5 \| 20 \| 0.41 \| 1.64 \| 0.72 \| A \| / \| / \| \| 1-60 \| B60 \| 16.8 \| 20 \| 0.34 \| 1.80 \| 0.93 \| A \| / \| / \| \| 1-61 \| B61 \| 14.4 \| 20 \| 0.29 \| 1.86 \| 0.76 \| A \| / \| / \| \| 1-62 \| B62 \| 9.0 \| 20 \| 0.18 \| 1.62 \| 0.48 \| A \| / \| / \| \| 1-63 \| B63 \| 10.3 \| 20 \| 0.21 \| 1.79 \| 0.77 \| A \| / \| / \| \| 1-64 \| B64 \| 11.8 \| 20 \| 0.24 \| 1.68 \| 0.60 \| A \| / \| / \| \| 1-65 \| B65 \| 13.3 \| 20 \| 0.27 \| 1.77 \| 0.66 \| A \| / \| / \| \| 1-66 \| B66 \| 12.8 \| 20 \| 0.26 \| 1.66 \| 0.66 \| A \| / \| / \| \| 1-67 \| B67 \| 16.2 \| 20 \| 0.32 \| 1.81 \| 0.76 \| A \| / \| / \| \| 1-68 \| B68 \| 23.9 \| 20 \| 0.48 \| 1.89 \| 1.09 \| A \| / \| / \| \| 1-69 \| B69 \| 15.2 \| 20 \| 0.30 \| 1.75 \| 0.87 \| A \| / \| / \| \| 1-70 \| B70 \| 13.4 \| 20 \| 0.27 \| 1.73 \| 0.87 \| A \| / \| / \| \| 1-71 \| B71 \| 7.7 \| 20 \| 0.15 \| 1.55 \| 0.46 \| A \| / \| / \| \| 1-72 \| B72 \| 3.8 \| 20 \| 0.08 \| 1.49 \| 0.26 \| A \| / \| / \| \| 1-73 \| B73 \| 11.8 \| 20 \| 0.24 \| 1.74 \| 0.63 \| A \| / \| / \| \| 1-74 \| B74 \| 11.7 \| 20 \| 0.23 \| 1.66 \| 0.57 \| A \| / \| / \| \| 1-75 \| B75 \| 7.8 \| 20 \| 0.16 \| 1.79 \| 0.50 \| A \| / \| / \| \| 1-76 \| B76 \| 26.7 \| 20 \| 0.53 \| 1.87 \| 1.14 \| A \| / \| / \| \| 1-77 \| B77 \| 8.5 \| 20 \| 0.17 \| 1.65 \| 0.61 \| A \| / \| / \| \| 1-78 \| B78 \| 28.3 \| 20 \| 0.57 \| 1.85 \| 1.26 \| A \| / \| / \| \| 1-79 \| B79 \| 7.6 \| 20 \| 0.15 \| 1.62 \| 0.45 \| A \| / \| / \| \| 1-80 \| B80 \| 5.7 \| 20 \| 0.11 \| 1.58 \| 0.34 \| A \| / \| / \| \| 1-81 \| B81 \| 6.3 \| 20 \| 0.13 \| 1.40 \| 0.37 \| A \| / \| / \| \| 1-82 \| B82 \| 10.3 \| 20 \| 0.21 \| 1.74 \| 0.69 \| A \| / \| / \| \| 1-83 \| B83 \| 8.3 \| 20 \| 0.17 \| 1.66 \| 0.65 \| A \| / \| / \| \| 1-84 \| B84 \| 3.7 \| 20 \| 0.07 \| 1.43 \| 0.27 \| A \| / \| / \| \| 1-85 \| B85 \| 14.5 \| 20 \| 0.29 \| 1.43 \| 0.50 \| A \| / \| / \| \| 1-86 \| B86 \| 7.7 \| 20 \| 0.15 \| 1.59 \| 0.52 \| A \| / \| / \| \| 1-87 \| B87 \| 8.1 \| 20 \| 0.16 \| 1.65 \| 0.47 \| A \| / \| / \| \| 1-88 \| B88 \| 10.0 \| 20 \| 0.20 \| 1.65 \| 0.69 \| A \| / \| / \| \| 1-89 \| B89 \| 4.9 \| 20 \| 0.10 \| 1.55 \| 0.24 \| A \| / \| / \| \| 1-90 \| B90 \| 13.6 \| 20 \| 0.27 \| 1.74 \| 0.65 \| A \| / \| / \| \| 1-91 \| B91 \| 9.8 \| 20 \| 0.20 \| 1.76 \| 0.62 \| A \| / \| / \| \| 1-92 \| B92 \| 6.8 \| 20 \| 0.14 \| 1.49 \| 0.43 \| A \| / \| / \| \| 1-93 \| B93 \| 16.7 \| 20 \| 0.33 \| 1.79 \| 0.78 \| A \| / \| / \| \| 1-94 \| B94 \| 9.9 \| 20 \| 0.20 \| 1.66 \| 0.51 \| A \| / \| / \| \| 1-95 \| B95 \| 10.6 \| 20 \| 0.21 \| 1.73 \| 0.55 \| A \| / \| / \| \| 1-96 \| B96 \| 26.3 \| 20 \| 0.53 \| 1.75 \| 1.02 \| A \| / \| / \| \| 2-1 \| B97 \| 6.1 \| 20 \| 0.12 \| 1.37 \| 0.27 \| A \| / \| / \| \| 2-2 \| B98 \| 44.2 \| 20 \| 0.88 \| 1.81 \| 1.26 \| A \| / \| / \| \| 2-3 \| B99 \| 7.3 \| 20 \| 0.15 \| 1.59 \| 0.43 \| A \| / \| / \| \| 2-4 \| B100 \| 4.1 \| 20 \| 0.08 \| 1.36 \| 0.19 \| A \| / \| / \| \| 2-5 \| B101 \| 26.8 \| 20 \| 0.54 \| 1.69 \| 0.79 \| A \| / \| / \| \| 2-6 \| B102 \| 36.3 \| 20 \| 0.73 \| 1.85 \| 1.02 \| A \| / \| / \| \| 2-7 \| B103 \| 42.4 \| 20 \| 0.85 \| 1.91 \| 1.01 \| A \| / \| / \| \| 2-8 \| B104 \| 23.4 \| 20 \| 0.47 \| 1.81 \| 0.72 \| A \| / \| / \| \| 2-9 \| B105 \| 20.0 \| 20 \| 0.40 \| 1.75 \| 0.69 \| A \| / \| / \| \| 2-10 \| B106 \| 89.5 \| 20 \| 1.79 \| 1.99 \| 1.79 \| A \| / \| / \| \| 2-11 \| B107 \| 15.4 \| 20 \| 0.31 \| 1.80 \| 0.83 \| A \| / \| / \| \| 2-12 \| B108 \| 27.6 \| 20 \| 0.55 \| 1.89 \| 1.11 \| A \| / \| / \| \| 2-13 \| B109 \| 42.3 \| 20 \| 0.85 \| 1.92 \| 1.33 \| A \| / \| / \| \| 2-14 \| B110 \| 46.1 \| 20 \| 0.92 \| 1.98 \| 1.36 \| A \| / \| / \| \| 2-15 \| B111 \| 7.8 \| 20 \| 0.16 \| 1.95 \| 0.43 \| A \| / \| / \| \| 2-16 \| B112 \| 18.8 \| 20 \| 0.38 \| 1.83 \| 1.02 \| A \| / \| / \| \| 2-17 \| B113 \| 11.8 \| 20 \| 0.24 \| 1.65 \| 0.56 \| A \| / \| / \| \| 2-18 \| B114 \| 26.3 \| 20 \| 0.53 \| 1.87 \| 1.21 \| A \| / \| / \| \| 2-19 \| B115 \| 34.8 \| 20 \| 0.70 \| 1.83 \| 1.24 \| A \| / \| / \| \| 2-20 \| B116 \| 54.8 \| 20 \| 1.10 \| 1.89 \| 1.52 \| A \| / \| / \| \| 2-21 \| B117 \| 41.3 \| 20 \| 0.83 \| 1.89 \| 1.42 \| A \| / \| / \| \| 2-22 \| B118 \| 54.0 \| 20 \| 1.08 \| 1.86 \| 1.49 \| A \| / \| / \| \| 2-23 \| B119 \| 27.6 \| 20 \| 0.55 \| 1.82 \| 0.71 \| A \| / \| / \| \| 2-24 \| B120 \| 21.0 \| 20 \| 0.42 \| 1.78 \| 0.94 \| A \| / \| / \| \| 2-25 \| B121 \| 39.3 \| 20 \| 0.79 \| 1.76 \| 1.37 \| A \| / \| / \| \| 2-26 \| B122 \| 64.9 \| 20 \| 1.30 \| 1.80 \| 1.16 \| A \| / \| / \| \| 2-27 \| B123 \| 48.1 \| 20 \| 0.96 \| 1.81 \| 1.47 \| A \| / \| / \| \| 2-28 \| B124 \| 115.2 \| 20 \| 2.30 \| 1.98 \| 1.83 \| A \| / \| / \| \| 2-29 \| B125 \| 44.4 \| 20 \| 0.89 \| 1.82 \| 1.52 \| A \| / \| / \| \| 2-30 \| B126 \| 7.9 \| 20 \| 0.16 \| 1.50 \| 0.41 \| A \| / \| / \| \| 2-31 \| B127 \| 20.6 \| 20 \| 0.41 \| 1.64 \| 0.77 \| A \| / \| / \| \| 2-32 \| B128 \| 13.0 \| 20 \| 0.26 \| 1.45 \| 0.69 \| A \| / \| / \| \| 2-33 \| B129 \| 91.6 \| 20 \| 1.83 \| 1.93 \| 1.67 \| A \| / \| / \| \| 2-34 \| B130 \| 63.8 \| 20 \| 1.28 \| 1.94 \| 1.61 \| A \| / \| / \| \| 2-35 \| B131 \| 59.2 \| 20 \| 1.18 \| 1.92 \| 1.58 \| A \| / \| / \| \| 2-36 \| B132 \| 48.3 \| 20 \| 0.97 \| 1.93 \| 1.50 \| A \| / \| / \| \| 2-37 \| B133 \| 67.2 \| 20 \| 1.34 \| 1.90 \| 1.54 \| A \| / \| / \| \| 2-38 \| B134 \| 95.8 \| 20 \| 1.92 \| 1.97 \| 1.73 \| A \| / \| / \| \| 2-39 \| B135 \| 42.8 \| 20 \| 0.86 \| 1.82 \| 1.36 \| A \| / \| / \| \| 2-40 \| B136 \| 96.0 \| 20 \| 1.92 \| 1.90 \| 1.77 \| A \| / \| / \| \| 2-41 \| B137 \| 86.5 \| 20 \| 1.73 \| 1.86 \| 1.69 \| A \| / \| / \| \| 2-42 \| B138 \| 123.4 \| 20 \| 2.47 \| 1.96 \| 1.91 \| A \| / \| / \| \| 2-43 \| B139 \| 97.4 \| 20 \| 1.95 \| 1.98 \| 1.97 \| A \| / \| / \| \| 2-44 \| B140 \| 27.0 \| 20 \| 0.54 \| 1.96 \| 1.28 \| A \| / \| / \| \| 2-45 \| B141 \| 91.8 \| 20 \| 1.84 \| 1.90 \| 1.70 \| A \| / \| / \| \| 2-46 \| B142 \| 56.4 \| 20 \| 1.13 \| 1.88 \| 1.67 \| A \| / \| / \| \| 2-47 \| B143 \| 39.4 \| 20 \| 0.79 \| 1.80 \| 1.38 \| A \| / \| / \| \| 2-48 \| B144 \| 93.4 \| 20 \| 1.87 \| 1.91 \| 1.73 \| A \| / \| / \| \| 2-49 \| B145 \| 24.3 \| 20 \| 0.49 \| 1.71 \| 0.91 \| A \| / \| / \| \| 2-50 \| B146 \| 12.8 \| 20 \| 0.26 \| 1.74 \| 0.74 \| A \| / \| / \| \| 2-51 \| B147 \| 29.1 \| 20 \| 0.58 \| 2.00 \| 1.24 \| A \| / \| / \| \| 2-52 \| B148 \| 61.2 \| 20 \| 1.22 \| 1.92 \| 1.67 \| A \| / \| / \| \| 2-53 \| B149 \| 7.4 \| 20 \| 0.15 \| 1.35 \| 0.18 \| A \| / \| / \| \| 2-54 \| B150 \| 75.6 \| 20 \| 1.51 \| 1.93 \| 1.64 \| A \| / \| / \| \| 2-55 \| B151 \| 58.8 \| 20 \| 1.18 \| 1.91 \| 1.62 \| A \| / \| / \| \| 2-56 \| B152 \| 44.2 \| 20 \| 0.88 \| 1.90 \| 1.46 \| A \| / \| / \| \| 2-57 \| B153 \| 68.3 \| 20 \| 1.37 \| 1.96 \| 1.77 \| A \| / \| / \| \| 2-58 \| B154 \| 113.5 \| 20 \| 2.27 \| 1.95 \| 1.82 \| A \| / \| / \| \| 2-59 \| B155 \| 74.5 \| 20 \| 1.49 \| 1.92 \| 1.69 \| A \| / \| / \| \| 2-60 \| B156 \| 36.8 \| 20 \| 0.74 \| 1.86 \| 1.33 \| A \| / \| / \| \| 2-61 \| B157 \| 63.1 \| 20 \| 1.26 \| 1.89 \| 1.56 \| A \| / \| / \| \| 2-62 \| B158 \| 26.0 \| 20 \| 0.52 \| 1.67 \| 0.99 \| A \| / \| / \| \| 2-63 \| B159 \| 47.8 \| 20 \| 0.96 \| 1.89 \| 1.45 \| A \| / \| / \| \| 2-64 \| B160 \| 33.6 \| 20 \| 0.67 \| 1.83 \| 1.22 \| A \| / \| / \| \| 2-65 \| B161 \| 39.7 \| 20 \| 0.79 \| 1.86 \| 1.39 \| A \| / \| / \| \| 2-66 \| B162 \| 50.0 \| 20 \| 1.00 \| 1.75 \| 1.09 \| A \| / \| / \| \| 2-67 \| B163 \| 73.4 \| 20 \| 1.47 \| 1.85 \| 1.74 \| A \| / \| / \| \| 2-68 \| B164 \| 60.0 \| 20 \| 1.20 \| 2.01 \| 1.66 \| A \| / \| / \| \| 2-69 \| B165 \| 65.2 \| 20 \| 1.30 \| 1.96 \| 1.59 \| A \| / \| / \| \| 2-70 \| B166 \| 44.5 \| 20 \| 0.89 \| 1.80 \| 1.53 \| A \| / \| / \| \| 2-71 \| B167 \| 24.5 \| 20 \| 0.49 \| 1.68 \| 0.92 \| A \| / \| / \| \| 2-72 \| B168 \| 10.5 \| 20 \| 0.21 \| 1.63 \| 0.49 \| A \| / \| / \| \| 2-73 \| B169 \| 22.2 \| 20 \| 0.44 \| 1.73 \| 0.92 \| A \| / \| / \| \| 2-74 \| B170 \| 127.2 \| 20 \| 2.54 \| 1.94 \| 1.83 \| A \| / \| / \| \| 2-75 \| B171 \| 49.4 \| 20 \| 0.99 \| 1.82 \| 1.47 \| A \| / \| / \| \| 2-76 \| B172 \| 68.0 \| 20 \| 1.36 \| 1.93 \| 1.67 \| A \| / \| / \| \| 2-77 \| B173 \| 80.2 \| 20 \| 1.60 \| 1.92 \| 1.75 \| A \| / \| / \| \| 2-78 \| B174 \| 29.2 \| 20 \| 0.58 \| 1.85 \| 1.16 \| A \| / \| / \| \| 2-79 \| B175 \| 24.3 \| 20 \| 0.49 \| 1.72 \| 0.99 \| A \| / \| / \| \| 2-80 \| B176 \| 48.4 \| 20 \| 0.97 \| 1.87 \| 1.33 \| A \| / \| / \| \| 2-81 \| B177 \| 48.8 \| 20 \| 0.98 \| 1.86 \| 1.52 \| A \| / \| / \| \| 2-82 \| B178 \| 7.9 \| 20 \| 0.16 \| 1.53 \| 0.55 \| A \| / \| / \| \| 2-83 \| B179 \| 9.1 \| 20 \| 0.18 \| 1.57 \| 0.55 \| A \| / \| / \| \| 2-84 \| B180 \| 25.0 \| 20 \| 0.50 \| 1.68 \| 1.06 \| A \| / \| / \| \| 2-85 \| B181 \| 9.2 \| 20 \| 0.18 \| 1.63 \| 0.66 \| A \| / \| / \| \| 2-86 \| B182 \| 6.2 \| 20 \| 0.12 \| 1.43 \| 0.44 \| A \| / \| / \| \| 2-87 \| B183 \| 9.4 \| 20 \| 0.19 \| 1.55 \| 0.55 \| A \| / \| / \| \| 2-88 \| B184 \| 64.0 \| 20 \| 1.28 \| 1.91 \| 1.48 \| A \| / \| / \| \| 2-89 \| B185 \| 71.6 \| 20 \| 1.43 \| 1.80 \| 1.62 \| A \| / \| / \| \| 2-90 \| B186 \| 40.6 \| 20 \| 0.81 \| 1.79 \| 1.42 \| A \| / \| / \| \| 2-91 \| B187 \| 22.8 \| 20 \| 0.46 \| 1.74 \| 1.17 \| A \| / \| / \| \| 2-92 \| B188 \| 79.8 \| 20 \| 1.60 \| 1.91 \| 1.51 \| A \| / \| / \| \| 2-93 \| B189 \| 173.0 \| 20 \| 3.46 \| 1.99 \| 1.89 \| A \| / \| / \| \| 2-94 \| B190 \| 42.5 \| 20 \| 0.85 \| 1.85 \| 1.26 \| A \| / \| / \| \| 2-95 \| B191 \| 40.8 \| 20 \| 0.82 \| 1.79 \| 1.26 \| A \| / \| / \| \| 2-96 \| B192 \| 50.8 \| 20 \| 1.02 \| 1.90 \| 1.30 \| A \| / \| / \| \| 3-1 \| B193 \| 93.3 \| 20 \| 1.87 \| 1.96 \| 1.59 \| A \| / \| / \| \| 3-2 \| B194 \| 94.8 \| 20 \| 1.90 \| 1.95 \| 1.47 \| A \| / \| / \| \| 3-3 \| B195 \| 16.0 \| 20 \| 0.32 \| 1.72 \| 0.74 \| A \| / \| / \| \| 3-4 \| B196 \| 17.9 \| 20 \| 0.36 \| 1.82 \| 0.74 \| A \| / \| / \| \| 3-5 \| B197 \| 93.5 \| 20 \| 1.87 \| 2.00 \| 1.45 \| A \| / \| / \| \| 3-6 \| B198 \| 30.4 \| 20 \| 0.61 \| 1.87 \| 0.74 \| A \| / \| / \| \| 3-7 \| B199 \| 9.4 \| 20 \| 0.19 \| 1.63 \| 0.34 \| A \| / \| / \| \| 3-8 \| B200 \| 74.1 \| 20 \| 1.48 \| 1.80 \| 1.51 \| A \| / \| / \| \| 3-9 \| B201 \| 21.9 \| 20 \| 0.44 \| 1.71 \| 1.22 \| A \| / \| / \| \| 3-10 \| B202 \| 62.7 \| 20 \| 1.25 \| 1.94 \| 1.19 \| A \| / \| / \| \| 3-11 \| B203 \| 58.1 \| 20 \| 1.16 \| 1.89 \| 1.23 \| A \| / \| / \| \| 3-12 \| B204 \| 55.1 \| 20 \| 1.10 \| 1.84 \| 1.23 \| A \| / \| / \| \| 3-13 \| B205 \| 97.4 \| 20 \| 1.95 \| 1.94 \| 1.54 \| A \| / \| / \| \| 3-14 \| B206 \| 145.3 \| 20 \| 2.91 \| 1.96 \| 1.55 \| A \| / \| / \| \| 3-15 \| B207 \| 9.8 \| 20 \| 0.20 \| 1.68 \| 0.38 \| A \| / \| / \| \| 3-16 \| B208 \| 85.5 \| 20 \| 1.71 \| 1.93 \| 1.39 \| A \| / \| / \| \| 3-17 \| B209 \| 94.6 \| 20 \| 1.89 \| 1.88 \| 1.36 \| A \| / \| / \| \| 3-18 \| B210 \| 81.3 \| 20 \| 1.63 \| 1.87 \| 1.37 \| A \| / \| / \| \| 3-19 \| B211 \| 28.2 \| 20 \| 0.56 \| 1.85 \| 1.03 \| A \| / \| / \| \| 3-20 \| B212 \| 253.4 \| 20 \| 5.07 \| 1.95 \| 1.84 \| A \| / \| / \| \| 3-21 \| B213 \| 143.7 \| 20 \| 2.87 \| 1.95 \| 1.66 \| A \| / \| / \| \| 3-22 \| B214 \| 24.9 \| 20 \| 0.50 \| 1.77 \| 0.71 \| A \| / \| / \| \| 3-23 \| B215 \| 45.1 \| 20 \| 0.90 \| 1.88 \| 1.03 \| A \| / \| / \| \| 3-24 \| B216 \| 49.6 \| 20 \| 0.99 \| 1.91 \| 1.33 \| A \| / \| / \| \| 3-25 \| B217 \| 114.8 \| 20 \| 2.30 \| 1.83 \| 1.68 \| A \| / \| / \| \| 3-26 \| B218 \| 157.3 \| 20 \| 3.15 \| 1.94 \| 1.71 \| A \| / \| / \| \| 3-27 \| B219 \| 50.8 \| 20 \| 1.02 \| 1.91 \| 1.39 \| A \| / \| / \| \| 3-28 \| B220 \| 52.4 \| 20 \| 1.05 \| 1.87 \| 1.20 \| A \| / \| / \| \| 3-29 \| B221 \| 39.9 \| 20 \| 0.80 \| 1.87 \| 1.26 \| A \| / \| / \| \| 3-30 \| B222 \| 64.0 \| 20 \| 1.28 \| 1.96 \| 1.45 \| A \| / \| / \| \| 3-31 \| B223 \| 53.2 \| 20 \| 1.06 \| 1.90 \| 1.27 \| A \| / \| / \| \| 3-32 \| B224 \| 17.8 \| 20 \| 0.36 \| 1.70 \| 0.82 \| A \| / \| / \| \| 3-33 \| B225 \| 797.2 \| 20 \| 15.94 \| 1.89 \| 2.29 \| A \| / \| / \| \| 3-34 \| B226 \| 53.1 \| 20 \| 1.06 \| 1.80 \| 1.37 \| A \| / \| / \| \| 3-35 \| B227 \| 45.0 \| 20 \| 0.90 \| 1.90 \| 1.52 \| A \| / \| / \| \| 3-36 \| B228 \| 24.1 \| 20 \| 0.48 \| 1.78 \| 1.09 \| A \| / \| / \| \| 3-37 \| B229 \| 155.4 \| 20 \| 3.11 \| 1.94 \| 1.85 \| A \| / \| / \| \| 3-38 \| B230 \| 88.8 \| 20 \| 1.78 \| 1.91 \| 1.76 \| A \| / \| / \| \| 3-39 \| B231 \| 52.2 \| 20 \| 1.04 \| 1.89 \| 1.50 \| A \| / \| / \| \| 3-40 \| B232 \| 109.0 \| 20 \| 2.18 \| 1.95 \| 1.87 \| A \| / \| / \| \| 3-41 \| B233 \| 151.8 \| 20 \| 3.04 \| 1.87 \| 1.98 \| A \| / \| / \| \| 3-42 \| B234 \| 218.8 \| 20 \| 4.38 \| 1.97 \| 1.99 \| A \| / \| / \| \| 3-43 \| B235 \| 36.4 \| 20 \| 0.73 \| 1.78 \| 1.43 \| A \| / \| / \| \| 3-44 \| B236 \| 65.7 \| 20 \| 1.31 \| 1.88 \| 1.57 \| A \| / \| / \| \| 3-45 \| B237 \| 47.9 \| 20 \| 0.96 \| 1.75 \| 1.22 \| A \| / \| / \| \| 3-46 \| B238 \| 77.0 \| 20 \| 1.54 \| 1.92 \| 1.69 \| A \| / \| / \| \| 3-47 \| B239 \| 44.8 \| 20 \| 0.90 \| 1.89 \| 1.41 \| A \| / \| / \| \| 3-48 \| B240 \| 85.9 \| 20 \| 1.72 \| 1.92 \| 1.72 \| A \| / \| / \| \| 3-49 \| B241 \| 83.8 \| 20 \| 1.68 \| 1.79 \| 1.62 \| A \| / \| / \| \| 3-50 \| B242 \| 43.1 \| 20 \| 0.86 \| 1.76 \| 1.40 \| A \| / \| / \| \| 3-51 \| B243 \| 184.2 \| 20 \| 3.68 \| 1.92 \| 1.86 \| A \| / \| / \| \| 3-52 \| B244 \| 73.1 \| 20 \| 1.46 \| 1.92 \| 1.65 \| A \| / \| / \| \| 3-53 \| B245 \| 133.8 \| 20 \| 2.68 \| 1.88 \| 1.87 \| A \| / \| / \| \| 3-54 \| B246 \| 68.5 \| 20 \| 1.37 \| 1.90 \| 1.61 \| A \| / \| / \| \| 3-55 \| B247 \| 88.1 \| 20 \| 1.76 \| 1.91 \| 1.72 \| A \| / \| / \| \| 3-56 \| B248 \| 176.9 \| 20 \| 3.54 \| 1.95 \| 2.00 \| A \| / \| / \| \| 3-57 \| B249 \| 66.7 \| 20 \| 1.33 \| 1.82 \| 1.80 \| A \| / \| / \| \| 3-58 \| B250 \| 74.0 \| 20 \| 1.48 \| 1.91 \| 1.70 \| A \| / \| / \| \| 3-59 \| B251 \| 37.0 \| 20 \| 0.74 \| 1.95 \| 1.40 \| A \| / \| / \| \| 3-60 \| B252 \| 12.7 \| 20 \| 0.25 \| 1.68 \| 0.82 \| A \| / \| / \| \| 3-61 \| B253 \| 67.7 \| 20 \| 1.35 \| 1.98 \| 1.70 \| A \| / \| / \| \| 3-62 \| B254 \| 144.6 \| 20 \| 2.89 \| 1.92 \| 1.94 \| A \| / \| / \| \| 3-63 \| B255 \| 54.2 \| 20 \| 1.08 \| 1.93 \| 1.56 \| A \| / \| / \| \| 3-64 \| B256 \| 20.8 \| 20 \| 0.42 \| 1.82 \| 0.99 \| A \| / \| / \| \| 3-65 \| B257 \| 13.4 \| 20 \| 0.27 \| 1.76 \| 0.74 \| A \| / \| / \| \| 3-66 \| B258 \| 9.3 \| 20 \| 0.19 \| 1.63 \| 0.64 \| A \| / \| / \| \| 3-67 \| B259 \| 119.2 \| 20 \| 2.38 \| 1.95 \| 1.69 \| A \| / \| / \| \| 3-68 \| B260 \| 51.2 \| 20 \| 1.02 \| 1.88 \| 1.56 \| A \| / \| / \| \| 3-69 \| B261 \| 60.4 \| 20 \| 1.21 \| 1.94 \| 1.58 \| A \| / \| / \| \| 3-70 \| B262 \| 12.8 \| 20 \| 0.26 \| 1.72 \| 0.87 \| A \| / \| / \| \| 3-71 \| B263 \| 313.7 \| 20 \| 6.27 \| 2.02 \| 2.08 \| A \| / \| / \| \| 3-72 \| B264 \| 68.1 \| 20 \| 1.36 \| 1.91 \| 1.69 \| A \| / \| / \| \| 3-73 \| B265 \| 19.1 \| 20 \| 0.38 \| 1.84 \| 0.95 \| A \| / \| / \| \| 3-74 \| B266 \| 70.4 \| 20 \| 1.41 \| 1.88 \| 1.65 \| A \| / \| / \| \| 3-75 \| B267 \| 65.1 \| 20 \| 1.30 \| 2.01 \| 1.71 \| A \| / \| / \| \| 3-76 \| B268 \| 10.9 \| 20 \| 0.22 \| 1.83 \| 0.79 \| A \| / \| / \| \| 3-77 \| B269 \| 75.6 \| 20 \| 1.51 \| 1.94 \| 1.71 \| A \| / \| / \| \| 3-78 \| B270 \| 49.1 \| 20 \| 0.98 \| 1.86 \| 1.60 \| A \| / \| / \| \| 3-79 \| B271 \| 129.9 \| 20 \| 2.60 \| 1.91 \| 1.85 \| A \| / \| / \| \| 3-80 \| B272 \| 180.3 \| 20 \| 3.61 \| 2.03 \| 2.00 \| A \| / \| / \| \| 3-81 \| B273 \| 19.2 \| 20 \| 0.38 \| 1.81 \| 0.76 \| A \| / \| / \| \| 3-82 \| B274 \| 32.6 \| 20 \| 0.65 \| 1.90 \| 1.41 \| A \| / \| / \| \| 3-83 \| B275 \| 62.9 \| 20 \| 1.26 \| 2.01 \| 1.71 \| A \| / \| / \| \| 3-84 \| B276 \| 261.8 \| 20 \| 5.24 \| 1.97 \| 2.09 \| A \| / \| / \| \| 3-85 \| B277 \| 146.1 \| 20 \| 2.92 \| 1.91 \| 1.87 \| A \| / \| / \| \| 3-86 \| B278 \| 76.9 \| 20 \| 1.54 \| 1.90 \| 1.77 \| A \| / \| / \| \| 3-87 \| B279 \| 75.8 \| 20 \| 1.52 \| 1.85 \| 1.84 \| A \| / \| / \| \| 3-88 \| B280 \| 94.7 \| 20 \| 1.89 \| 2.00 \| 1.81 \| A \| / \| / \| \| 3-89 \| B281 \| 88.4 \| 20 \| 1.77 \| 1.98 \| 1.56 \| A \| / \| / \| \| 3-90 \| B282 \| 20.8 \| 20 \| 0.42 \| 1.73 \| 1.09 \| A \| / \| / \| \| 3-91 \| B283 \| 132.0 \| 20 \| 2.64 \| 1.92 \| 1.95 \| A \| / \| / \| \| 3-92 \| B284 \| 48.6 \| 20 \| 0.97 \| 1.81 \| 1.83 \| A \| / \| / \| \| 3-93 \| B285 \| 73.5 \| 20 \| 1.47 \| 1.86 \| 1.58 \| A \| / \| / \| \| 3-94 \| B286 \| 75.6 \| 20 \| 1.51 \| 1.77 \| 1.41 \| A \| / \| / \| \| 3-95 \| B287 \| 104.2 \| 20 \| 2.08 \| 1.82 \| 2.10 \| A \| / \| / \| \| 3-96 \| B288 \| 23.4 \| 20 \| 0.47 \| 1.79 \| 0.94 \| A \| / \| / \| \| 4-1 \| B289 \| 95.6 \| 20 \| 1.91 \| 1.86 \| 1.55 \| A \| / \| / \| \| 4-2 \| B290 \| 94.9 \| 20 \| 1.90 \| 1.87 \| 1.46 \| A \| / \| / \| \| 4-3 \| B291 \| 183.8 \| 20 \| 3.68 \| 1.84 \| 1.74 \| A \| / \| / \| \| 4-4 \| B292 \| 53.8 \| 20 \| 1.08 \| 1.84 \| 1.14 \| A \| / \| / \| \| 4-5 \| B293 \| 231.4 \| 20 \| 4.63 \| 1.84 \| 1.61 \| A \| / \| / \| \| 4-6 \| B294 \| 79.4 \| 20 \| 1.59 \| 1.93 \| 1.28 \| A \| / \| / \| \| 4-7 \| B295 \| 256.8 \| 20 \| 5.14 \| 1.93 \| 1.76 \| A \| / \| / \| \| 4-8 \| B296 \| 49.0 \| 20 \| 0.98 \| 1.92 \| 0.97 \| A \| / \| / \| \| 4-9 \| B297 \| 165.4 \| 20 \| 3.31 \| 1.85 \| 1.88 \| A \| / \| / \| \| 4-10 \| B298 \| 67.7 \| 20 \| 1.35 \| 1.94 \| 1.64 \| A \| / \| / \| \| 4-11 \| B299 \| 23.6 \| 20 \| 0.47 \| 2.07 \| 1.19 \| A \| / \| / \| \| 4-12 \| B300 \| 13.7 \| 20 \| 0.27 \| 1.94 \| 1.12 \| A \| / \| / \| \| 4-13 \| B301 \| 185.4 \| 20 \| 3.71 \| 1.71 \| 1.27 \| A \| / \| / \| \| 4-14 \| B302 \| 320.0 \| 20 \| 6.40 \| 1.84 \| 2.27 \| A \| / \| / \| \| 4-15 \| B303 \| 30.0 \| 20 \| 0.60 \| 1.90 \| 1.21 \| A \| / \| / \| \| 4-16 \| B304 \| 44.4 \| 20 \| 0.89 \| 1.93 \| 0.95 \| A \| / \| / \| \| 4-17 \| B305 \| 145.6 \| 20 \| 2.91 \| 1.99 \| 1.93 \| A \| / \| / \| \| 4-18 \| B306 \| 97.9 \| 20 \| 1.96 \| 1.84 \| 1.87 \| A \| / \| / \| \| 4-19 \| B307 \| 51.0 \| 20 \| 1.02 \| 1.88 \| 1.65 \| A \| / \| / \| \| 4-20 \| B308 \| 41.3 \| 20 \| 0.83 \| 1.80 \| 1.48 \| A \| / \| / \| \| 4-21 \| B309 \| 31.9 \| 20 \| 0.64 \| 1.81 \| 1.41 \| A \| / \| / \| \| 4-22 \| B310 \| 20.3 \| 20 \| 0.41 \| 1.86 \| 1.20 \| A \| / \| / \| \| 4-23 \| B312 \| 12.2 \| 20 \| 0.24 \| 1.82 \| 0.81 \| A \| / \| / \| \| 4-24 \| B313 \| 96.1 \| 20 \| 1.92 \| 1.84 \| 1.47 \| A \| / \| / \| \| 4-25 \| B314 \| 107.1 \| 20 \| 2.14 \| 1.90 \| 1.81 \| A \| / \| / \| \| 4-26 \| B315 \| 46.2 \| 20 \| 0.92 \| 1.81 \| 1.44 \| A \| / \| / \| \| 4-27 \| B316 \| 98.9 \| 20 \| 1.98 \| 1.82 \| 1.55 \| A \| / \| / \| \| 4-28 \| B317 \| 58.5 \| 20 \| 1.17 \| 1.98 \| 1.71 \| A \| / \| / \| \| 4-29 \| B318 \| 30.6 \| 20 \| 0.61 \| 1.85 \| 1.50 \| A \| / \| / \| \| 4-30 \| B319 \| 32.9 \| 20 \| 0.66 \| 1.86 \| 1.48 \| A \| / \| / \| \| 4-31 \| B320 \| 123.1 \| 20 \| 2.46 \| 1.86 \| 1.66 \| A \| / \| / \| \| 4-32 \| B321 \| 54.1 \| 20 \| 1.08 \| 1.77 \| 1.18 \| A \| / \| / \| \| 4-33 \| B322 \| 31.6 \| 20 \| 0.63 \| 1.95 \| 1.33 \| A \| / \| / \| \| 4-34 \| B323 \| 12.7 \| 20 \| 0.25 \| 1.86 \| 0.88 \| A \| / \| / \| \| 4-35 \| B324 \| 60.7 \| 20 \| 1.21 \| 1.95 \| 1.68 \| A \| / \| / \| \| 4-36 \| B325 \| 26.1 \| 20 \| 0.52 \| 1.96 \| 1.40 \| A \| / \| / \| \| 4-37 \| B326 \| 83.0 \| 20 \| 1.66 \| 1.91 \| 1.79 \| A \| / \| / \| \| 4-38 \| B327 \| 52.7 \| 20 \| 1.05 \| 1.93 \| 1.73 \| A \| / \| / \| \| 4-39 \| B328 \| 75.5 \| 20 \| 1.51 \| 1.83 \| 1.75 \| A \| / \| / \| \| 4-40 \| B329 \| 54.8 \| 20 \| 1.10 \| 1.81 \| 1.36 \| A \| / \| / \| \| 4-41 \| B330 \| 96.8 \| 20 \| 1.94 \| 1.77 \| 1.41 \| A \| / \| / \| \| 4-42 \| B331 \| 32.1 \| 20 \| 0.64 \| 1.86 \| 1.32 \| A \| / \| / \| \| 4-43 \| B332 \| 89.1 \| 20 \| 1.78 \| 2.00 \| 1.76 \| A \| / \| / \| \| 4-44 \| B333 \| 21.0 \| 20 \| 0.42 \| 1.82 \| 1.20 \| A \| / \| / \| \| 4-45 \| B334 \| 34.6 \| 20 \| 0.69 \| 1.84 \| 1.57 \| A \| / \| / \| \| 4-46 \| B335 \| 19.6 \| 20 \| 0.39 \| 1.78 \| 1.24 \| A \| / \| / \| \| 4-47 \| B336 \| 51.2 \| 20 \| 1.02 \| 1.90 \| 1.67 \| A \| / \| / \| \| 4-48 \| B337 \| 36.4 \| 20 \| 0.73 \| 1.80 \| 1.22 \| A \| / \| / \| \| 4-49 \| B338 \| 77.8 \| 20 \| 1.56 \| 1.94 \| 1.68 \| A \| / \| / \| \| 4-50 \| B339 \| 27.8 \| 20 \| 0.56 \| 1.86 \| 1.37 \| A \| / \| / \| \| 4-51 \| B340 \| 109.3 \| 20 \| 2.19 \| 1.86 \| 1.73 \| A \| / \| / \| \| 4-52 \| B341 \| 94.2 \| 20 \| 1.88 \| 1.80 \| 1.62 \| A \| / \| / \| \| 4-53 \| B342 \| 149.1 \| 20 \| 2.98 \| 1.82 \| 1.95 \| A \| / \| / \| \| 4-54 \| B343 \| 41.9 \| 20 \| 0.84 \| 1.90 \| 1.50 \| A \| / \| / \| \| 4-55 \| B344 \| 37.7 \| 20 \| 0.75 \| 1.84 \| 1.53 \| A \| / \| / \| \| 4-56 \| B345 \| 20.0 \| 20 \| 0.40 \| 1.72 \| 0.83 \| A \| / \| / \| \| 4-57 \| B346 \| 58.9 \| 20 \| 1.18 \| 1.89 \| 1.56 \| A \| / \| / \| \| 4-58 \| B347 \| 18.8 \| 20 \| 0.38 \| 1.85 \| 1.02 \| A \| / \| / \| \| 4-59 \| B348 \| 52.3 \| 20 \| 1.05 \| 1.84 \| 1.66 \| A \| / \| / \| \| 4-60 \| B349 \| 60.2 \| 20 \| 1.20 \| 1.98 \| 1.72 \| A \| / \| / \| \| 4-61 \| B350 \| 38.0 \| 20 \| 0.76 \| 1.94 \| 1.57 \| A \| / \| / \| \| 4-62 \| B351 \| 62.6 \| 20 \| 1.25 \| 1.82 \| 1.85 \| A \| / \| / \| \| 4-63 \| B352 \| 18.7 \| 20 \| 0.37 \| 1.67 \| 0.97 \| A \| / \| / \| \| 4-64 \| B353 \| 26.3 \| 20 \| 0.53 \| 1.85 \| 0.99 \| A \| / \| / \| \| 4-65 \| B354 \| 134.3 \| 20 \| 2.69 \| 1.84 \| 1.59 \| A \| / \| / \| \| 4-66 \| B355 \| 57.8 \| 20 \| 1.16 \| 1.80 \| 1.69 \| A \| / \| / \| \| 4-67 \| B356 \| 71.4 \| 20 \| 1.43 \| 1.95 \| 1.81 \| A \| / \| / \| \| 4-68 \| B357 \| 67.3 \| 20 \| 1.35 \| 1.84 \| 2.08 \| A1 \| / \| / \| \| 4-69 \| B358 \| 142.0 \| 20 \| 2.84 \| 2.00 \| 1.98 \| A \| / \| / \| \| 4-70 \| B359 \| 291.4 \| 20 \| 5.83 \| 1.87 \| 2.23 \| A \| / \| / \| \| 4-71 \| B360 \| 243.2 \| 20 \| 4.86 \| 1.84 \| 2.11 \| A \| / \| / \| \| 4-72 \| B361 \| 61.6 \| 20 \| 1.23 \| 1.92 \| 1.45 \| A \| / \| / \| \| 4-73 \| B362 \| 201.7 \| 20 \| 4.03 \| 1.82 \| 1.86 \| A \| / \| / \| \| 4-74 \| B363 \| 59.5 \| 20 \| 1.19 \| 1.98 \| 1.66 \| A \| / \| / \| \| 4-75 \| B364 \| 85.5 \| 20 \| 1.71 \| 1.90 \| 1.84 \| A \| / \| / \| \| 4-76 \| B365 \| 74.0 \| 20 \| 1.48 \| 1.99 \| 1.74 \| A \| / \| / \| \| 4-77 \| B366 \| 57.7 \| 20 \| 1.15 \| 1.93 \| 1.65 \| A \| / \| / \| \| 4-78 \| B367 \| 26.0 \| 20 \| 0.52 \| 1.87 \| 1.38 \| A \| / \| / \| \| 4-79 \| B368 \| 68.1 \| 20 \| 1.36 \| 1.90 \| 1.76 \| A \| / \| / \| \| 4-80 \| B369 \| 89.8 \| 20 \| 1.80 \| 1.94 \| 1.61 \| A \| / \| / \| \| 4-81 \| B370 \| 90.6 \| 20 \| 1.81 \| 1.88 \| 1.83 \| A \| / \| / \| \| 4-82 \| B371 \| 76.1 \| 20 \| 1.52 \| 1.84 \| 1.66 \| A \| / \| / \| \| 4-83 \| B372 \| 141.4 \| 20 \| 2.83 \| 1.96 \| 1.91 \| A \| / \| / \| \| 4-84 \| B373 \| 72.1 \| 20 \| 1.44 \| 1.89 \| 1.74 \| A \| / \| / \| \| 4-85 \| B374 \| 62.1 \| 20 \| 1.24 \| 1.91 \| 1.69 \| A \| / \| / \| \| 4-86 \| B375 \| 86.3 \| 20 \| 1.73 \| 1.97 \| 1.89 \| A \| / \| / \| \| 4-87 \| B376 \| 131.5 \| 20 \| 2.63 \| 1.86 \| 1.96 \| A \| / \| / \| \| 4-88 \| B377 \| 38.6 \| 20 \| 0.77 \| 1.84 \| 1.22 \| A \| / \| / \| \| 4-89 \| B378 \| 146.8 \| 20 \| 2.94 \| 1.87 \| 2.01 \| A \| / \| / \| \| 4-90 \| B379 \| 93.1 \| 20 \| 1.86 \| 1.87 \| 1.86 \| A \| / \| / \| \| 4-91 \| B380 \| 78.1 \| 20 \| 1.56 \| 1.83 \| 1.65 \| A \| / \| / \| \| 4-92 \| B381 \| 140.4 \| 20 \| 2.81 \| 1.90 \| 1.86 \| A \| / \| / \| \| 4-93 \| B382 \| 93.7 \| 20 \| 1.87 \| 1.82 \| 1.73 \| A \| / \| / \| \| 4-94 \| B383 \| 77.6 \| 20 \| 1.55 \| 1.84 \| 1.70 \| A \| / \| / \| \| 4-95 \| B384 \| 151.1 \| 20 \| 3.02 \| 1.93 \| 1.89 \| A \| / \| / \| \| 4-96 \| B385 \| 150.5 \| 20 \| 3.01 \| 1.83 \| 1.82 \| A \| / \| / \| \| 5-1 \| B386 \| 131.2 \| 20 \| 2.62 \| 1.84 \| 1.75 \| A \| / \| / \| \| 5-2 \| B387 \| 247.1 \| 20 \| 4.94 \| 1.84 \| 2.07 \| A \| / \| / \| \| 5-3 \| B388 \| 43.9 \| 20 \| 0.88 \| 1.86 \| 1.34 \| A \| / \| / \| \| 5-4 \| B389 \| 91.8 \| 20 \| 1.84 \| 1.90 \| 1.64 \| A \| / \| / \| \| 5-5 \| B390 \| 33.8 \| 20 \| 0.68 \| 1.96 \| 1.11 \| A \| / \| / \| \| 5-6 \| B391 \| 56.9 \| 20 \| 1.14 \| 1.94 \| 1.40 \| A \| / \| / \| \| 5-7 \| B392 \| 28.9 \| 20 \| 0.58 \| 1.92 \| 0.95 \| A \| / \| / \| \| 5-8 \| B393 \| 122.1 \| 20 \| 2.44 \| 1.90 \| 1.65 \| A \| / \| / \| \| 5-9 \| B394 \| 22.2 \| 20 \| 0.44 \| 1.86 \| 1.02 \| A \| / \| / \| \| 5-10 \| B395 \| 34.3 \| 20 \| 0.69 \| 1.82 \| 1.19 \| A \| / \| / \| \| 5-11 \| B396 \| 30.3 \| 20 \| 0.61 \| 1.85 \| 1.25 \| A \| / \| / \| \| 5-12 \| B397 \| 15.8 \| 20 \| 0.32 \| 1.82 \| 0.96 \| A \| / \| / \| \| 5-13 \| B398 \| 68.4 \| 20 \| 1.37 \| 1.84 \| 1.68 \| A \| / \| / \| \| 5-14 \| B399 \| 118.4 \| 20 \| 2.37 \| 1.93 \| 1.88 \| A \| / \| / \| \| 5-15 \| B400 \| 60.1 \| 20 \| 1.20 \| 1.91 \| 1.68 \| A \| / \| / \| \| 5-16 \| B401 \| 31.2 \| 20 \| 0.62 \| 1.93 \| 1.29 \| A \| / \| / \| \| 5-17 \| B402 \| 54.0 \| 20 \| 1.08 \| 2.00 \| 1.60 \| A \| / \| / \| \| 5-18 \| B403 \| 11.1 \| 20 \| 0.22 \| 1.83 \| 0.75 \| A \| / \| / \| \| 5-19 \| B404 \| 40.0 \| 20 \| 0.80 \| 1.96 \| 1.51 \| A \| / \| / \| \| 5-20 \| B405 \| 5.5 \| 20 \| 0.11 \| 1.72 \| 0.42 \| A \| / \| / \| \| 5-21 \| B406 \| 18.6 \| 20 \| 0.37 \| 1.93 \| 1.05 \| A \| / \| / \| \| 5-22 \| B407 \| 11.8 \| 20 \| 0.24 \| 1.92 \| 0.81 \| A \| / \| / \| \| 5-23 \| B408 \| 28.2 \| 20 \| 0.56 \| 1.91 \| 1.25 \| A \| / \| / \| \| 5-24 \| B409 \| 36.1 \| 20 \| 0.72 \| 1.99 \| 1.39 \| A \| / \| / \| \| 5-25 \| B410 \| 58.9 \| 20 \| 1.18 \| 1.98 \| 1.58 \| A \| / \| / \| \| 5-26 \| B411 \| 7.9 \| 20 \| 0.16 \| 1.89 \| 0.55 \| C \| / \| / \| \| 5-27 \| B412 \| 74.7 \| 20 \| 1.49 \| 1.98 \| 1.78 \| A \| / \| / \| \| 5-28 \| B413 \| 24.8 \| 20 \| 0.50 \| 1.92 \| 1.24 \| A \| / \| / \| \| 5-29 \| B414 \| 31.7 \| 20 \| 0.63 \| 1.95 \| 1.35 \| A \| / \| / \| \| 5-30 \| B415 \| 33.4 \| 20 \| 0.67 \| 2.01 \| 1.45 \| A \| / \| / \| \| 5-31 \| B416 \| 14.5 \| 20 \| 0.29 \| 1.85 \| 0.87 \| A \| / \| / \| \| 5-32 \| B417 \| 12.4 \| 20 \| 0.25 \| 1.91 \| 0.78 \| A \| / \| / \| \| 5-33 \| B418 \| 56.8 \| 20 \| 1.14 \| 1.97 \| 1.55 \| A \| / \| / \| \| 5-34 \| B419 \| 23.9 \| 20 \| 0.48 \| 1.96 \| 1.19 \| A \| / \| / \| \| 5-35 \| B420 \| 22.3 \| 20 \| 0.45 \| 2.02 \| 1.17 \| A \| / \| / \| \| 5-36 \| B421 \| 25.6 \| 20 \| 0.51 \| 1.97 \| 1.20 \| A \| / \| / \| \| 5-37 \| B422 \| 74.5 \| 20 \| 1.49 \| 2.01 \| 1.79 \| A \| / \| / \| \| 5-38 \| B423 \| 126.9 \| 20 \| 2.54 \| 1.83 \| 2.07 \| A \| / \| / \| \| 5-39 \| B424 \| 5.5 \| 20 \| 0.11 \| 1.54 \| 0.47 \| A \| / \| / \| \| 5-40 \| B425 \| 9.6 \| 20 \| 0.19 \| 1.77 \| 0.60 \| A \| / \| / \| \| 5-41 \| B426 \| 50.0 \| 20 \| 1.00 \| 2.03 \| 1.51 \| A \| / \| / \| \| 5-42 \| B427 \| 12.3 \| 20 \| 0.25 \| 2.13 \| 0.82 \| A \| / \| / \| \| 5-43 \| B428 \| 12.9 \| 20 \| 0.26 \| 1.87 \| 0.87 \| A \| / \| / \| \| 5-44 \| B429 \| 14.1 \| 20 \| 0.28 \| 2.03 \| 1.01 \| A \| / \| / \| \| 5-45 \| B430 \| 15.3 \| 20 \| 0.31 \| 1.91 \| 0.96 \| A \| / \| / \| \| 5-46 \| B431 \| 13.8 \| 20 \| 0.28 \| 1.85 \| 1.00 \| A \| / \| / \| \| 5-47 \| B432 \| 14.6 \| 20 \| 0.29 \| 1.78 \| 0.94 \| A \| / \| / \| \| 5-48 \| B433 \| 31.9 \| 20 \| 0.64 \| 1.91 \| 1.31 \| A \| / \| / \| \| 5-49 \| B434 \| 11.7 \| 20 \| 0.23 \| 1.82 \| 0.73 \| A \| / \| / \| \| 5-50 \| B435 \| 8.3 \| 20 \| 0.17 \| 2.02 \| 0.76 \| A \| / \| / \| \| 5-51 \| B436 \| 10.3 \| 20 \| 0.21 \| 1.85 \| 0.76 \| A \| / \| / \| \| 5-52 \| B437 \| 15.1 \| 20 \| 0.30 \| 1.95 \| 0.99 \| A \| / \| / \| \| 5-53 \| B438 \| 7.9 \| 20 \| 0.16 \| 1.75 \| 0.63 \| A \| / \| / \| \| 5-54 \| B439 \| 22.5 \| 20 \| 0.45 \| 1.87 \| 1.22 \| A \| / \| / \| \| 5-55 \| B440 \| 104.4 \| 20 \| 2.09 \| 1.99 \| 1.83 \| A \| / \| / \| \| 5-56 \| B441 \| 38.0 \| 20 \| 0.76 \| 1.98 \| 1.40 \| A \| / \| / \| \| 5-57 \| B442 \| 57.5 \| 20 \| 1.15 \| 1.97 \| 1.55 \| A \| / \| / \| \| 5-58 \| B443 \| 11.0 \| 20 \| 0.22 \| 1.82 \| 0.69 \| A \| / \| / \| \| 5-59 \| B444 \| 28.7 \| 20 \| 0.57 \| 1.99 \| 1.27 \| A \| / \| / \| \| 5-60 \| B445 \| 48.4 \| 20 \| 0.97 \| 1.99 \| 1.62 \| A \| / \| / \| \| 5-61 \| B446 \| 66.5 \| 20 \| 1.33 \| 2.01 \| 1.74 \| A \| / \| / \| \| 5-62 \| B447 \| 57.7 \| 20 \| 1.15 \| 1.97 \| 1.65 \| A \| / \| / \| \| 5-63 \| B448 \| 13.5 \| 20 \| 0.27 \| 1.80 \| 0.92 \| A \| / \| / \| \| 5-64 \| B449 \| 57.5 \| 20 \| 1.15 \| 1.97 \| 1.64 \| A \| / \| / \| \| 5-65 \| B450 \| 14.3 \| 20 \| 0.29 \| 1.89 \| 0.95 \| A \| / \| / \| \| 5-66 \| B451 \| 9.3 \| 20 \| 0.19 \| 1.65 \| 0.58 \| A \| / \| / \| \| 5-67 \| B452 \| 22.7 \| 20 \| 0.45 \| 1.70 \| 1.15 \| A \| / \| / \| \| 5-68 \| B453 \| 28.3 \| 20 \| 0.57 \| 1.77 \| 1.27 \| A \| / \| / \| \| 5-69 \| B454 \| 25.8 \| 20 \| 0.52 \| 1.70 \| 1.17 \| A \| / \| / \| \| 5-70 \| B455 \| 28.5 \| 20 \| 0.57 \| 1.76 \| 1.21 \| A \| / \| / \| \| 5-71 \| B456 \| 58.6 \| 20 \| 1.17 \| 1.79 \| 1.56 \| A \| / \| / \| \| 5-72 \| B457 \| 45.9 \| 20 \| 0.92 \| 1.81 \| 1.46 \| A \| / \| / \| \| 5-73 \| B458 \| 18.3 \| 20 \| 0.37 \| 1.78 \| 0.86 \| A \| / \| / \| \| 5-74 \| B459 \| 47.7 \| 20 \| 0.95 \| 1.88 \| 1.37 \| A \| / \| / \| \| 5-75 \| B460 \| 5.6 \| 20 \| 0.11 \| 1.76 \| 0.49 \| A \| / \| / \| \| 5-76 \| B461 \| 49.6 \| 20 \| 0.99 \| 1.83 \| 1.52 \| A \| / \| / \| \| 5-77 \| B462 \| 20.7 \| 20 \| 0.41 \| 1.72 \| 1.01 \| A \| / \| / \| \| 5-78 \| B463 \| 19.5 \| 20 \| 0.39 \| 1.73 \| 0.92 \| A \| / \| / \| \| 5-79 \| B464 \| 20.1 \| 20 \| 0.40 \| 1.78 \| 0.98 \| A \| / \| / \| \| 5-80 \| B465 \| 45.2 \| 20 \| 0.90 \| 1.82 \| 1.40 \| A \| / \| / \| \| 5-81 \| B466 \| 17.0 \| 20 \| 0.34 \| 1.67 \| 0.73 \| A \| / \| / \| \| 5-82 \| B467 \| 9.0 \| 20 \| 0.18 \| 1.83 \| 0.68 \| A \| / \| / \| \| 5-83 \| B468 \| 52.5 \| 20 \| 1.05 \| 1.79 \| 1.57 \| A \| / \| / \| \| 5-84 \| B469 \| 146.8 \| 20 \| 2.94 \| 1.96 \| 1.90 \| A \| / \| / \| \| 5-85 \| B470 \| 35.6 \| 20 \| 0.71 \| 1.83 \| 1.28 \| A \| / \| / \| \| 5-86 \| B471 \| 10.7 \| 20 \| 0.21 \| 1.74 \| 0.75 \| A \| / \| / \| \| 5-87 \| B472 \| 35.1 \| 20 \| 0.70 \| 1.84 \| 1.34 \| A \| / \| / \| \| 5-88 \| B473 \| 41.6 \| 20 \| 0.83 \| 1.87 \| 1.50 \| A \| / \| / \| \| 5-89 \| B474 \| 41.8 \| 20 \| 0.84 \| 1.77 \| 1.26 \| A \| / \| / \| \| 5-90 \| B475 \| 90.1 \| 20 \| 1.80 \| 1.86 \| 1.68 \| A \| / \| / \| \| 5-91 \| B476 \| 37.4 \| 20 \| 0.75 \| 1.80 \| 1.24 \| A \| / \| / \| \| 5-92 \| B477 \| 62.7 \| 20 \| 1.25 \| 1.81 \| 1.56 \| A \| / \| / \| \| 5-93 \| B478 \| 77.1 \| 20 \| 1.54 \| 1.93 \| 1.61 \| A \| / \| / \| \| 5-94 \| B479 \| 86.9 \| 20 \| 1.74 \| 1.86 \| 1.60 \| A \| / \| / \| \| 5-95 \| B480 \| 30.4 \| 20 \| 0.61 \| 1.76 \| 1.15 \| A \| / \| / \| \| 5-96 \| B481 \| 23.2 \| 20 \| 0.46 \| 1.72 \| 0.99 \| A \| / \| / \| \| 6-1 \| B482 \| 43.5 \| 20 \| 0.87 \| 1.81 \| 1.42 \| A \| / \| / \| \| 6-2 \| B483 \| 158.9 \| 20 \| 3.18 \| 2.00 \| 1.95 \| A \| / \| / \| \| 6-3 \| B484 \| 55.2 \| 20 \| 1.10 \| 1.79 \| 1.52 \| A \| / \| / \| \| 6-4 \| B485 \| 146.8 \| 20 \| 2.94 \| 1.89 \| 1.88 \| A \| / \| / \| \| 6-5 \| B486 \| 105.7 \| 20 \| 2.11 \| 1.80 \| 1.96 \| A \| / \| / \| \| 6-6 \| B487 \| 130.7 \| 20 \| 2.61 \| 1.86 \| 2.00 \| A \| / \| / \| \| 6-7 \| B488 \| 42.0 \| 20 \| 0.84 \| 1.83 \| 1.47 \| A \| / \| / \| \| 6-8 \| B489 \| 128.1 \| 20 \| 2.56 \| 1.85 \| 1.79 \| A \| / \| / \| \| 6-9 \| B490 \| 53.5 \| 20 \| 1.07 \| 1.81 \| 1.61 \| A \| / \| / \| \| 6-10 \| B491 \| 20.5 \| 20 \| 0.41 \| 1.73 \| 0.96 \| A \| / \| / \| \| 6-11 \| B492 \| 91.1 \| 20 \| 1.82 \| 1.86 \| 1.52 \| A \| / \| / \| \| 6-12 \| B493 \| 23.9 \| 20 \| 0.48 \| 1.82 \| 1.08 \| A \| / \| / \| \| 6-13 \| B494 \| 61.9 \| 20 \| 1.24 \| 1.88 \| 1.49 \| A \| / \| / \| \| 6-14 \| B495 \| 89.0 \| 20 \| 1.78 \| 1.82 \| 1.77 \| A \| / \| / \| \| 6-15 \| B496 \| 292.6 \| 20 \| 5.85 \| 1.84 \| 2.01 \| A \| / \| / \| \| 6-16 \| B497 \| 90.2 \| 20 \| 1.80 \| 1.84 \| 1.68 \| A \| / \| / \| \| 6-17 \| B498 \| 42.0 \| 20 \| 0.84 \| 1.85 \| 1.52 \| A \| / \| / \| \| 6-18 \| B499 \| 53.0 \| 20 \| 1.06 \| 1.92 \| 1.51 \| A \| / \| / \| \| 6-19 \| B500 \| 106.5 \| 20 \| 2.13 \| 1.86 \| 1.71 \| A \| / \| / \| \| 6-20 \| B501 \| 34.3 \| 20 \| 0.69 \| 1.96 \| 1.46 \| A \| / \| / \| \| 6-21 \| B502 \| 17.8 \| 20 \| 0.36 \| 1.91 \| 1.09 \| A \| / \| / \| \| 6-22 \| B503 \| 57.6 \| 20 \| 1.15 \| 2.05 \| 1.67 \| A \| / \| / \| \| 6-23 \| B504 \| 18.2 \| 20 \| 0.36 \| 1.93 \| 1.13 \| A \| / \| / \| \| 6-24 \| B505 \| 48.0 \| 20 \| 0.96 \| 2.04 \| 1.60 \| A \| / \| / \| \| 6-25 \| B506 \| 28.4 \| 20 \| 0.57 \| 1.99 \| 1.42 \| A \| / \| / \| \| 6-26 \| B507 \| 112.8 \| 20 \| 2.26 \| 2.02 \| 1.91 \| A \| / \| / \| \| 6-27 \| B508 \| 11.2 \| 20 \| 0.22 \| 1.80 \| 0.74 \| A \| / \| / \| \| 6-28 \| B509 \| 44.9 \| 20 \| 0.90 \| 2.01 \| 1.57 \| A \| / \| / \| \| 6-29 \| B510 \| 29.9 \| 20 \| 0.60 \| 1.99 \| 1.36 \| A \| / \| / \| \| 6-30 \| B511 \| 131.2 \| 20 \| 2.62 \| 2.00 \| 1.87 \| A \| / \| / \| \| 6-31 \| B512 \| 34.3 \| 20 \| 0.69 \| 1.98 \| 1.41 \| A \| / \| / \| \| 6-32 \| B513 \| 26.9 \| 20 \| 0.54 \| 1.96 \| 1.23 \| A \| / \| / \| \| 6-33 \| B514 \| 74.8 \| 20 \| 1.50 \| 2.06 \| 1.88 \| C \| / \| / \| \| 6-34 \| B515 \| 33.2 \| 20 \| 0.66 \| 1.85 \| 1.30 \| A \| / \| / \| \| 6-35 \| B516 \| 71.3 \| 20 \| 1.43 \| 1.97 \| 1.68 \| A \| / \| / \| \| 6-36 \| B517 \| 48.0 \| 20 \| 0.96 \| 1.98 \| 1.61 \| A \| / \| / \| \| 6-37 \| B518 \| 65.7 \| 20 \| 1.31 \| 1.94 \| 1.54 \| A \| / \| / \| \| 6-38 \| B519 \| 49.8 \| 20 \| 1.00 \| 1.94 \| 1.62 \| A \| / \| / \| \| 6-39 \| B520 \| 37.8 \| 20 \| 0.76 \| 1.92 \| 1.41 \| A \| / \| / \| \| 6-40 \| B521 \| 34.0 \| 20 \| 0.68 \| 1.95 \| 1.42 \| A \| / \| / \| \| 6-41 \| B522 \| 55.1 \| 20 \| 1.10 \| 1.97 \| 1.60 \| A \| / \| / \| \| 6-42 \| B523 \| 95.5 \| 20 \| 1.91 \| 2.01 \| 1.94 \| A \| / \| / \| \| 6-43 \| B524 \| 125.6 \| 20 \| 2.51 \| 1.97 \| 1.98 \| A \| / \| / \| \| 6-44 \| B525 \| 29.7 \| 20 \| 0.59 \| 1.92 \| 1.36 \| A \| / \| / \| \| 6-45 \| B526 \| 140.5 \| 20 \| 2.81 \| 1.96 \| 1.98 \| A \| / \| / \| \| 6-46 \| B527 \| 41.0 \| 20 \| 0.82 \| 1.85 \| 1.35 \| A \| / \| / \| \| 6-47 \| B528 \| 61.1 \| 20 \| 1.22 \| 1.98 \| 1.70 \| A \| / \| / \| \| 6-48 \| B529 \| 117.3 \| 20 \| 2.35 \| 2.05 \| 1.95 \| A \| / \| / \| \| 6-49 \| B530 \| 41.5 \| 20 \| 0.83 \| 2.01 \| 1.48 \| A \| / \| / \| \| 6-50 \| B531 \| 82.8 \| 20 \| 1.66 \| 1.92 \| 2.04 \| A \| / \| / \| \| 6-51 \| B532 \| 20.4 \| 20 \| 0.41 \| 1.86 \| 1.08 \| A \| / \| / \| \| 6-52 \| B533 \| 39.5 \| 20 \| 0.79 \| 1.85 \| 1.35 \| A \| / \| / \| \| 6-53 \| B534 \| 26.8 \| 20 \| 0.54 \| 1.90 \| 1.24 \| A \| / \| / \| \| 6-54 \| B535 \| 28.2 \| 20 \| 0.56 \| 1.91 \| 1.35 \| A \| / \| / \| \| 6-55 \| B536 \| 52.2 \| 20 \| 1.04 \| 2.05 \| 1.61 \| A \| / \| / \| \| 6-56 \| B537 \| 96.6 \| 20 \| 1.93 \| 1.86 \| 1.96 \| A \| / \| / \| \| 6-57 \| B538 \| 41.8 \| 20 \| 0.84 \| 1.95 \| 1.57 \| A \| / \| / \| \| 6-58 \| B539 \| 26.2 \| 20 \| 0.52 \| 1.95 \| 1.37 \| A \| / \| / \| \| 6-59 \| B540 \| 27.2 \| 20 \| 0.54 \| 1.94 \| 1.35 \| A \| / \| / \| \| 6-60 \| B541 \| 83.1 \| 20 \| 1.66 \| 2.03 \| 1.82 \| A \| / \| / \| \| 6-61 \| B542 \| 24.8 \| 20 \| 0.50 \| 1.99 \| 1.26 \| A \| / \| / \| \| 6-62 \| B543 \| 35.8 \| 20 \| 0.72 \| 2.02 \| 1.42 \| A \| / \| / \| \| 6-63 \| B544 \| 50.9 \| 20 \| 1.02 \| 1.93 \| 1.64 \| A \| / \| / \| \| 6-64 \| B545 \| 72.3 \| 20 \| 1.45 \| 2.02 \| 1.83 \| A \| / \| / \| \| 6-65 \| B546 \| 20.6 \| 20 \| 0.41 \| 1.91 \| 1.07 \| A \| / \| / \| \| 6-66 \| B547 \| 29.7 \| 20 \| 0.59 \| 1.95 \| 1.31 \| A \| / \| / \| \| 6-67 \| B548 \| 40.9 \| 20 \| 0.82 \| 1.95 \| 1.50 \| A \| / \| / \| \| 6-68 \| B549 \| 41.9 \| 20 \| 0.84 \| 1.80 \| 1.54 \| A \| / \| / \| \| 6-69 \| B550 \| 30.6 \| 20 \| 0.61 \| 2.01 \| 1.53 \| A \| / \| / \| \| \| --- \| --- \| --- \| --- \| --- \| --- \| --- \| --- \| --- \| --- \| --- \| --- \| --- \| --- \| --- \| --- \| --- \| --- \| --- \| --- \| --- \| --- \| --- \| --- \| --- \| --- \| --- \| --- \| --- \| --- \| --- \| --- \| --- \| --- \| --- \| --- \| --- \| --- \| --- \| --- \| --- \| --- \| --- \| --- \| --- \| --- \| --- \| --- \| --- \| --- \| --- \| --- \| --- \| --- \| --- \| --- \| --- \| --- \| --- \| --- \| --- \| --- \| --- \| --- \| --- \| --- \| --- \| --- \| --- \| --- \| --- \| --- \| --- \| --- \| --- \| --- \| --- \| --- \| --- \| --- \| --- \| --- \| --- \| --- \| --- \| --- \| --- \| --- \| --- \| --- \| --- \| --- \| --- \| --- \| --- \| --- \| --- \| --- \| --- \| --- \| --- \| --- \| --- \| --- \| --- \| --- \| --- \| --- \| --- \| --- \| --- \| --- \| --- \| --- \| --- \| --- \| --- \| --- \| --- \| --- \| --- \| --- \| --- \| --- \| --- \| --- \| --- \| --- \| --- \| --- \| --- \| --- \| --- \| --- \| --- \| --- \| --- \| --- \| --- \| --- \| --- \| --- \| --- \| --- \| --- \| --- \| --- \| --- \| --- \| --- \| --- \| --- \| --- \| --- \| --- \| --- \| --- \| --- \| --- \| --- \| --- \| --- \| --- \| --- \| --- \| --- \| --- \| --- \| --- \| --- \| --- \| --- \| --- \| --- \| --- \| --- \| --- \| --- \| --- \| --- \| --- \| --- \| --- \| --- \| --- \| --- \| --- \| --- \| --- \| --- \| --- \| --- \| --- \| --- \| --- \| --- \| --- \| --- \| --- \| --- \| --- \| --- \| --- \| --- \| --- \| --- \| --- \| --- \| --- \| --- \| --- \| --- \| --- \| --- \| --- \| --- \| --- \| --- \| --- \| --- \| --- \| --- \| --- \| --- \| --- \| --- \| --- \| --- \| --- \| --- \| --- \| --- \| --- \| --- \| --- \| --- \| --- \| --- \| --- \| --- \| --- \| --- \| --- \| --- \| --- \| --- \| --- \| --- \| --- \| --- \| --- \| --- \| --- \| --- \| --- \| --- \| --- \| --- \| --- \| --- \| --- \| --- \| --- \| --- \| --- \| --- \| --- \| --- \| --- \| --- \| --- \| --- \| --- \| --- \| --- \| --- \| --- \| --- \| --- \| --- \| --- \| --- \| --- \| --- \| --- \| --- \| --- \| --- \| --- \| --- \| --- \| --- \| --- \| --- \| --- \| --- \| --- \| --- \| --- \| --- \| --- \| --- \| --- \| --- \| --- \| --- \| --- \| --- \| --- \| --- \| --- \| --- \| --- \| --- \| --- \| --- \| --- \| --- \| --- \| --- \| --- \| --- \| --- \| --- \| --- \| --- \| --- \| --- \| --- \| --- \| --- \| --- \| --- \| --- \| --- \| --- \| --- \| --- \| --- \| --- \| --- \| --- \| --- \| --- \| --- \| --- \| --- \| --- \| --- \| --- \| --- \| --- \| --- \| --- \| --- \| --- \| --- \| --- \| --- \| --- \| --- \| --- \| --- \| --- \| --- \| --- \| --- \| --- \| --- \| --- \| --- \| --- \| --- \| --- \| --- \| --- \| --- \| --- \| --- \| --- \| --- \| --- \| --- \| --- \| --- \| --- \| --- \| --- \| --- \| --- \| --- \| --- \| --- \| --- \| --- \| --- \| --- \| --- \| --- \| --- \| --- \| --- \| --- \| --- \| --- \| --- \| --- \| --- \| --- \| --- \| --- \| --- \| --- \| --- \| --- \| --- \| --- \| --- \| --- \| --- \| --- \| --- \| --- \| --- \| --- \| --- \| --- \| --- \| --- \| --- \| --- \| --- \| --- \| --- \| --- \| --- \| --- \| --- \| --- \| --- \| --- \| --- \| --- \| --- \| --- \| --- \| --- \| --- \| --- \| --- \| --- \| --- \| --- \| --- \| --- \| --- \| --- \| --- \| --- \| --- \| --- \| --- \| --- \| --- \| --- \| --- \| --- \| --- \| --- \| --- \| --- \| --- \| --- \| --- \| --- \| --- \| --- \| --- \| --- \| --- \| --- \| --- \| --- \| --- \| --- \| --- \| --- \| --- \| --- \| --- \| --- \| --- \| --- \| --- \| --- \| --- \| --- \| --- \| --- \| --- \| --- \| --- \| --- \| --- \| --- \| --- \| --- \| --- \| --- \| --- \| --- \| --- \| --- \| --- \| --- \| --- \| --- \| --- \| --- \| --- \| --- \| --- \| --- \| --- \| --- \| --- \| --- \| --- \| --- \| --- \| --- \| --- \| --- \| --- \| --- \| --- \| --- \| --- \| --- \| --- \| --- \| --- \| --- \| --- \| --- \| --- \| --- \| --- \| --- \| --- \| --- \| --- \| --- \| --- \| --- \| --- \| --- \| --- \| --- \| --- \| --- \| --- \| --- \| --- \| --- \| --- \| --- \| --- \| --- \| --- \| --- \| --- \| --- \| --- \| --- \| --- \| --- \| --- \| --- \| --- \| --- \| --- \| --- \| --- \| --- \| --- \| --- \| --- \| --- \| --- \| --- \| --- \| --- \| --- \| --- \| --- \| --- \| --- \| --- \| --- \| --- \| --- \| --- \| --- \| --- \| --- \| --- \| --- \| --- \| --- \| --- \| --- \| --- \| --- \| --- \| --- \| --- \| --- \| --- \| --- \| --- \| --- \| --- \| --- \| --- \| --- \| --- \| --- \| --- \| --- \| --- \| --- \| --- \| --- \| --- \| --- \| --- \| --- \| --- \| --- \| --- \| --- \| --- \| --- \| --- \| --- \| --- \| --- \| --- \| --- \| --- \| --- \| --- \| --- \| --- \| --- \| --- \| --- \| --- \| --- \| --- \| --- \| --- \| --- \| --- \| --- \| --- \| --- \| --- \| --- \| --- \| --- \| --- \| --- \| --- \| --- \| --- \| --- \| --- \| --- \| --- \| --- \| --- \| --- \| --- \| --- \| --- \| --- \| --- \| --- \| --- \| --- \| --- \| --- \| --- \| --- \| --- \| --- \| --- \| --- \| --- \| --- \| --- \| --- \| --- \| --- \| --- \| --- \| --- \| --- \| --- \| --- \| --- \| --- \| --- \| --- \| --- \| --- \| --- \| --- \| --- \| --- \| --- \| --- \| --- \| --- \| --- \| --- \| --- \| --- \| --- \| --- \| --- \| --- \| --- \| --- \| --- \| --- \| --- \| --- \| --- \| --- \| --- \| --- \| --- \| --- \| --- \| --- \| --- \| --- \| --- \| --- \| --- \| --- \| --- \| --- \| --- \| --- \| --- \| --- \| --- \| --- \| --- \| --- \| --- \| --- \| --- \| --- \| --- \| --- \| --- \| --- \| --- \| --- \| --- \| --- \| --- \| --- \| --- \| --- \| --- \| --- \| --- \| --- \| --- \| --- \| --- \| --- \| --- \| --- \| --- \| --- \| --- \| --- \| --- \| --- \| --- \| --- \| --- \| --- \| --- \| --- \| --- \| --- \| --- \| --- \| --- \| --- \| --- \| --- \| --- \| --- \| --- \| --- \| --- \| --- \| --- \| --- \| --- \| --- \| --- \| --- \| --- \| --- \| --- \| --- \| --- \| --- \| --- \| --- \| --- \| --- \| --- \| --- \| --- \| --- \| --- \| --- \| --- \| --- \| --- \| --- \| --- \| --- \| --- \| --- \| --- \| --- \| --- \| --- \| --- \| --- \| --- \| --- \| --- \| --- \| --- \| --- \| --- \| --- \| --- \| --- \| --- \| --- \| --- \| --- \| --- \| --- \| --- \| --- \| --- \| --- \| --- \| --- \| --- \| --- \| --- \| --- \| --- \| --- \| --- \| --- \| --- \| --- \| --- \| --- \| --- \| --- \| --- \| --- \| --- \| --- \| --- \| --- \| --- \| --- \| --- \| --- \| --- \| --- \| --- \| --- \| --- \| --- \| --- \| --- \| --- \| --- \| --- \| --- \| --- \| --- \| --- \| --- \| --- \| --- \| --- \| --- \| --- \| --- \| --- \| --- \| --- \| --- \| --- \| --- \| --- \| --- \| --- \| --- \| --- \| --- \| --- \| --- \| --- \| --- \| --- \| --- \| --- \| --- \| --- \| --- \| --- \| --- \| --- \| --- \| --- \| --- \| --- \| --- \| --- \| --- \| --- \| --- \| --- \| --- \| --- \| --- \| --- \| --- \| --- \| --- \| --- \| --- \| --- \| --- \| --- \| --- \| --- \| --- \| --- \| --- \| --- \| --- \| --- \| --- \| --- \| --- \| --- \| --- \| --- \| --- \| --- \| --- \| --- \| --- \| --- \| --- \| --- \| --- \| --- \| --- \| --- \| --- \| --- \| --- \| --- \| --- \| --- \| --- \| --- \| --- \| --- \| --- \| --- \| --- \| --- \| --- \| --- \| --- \| --- \| --- \| --- \| --- \| --- \| --- \| --- \| --- \| --- \| --- \| --- \| --- \| --- \| --- \| --- \| --- \| --- \| --- \| --- \| --- \| --- \| --- \| --- \| --- \| --- \| --- \| --- \| --- \| --- \| --- \| --- \| --- \| --- \| --- \| --- \| --- \| --- \| --- \| --- \| --- \| --- \| --- \| --- \| --- \| --- \| --- \| --- \| --- \| --- \| --- \| --- \| --- \| --- \| --- \| --- \| --- \| --- \| --- \| --- \| --- \| --- \| --- \| --- \| --- \| --- \| --- \| --- \| --- \| --- \| --- \| --- \| --- \| --- \| --- \| --- \| --- \| --- \| --- \| --- \| --- \| --- \| --- \| --- \| --- \| --- \| --- \| --- \| --- \| --- \| --- \| --- \| --- \| --- \| --- \| --- \| --- \| --- \| --- \| --- \| --- \| --- \| --- \| --- \| --- \| --- \| --- \| --- \| --- \| --- \| --- \| --- \| --- \| --- \| --- \| --- \| --- \| --- \| --- \| --- \| --- \| --- \| --- \| --- \| --- \| --- \| --- \| --- \| --- \| --- \| --- \| --- \| --- \| --- \| --- \| --- \| --- \| --- \| --- \| --- \| --- \| --- \| --- \| --- \| --- \| --- \| --- \| --- \| --- \| --- \| --- \| --- \| --- \| --- \| --- \| --- \| --- \| --- \| --- \| --- \| --- \| --- \| --- \| --- \| --- \| --- \| --- \| --- \| --- \| --- \| --- \| --- \| --- \| --- \| --- \| --- \| --- \| --- \| --- \| --- \| --- \| --- \| --- \| --- \| --- \| --- \| --- \| --- \| --- \| --- \| --- \| --- \| --- \| --- \| --- \| --- \| --- \| --- \| --- \| --- \| --- \| --- \| --- \| --- \| --- \| --- \| --- \| --- \| --- \| --- \| --- \| --- \| --- \| --- \| --- \| --- \| --- \| --- \| --- \| --- \| --- \| --- \| --- \| --- \| --- \| --- \| --- \| --- \| --- \| --- \| --- \| --- \| --- \| --- \| --- \| --- \| --- \| --- \| --- \| --- \| --- \| --- \| --- \| --- \| --- \| --- \| --- \| --- \| --- \| --- \| --- \| --- \| --- \| --- \| --- \| --- \| --- \| --- \| --- \| --- \| --- \| --- \| --- \| --- \| --- \| --- \| --- \| --- \| --- \| --- \| --- \| --- \| --- \| --- \| --- \| --- \| --- \| --- \| --- \| --- \| --- \| --- \| --- \| --- \| --- \| --- \| --- \| --- \| --- \| --- \| --- \| --- \| --- \| --- \| --- \| --- \| --- \| --- \| --- \| --- \| --- \| --- \| --- \| --- \| --- \| --- \| --- \| --- \| --- \| --- \| --- \| --- \| --- \| --- \| --- \| --- \| --- \| --- \| --- \| --- \| --- \| --- \| --- \| --- \| --- \| --- \| --- \| --- \| --- \| --- \| --- \| --- \| --- \| --- \| --- \| --- \| --- \| --- \| --- \| --- \| --- \| --- \| --- \| --- \| --- \| --- \| --- \| --- \| --- \| --- \| --- \| --- \| --- \| --- \| --- \| --- \| --- \| --- \| --- \| --- \| --- \| --- \| --- \| --- \| --- \| --- \| --- \| --- \| --- \| --- \| --- \| --- \| --- \| --- \| --- \| --- \| --- \| --- \| --- \| --- \| --- \| --- \| --- \| --- \| --- \| --- \| --- \| --- \| --- \| --- \| --- \| --- \| --- \| --- \| --- \| --- \| --- \| --- \| --- \| --- \| --- \| --- \| --- \| --- \| --- \| --- \| --- \| --- \| --- \| --- \| --- \| --- \| --- \| --- \| --- \| --- \| --- \| --- \| --- \| --- \| --- \| --- \| --- \| --- \| --- \| --- \| --- \| --- \| --- \| --- \| --- \| --- \| --- \| --- \| --- \| --- \| --- \| --- \| --- \| --- \| --- \| --- \| --- \| --- \| --- \| --- \| --- \| --- \| --- \| --- \| --- \| --- \| --- \| --- \| --- \| --- \| --- \| --- \| --- \| --- \| --- \| --- \| --- \| --- \| --- \| --- \| --- \| --- \| --- \| --- \| --- \| --- \| --- \| --- \| --- \| --- \| --- \| --- \| --- \| --- \| --- \| --- \| --- \| --- \| --- \| --- \| --- \| --- \| --- \| --- \| --- \| --- \| --- \| --- \| --- \| --- \| --- \| --- \| --- \| --- \| --- \| --- \| --- \| --- \| --- \| --- \| --- \| --- \| --- \| --- \| --- \| --- \| --- \| --- \| --- \| --- \| --- \| --- \| --- \| --- \| --- \| --- \| --- \| --- \| --- \| --- \| --- \| --- \| --- \| --- \| --- \| --- \| --- \| --- \| --- \| --- \| --- \| --- \| --- \| --- \| --- \| --- \| --- \| --- \| --- \| --- \| --- \| --- \| --- \| --- \| --- \| --- \| --- \| --- \| --- \| --- \| --- \| --- \| --- \| --- \| --- \| --- \| --- \| --- \| --- \| --- \| --- \| --- \| --- \| --- \| --- \| --- \| --- \| --- \| --- \| --- \| --- \| --- \| --- \| --- \| --- \| --- \| --- \| --- \| --- \| --- \| --- \| --- \| --- \| --- \| --- \| --- \| --- \| --- \| --- \| --- \| --- \| --- \| --- \| --- \| --- \| --- \| --- \| --- \| --- \| --- \| --- \| --- \| --- \| --- \| --- \| --- \| --- \| --- \| --- \| --- \| --- \| --- \| --- \| --- \| --- \| --- \| --- \| --- \| --- \| --- \| --- \| --- \| --- \| --- \| --- \| --- \| --- \| --- \| --- \| --- \| --- \| --- \| --- \| --- \| --- \| --- \| --- \| --- \| --- \| --- \| --- \| --- \| --- \| --- \| --- \| --- \| --- \| --- \| --- \| --- \| --- \| --- \| --- \| --- \| --- \| --- \| --- \| --- \| --- \| --- \| --- \| --- \| --- \| --- \| --- \| --- \| --- \| --- \| --- \| --- \| --- \| --- \| --- \| --- \| --- \| --- \| --- \| --- \| --- \| --- \| --- \| --- \| --- \| --- \| --- \| --- \| --- \| --- \| --- \| --- \| --- \| --- \| --- \| --- \| --- \| --- \| --- \| --- \| --- \| --- \| --- \| --- \| --- \| --- \| --- \| --- \| --- \| --- \| --- \| --- \| --- \| --- \| --- \| --- \| --- \| --- \| --- \| --- \| --- \| --- \| --- \| --- \| --- \| --- \| --- \| --- \| --- \| --- \| --- \| --- \| --- \| --- \| --- \| --- \| --- \| --- \| --- \| --- \| --- \| --- \| --- \| --- \| --- \| --- \| --- \| --- \| --- \| --- \| --- \| --- \| --- \| --- \| --- \| --- \| --- \| --- \| --- \| --- \| --- \| --- \| --- \| --- \| --- \| --- \| --- \| --- \| --- \| --- \| --- \| --- \| --- \| --- \| --- \| --- \| --- \| --- \| --- \| --- \| --- \| --- \| --- \| --- \| --- \| --- \| --- \| --- \| --- \| --- \| --- \| --- \| --- \| --- \| --- \| --- \| --- \| --- \| --- \| --- \| --- \| --- \| --- \| --- \| --- \| --- \| --- \| --- \| --- \| --- \| --- \| --- \| --- \| --- \| --- \| --- \| --- \| --- \| --- \| --- \| --- \| --- \| --- \| --- \| --- \| --- \| --- \| --- \| --- \| --- \| --- \| --- \| --- \| --- \| --- \| --- \| --- \| --- \| --- \| --- \| --- \| --- \| --- \| --- \| --- \| --- \| --- \| --- \| --- \| --- \| --- \| --- \| --- \| --- \| --- \| --- \| --- \| --- \| --- \| --- \| --- \| --- \| --- \| --- \| --- \| --- \| --- \| --- \| --- \| --- \| --- \| --- \| --- \| --- \| --- \| --- \| --- \| --- \| --- \| --- \| --- \| --- \| --- \| --- \| --- \| --- \| --- \| --- \| --- \| --- \| --- \| --- \| --- \| --- \| --- \| --- \| --- \| --- \| --- \| --- \| --- \| --- \| --- \| --- \| --- \| --- \| --- \| --- \| --- \| --- \| --- \| --- \| --- \| --- \| --- \| --- \| --- \| --- \| --- \| --- \| --- \| --- \| --- \| --- \| --- \| --- \| --- \| --- \| --- \| --- \| --- \| --- \| --- \| --- \| --- \| --- \| --- \| --- \| --- \| --- \| --- \| --- \| --- \| --- \| --- \| --- \| --- \| --- \| --- \| --- \| --- \| --- \| --- \| --- \| --- \| --- \| --- \| --- \| --- \| --- \| --- \| --- \| --- \| --- \| --- \| --- \| --- \| --- \| --- \| --- \| --- \| --- \| --- \| --- \| --- \| --- \| --- \| --- \| --- \| --- \| --- \| --- \| --- \| --- \| --- \| --- \| --- \| --- \| --- \| --- \| --- \| --- \| --- \| --- \| --- \| --- \| --- \| --- \| --- \| --- \| --- \| --- \| --- \| --- \| --- \| --- \| --- \| --- \| --- \| --- \| --- \| --- \| --- \| --- \| --- \| --- \| --- \| --- \| --- \| --- \| --- \| --- \| --- \| --- \| --- \| --- \| --- \| --- \| --- \| --- \| --- \| --- \| --- \| --- \| --- \| --- \| --- \| --- \| --- \| --- \| --- \| --- \| --- \| --- \| --- \| --- \| --- \| --- \| --- \| --- \| --- \| --- \| --- \| --- \| --- \| --- \| --- \| --- \| --- \| --- \| --- \| --- \| --- \| --- \| --- \| --- \| --- \| --- \| --- \| --- \| --- \| --- \| --- \| --- \| --- \| --- \| --- \| --- \| --- \| --- \| --- \| --- \| --- \| --- \| --- \| --- \| --- \| --- \| --- \| --- \| --- \| --- \| --- \| --- \| --- \| --- \| --- \| --- \| --- \| --- \| --- \| --- \| --- \| --- \| --- \| --- \| --- \| --- \| --- \| --- \| --- \| --- \| --- \| --- \| --- \| --- \| --- \| --- \| --- \| --- \| --- \| --- \| --- \| --- \| --- \| --- \| --- \| --- \| --- \| --- \| --- \| --- \| --- \| --- \| --- \| --- \| --- \| --- \| --- \| --- \| --- \| --- \| --- \| --- \| --- \| --- \| --- \| --- \| --- \| --- \| --- \| --- \| --- \| --- \| --- \| --- \| --- \| --- \| --- \| --- \| --- \| --- \| --- \| --- \| --- \| --- \| --- \| --- \| --- \| --- \| --- \| --- \| --- \| --- \| --- \| --- \| --- \| --- \| --- \| --- \| --- \| --- \| --- \| --- \| --- \| --- \| --- \| --- \| --- \| --- \| --- \| --- \| --- \| --- \| --- \| --- \| --- \| --- \| --- \| --- \| --- \| --- \| --- \| --- \| --- \| --- \| --- \| --- \| --- \| --- \| --- \| --- \| --- \| --- \| --- \| --- \| --- \| --- \| --- \| --- \| --- \| --- \| --- \| --- \| --- \| --- \| --- \| --- \| --- \| --- \| --- \| --- \| --- \| --- \| --- \| --- \| --- \| --- \| --- \| --- \| --- \| --- \| --- \| --- \| --- \| --- \| --- \| --- \| --- \| --- \| --- \| --- \| --- \| --- \| --- \| --- \| --- \| --- \| --- \| --- \| --- \| --- \| --- \| --- \| --- \| --- \| --- \| --- \| --- \| --- \| --- \| --- \| --- \| --- \| --- \| --- \| --- \| --- \| --- \| --- \| --- \| --- \| --- \| --- \| --- \| --- \| --- \| --- \| --- \| --- \| --- \| --- \| --- \| --- \| --- \| --- \| --- \| --- \| --- \| --- \| --- \| --- \| --- \| --- \| --- \| --- \| --- \| --- \| --- \| --- \| --- \| --- \| --- \| --- \| --- \| --- \| --- \| --- \| --- \| --- \| --- \| --- \| --- \| --- \| --- \| --- \| --- \| --- \| --- \| --- \| --- \| --- \| --- \| --- \| --- \| --- \| --- \| --- \| --- \| --- \| --- \| --- \| --- \| --- \| --- \| --- \| --- \| --- \| --- \| --- \| --- \| --- \| --- \| --- \| --- \| --- \| --- \| --- \| --- \| --- \| --- \| --- \| --- \| --- \| --- \| --- \| --- \| --- \| --- \| --- \| --- \| --- \| --- \| --- \| --- \| --- \| --- \| --- \| --- \| --- \| --- \| --- \| --- \| --- \| --- \| --- \| --- \| --- \| --- \| --- \| --- \| --- \| --- \| --- \| --- \| --- \| --- \| --- \| --- \| --- \| --- \| --- \| --- \| --- \| --- \| --- \| --- \| --- \| --- \| --- \| --- \| --- \| --- \| --- \| --- \| --- \| --- \| --- \| --- \| --- \| --- \| --- \| --- \| --- \| --- \| --- \| --- \| --- \| --- \| --- \| --- \| --- \| --- \| --- \| --- \| --- \| --- \| --- \| --- \| --- \| --- \| --- \| --- \| --- \| --- \| --- \| --- \| --- \| --- \| --- \| --- \| --- \| --- \| --- \| --- \| --- \| --- \| --- \| --- \| --- \| --- \| --- \| --- \| --- \| --- \| --- \| --- \| --- \| --- \| --- \| --- \| --- \| --- \| --- \| --- \| --- \| --- \| --- \| --- \| --- \| --- \| --- \| --- \| --- \| --- \| --- \| --- \| --- \| --- \| --- \| --- \| --- \| --- \| --- \| --- \| --- \| --- \| --- \| --- \| --- \| --- \| --- \| --- \| --- \| --- \| --- \| --- \| --- \| --- \| --- \| --- \| --- \| --- \| --- \| --- \| --- \| --- \| --- \| --- \| --- \| --- \| --- \| --- \| --- \| --- \| --- \| --- \| --- \| --- \| --- \| --- \| --- \| --- \| --- \| --- \| --- \| --- \| --- \| --- \| --- \| --- \| --- \| --- \| --- \| --- \| --- \| --- \| --- \| --- \| --- \| --- \| --- \| --- \| --- \| --- \| --- \| --- \| --- \| --- \| --- \| --- \| --- \| --- \| --- \| --- \| --- \| --- \| --- \| --- \| --- \| --- \| --- \| --- \| --- \| --- \| --- \| --- \| --- \| --- \| --- \| --- \| --- \| --- \| --- \| --- \| --- \| --- \| --- \| --- \| --- \| --- \| --- \| --- \| --- \| --- \| --- \| --- \| --- \| --- \| --- \| --- \| --- \| --- \| --- \| --- \| --- \| --- \| --- \| --- \| --- \| --- \| --- \| --- \| --- \| --- \| --- \| --- \| --- \| --- \| --- \| --- \| --- \| --- \| --- \| --- \| --- \| --- \| --- \| --- \| --- \| --- \| --- \| --- \| --- \| --- \| --- \| --- \| --- \| --- \| --- \| --- \| --- \| --- \| --- \| --- \| --- \| --- \| --- \| --- \| --- \| --- \| --- \| --- \| --- \| --- \| --- \| --- \| --- \| --- \| --- \| --- \| --- \| --- \| --- \| --- \| --- \| --- \| --- \| --- \| --- \| --- \| --- \| --- \| --- \| --- \| --- \| --- \| --- \| --- \| --- \| --- \| --- \| --- \| --- \| --- \| --- \| --- \| --- \| --- \| --- \| --- \| --- \| --- \| --- \| --- \| --- \| --- \| --- \| --- \| --- \| --- \| --- \| --- \| --- \| --- \| --- \| --- \| --- \| --- \| --- \| --- \| --- \| --- \| --- \| --- \| --- \| --- \| --- \| --- \| --- \| --- \| --- \| --- \| --- \| --- \| --- \| --- \| --- \| --- \| --- \| --- \| --- \| --- \| --- \| --- \| --- \| --- \| --- \| --- \| --- \| --- \| --- \| --- \| --- \| --- \| --- \| --- \| --- \| --- \| --- \| --- \| --- \| --- \| --- \| --- \| --- \| --- \| --- \| --- \| --- \| --- \| --- \| --- \| --- \| --- \| --- \| --- \| --- \| --- \| --- \| --- \| --- \| --- \| --- \| --- \| --- \| --- \| --- \| --- \| --- \| --- \| --- \| --- \| --- \| --- \| --- \| --- \| --- \| --- \| --- \| --- \| --- \| --- \| --- \| --- \| --- \| --- \| --- \| --- \| --- \| --- \| --- \| --- \| --- \| --- \| --- \| --- \| --- \| --- \| --- \| --- \| --- \| --- \| --- \| --- \| --- \| --- \| --- \| --- \| --- \| --- \| --- \| --- \| --- \| --- \| --- \| --- \| --- \| --- \| --- \| --- \| --- \| --- \| --- \| --- \| --- \| --- \| --- \| --- \| --- \| --- \| --- \| --- \| --- \| --- \| --- \| --- \| --- \| --- \| --- \| --- \| --- \| --- \| --- \| --- \| --- \| --- \| --- \| --- \| --- \| --- \| --- \| --- \| --- \| --- \| --- \| --- \| --- \| --- \| --- \| --- \| --- \| --- \| --- \| --- \| --- \| --- \| --- \| --- \| --- \| --- \| --- \| --- \| --- \| --- \| --- \| --- \| --- \| --- \| --- \| --- \| --- \| --- \| --- \| --- \| --- \| --- \| --- \| --- \| --- \| --- \| --- \| --- \| --- \| --- \| --- \| --- \| --- \| --- \| --- \| --- \| --- \| --- \| --- \| --- \| --- \| --- \| --- \| --- \| --- \| --- \| --- \| --- \| --- \| --- \| --- \| --- \| --- \| --- \| --- \| --- \| --- \| --- \| --- \| --- \| --- \| --- \| --- \| --- \| --- \| --- \| --- \| --- \| --- \| --- \| --- \| --- \| --- \| --- \| --- \| --- \| --- \| --- \| --- \| --- \| --- \| --- \| --- \| --- \| --- \| --- \| --- \| --- \| --- \| --- \| --- \| --- \| --- \| --- \| --- \| --- \| --- \| --- \| --- \| --- \| --- \| --- \| --- \| --- \| --- \| --- \| --- \| --- \| --- \| --- \| --- \| --- \| --- \| --- \| --- \| --- \| --- \| --- \| --- \| --- \| --- \| --- \| --- \| --- \| --- \| --- \| --- \| --- \| --- \| --- \| --- \| --- \| --- \| --- \| --- \| --- \| --- \| --- \| --- \| --- \| --- \| --- \| --- \| --- \| --- \| --- \| --- \| --- \| --- \| --- \| --- \| --- \| --- \| --- \| --- \| --- \| --- \| --- \| --- \| --- \| --- \| --- \| --- \| --- \| --- \| --- \| --- \| --- \| --- \| --- \| --- \| --- \| --- \| --- \| --- \| --- \| --- \| --- \| --- \| --- \| --- \| --- \| --- \| --- \| --- \| --- \| --- \| --- \| --- \| --- \| --- \| --- \| --- \| --- \| --- \| --- \| --- \| --- \| --- \| --- \| --- \| --- \| --- \| --- \| --- \| --- \| --- \| --- \| --- \| --- \| --- \| --- \| --- \| --- \| --- \| --- \| --- \| --- \| --- \| --- \| --- \| --- \| --- \| --- \| --- \| --- \| --- \| --- \| --- \| --- \| --- \| --- \| --- \| --- \| --- \| --- \| --- \| --- \| --- \| --- \| --- \| --- \| --- \| --- \| --- \| --- \| --- \| --- \| --- \| --- \| --- \| --- \| --- \| --- \| --- \| --- \| --- \| --- \| --- \| --- \| --- \| --- \| --- \| --- \| --- \| --- \| --- \| --- \| --- \| --- \| --- \| --- \| --- \| --- \| --- \| --- \| --- \| --- \| --- \| --- \| --- \| --- \| --- \| --- \| --- \| --- \| --- \| --- \| --- \| --- \| --- \| --- \| --- \| --- \| --- \| --- \| --- \| --- \| --- \| --- \| --- \| --- \| --- \| --- \| --- \| --- \| --- \| --- \| --- \| --- \| --- \| --- \| --- \| --- \| --- \| --- \| --- \| --- \| --- \| --- \| --- \| --- \| --- \| --- \| --- \| --- \| --- \| --- \| --- \| --- \| --- \| --- \| --- \| --- \| --- \| --- \| --- \| --- \| --- \| --- \| --- \| --- \| --- \| --- \| --- \| --- \| --- \| --- \| --- \| --- \| --- \| --- \| --- \| --- \| --- \| --- \| --- \| --- \| --- \| --- \| --- \| --- \| --- \| --- \| --- \| --- \| --- \| --- \| --- \| --- \| --- \| --- \| --- \| --- \| --- \| --- \| --- \| --- \| --- \| --- \| --- \| --- \| --- \| --- \| --- \| --- \| --- \| --- \| --- \| --- \| --- \| --- \| --- \| --- \| --- \| --- \| --- \| --- \| --- \| --- \| --- \| --- \| --- \| --- \| --- \| --- \| --- \| --- \| --- \| --- \| --- \| --- \| --- \| --- \| --- \| --- \| --- \| --- \| --- \| --- \| --- \| --- \| --- \| --- \| --- \| --- \| --- \| --- \| --- \| --- \| --- \| --- \| --- \| --- \| --- \| --- \| --- \| --- \| --- \| --- \| --- \| --- \| --- \| --- \| --- \| --- \| --- \| --- \| --- \| --- \| --- \| --- \| --- \| --- \| --- \| --- \| --- \| --- \| --- \| --- \| --- \| --- \| --- \| --- \| --- \| --- \| --- \| --- \| --- \| --- \| --- \| --- \| --- \| --- \| --- \| --- \| --- \| --- \| --- \| --- \| --- \| --- \| --- \| --- \| --- \| --- \| --- \| --- \| --- \| --- \| --- \| --- \| --- \| --- \| --- \| --- \| --- \| --- \| --- \| --- \| --- \| --- \| --- \| --- \| --- \| --- \| --- \| --- \| --- \| --- \| --- \| --- \| --- \| --- \| --- \| --- \| --- \| --- \| --- \| --- \| --- \| --- \| --- \| --- \| --- \| --- \| --- \| --- \| --- \| --- \| --- \| --- \| --- \| --- \| --- \| --- \| --- \| --- \| --- \| --- \| --- \| --- \| --- \| --- \| --- \| --- \| --- \| --- \| --- \| --- \| --- \| --- \| --- \| --- \| --- \| --- \| --- \| --- \| --- \| --- \| --- \| --- \| --- \| --- \| --- \| --- \| --- \| --- \| --- \| --- \| --- \| --- \| --- \| --- \| --- \| --- \| --- \| --- \| --- \| --- \| --- \| --- \| --- \| --- \| --- \| --- \| --- \| --- \| --- \| --- \| --- \| --- \| --- \| --- \| --- \| --- \| --- \| --- \| --- \| --- \| --- \| --- \| --- \| --- \| --- \| --- \| --- \| --- \| --- \| --- \| --- \| --- \| --- \| --- \| --- \| --- \| --- \| --- \| --- \| --- \| --- \| --- \| --- \| --- \| --- \| --- \| --- \| --- \| --- \| --- \| --- \| --- \| --- \| --- \| --- \| --- \| --- \| --- \| --- \| --- \| --- \| --- \| --- \| --- \| --- \| --- \| --- \| --- \| --- \| --- \| --- \| --- \| --- \| --- \| --- \| --- \| --- \| --- \| --- \| --- \| --- \| --- \| --- \| --- \| --- \| --- \| --- \| --- \| --- \| --- \| --- \| --- \| --- \| --- \| --- \| --- \| --- \| --- \| --- \| --- \| --- \| --- \| --- \| --- \| --- \| --- \| --- \| --- \| --- \| --- \| --- \| --- \| --- \| --- \| --- \| --- \| --- \| --- \| --- \| --- \| --- \| --- \| --- \| --- \| --- \| --- \| --- \| --- \| --- \| --- \| --- \| --- \| --- \| --- \| --- \| --- \| --- \| --- \| --- \| --- \| --- \| --- \| --- \| --- \| --- \| --- \| --- \| --- \| --- \| --- \| --- \| --- \| --- \| --- \| --- \| --- \| --- \| --- \| --- \| --- \| --- \| --- \| --- \| --- \| --- \| --- \| --- \| --- \| --- \| --- \| --- \| --- \| --- \| --- \| --- \| --- \| --- \| --- \| --- \| --- \| --- \| --- \| --- \| --- \| --- \| --- \| --- \| --- \| --- \| --- \| --- \| --- \| --- \| --- \| --- \| --- \| --- \| --- \| --- \| --- \| --- \| --- \| --- \| --- \| --- \| --- \| --- \| --- \| --- \| --- \| --- \| --- \| --- \| --- \| --- \| --- \| --- \| --- \| --- \| --- \| --- \| --- \| --- \| --- \| --- \| --- \| --- \| --- \| --- \| --- \| --- \| --- \| --- \| --- \| --- \| --- \| --- \| --- \| --- \| --- \| --- \| --- \| --- \| --- \| --- \| --- \| --- \| --- \| --- \| --- \| --- \| --- \| --- \| --- \| --- \| --- \| --- \| --- \| --- \| --- \| --- \| --- \| --- \| --- \| --- \| --- \| --- \| --- \| --- \| --- \| --- \| --- \| --- \| --- \| --- \| --- \| --- \| --- \| --- \| --- \| --- \| --- \| --- \| --- \| --- \| --- \| --- \| --- \| --- \| --- \| --- \| --- \| --- \| --- \| --- \| --- \| --- \| --- \| --- \| --- \| --- \| --- \| --- \| --- \| --- \| --- \| --- \| --- \| --- \| --- \| --- \| --- \| --- \| --- \| --- \| --- \| --- \| --- \| --- \| --- \| --- \| --- \| --- \| --- \| --- \| --- \| --- \| --- \| --- \| --- \| --- \| --- \| --- \| --- \| --- \| --- \| --- \| --- \| --- \| --- \| --- \| --- \| --- \| --- \| --- \| --- \| --- \| --- \| --- \| --- \| --- \| --- \| --- \| --- \| --- \| --- \| --- \| --- \| --- \| --- \| --- \| --- \| --- \| --- \| --- \| --- \| --- \| --- \| --- \| --- \| --- \| --- \| --- \| --- \| --- \| --- \| --- \| --- \| --- \| --- \| --- \| --- \| --- \| --- \| --- \| --- \| --- \| --- \| --- \| --- \| --- \| --- \| --- \| --- \| --- \| --- \| --- \| --- \| --- \| --- \| --- \| --- \| --- \| --- \| --- \| --- \| --- \| --- \| --- \| --- \| --- \| --- \| --- \| --- \| --- \| --- \| --- \| --- \| --- \| --- \| --- \| --- \| --- \| --- \| --- \| --- \| --- \| --- \| --- \| --- \| --- \| --- \| --- \| --- \| --- \| --- \| --- \| --- \| --- \| --- \| --- \| --- \| --- \| --- \| --- \| --- \| --- \| --- \| --- \| --- \| --- \| --- \| --- \| --- \| --- \| --- \| --- \| --- \| --- \| --- \| --- \| --- \| --- \| --- \| --- \| --- \| --- \| --- \| --- \| --- \| --- \| --- \| --- \| --- \| --- \| --- \| --- \| --- \| --- \| --- \| --- \| --- \| --- \| --- \| --- \| --- \| --- \| --- \| --- \| --- \| --- \| --- \| --- \| --- \| --- \| --- \| --- \| --- \| --- \| --- \| --- \| --- \| --- \| --- \| --- \| --- \| --- \| --- \| --- \| --- \| --- \| --- \| --- \| --- \| --- \| --- \| --- \| --- \| --- \| --- \| --- \| --- \| --- \| --- \| --- \| --- \| --- \| --- \| --- \| --- \| --- \| --- \| --- \| --- \| --- \| --- \| --- \| --- \| --- \| --- \| --- \| --- \| --- \| --- \| --- \| --- \| --- \| --- \| --- \| --- \| --- \| --- \| --- \| --- \| --- \| --- \| --- \| --- \| --- \| --- \| --- \| --- \| --- \| --- \| --- \| --- \| --- \| --- \| --- \| --- \| --- \| --- \| --- \| --- \| --- \| --- \| --- \| --- \| --- \| --- \| --- \| --- \| --- \| --- \| --- \| --- \| --- \| --- \| --- \| --- \| --- \| --- \| --- \| --- \| --- \| --- \| --- \| --- \| --- \| --- \| --- \| --- \| --- \| --- \| --- \| --- \| --- \| --- \| --- \| --- \| --- \| --- \| --- \| --- \| --- \| --- \| --- \| --- \| --- \| --- \| --- \| --- \| --- \| --- \| --- \| --- \| --- \| --- \| --- \| --- \| --- \| --- \| --- \| --- \| --- \| --- \| --- \| --- \| --- \| --- \| --- \| --- \| --- \| --- \| --- \| --- \| --- \| --- \| --- \| --- \| --- \| --- \| --- \| --- \| --- \| --- \| --- \| --- \| --- \| --- \| --- \| --- \| --- \| --- \| --- \| --- \| --- \| --- \| --- \| --- \| --- \| --- \| --- \| --- \| --- \| --- \| --- \| --- \| --- \| --- \| --- \| --- \| --- \| --- \| --- \| --- \| --- \| --- \| --- \| --- \| --- \| --- \| --- \| --- \| --- \| --- \| --- \| --- \| --- \| --- \| --- \| --- \| --- \| --- \| --- \| --- \| --- \| --- \| --- \| --- \| --- \| --- \| --- \| --- \| --- \| --- \| --- \| --- \| --- \| --- \| --- \| --- \| --- \| --- \| --- \| --- \| --- \| --- \| --- \| --- \| --- \| --- \| --- \| --- \| --- \| --- \| --- \| --- \| --- \| --- \| --- \| --- \| --- \| --- \| --- \| --- \| --- \| --- \| --- \| --- \| --- \| --- \| --- \| --- \| --- \| --- \| --- \| --- \| --- \| --- \| --- \| --- \| --- \| --- \| --- \| --- \| --- \| --- \| --- \| --- \| --- \| --- \| --- \| --- \| --- \| --- \| --- \| --- \| --- \| --- \| --- \| --- \| --- \| --- \| --- \| --- \| --- \| --- \| --- \| --- \| --- \| --- \| --- \| --- \| --- \| --- \| --- \| --- \| --- \| --- \| --- \| --- \| --- \| --- \| --- \| --- \| --- \| --- \| --- \| --- \| --- \| --- \| --- \| --- \| --- \| --- \| --- \| --- \| --- \| --- \| --- \| --- \| --- \| --- \| --- \| --- \| --- \| --- \| --- \| --- \| --- \| --- \| --- \| --- \| --- \| --- \| --- \| --- \| --- \| --- \| --- \| --- \| --- \| --- \| --- \| --- \| --- \| --- \| --- \| --- \| --- \| --- \| --- \| --- \| --- \| --- \| --- \| --- \| --- \| --- \| --- \| --- \| --- \| --- \| --- \| --- \| --- \| --- \| --- \| --- \| --- \| --- \| --- \| --- \| --- \| --- \| --- \| --- \| --- \| --- \| --- \| --- \| --- \| --- \| --- \| --- \| --- \| --- \| --- \| --- \| --- \| --- \| --- \| --- \| --- \| --- \| --- \| --- \| --- \| --- \| --- \| --- \| --- \| --- \| --- \| --- \| --- \| --- \| --- \| --- \| --- \| --- \| --- \| --- \| --- \| --- \| --- \| --- \| --- \| --- \| --- \| --- \| --- \| --- \| --- \| --- \| --- \| --- \| --- \| --- \| --- \| --- \| --- \| --- \| --- \| --- \| --- \| --- \| --- \| --- \| --- \| --- \| --- \| --- \| --- \| --- \| --- \| --- \| --- \| --- \| --- \| --- \| --- \| --- \| --- \| --- \| --- \| --- \| --- \| --- \| --- \| --- \| --- \| --- \| --- \| --- \| --- \| --- \| --- \| --- \| --- \| --- \| --- \| --- \| --- \| --- \| --- \| --- \| --- \| --- \| --- \| --- \| --- \| --- \| --- \| --- \| --- \| --- \| --- \| --- \| --- \| --- \| --- \| --- \| --- \| --- \| --- \| --- \| --- \| --- \| --- \| --- \| --- \| --- \| --- \| --- \| --- \| --- \| --- \| --- \| --- \| --- \| --- \| --- \| --- \| --- \| --- \| --- \| --- \| --- \| --- \| --- \| --- \| --- \| --- \| --- \| --- \| --- \| --- \| --- \| --- \| --- \| --- \| --- \| --- \| --- \| --- \| --- \| --- \| --- \| --- \| --- \| --- \| --- \| --- \| --- \| --- \| --- \| --- \| --- \| --- \| --- \| --- \| --- \| --- \| --- \| --- \| --- \| --- \| --- \| --- \| --- \| --- \| --- \| --- \| --- \| --- \| --- \| --- \| --- \| --- \| --- \| --- \| --- \| --- \| --- \| --- \| --- \| --- \| --- \| --- \| --- \| --- \| --- \| --- \| --- \| --- \| --- \| --- \| --- \| --- \| --- \| --- \| --- \| --- \| --- \| --- \| --- \| --- \| --- \| --- \| --- \| --- \| --- \| --- \| --- \| --- \| --- \| --- \| --- \| --- \| --- \| --- \| --- \| --- \| --- \| --- \| --- \| --- \| --- \| --- \| --- \| --- \| --- \| --- \| --- \| --- \| --- \| --- \| --- \| --- \| --- \| --- \| --- \| --- \| --- \| --- \| --- \| --- \| --- \| --- \| --- \| --- \| --- \| --- \| --- \| --- \| --- \| --- \| --- \| --- \| --- \| --- \| --- \| --- \| --- \| --- \| --- \| --- \| --- \| --- \| --- \| --- \| --- \| --- \| --- \| --- \| --- \| --- \| --- \| --- \| --- \| --- \| --- \| --- \| --- \| --- \| --- \| --- \| --- \| --- \| --- \| --- \| --- \| --- \| --- \| --- \| --- \| --- \| --- \| --- \| --- \| --- \| --- \| --- \| --- \| --- \| --- \| --- \| --- \| --- \| --- \| --- \| --- \| --- \| --- \| --- \| --- \| --- \| --- \| --- \| --- \| --- \| --- \| --- \| --- \| --- \| --- \| --- \| --- \| --- \| --- \| --- \| --- \| --- \| --- \| --- \| --- \| --- \| --- \| --- \| --- \| --- \| --- \| --- \| --- \| --- \| --- \| --- \| --- \| --- \| --- \| --- \| --- \| --- \| --- \| --- \| --- \| --- \| --- \| --- \| --- \| --- \| --- \| --- \| --- \| --- \| --- \| --- \| --- \| --- \| --- \| --- \| --- \| --- \| --- \| --- \| --- \| --- \| --- \| --- \| --- \| --- \| --- \| --- \| --- \| --- \| --- \| --- \| --- \| --- \| --- \| --- \| --- \| --- \| --- \| --- \| --- \| --- \| --- \| --- \| --- \| --- \| --- \| --- \| --- \| --- \| --- \| --- \| --- \| --- \| --- \| --- \| --- \| --- \| --- \| --- \| --- \| --- \| --- \| --- \| --- \| --- \| --- \| --- \| --- \| --- \| --- \| --- \| --- \| --- \| --- \| --- \| --- \| --- \| --- \| --- \| --- \| --- \| --- \| --- \| --- \| --- \| --- \| --- \| --- \| --- \| --- \| --- \| --- \| --- \| --- \| --- \| --- \| --- \| --- \| --- \| --- \| --- \| --- \| --- \| --- \| --- \| --- \| --- \| --- \| --- \| --- \| --- \| --- \| --- \| --- \| --- \| --- \| --- \| --- \| --- \| --- \| --- \| --- \| --- \| --- \| --- \| --- \| --- \| --- \| --- \| --- \| --- \| --- \| --- \| --- \| --- \| --- \| --- \| --- \| --- \| --- \| --- \| --- \| --- \| --- \| --- \| --- \| --- \| --- \| --- \| --- \| --- \| --- \| --- \| --- \| --- \| --- \| --- \| --- \| --- \| --- \| --- \| --- \| --- \| --- \| --- \| --- \| --- \| --- \| --- \| --- \| --- \| --- \| --- \| --- \| --- \| --- \| --- \| --- \| --- \| --- \| --- \| --- \| --- \| --- \| --- \| --- \| --- \| --- \| --- \| --- \| --- \| --- \| --- \| --- \| --- \| --- \| --- \| --- \| --- \| --- \| --- \| --- \| --- \| --- \| --- \| --- \| --- \| --- \| --- \| --- \| --- \| --- \| --- \| --- \| --- \| --- \| --- \| --- \| --- \| --- \| --- \| --- \| --- \| --- \| --- \| --- \| --- \| --- \| --- \| --- \| --- \| --- \| --- \| --- \| --- \| --- \| --- \| --- \| --- \| --- \| --- \| --- \| --- \| --- \| --- \| --- \| --- \| --- \| --- \| --- \| --- \| --- \| --- \| --- \| --- \| --- \| --- \| --- \| --- \| --- \| --- \| --- \| --- \| --- \| --- \| --- \| --- \| --- \| --- \| --- \| --- \| --- \| --- \| --- \| --- \| --- \| --- \| --- \| --- \| --- \| --- \| --- \| --- \| --- \| --- \| --- \| --- \| --- \| --- \| --- \| --- \| --- \| --- \| --- \| --- \| --- \| --- \| --- \| --- \| --- \| --- \| --- \| --- \| --- \| --- \| --- \| --- \| --- \| --- \| --- \| --- \| --- \| --- \| --- \| --- \| --- \| --- \| --- \| --- \| --- \| --- \| --- \| --- \| --- \| --- \| --- \| --- \| --- \| --- \| --- \| --- \| --- \| --- \| --- \| --- \| --- \| --- \| --- \| --- \| --- \| --- \| --- \| --- \| --- \| --- \| --- \| --- \| --- \| --- \| --- \| --- \| --- \| --- \| --- \| --- \| --- \| --- \| --- \| --- \| --- \| --- \| --- \| --- \| --- \| --- \| --- \| --- \| --- \| --- \| --- \| --- \| --- \| --- \| --- \| --- \| --- \| --- \| --- \| --- \| --- \| --- \| --- \| --- \| --- \| --- \| --- \| --- \| --- \| --- \| --- \| --- \| --- \| --- \| --- \| --- \| --- \| --- \| --- \| --- \| --- \| --- \| --- \| --- \| --- \| --- \| --- \| --- \| --- \| --- \| --- \| --- \| --- \| --- \| --- \| --- \| --- \| --- \| --- \| --- \| --- \| --- \| --- \| --- \| --- \| --- \| --- \| --- \| --- \| --- \| --- \| --- \| --- \| --- \| --- \| --- \| --- \| --- \| --- \| --- \| --- \| --- \| --- \| --- \| --- \| --- \| --- \| --- \| --- \| --- \| --- \| --- \| --- \| --- \| --- \| --- \| --- \| --- \| --- \| --- \| --- \| --- \| --- \| --- \| --- \| --- \| --- \| --- \| --- \| --- \| --- \| --- \| --- \| --- \| --- \| --- \| --- \| --- \| --- \| --- \| --- \| --- \| --- \| --- \| --- \| --- \| --- \| --- \| --- \| --- \| --- \| --- \| --- \| --- \| --- \| --- \| --- \| --- \| --- \| --- \| --- \| --- \| --- \| --- \| --- \| --- \| --- \| --- \| --- \| --- \| --- \| --- \| --- \| --- \| --- \| --- \| --- \| --- \| --- \| --- \| --- \| --- \| --- \| --- \| --- \| --- \| --- \| --- \| --- \| --- \| --- \| --- \| --- \| --- \| --- \| --- \| --- \| --- \| --- \| --- \| --- \| --- \| --- \| --- \| --- \| --- \| --- \| --- \| --- \| --- \| --- \| --- \| --- \| --- \| --- \| --- \| --- \| --- \| --- \| --- \| --- \| --- \| --- \| --- \| --- \| --- \| --- \| --- \| --- \| --- \| --- \| --- \| --- \| --- \| --- \| --- \| --- \| --- \| --- \| --- \| --- \| --- \| --- \| --- \| --- \| --- \| --- \| --- \| --- \| --- \| --- \| --- \| --- \| --- \| --- \| --- \| --- \| --- \| --- \| --- \| --- \| --- \| --- \| --- \| --- \| --- \| --- \| --- \| --- \| --- \| --- \| --- \| --- \| --- \| --- \| --- \| --- \| --- \| --- \| --- \| --- \| --- \| --- \| --- \| --- \| --- \| --- \| --- \| --- \| --- \| --- \| --- \| --- \| --- \| --- \| --- \| --- \| --- \| --- \| --- \| --- \| --- \| --- \| --- \| --- \| --- \| --- \| --- \| --- \| --- \| --- \| --- \| --- \| --- \| --- \| --- \| --- \| --- \| --- \| --- \| --- \| --- \| --- \| --- \| --- \| --- \| --- \| --- \| --- \| --- \| --- \| --- \| --- \| --- \| --- \| --- \| --- \| --- \| --- \| --- \| --- \| --- \| --- \| --- \| --- \| --- \| --- \| --- \| --- \| --- \| --- \| --- \| --- \| --- \| --- \| --- \| --- \| --- \| --- \| --- \| --- \| --- \| --- \| --- \| --- \| --- \| --- \| --- \| --- \| --- \| --- \| --- \| --- \| --- \| --- \| --- \| --- \| --- \| --- \| --- \| --- \| --- \| --- \| --- \| --- \| --- \| --- \| --- \| --- \| --- \| --- \| --- \| --- \| --- \| --- \| --- \| --- \| --- \| --- \| --- \| --- \| --- \| --- \| --- \| --- \| --- \| --- \| --- \| --- \| --- \| --- \| --- \| --- \| --- \| --- \| --- \| --- \| --- \| --- \| --- \| --- \| --- \| --- \| --- \| --- \| --- \| --- \| --- \| --- \| --- \| --- \| --- \| --- \| --- \| --- \| --- \| --- \| --- \| --- \| --- \| --- \| --- \| --- \| --- \| --- \| --- \| --- \| --- \| | | | |
|  | | | |
| \| **Electrophoretogram** \| \| --- \|   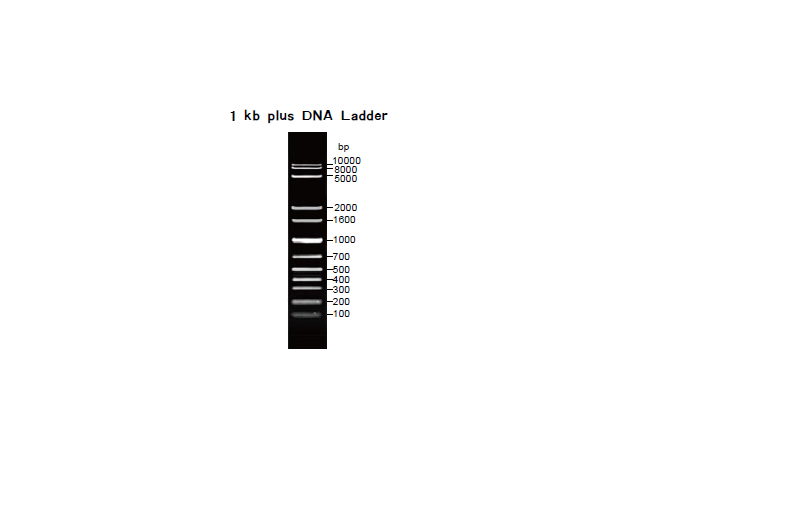  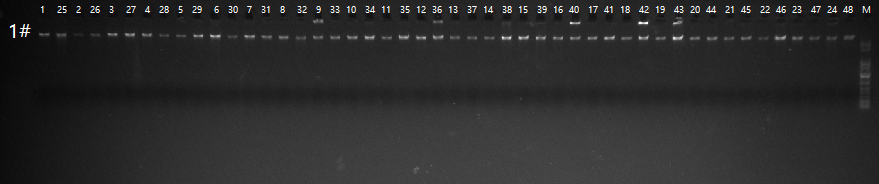  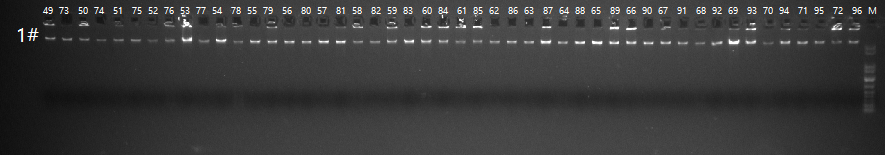  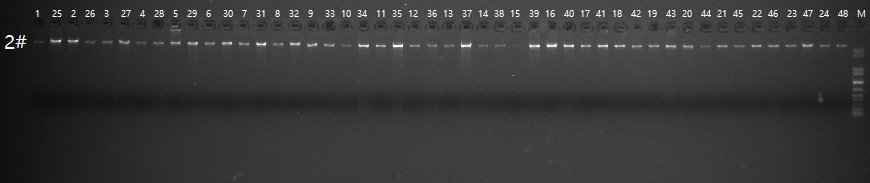  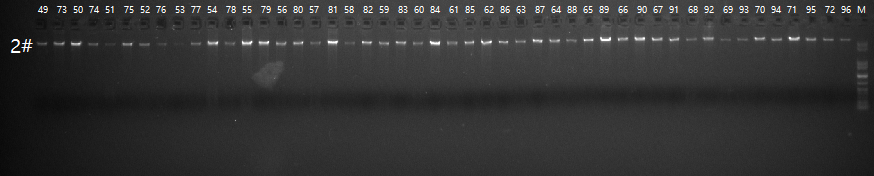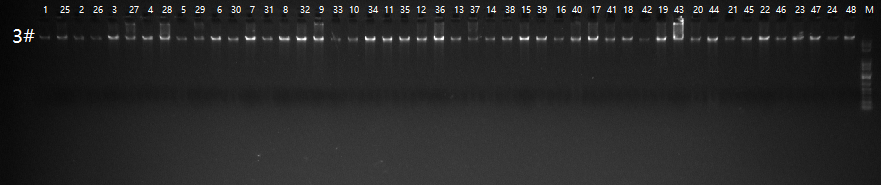  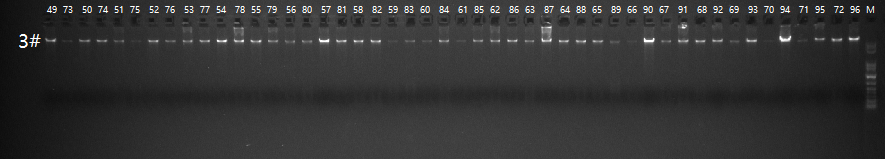  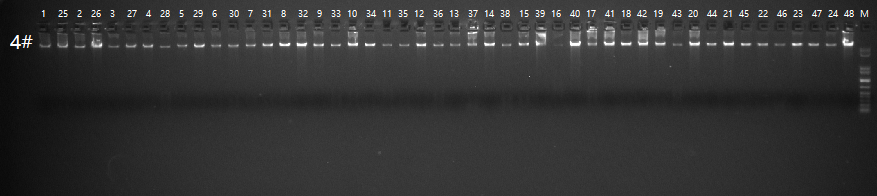  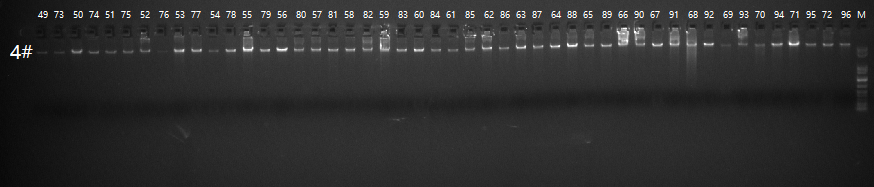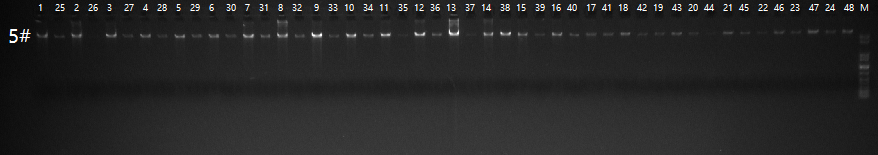  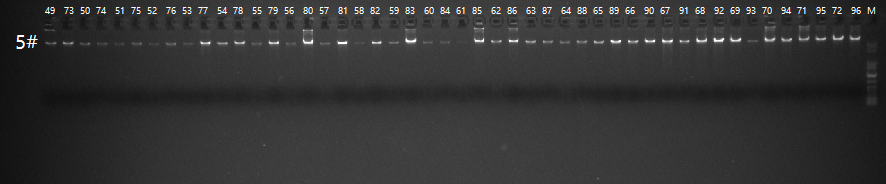  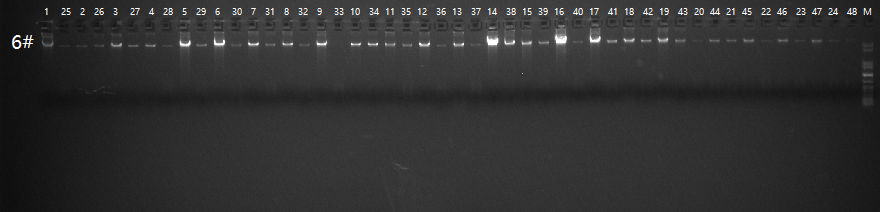  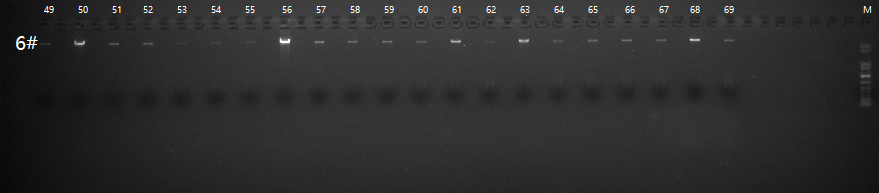   \|  \| \| --- \| \|  \| | | | |
