# Supplementary material for: Assessing the impact of long‐term storage on the quality and integrity of biological specimens in a reproductive biobank
Source: Bioeng Transl Med. 2024 Jun 26;9(6):e10692. doi: 10.1002/btm2.10692 (PMC11558192; doi:10.1002/btm2.10692)
Supplement: Supplementary file 2 — Appendix S2: Supporting information. [file BTM2-9-e10692-s003.docx]

**RNA Sample Quality Inspection Report**

| **Number** | 549 | **SAMPLE TYPES** | Blood |
| --- | --- | --- | --- |
| **Quantitative Method** | **Nanodrop one** | | |
| **Quality Inspection Method** | **Agilent 2100 bioanalyzer** | | |

| **The name of the reagent** | Tianmo#TR205-200 | **Batch Number** | JS206060 |
| --- | --- | --- | --- |
| \| \| **order** \| **Name** \| **concentration (ng/μL)** \| **volume (μL)** \| **total (μg)** \| **A260/ A280** \| **A260/ A230** \| **Electrophoresis** \| \| **Conclusion** \| \| **Remarks** \| \| --- \| --- \| --- \| --- \| --- \| --- \| --- \| --- \| --- \| --- \| --- \| --- \| \| **RIN** \| **28S/**  **18S** \| **Quality** \| **total** \| \| 001 \| B1 \| 2.2 \| 25 \| 0.06 \| 1.15 \| 0.30 \| 2.6 \| 0.0 \| C \| / \| / \| \| 002 \| B2 \| 1.2 \| 25 \| 0.03 \| 1.15 \| 0.35 \| 1.2 \| 0.0 \| C \| / \| / \| \| 003 \| B3 \| 2.4 \| 25 \| 0.06 \| 1.19 \| 0.30 \| 2.6 \| 0.0 \| C \| / \| / \| \| 004 \| B4 \| 2.9 \| 45 \| 0.13 \| 1.23 \| 0.41 \| 2.6 \| 0.0 \| C \| / \| / \| \| 005 \| B5 \| 4.9 \| 45 \| 0.22 \| 1.59 \| 0.83 \| 2.5 \| 0.0 \| C \| / \| / \| \| 006 \| B6 \| 1.2 \| 45 \| 0.05 \| 0.91 \| 0.39 \| 1.0 \| 0.0 \| C \| / \| / \| \| 007 \| B7 \| 2.1 \| 45 \| 0.09 \| 1.31 \| 0.68 \| 2.6 \| 0.0 \| C \| / \| / \| \| 008 \| B8 \| 1.3 \| 45 \| 0.06 \| 0.94 \| 0.53 \| 2.1 \| 0.0 \| C \| / \| / \| \| 009 \| B9 \| 1.2 \| 45 \| 0.05 \| 1.08 \| 0.25 \| 2.0 \| 0.0 \| C \| / \| / \| \| 010 \| B10 \| 1.4 \| 45 \| 0.06 \| 1.29 \| 0.11 \| 2.3 \| 0.0 \| C \| / \| / \| \| 011 \| B11 \| 0.9 \| 45 \| 0.04 \| 0.91 \| 0.49 \| 1.5 \| 0.0 \| C \| / \| / \| \| 012 \| B12 \| 3.7 \| 45 \| 0.17 \| 0.94 \| 0.49 \| 2.6 \| 0.0 \| C \| / \| / \| \| 013 \| B13 \| 2.0 \| 45 \| 0.09 \| 1.27 \| 0.30 \| 1.9 \| 0.0 \| C \| / \| / \| \| 014 \| B14 \| 1.7 \| 45 \| 0.08 \| 1.81 \| 0.65 \| 2.5 \| 0.0 \| C \| / \| / \| \| 015 \| B15 \| 3.5 \| 45 \| 0.16 \| 1.58 \| 1.15 \| 2.5 \| 0.0 \| C \| / \| / \| \| 016 \| B16 \| 1.5 \| 45 \| 0.07 \| 1.35 \| 0.41 \| 2.3 \| 0.0 \| C \| / \| / \| \| 017 \| B17 \| 5.8 \| 45 \| 0.26 \| 1.28 \| 0.47 \| 2.6 \| 0.0 \| C \| / \| / \| \| 018 \| B18 \| 2.1 \| 45 \| 0.10 \| 1.45 \| 0.18 \| 2.5 \| 0.0 \| C \| / \| / \| \| 019 \| B19 \| 0.8 \| 45 \| 0.04 \| 1.26 \| 0.27 \| 1.0 \| 0.0 \| C \| / \| / \| \| 020 \| B20 \| 0.8 \| 45 \| 0.03 \| 1.03 \| 0.21 \| 1.0 \| 0.0 \| C \| / \| / \| \| 021 \| B21 \| 1.6 \| 45 \| 0.07 \| 1.29 \| 0.20 \| 2.5 \| 0.0 \| C \| / \| / \| \| 022 \| B22 \| 0.8 \| 45 \| 0.03 \| 1.14 \| 0.26 \| 1.5 \| 0.0 \| C \| / \| / \| \| 023 \| B23 \| 1.4 \| 45 \| 0.06 \| 1.13 \| 0.16 \| 2.5 \| 0.0 \| C \| / \| / \| \| 024 \| B24 \| 2.6 \| 45 \| 0.12 \| 1.55 \| 0.32 \| 2.4 \| 0.0 \| C \| / \| / \| \| 025 \| B25 \| 1.3 \| 45 \| 0.06 \| 1.31 \| 0.04 \| 2.3 \| 0.0 \| C \| / \| / \| \| 026 \| B26 \| 1.7 \| 45 \| 0.08 \| 1.17 \| 0.34 \| 2.6 \| 0.0 \| C \| / \| / \| \| 027 \| B27 \| 1.2 \| 45 \| 0.05 \| 1.74 \| 0.52 \| 1.1 \| 0.0 \| C \| / \| / \| \| 028 \| B28 \| 3.9 \| 45 \| 0.17 \| 1.15 \| 0.41 \| N/A \| 0.0 \| C \| / \| / \| \| 029 \| B29 \| 2.6 \| 45 \| 0.12 \| 1.24 \| 0.21 \| N/A \| 0.0 \| C \| / \| / \| \| 030 \| B30 \| 3.2 \| 45 \| 0.14 \| 1.73 \| 0.03 \| 1.1 \| 0.0 \| C \| / \| / \| \| 031 \| B31 \| 4.0 \| 45 \| 0.18 \| 1.38 \| 0.41 \| 1.8 \| 0.0 \| C \| / \| / \| \| 032 \| B32 \| 4.1 \| 45 \| 0.19 \| 1.36 \| 0.32 \| 1.2 \| 0.0 \| C \| / \| / \| \| 033 \| B33 \| 4.4 \| 45 \| 0.20 \| 1.29 \| 0.41 \| 1.1 \| 0.0 \| C \| / \| / \| \| 034 \| B34 \| 5.6 \| 45 \| 0.25 \| 1.24 \| 0.40 \| N/A \| 0.0 \| C \| / \| / \| \| 035 \| B35 \| 4.2 \| 45 \| 0.19 \| 1.21 \| 0.36 \| 1.8 \| 0.0 \| C \| / \| / \| \| 036 \| B36 \| 5.3 \| 45 \| 0.24 \| 1.33 \| 0.39 \| 1.9 \| 0.0 \| C \| / \| / \| \| 037 \| B37 \| 3.7 \| 45 \| 0.17 \| 1.44 \| 0.39 \| N/A \| 0.0 \| C \| / \| / \| \| 038 \| B38 \| 3.8 \| 45 \| 0.17 \| 1.31 \| 0.34 \| N/A \| 0.0 \| C \| / \| / \| \| 039 \| B39 \| 4.2 \| 45 \| 0.19 \| 1.34 \| 0.42 \| N/A \| 0.0 \| C \| / \| / \| \| 040 \| B40 \| 4.8 \| 45 \| 0.22 \| 1.47 \| 0.24 \| N/A \| 0.0 \| C \| / \| / \| \| 041 \| B41 \| 3.1 \| 45 \| 0.14 \| 1.60 \| 0.30 \| N/A \| 0.0 \| C \| / \| / \| \| 042 \| B42 \| 4.5 \| 45 \| 0.20 \| 1.46 \| 0.46 \| 1.0 \| 0.0 \| C \| / \| / \| \| 043 \| B43 \| 2.7 \| 45 \| 0.12 \| 1.16 \| 0.25 \| 1.1 \| 0.0 \| C \| / \| / \| \| 044 \| B44 \| 3.9 \| 45 \| 0.18 \| 1.31 \| 0.44 \| 1.1 \| 0.0 \| C \| / \| / \| \| 045 \| B45 \| 4.0 \| 45 \| 0.18 \| 1.14 \| 0.42 \| 1.8 \| 0.0 \| C \| / \| / \| \| 046 \| B46 \| 3.5 \| 45 \| 0.16 \| 1.36 \| 0.23 \| N/A \| 0.0 \| C \| / \| / \| \| 047 \| B47 \| 4.3 \| 45 \| 0.20 \| 1.45 \| 0.43 \| N/A \| 0.0 \| C \| / \| / \| \| 048 \| B48 \| 6.3 \| 45 \| 0.28 \| 1.31 \| 0.57 \| N/A \| 0.0 \| C \| / \| / \| \| 049 \| B49 \| 3.4 \| 45 \| 0.15 \| 1.33 \| 0.22 \| N/A \| 0.0 \| C \| / \| / \| \| 050 \| B50 \| 3.5 \| 45 \| 0.16 \| 1.57 \| 0.38 \| 1.0 \| 0.0 \| C \| / \| / \| \| 051 \| B51 \| 19.0 \| 25 \| 0.48 \| 1.73 \| 0.68 \| 3.8 \| 0.6 \| C \| / \| / \| \| 052 \| B52 \| 9.2 \| 25 \| 0.23 \| 1.85 \| 0.94 \| 3.5 \| 0.8 \| C \| / \| / \| \| 053 \| B53 \| 16.3 \| 25 \| 0.41 \| 1.83 \| 1.41 \| 2.6 \| 0.7 \| C \| / \| / \| \| 054 \| B54 \| 2.0 \| 45 \| 0.09 \| 1.72 \| 0.09 \| 1.3 \| 0.0 \| C \| / \| / \| \| 055 \| B55 \| 12.8 \| 45 \| 0.58 \| 1.75 \| 1.28 \| 3.2 \| 1.2 \| C \| / \| / \| \| 056 \| B56 \| 4.4 \| 45 \| 0.20 \| 1.98 \| 1.59 \| 2.1 \| 0.0 \| C \| / \| / \| \| 057 \| B57 \| 13.9 \| 45 \| 0.63 \| 1.55 \| 0.68 \| 2.4 \| 0.8 \| C \| / \| / \| \| 058 \| B58 \| 20.5 \| 45 \| 0.92 \| 2.00 \| 1.58 \| 3.2 \| 0.9 \| C \| / \| / \| \| 059 \| B59 \| 5.9 \| 45 \| 0.27 \| 2.00 \| 1.47 \| 2.7 \| 0.4 \| C \| / \| / \| \| 060 \| B60 \| 22.6 \| 45 \| 1.02 \| 1.92 \| 1.37 \| 2.4 \| 0.1 \| C \| / \| / \| \| 061 \| B61 \| 5.5 \| 45 \| 0.25 \| 1.74 \| 1.01 \| 2.7 \| 0.7 \| C \| / \| / \| \| 062 \| B62 \| 11.2 \| 45 \| 0.50 \| 1.86 \| 0.99 \| 2.0 \| 0.1 \| C \| / \| / \| \| 063 \| B63 \| 6.8 \| 45 \| 0.31 \| 1.66 \| 0.70 \| 2.4 \| 0.7 \| C \| / \| / \| \| 064 \| B64 \| 9.9 \| 45 \| 0.45 \| 1.86 \| 0.98 \| 2.6 \| 1.5 \| C \| / \| / \| \| 065 \| B65 \| 8.7 \| 45 \| 0.39 \| 1.85 \| 1.24 \| 2.6 \| 0.2 \| C \| / \| / \| \| 066 \| B66 \| 5.3 \| 45 \| 0.24 \| 1.85 \| 1.30 \| 2.7 \| 0.3 \| C \| / \| / \| \| 067 \| B67 \| 10.8 \| 45 \| 0.49 \| 1.67 \| 0.81 \| 2.6 \| 0.6 \| C \| / \| / \| \| 068 \| B68 \| 21.6 \| 45 \| 0.97 \| 1.89 \| 1.40 \| 2.5 \| 0.4 \| C \| / \| / \| \| 069 \| B69 \| 16.2 \| 45 \| 0.73 \| 1.80 \| 1.31 \| 2.6 \| 0.4 \| C \| / \| / \| \| 070 \| B70 \| 5.7 \| 45 \| 0.26 \| 1.70 \| 0.43 \| 4.9 \| 1.1 \| C \| / \| / \| \| 071 \| B71 \| 4.0 \| 45 \| 0.18 \| 1.63 \| 0.44 \| 2.5 \| 0.0 \| C \| / \| / \| \| 072 \| B72 \| 3.7 \| 45 \| 0.17 \| 1.57 \| 1.27 \| 2.4 \| 0.2 \| C \| / \| / \| \| 073 \| B73 \| 26.8 \| 45 \| 1.21 \| 1.80 \| 1.28 \| 2.7 \| 0.1 \| C \| / \| / \| \| 074 \| B74 \| 19.6 \| 45 \| 0.88 \| 1.81 \| 1.24 \| 2.5 \| 0.0 \| C \| / \| / \| \| 075 \| B75 \| 4.0 \| 45 \| 0.18 \| 1.41 \| 0.35 \| 2.6 \| 0.0 \| C \| / \| / \| \| 076 \| B76 \| 17.1 \| 45 \| 0.77 \| 1.84 \| 1.44 \| 2.6 \| 0.3 \| C \| / \| / \| \| 077 \| B77 \| 4.3 \| 45 \| 0.20 \| 1.47 \| 0.45 \| 2.3 \| 0.0 \| C \| / \| / \| \| 078 \| B78 \| 24.6 \| 45 \| 1.11 \| 1.81 \| 1.27 \| 2.2 \| 0.4 \| C \| / \| / \| \| 079 \| B79 \| 14.8 \| 45 \| 0.67 \| 1.52 \| 0.56 \| 2.3 \| 0.1 \| C \| / \| / \| \| 080 \| B80 \| 4.9 \| 45 \| 0.22 \| 1.20 \| 0.28 \| 2.3 \| 0.0 \| C \| / \| / \| \| 081 \| B81 \| 30.2 \| 45 \| 1.36 \| 1.52 \| 0.62 \| 2.5 \| 0.3 \| C \| / \| / \| \| 082 \| B82 \| 22.1 \| 45 \| 1.00 \| 1.93 \| 1.54 \| 2.2 \| 0.2 \| C \| / \| / \| \| 083 \| B83 \| 7.3 \| 45 \| 0.33 \| 1.71 \| 0.72 \| 2.2 \| 0.0 \| C \| / \| / \| \| 084 \| B84 \| 17.9 \| 45 \| 0.81 \| 1.89 \| 1.34 \| 2.2 \| 0.1 \| C \| / \| / \| \| 085 \| B85 \| 8.6 \| 45 \| 0.38 \| 1.84 \| 1.34 \| 2.7 \| 0.2 \| C \| / \| / \| \| 086 \| B86 \| 7.2 \| 45 \| 0.33 \| 1.99 \| 0.47 \| 2.3 \| 0.1 \| C \| / \| / \| \| 087 \| B87 \| 12.3 \| 45 \| 0.55 \| 1.70 \| 0.89 \| 2.2 \| 0.0 \| C \| / \| / \| \| 088 \| B88 \| 6.5 \| 45 \| 0.29 \| 1.56 \| 0.58 \| 2.6 \| 0.2 \| C \| / \| / \| \| 089 \| B89 \| 8.7 \| 45 \| 0.39 \| 1.78 \| 0.79 \| 2.3 \| 0.0 \| C \| / \| / \| \| 090 \| B90 \| 3.9 \| 45 \| 0.18 \| 1.52 \| 0.61 \| 2.7 \| 0.3 \| C \| / \| / \| \| 091 \| B91 \| 9.0 \| 45 \| 0.40 \| 1.67 \| 0.61 \| 2.5 \| 0.1 \| C \| / \| / \| \| 092 \| B92 \| 4.7 \| 45 \| 0.21 \| 1.56 \| 0.64 \| 2.3 \| 0.0 \| C \| / \| / \| \| 093 \| B93 \| 4.0 \| 45 \| 0.18 \| 1.38 \| 0.39 \| 2.4 \| 0.0 \| C \| / \| / \| \| 094 \| B94 \| 30.3 \| 45 \| 1.36 \| 1.71 \| 0.86 \| 2.2 \| 0.1 \| C \| / \| / \| \| 095 \| B95 \| 4.8 \| 45 \| 0.22 \| 1.98 \| 0.86 \| 2.7 \| 0.2 \| C \| / \| / \| \| 096 \| B96 \| 8.1 \| 45 \| 0.36 \| 1.73 \| 0.67 \| 2.3 \| 0.1 \| C \| / \| / \| \| 097 \| B97 \| 11.9 \| 45 \| 0.53 \| 1.66 \| 0.79 \| 2.6 \| 0.2 \| C \| / \| / \| \| 098 \| B98 \| 2.6 \| 45 \| 0.12 \| 1.62 \| 0.15 \| 1.1 \| 0.0 \| C \| / \| / \| \| 099 \| B99 \| 11.6 \| 45 \| 0.52 \| 1.71 \| 1.13 \| 2.6 \| 0.4 \| C \| / \| / \| \| 100 \| B100 \| 14.6 \| 45 \| 0.66 \| 1.61 \| 0.91 \| 2.6 \| 0.3 \| C \| / \| / \| \| 101 \| B101 \| 2.0 \| 45 \| 0.09 \| 1.63 \| 0.62 \| 1.4 \| 0.0 \| C \| / \| / \| \| 102 \| B102 \| 13.8 \| 45 \| 0.62 \| 1.61 \| 0.82 \| 5.8 \| 0.7 \| B1 \| / \| / \| \| 103 \| B103 \| 104.8 \| 45 \| 4.71 \| 1.58 \| 0.74 \| 4.8 \| 0.9 \| C \| / \| / \| \| 104 \| B104 \| 29.6 \| 45 \| 1.33 \| 1.66 \| 0.92 \| 3.6 \| 0.8 \| C \| / \| / \| \| 105 \| B105 \| 111.4 \| 45 \| 5.01 \| 1.57 \| 0.73 \| 3.5 \| 0.6 \| C \| / \| / \| \| 106 \| B106 \| 72.4 \| 45 \| 3.26 \| 1.91 \| 1.61 \| 5.7 \| 0.7 \| B1 \| / \| / \| \| 107 \| B107 \| 65.6 \| 45 \| 2.95 \| 1.62 \| 0.85 \| 3.0 \| 0.7 \| C \| / \| / \| \| 108 \| B108 \| 49.4 \| 45 \| 2.22 \| 1.82 \| 1.24 \| 3.1 \| 0.5 \| C \| / \| / \| \| 109 \| B109 \| 98.4 \| 45 \| 4.43 \| 1.57 \| 0.78 \| 4.4 \| 0.8 \| C \| / \| / \| \| 110 \| B110 \| 36.8 \| 45 \| 1.66 \| 1.72 \| 0.98 \| 5.3 \| 0.6 \| B1 \| / \| / \| \| 111 \| B111 \| 11.6 \| 45 \| 0.52 \| 1.76 \| 1.40 \| 6.4 \| 1.0 \| A1 \| / \| / \| \| 112 \| B112 \| 41.4 \| 45 \| 1.86 \| 1.62 \| 0.77 \| 5.0 \| 1.2 \| B1 \| / \| / \| \| 113 \| B113 \| 67.4 \| 45 \| 3.03 \| 1.90 \| 1.48 \| 3.4 \| 0.8 \| C \| / \| / \| \| 114 \| B114 \| 38.5 \| 45 \| 1.73 \| 1.81 \| 1.28 \| 4.2 \| 0.8 \| C \| / \| / \| \| 115 \| B115 \| 43.6 \| 45 \| 1.96 \| 1.91 \| 1.65 \| 3.9 \| 0.7 \| C \| / \| / \| \| 116 \| B116 \| 17.2 \| 45 \| 0.77 \| 1.60 \| 0.87 \| 6.1 \| 0.8 \| A1 \| / \| / \| \| 117 \| B117 \| 88.9 \| 45 \| 4.00 \| 1.70 \| 0.94 \| 4.3 \| 1.0 \| C \| / \| / \| \| 118 \| B118 \| 33.4 \| 45 \| 1.50 \| 1.92 \| 1.70 \| 5.0 \| 1.3 \| B1 \| / \| / \| \| 119 \| B119 \| 33.0 \| 45 \| 1.49 \| 1.78 \| 0.93 \| 4.5 \| 1.1 \| C \| / \| / \| \| 120 \| B120 \| 43.9 \| 45 \| 1.97 \| 1.79 \| 1.20 \| 3.8 \| 0.8 \| C \| / \| / \| \| 121 \| B121 \| 17.1 \| 45 \| 0.77 \| 1.78 \| 0.96 \| 2.3 \| 0.3 \| C \| / \| / \| \| 122 \| B122 \| 18.8 \| 45 \| 0.85 \| 1.91 \| 0.77 \| 3.4 \| 0.9 \| C \| / \| / \| \| 123 \| B123 \| 29.7 \| 45 \| 1.34 \| 1.89 \| 1.33 \| 2.4 \| 0.2 \| C \| / \| / \| \| 124 \| B124 \| 41.8 \| 45 \| 1.88 \| 1.86 \| 1.23 \| 4.0 \| 0.7 \| C \| / \| / \| \| 125 \| B125 \| 18.4 \| 45 \| 0.83 \| 1.64 \| 0.54 \| 1.8 \| 0.0 \| C \| / \| / \| \| 126 \| B126 \| 28.6 \| 45 \| 1.28 \| 1.75 \| 0.90 \| 2.3 \| 0.3 \| C \| / \| / \| \| 127 \| B127 \| 27.3 \| 45 \| 1.23 \| 1.79 \| 1.10 \| 2.6 \| 0.7 \| C \| / \| / \| \| 128 \| B128 \| 14.7 \| 45 \| 0.66 \| 1.65 \| 0.87 \| 2.6 \| 0.3 \| C \| / \| / \| \| 129 \| B129 \| 39.3 \| 45 \| 1.77 \| 1.90 \| 1.69 \| 2.9 \| 0.9 \| C \| / \| / \| \| 130 \| B130 \| 40.1 \| 45 \| 1.81 \| 1.86 \| 1.22 \| 4.0 \| 0.6 \| C \| / \| / \| \| 131 \| B131 \| 17.0 \| 45 \| 0.77 \| 1.86 \| 1.33 \| 4.7 \| 0.7 \| C \| / \| / \| \| 132 \| B132 \| 44.9 \| 45 \| 2.02 \| 1.90 \| 1.45 \| 2.6 \| 0.6 \| C \| / \| / \| \| 133 \| B133 \| 11.9 \| 45 \| 0.54 \| 1.55 \| 0.70 \| 5.5 \| 0.8 \| B1 \| / \| / \| \| 134 \| B134 \| 21.0 \| 45 \| 0.95 \| 1.73 \| 0.81 \| 5.9 \| 0.7 \| B1 \| / \| / \| \| 135 \| B135 \| 15.0 \| 45 \| 0.68 \| 1.72 \| 0.91 \| 5.2 \| 0.6 \| B1 \| / \| / \| \| 136 \| B136 \| 26.4 \| 45 \| 1.19 \| 1.96 \| 1.14 \| 5.6 \| 0.9 \| B1 \| / \| / \| \| 137 \| B137 \| 94.6 \| 45 \| 4.26 \| 1.71 \| 0.95 \| 4.5 \| 0.6 \| C \| / \| / \| \| 138 \| B138 \| 47.2 \| 45 \| 2.12 \| 1.68 \| 0.96 \| 5.2 \| 0.7 \| B1 \| / \| / \| \| 139 \| B139 \| 29.4 \| 45 \| 1.32 \| 1.65 \| 0.79 \| 5.9 \| 0.7 \| B1 \| / \| / \| \| 140 \| B140 \| 13.5 \| 45 \| 0.61 \| 1.68 \| 0.34 \| 5.6 \| 0.6 \| B1 \| / \| / \| \| 141 \| B141 \| 16.4 \| 45 \| 0.74 \| 1.47 \| 0.64 \| 5.7 \| 1.1 \| B1 \| / \| / \| \| 142 \| B142 \| 26.8 \| 45 \| 1.21 \| 1.94 \| 1.28 \| 6.8 \| 1.4 \| A1 \| / \| / \| \| 143 \| B143 \| 11.3 \| 45 \| 0.51 \| 1.79 \| 1.19 \| 6.4 \| 1.1 \| A1 \| / \| / \| \| 144 \| B144 \| 24.2 \| 45 \| 1.09 \| 1.71 \| 0.88 \| 5.5 \| 1.0 \| B1 \| / \| / \| \| 145 \| B145 \| 104.7 \| 45 \| 4.71 \| 1.96 \| 1.84 \| 2.8 \| 0.7 \| C \| / \| / \| \| 146 \| B146 \| 47.8 \| 45 \| 2.15 \| 1.94 \| 1.20 \| 3.9 \| 1.1 \| C \| / \| / \| \| 147 \| B147 \| 9.5 \| 45 \| 0.43 \| 1.73 \| 0.98 \| 5.8 \| 0.6 \| B1 \| / \| / \| \| 148 \| B148 \| 20.0 \| 45 \| 0.90 \| 1.67 \| 0.87 \| 6.5 \| 0.8 \| A1 \| / \| / \| \| 149 \| B149 \| 11.0 \| 45 \| 0.49 \| 1.60 \| 0.33 \| 4.9 \| 0.4 \| C \| / \| / \| \| 150 \| B150 \| 52.2 \| 45 \| 2.35 \| 1.94 \| 1.55 \| 4.3 \| 0.8 \| C \| / \| / \| \| 151 \| B151 \| 47.2 \| 45 \| 2.12 \| 1.88 \| 0.64 \| 3.9 \| 0.8 \| C \| / \| / \| \| 152 \| B152 \| 31.6 \| 45 \| 1.42 \| 1.68 \| 0.57 \| 6.1 \| 1.2 \| A1 \| / \| / \| \| 153 \| B153 \| 36.0 \| 45 \| 1.62 \| 1.98 \| 0.96 \| 6.2 \| 0.8 \| A1 \| / \| / \| \| 154 \| B154 \| 36.0 \| 45 \| 1.62 \| 1.93 \| 1.52 \| 5.3 \| 0.8 \| B1 \| / \| / \| \| 155 \| B155 \| 38.0 \| 45 \| 1.71 \| 1.85 \| 1.00 \| 5.0 \| 0.9 \| B1 \| / \| / \| \| 156 \| B156 \| 21.2 \| 45 \| 0.95 \| 1.72 \| 0.97 \| 6.1 \| 0.9 \| A1 \| / \| / \| \| 157 \| B157 \| 24.8 \| 45 \| 1.12 \| 1.63 \| 0.92 \| 6.0 \| 0.8 \| A1 \| / \| / \| \| 158 \| B158 \| 160.6 \| 45 \| 7.23 \| 1.55 \| 0.78 \| 2.6 \| 0.5 \| C \| / \| / \| \| 159 \| B159 \| 13.0 \| 45 \| 0.59 \| 1.77 \| 0.74 \| 4.5 \| 0.7 \| C \| / \| / \| \| 160 \| B160 \| 16.4 \| 45 \| 0.74 \| 1.84 \| 1.41 \| 6.1 \| 1.2 \| A1 \| / \| / \| \| 161 \| B161 \| 34.7 \| 45 \| 1.56 \| 1.82 \| 1.28 \| 5.3 \| 0.6 \| B1 \| / \| / \| \| 162 \| B162 \| 86.3 \| 45 \| 3.88 \| 1.68 \| 0.87 \| 4.2 \| 0.5 \| C \| / \| / \| \| 163 \| B163 \| 74.3 \| 45 \| 3.34 \| 1.67 \| 0.81 \| 2.8 \| 0.6 \| C \| / \| / \| \| 164 \| B164 \| 28.7 \| 45 \| 1.29 \| 1.99 \| 1.93 \| 6.1 \| 0.6 \| A1 \| / \| / \| \| 165 \| B165 \| 44.7 \| 45 \| 2.01 \| 1.84 \| 1.16 \| 5.7 \| 0.8 \| B1 \| / \| / \| \| 166 \| B166 \| 32.3 \| 45 \| 1.45 \| 1.86 \| 1.33 \| 3.3 \| 0.7 \| C \| / \| / \| \| 167 \| B167 \| 80.9 \| 45 \| 3.64 \| 1.99 \| 2.03 \| 2.9 \| 0.6 \| C \| / \| / \| \| 168 \| B168 \| 35.8 \| 45 \| 1.61 \| 1.80 \| 1.10 \| 4.8 \| 0.8 \| C \| / \| / \| \| 169 \| B169 \| 12.9 \| 45 \| 0.58 \| 1.47 \| 0.55 \| 2.5 \| 0.0 \| C \| / \| / \| \| 170 \| B170 \| 60.4 \| 45 \| 2.72 \| 1.79 \| 1.19 \| 4.9 \| 0.8 \| C \| / \| / \| \| 171 \| B171 \| 39.6 \| 45 \| 1.78 \| 1.79 \| 0.98 \| 4.8 \| 0.4 \| C \| / \| / \| \| 172 \| B172 \| 25.5 \| 45 \| 1.15 \| 1.92 \| 1.16 \| 6.2 \| 1.1 \| A1 \| / \| / \| \| 173 \| B173 \| 30.5 \| 45 \| 1.37 \| 1.84 \| 1.23 \| 6.3 \| 0.7 \| A1 \| / \| / \| \| 174 \| B174 \| 21.2 \| 45 \| 0.95 \| 1.74 \| 0.98 \| 6.6 \| 0.9 \| A1 \| / \| / \| \| 175 \| B175 \| 34.8 \| 45 \| 1.57 \| 1.85 \| 1.41 \| 4.9 \| 0.6 \| C \| / \| / \| \| 176 \| B176 \| 31.6 \| 45 \| 1.42 \| 1.71 \| 0.88 \| 6.1 \| 0.7 \| A1 \| / \| / \| \| 177 \| B177 \| 84.5 \| 45 \| 3.80 \| 1.59 \| 0.78 \| 4.4 \| 0.8 \| C \| / \| / \| \| 178 \| B178 \| 25.4 \| 45 \| 1.14 \| 1.90 \| 1.34 \| 2.3 \| 0.9 \| C \| / \| / \| \| 179 \| B179 \| 71.9 \| 45 \| 3.23 \| 2.01 \| 1.75 \| 2.7 \| 0.5 \| C \| / \| / \| \| 180 \| B180 \| 43.8 \| 45 \| 1.97 \| 1.94 \| 1.62 \| 2.8 \| 0.6 \| C \| / \| / \| \| 181 \| B181 \| 47.0 \| 45 \| 2.11 \| 1.97 \| 1.82 \| 2.5 \| 0.9 \| C \| / \| / \| \| 182 \| B182 \| 12.7 \| 45 \| 0.57 \| 1.95 \| 1.09 \| 2.6 \| 1.1 \| C \| / \| / \| \| 183 \| B183 \| 1.9 \| 45 \| 0.08 \| 1.92 \| 0.40 \| 2.7 \| 0.2 \| C \| / \| / \| \| 184 \| B184 \| 27.0 \| 45 \| 1.21 \| 1.75 \| 0.93 \| 4.8 \| 0.8 \| C \| / \| / \| \| 185 \| B185 \| 34.9 \| 45 \| 1.57 \| 1.91 \| 1.37 \| 2.6 \| 0.5 \| C \| / \| / \| \| 186 \| B186 \| 57.5 \| 45 \| 2.59 \| 1.82 \| 1.19 \| 3.8 \| 0.6 \| C \| / \| / \| \| 187 \| B187 \| 36.3 \| 45 \| 1.63 \| 1.72 \| 0.94 \| 3.0 \| 0.5 \| C \| / \| / \| \| 188 \| B188 \| 31.1 \| 45 \| 1.40 \| 1.61 \| 0.77 \| 6.8 \| 1.1 \| A1 \| / \| / \| \| 189 \| B189 \| 68.5 \| 45 \| 3.08 \| 1.82 \| 1.27 \| 5.3 \| 0.6 \| B1 \| / \| / \| \| 190 \| B190 \| 20.7 \| 45 \| 0.93 \| 1.78 \| 1.20 \| 6.2 \| 0.9 \| A1 \| / \| / \| \| 191 \| B191 \| 36.9 \| 45 \| 1.66 \| 2.00 \| 1.76 \| 4.7 \| 0.7 \| C \| / \| / \| \| 192 \| B192 \| 68.9 \| 45 \| 3.10 \| 1.67 \| 0.85 \| 5.0 \| 0.7 \| B1 \| / \| / \| \| 193 \| B193 \| 28.3 \| 45 \| 1.27 \| 1.96 \| 1.79 \| 5.8 \| 1.0 \| B1 \| / \| / \| \| 194 \| B194 \| 41.6 \| 45 \| 1.87 \| 1.91 \| 1.10 \| 5.9 \| 0.9 \| B1 \| / \| / \| \| 195 \| B195 \| 88.2 \| 45 \| 3.97 \| 2.02 \| 1.65 \| 3.1 \| 0.7 \| C \| / \| / \| \| 196 \| B196 \| 59.7 \| 45 \| 2.68 \| 1.97 \| 1.89 \| 4.9 \| 1.1 \| C \| / \| / \| \| 197 \| B197 \| 53.7 \| 45 \| 2.42 \| 1.99 \| 0.43 \| 6.0 \| 0.9 \| A1 \| / \| / \| \| 198 \| B198 \| 29.1 \| 45 \| 1.31 \| 2.06 \| 1.14 \| 3.5 \| 0.8 \| C \| / \| / \| \| 199 \| B199 \| 34.7 \| 45 \| 1.56 \| 1.99 \| 1.68 \| 2.6 \| 0.4 \| C \| / \| / \| \| 200 \| B200 \| 94.4 \| 45 \| 4.25 \| 1.97 \| 1.78 \| 2.4 \| 0.8 \| C \| / \| / \| \| 201 \| B201 \| 48.4 \| 45 \| 2.18 \| 1.98 \| 1.67 \| 2.4 \| 0.3 \| C \| / \| / \| \| 202 \| B202 \| 20.0 \| 45 \| 0.90 \| 1.91 \| 0.53 \| 3.8 \| 0.6 \| C \| / \| / \| \| 203 \| B203 \| 28.7 \| 45 \| 1.29 \| 2.01 \| 0.82 \| 3.8 \| 0.9 \| C \| / \| / \| \| 204 \| B204 \| 56.4 \| 45 \| 2.54 \| 1.80 \| 0.78 \| 2.9 \| 0.8 \| C \| / \| / \| \| 205 \| B205 \| 28.2 \| 45 \| 1.27 \| 2.08 \| 0.22 \| 5.0 \| 0.9 \| B1 \| / \| / \| \| 206 \| B206 \| 36.9 \| 45 \| 1.66 \| 1.98 \| 0.96 \| 5.6 \| 1.0 \| B1 \| / \| / \| \| 207 \| B207 \| 26.7 \| 45 \| 1.20 \| 1.99 \| 1.49 \| 2.5 \| 0.9 \| C \| / \| / \| \| 208 \| B208 \| 63.8 \| 45 \| 2.87 \| 1.88 \| 1.13 \| 3.0 \| 0.7 \| C \| / \| / \| \| 209 \| B209 \| 21.2 \| 45 \| 0.95 \| 2.02 \| 1.58 \| 3.7 \| 0.6 \| C \| / \| / \| \| 210 \| B210 \| 34.9 \| 45 \| 1.57 \| 1.83 \| 1.15 \| 3.8 \| 0.6 \| C \| / \| / \| \| 211 \| B211 \| 13.7 \| 45 \| 0.62 \| 1.90 \| 0.77 \| 3.6 \| 0.6 \| C \| / \| / \| \| 212 \| B212 \| 40.0 \| 45 \| 1.80 \| 1.96 \| 1.69 \| 4.5 \| 0.9 \| C \| / \| / \| \| 213 \| B213 \| 53.1 \| 45 \| 2.39 \| 1.82 \| 1.10 \| 4.8 \| 0.8 \| C \| / \| / \| \| 214 \| B214 \| 28.6 \| 45 \| 1.29 \| 2.01 \| 1.64 \| 2.8 \| 0.7 \| C \| / \| / \| \| 215 \| B215 \| 11.0 \| 45 \| 0.49 \| 1.85 \| 0.36 \| 5.1 \| 0.7 \| B1 \| / \| / \| \| 216 \| B216 \| 32.7 \| 45 \| 1.47 \| 1.72 \| 0.58 \| 5.9 \| 0.6 \| B1 \| / \| / \| \| 217 \| B217 \| 51.5 \| 45 \| 2.32 \| 1.90 \| 1.36 \| 3.8 \| 0.7 \| C \| / \| / \| \| 218 \| B218 \| 47.3 \| 45 \| 2.13 \| 1.76 \| 1.13 \| 5.9 \| 0.9 \| B1 \| / \| / \| \| 219 \| B219 \| 23.7 \| 45 \| 1.07 \| 1.68 \| 0.98 \| 4.8 \| 0.6 \| C \| / \| / \| \| 220 \| B220 \| 45.7 \| 45 \| 2.06 \| 1.84 \| 1.07 \| 2.3 \| 0.1 \| C \| / \| / \| \| 221 \| B221 \| 62.6 \| 45 \| 2.81 \| 1.69 \| 0.90 \| 2.7 \| 0.7 \| C \| / \| / \| \| 222 \| B222 \| 32.2 \| 45 \| 1.45 \| 1.79 \| 1.02 \| 5.5 \| 0.9 \| B1 \| / \| / \| \| 223 \| B223 \| 60.0 \| 45 \| 2.70 \| 1.70 \| 0.92 \| 4.1 \| 0.5 \| C \| / \| / \| \| 224 \| B224 \| 16.0 \| 45 \| 0.72 \| 1.91 \| 1.82 \| 4.7 \| 0.9 \| C \| / \| / \| \| 225 \| B225 \| 14.6 \| 45 \| 0.66 \| 1.75 \| 1.17 \| 5.1 \| 0.9 \| B1 \| / \| / \| \| 226 \| B226 \| 20.5 \| 45 \| 0.92 \| 1.61 \| 0.83 \| 6.1 \| 0.7 \| A1 \| / \| / \| \| 227 \| B227 \| 36.6 \| 45 \| 1.65 \| 1.70 \| 0.99 \| 5.8 \| 0.9 \| B1 \| / \| / \| \| 228 \| B228 \| 96.8 \| 45 \| 4.35 \| 1.53 \| 0.76 \| 4.6 \| 0.8 \| C \| / \| / \| \| 229 \| B229 \| 63.2 \| 45 \| 2.84 \| 1.91 \| 1.59 \| 4.6 \| 0.8 \| C \| / \| / \| \| 230 \| B230 \| 23.2 \| 45 \| 1.04 \| 1.75 \| 1.03 \| 6.0 \| 0.9 \| A1 \| / \| / \| \| 231 \| B231 \| 38.6 \| 45 \| 1.74 \| 1.71 \| 0.87 \| 5.7 \| 1.1 \| B1 \| / \| / \| \| 232 \| B232 \| 37.7 \| 45 \| 1.70 \| 1.74 \| 0.92 \| 6.5 \| 1.0 \| A1 \| / \| / \| \| 233 \| B233 \| 44.1 \| 45 \| 1.98 \| 1.71 \| 0.94 \| 2.8 \| 1.4 \| C \| / \| / \| \| 234 \| B234 \| 47.2 \| 45 \| 2.12 \| 1.95 \| 1.66 \| 4.9 \| 0.9 \| C \| / \| / \| \| 235 \| B235 \| 35.9 \| 45 \| 1.62 \| 1.76 \| 0.36 \| 2.7 \| 0.9 \| C \| / \| / \| \| 236 \| B236 \| 85.0 \| 45 \| 3.82 \| 1.58 \| 0.75 \| 4.4 \| 0.8 \| C \| / \| / \| \| 237 \| B237 \| 14.4 \| 45 \| 0.65 \| 1.87 \| 1.42 \| 3.4 \| 0.8 \| C \| / \| / \| \| 238 \| B238 \| 55.6 \| 45 \| 2.50 \| 1.67 \| 0.46 \| 4.2 \| 0.6 \| C \| / \| / \| \| 239 \| B239 \| 0.9 \| 45 \| 0.04 \| 1.58 \| 0.30 \| 1.0 \| 0.0 \| C \| / \| / \| \| 240 \| B240 \| 53.4 \| 45 \| 2.40 \| 1.67 \| 0.85 \| 5.1 \| 0.7 \| B1 \| / \| / \| \| 241 \| B241 \| 46.5 \| 45 \| 2.09 \| 1.79 \| 1.13 \| 2.9 \| 0.7 \| C \| / \| / \| \| 242 \| B242 \| 15.2 \| 45 \| 0.68 \| 1.72 \| 0.90 \| 3.3 \| 0.6 \| C \| / \| / \| \| 243 \| B243 \| 31.6 \| 45 \| 1.42 \| 1.86 \| 1.37 \| 4.8 \| 0.9 \| C \| / \| / \| \| 244 \| B244 \| 20.5 \| 45 \| 0.92 \| 1.70 \| 0.21 \| 5.0 \| 1.0 \| B1 \| / \| / \| \| 245 \| B245 \| 57.8 \| 45 \| 2.60 \| 1.83 \| 1.31 \| 4.2 \| 0.6 \| C \| / \| / \| \| 246 \| B246 \| 33.9 \| 45 \| 1.52 \| 1.75 \| 1.18 \| 4.0 \| 1.0 \| C \| / \| / \| \| 247 \| B247 \| 29.9 \| 45 \| 1.35 \| 1.87 \| 1.51 \| 4.6 \| 0.9 \| C \| / \| / \| \| 248 \| B248 \| 47.5 \| 45 \| 2.14 \| 1.83 \| 1.40 \| 5.4 \| 0.9 \| B1 \| / \| / \| \| 249 \| B249 \| 42.6 \| 45 \| 1.92 \| 1.87 \| 1.36 \| 3.5 \| 0.7 \| C \| / \| / \| \| 250 \| B250 \| 38.7 \| 45 \| 1.74 \| 1.95 \| 1.88 \| 2.7 \| 0.9 \| C \| / \| / \| \| 251 \| B251 \| 30.6 \| 45 \| 1.38 \| 1.79 \| 1.14 \| 4.8 \| 0.7 \| C \| / \| / \| \| 252 \| B252 \| 13.1 \| 45 \| 0.59 \| 1.67 \| 1.06 \| 3.7 \| 0.6 \| C \| / \| / \| \| 253 \| B253 \| 26.8 \| 45 \| 1.21 \| 1.98 \| 1.84 \| 5.7 \| 1.0 \| B1 \| / \| / \| \| 254 \| B254 \| 97.9 \| 45 \| 4.41 \| 1.66 \| 0.89 \| 2.8 \| 1.1 \| C \| / \| / \| \| 255 \| B255 \| 30.4 \| 45 \| 1.37 \| 1.64 \| 0.87 \| 6.8 \| 1.0 \| A1 \| / \| / \| \| 256 \| B256 \| 82.0 \| 45 \| 3.69 \| 1.48 \| 0.68 \| 6.3 \| 0.7 \| A1 \| / \| / \| \| 257 \| B257 \| 14.7 \| 45 \| 0.66 \| 1.74 \| 1.13 \| 6.9 \| 0.9 \| A1 \| / \| / \| \| 258 \| B258 \| 16.8 \| 45 \| 0.76 \| 1.62 \| 0.88 \| 6.4 \| 0.6 \| A1 \| / \| / \| \| 259 \| B259 \| 60.9 \| 45 \| 2.74 \| 1.67 \| 0.83 \| 6.6 \| 1.0 \| A1 \| / \| / \| \| 260 \| B260 \| 28.1 \| 45 \| 1.26 \| 1.65 \| 0.87 \| 6.9 \| 1.0 \| A1 \| / \| / \| \| 261 \| B261 \| 41.1 \| 45 \| 1.85 \| 1.60 \| 0.77 \| 5.8 \| 0.8 \| B1 \| / \| / \| \| 262 \| B262 \| 26.6 \| 45 \| 1.20 \| 1.61 \| 0.78 \| 7.0 \| 0.7 \| A \| / \| / \| \| 263 \| B263 \| 33.4 \| 45 \| 1.50 \| 1.95 \| 1.63 \| 6.7 \| 0.8 \| A1 \| / \| / \| \| 264 \| B264 \| 86.3 \| 45 \| 3.88 \| 1.53 \| 0.71 \| 6.2 \| 0.9 \| A1 \| / \| / \| \| 265 \| B265 \| 55.5 \| 45 \| 2.50 \| 1.61 \| 0.71 \| 6.5 \| 0.7 \| A1 \| / \| / \| \| 266 \| B266 \| 137.0 \| 45 \| 6.16 \| 1.52 \| 0.68 \| N/A \| 1.0 \| B1 \| / \| / \| \| 267 \| B267 \| 69.5 \| 45 \| 3.13 \| 1.63 \| 0.78 \| 6.5 \| 0.8 \| A1 \| / \| / \| \| 268 \| B268 \| 10.9 \| 45 \| 0.49 \| 2.08 \| 1.21 \| N/A \| 1.0 \| B1 \| / \| / \| \| 269 \| B269 \| 11.9 \| 45 \| 0.53 \| 2.03 \| 0.25 \| N/A \| 0.9 \| B1 \| / \| / \| \| 270 \| B270 \| 77.3 \| 45 \| 3.48 \| 1.58 \| 0.73 \| 3.6 \| 0.8 \| C \| / \| / \| \| 271 \| B271 \| 46.0 \| 45 \| 2.07 \| 1.97 \| 1.70 \| 4.6 \| 1.0 \| C \| / \| / \| \| 272 \| B272 \| 75.7 \| 45 \| 3.41 \| 1.76 \| 0.89 \| 6.3 \| 0.7 \| A1 \| / \| / \| \| 273 \| B273 \| 8.1 \| 45 \| 0.36 \| 1.82 \| 1.02 \| N/A \| 1.0 \| B1 \| / \| / \| \| 274 \| B274 \| 25.8 \| 45 \| 1.16 \| 1.72 \| 0.89 \| 6.0 \| 0.7 \| A1 \| / \| / \| \| 275 \| B275 \| 146.9 \| 45 \| 6.61 \| 1.52 \| 0.69 \| N/A \| 1.0 \| B1 \| / \| / \| \| 276 \| B276 \| 82.1 \| 45 \| 3.70 \| 1.73 \| 0.99 \| 6.1 \| 1.0 \| A1 \| / \| / \| \| 277 \| B277 \| 27.5 \| 45 \| 1.24 \| 1.73 \| 0.89 \| 5.0 \| 0.8 \| B1 \| / \| / \| \| 278 \| B278 \| 25.3 \| 45 \| 1.14 \| 1.83 \| 1.31 \| 5.1 \| 1.0 \| B1 \| / \| / \| \| 279 \| B279 \| 15.0 \| 45 \| 0.68 \| 1.84 \| 1.00 \| N/A \| 1.2 \| B1 \| / \| / \| \| 280 \| B280 \| 21.2 \| 45 \| 0.95 \| 2.00 \| 1.29 \| 6.9 \| 0.9 \| A1 \| / \| / \| \| 281 \| B281 \| 45.5 \| 45 \| 2.05 \| 1.74 \| 0.98 \| 6.8 \| 0.9 \| A1 \| / \| / \| \| 282 \| B282 \| 68.3 \| 45 \| 3.07 \| 1.99 \| 2.03 \| 2.9 \| 0.9 \| C \| / \| / \| \| 283 \| B283 \| 75.5 \| 45 \| 3.40 \| 1.95 \| 1.63 \| N/A \| 1.6 \| C \| / \| / \| \| 284 \| B284 \| 64.0 \| 45 \| 2.88 \| 1.99 \| 1.71 \| 4.4 \| 1.4 \| C \| / \| / \| \| 285 \| B285 \| 70.7 \| 45 \| 3.18 \| 1.90 \| 1.54 \| 4.1 \| 1.0 \| C \| / \| / \| \| 286 \| B286 \| 73.4 \| 45 \| 3.30 \| 1.96 \| 1.59 \| 3.0 \| 1.0 \| C \| / \| / \| \| 287 \| B287 \| 72.2 \| 45 \| 3.25 \| 1.81 \| 1.17 \| N/A \| 1.3 \| C \| / \| / \| \| 288 \| B288 \| 89.4 \| 45 \| 4.02 \| 1.84 \| 1.33 \| 3.0 \| 0.7 \| C \| / \| / \| \| 289 \| B289 \| 53.3 \| 45 \| 2.40 \| 1.93 \| 1.74 \| 2.9 \| 1.0 \| C \| / \| / \| \| 290 \| B290 \| 77.3 \| 45 \| 3.48 \| 1.53 \| 0.68 \| 6.4 \| 1.0 \| A1 \| / \| / \| \| 291 \| B291 \| 15.7 \| 45 \| 0.71 \| 1.85 \| 1.39 \| 6.0 \| 1.0 \| A1 \| / \| / \| \| 292 \| B292 \| 24.9 \| 45 \| 1.12 \| 1.93 \| 1.24 \| 4.3 \| 1.1 \| C \| / \| / \| \| 293 \| B293 \| 20.5 \| 45 \| 0.92 \| 1.87 \| 1.03 \| 6.0 \| 1.3 \| A1 \| / \| / \| \| 294 \| B294 \| 210.0 \| 45 \| 9.45 \| 1.54 \| 0.76 \| 2.6 \| 1.1 \| C \| / \| / \| \| 295 \| B295 \| 23.1 \| 45 \| 1.04 \| 1.95 \| 1.49 \| 6.8 \| 0.7 \| A1 \| / \| / \| \| 296 \| B296 \| 7.7 \| 45 \| 0.35 \| 1.86 \| 0.80 \| 5.3 \| 0.8 \| B1 \| / \| / \| \| 297 \| B297 \| 35.8 \| 45 \| 1.61 \| 1.89 \| 1.18 \| 4.0 \| 1.0 \| C \| / \| / \| \| 298 \| B298 \| 22.2 \| 45 \| 1.00 \| 1.83 \| 1.14 \| 7.2 \| 1.1 \| A \| / \| / \| \| 299 \| B299 \| 8.6 \| 45 \| 0.39 \| 1.75 \| 0.90 \| 7.3 \| 0.8 \| A \| / \| / \| \| 300 \| B300 \| 3.7 \| 45 \| 0.17 \| 1.50 \| 0.90 \| 6.1 \| 0.8 \| A1 \| / \| / \| \| 301 \| B301 \| 41.2 \| 45 \| 1.85 \| 1.89 \| 1.35 \| 2.2 \| 1.6 \| C \| / \| / \| \| 302 \| B302 \| 48.3 \| 45 \| 2.17 \| 1.96 \| 1.60 \| 2.4 \| 1.0 \| C \| / \| / \| \| 303 \| B303 \| 86.4 \| 45 \| 3.89 \| 1.51 \| 0.61 \| 6.3 \| 1.0 \| A1 \| / \| / \| \| 304 \| B304 \| 8.4 \| 45 \| 0.38 \| 1.81 \| 0.45 \| 6.9 \| 0.7 \| A1 \| / \| / \| \| 305 \| B305 \| 50.9 \| 45 \| 2.29 \| 1.70 \| 0.83 \| 6.4 \| 0.9 \| A1 \| / \| / \| \| 306 \| B306 \| 12.0 \| 45 \| 0.54 \| 1.88 \| 0.78 \| N/A \| 1.1 \| A1 \| / \| / \| \| 307 \| B307 \| 14.1 \| 45 \| 0.63 \| 1.89 \| 1.32 \| 4.0 \| 1.0 \| C \| / \| / \| \| 308 \| B308 \| 29.8 \| 45 \| 1.34 \| 1.85 \| 1.29 \| 3.8 \| 0.7 \| C \| / \| / \| \| 309 \| B309 \| 38.6 \| 45 \| 1.74 \| 1.72 \| 0.94 \| 6.2 \| 1.5 \| A1 \| / \| / \| \| 310 \| B310 \| 24.4 \| 45 \| 1.10 \| 1.95 \| 1.66 \| 3.0 \| 0.9 \| C \| / \| / \| \| 311 \| B312 \| 51.2 \| 45 \| 2.30 \| 1.61 \| 0.70 \| 3.5 \| 0.6 \| C \| / \| / \| \| 312 \| B313 \| 24.8 \| 45 \| 1.12 \| 1.94 \| 1.64 \| 5.5 \| 1.2 \| B1 \| / \| / \| \| 313 \| B314 \| 63.7 \| 45 \| 2.87 \| 1.71 \| 0.99 \| 5.6 \| 0.6 \| B1 \| / \| / \| \| 314 \| B315 \| 45.4 \| 45 \| 2.04 \| 1.69 \| 0.90 \| 5.0 \| 1.0 \| B1 \| / \| / \| \| 315 \| B316 \| 29.9 \| 45 \| 1.35 \| 1.71 \| 0.92 \| 5.6 \| 1.1 \| B1 \| / \| / \| \| 316 \| B317 \| 41.6 \| 45 \| 1.87 \| 1.90 \| 1.42 \| 4.6 \| 1.1 \| C \| / \| / \| \| 317 \| B318 \| 46.3 \| 45 \| 2.08 \| 1.58 \| 0.73 \| 4.5 \| 0.9 \| C \| / \| / \| \| 318 \| B319 \| 27.5 \| 45 \| 1.24 \| 1.86 \| 1.36 \| 4.7 \| 0.8 \| C \| / \| / \| \| 319 \| B320 \| 68.4 \| 45 \| 3.08 \| 1.60 \| 0.78 \| 6.1 \| 0.8 \| A1 \| / \| / \| \| 320 \| B321 \| 20.8 \| 45 \| 0.94 \| 1.65 \| 0.74 \| 3.7 \| 0.5 \| C \| / \| / \| \| 321 \| B322 \| 19.4 \| 45 \| 0.87 \| 1.87 \| 1.42 \| 6.7 \| 0.8 \| A1 \| / \| / \| \| 322 \| B323 \| 28.0 \| 45 \| 1.26 \| 1.75 \| 1.05 \| 4.6 \| 0.8 \| C \| / \| / \| \| 323 \| B324 \| 30.7 \| 45 \| 1.38 \| 1.92 \| 1.65 \| 6.7 \| 1.1 \| A1 \| / \| / \| \| 324 \| B325 \| 40.1 \| 45 \| 1.81 \| 1.57 \| 0.75 \| 5.4 \| 0.7 \| B1 \| / \| / \| \| 325 \| B326 \| 83.7 \| 45 \| 3.77 \| 1.84 \| 1.37 \| 5.2 \| 1.0 \| B1 \| / \| / \| \| 326 \| B327 \| 53.1 \| 45 \| 2.39 \| 1.69 \| 0.79 \| 5.6 \| 1.0 \| B1 \| / \| / \| \| 327 \| B328 \| 101.4 \| 45 \| 4.56 \| 1.74 \| 1.03 \| 6.3 \| 1.3 \| A1 \| / \| / \| \| 328 \| B329 \| 70.8 \| 45 \| 3.19 \| 1.95 \| 1.92 \| 4.0 \| 1.0 \| C \| / \| / \| \| 329 \| B330 \| 105.6 \| 45 \| 4.75 \| 1.72 \| 0.99 \| 4.3 \| 0.8 \| C \| / \| / \| \| 330 \| B331 \| 64.6 \| 45 \| 2.91 \| 1.94 \| 1.75 \| 5.0 \| 1.2 \| B1 \| / \| / \| \| 331 \| B332 \| 42.7 \| 45 \| 1.92 \| 1.95 \| 1.79 \| 6.9 \| 1.0 \| A1 \| / \| / \| \| 332 \| B333 \| 44.1 \| 45 \| 1.99 \| 1.83 \| 1.25 \| 3.6 \| 1.0 \| C \| / \| / \| \| 333 \| B334 \| 15.2 \| 45 \| 0.68 \| 1.81 \| 0.92 \| 6.5 \| 1.1 \| A1 \| / \| / \| \| 334 \| B335 \| 80.5 \| 45 \| 3.62 \| 1.65 \| 0.90 \| 4.7 \| 0.7 \| C \| / \| / \| \| 335 \| B336 \| 25.1 \| 45 \| 1.13 \| 1.75 \| 0.91 \| 6.8 \| 1.1 \| A1 \| / \| / \| \| 336 \| B337 \| 71.1 \| 45 \| 3.20 \| 1.97 \| 1.88 \| 2.6 \| 0.8 \| C \| / \| / \| \| 337 \| B338 \| 54.9 \| 45 \| 2.47 \| 2.01 \| 2.01 \| 5.4 \| 0.9 \| B1 \| / \| / \| \| 338 \| B339 \| 11.3 \| 45 \| 0.51 \| 1.86 \| 1.53 \| 6.9 \| 1.3 \| A1 \| / \| / \| \| 339 \| B340 \| 62.9 \| 45 \| 2.83 \| 1.63 \| 0.75 \| 6.2 \| 0.9 \| A1 \| / \| / \| \| 340 \| B341 \| 23.5 \| 45 \| 1.06 \| 2.00 \| 1.86 \| 6.0 \| 0.9 \| A1 \| / \| / \| \| 341 \| B342 \| 18.2 \| 45 \| 0.82 \| 1.90 \| 1.50 \| 5.8 \| 1.2 \| B1 \| / \| / \| \| 342 \| B343 \| 17.7 \| 45 \| 0.79 \| 2.00 \| 1.17 \| 4.6 \| 1.1 \| C \| / \| / \| \| 343 \| B344 \| 11.3 \| 45 \| 0.51 \| 1.98 \| 1.20 \| 6.7 \| 0.9 \| A1 \| / \| / \| \| 344 \| B345 \| 8.2 \| 45 \| 0.37 \| 2.07 \| 1.30 \| 7.1 \| 1.3 \| A \| / \| / \| \| 345 \| B346 \| 23.2 \| 45 \| 1.04 \| 1.94 \| 0.67 \| 4.6 \| 0.8 \| C \| / \| / \| \| 346 \| B347 \| 4.0 \| 45 \| 0.18 \| 1.73 \| 0.22 \| 7.0 \| 0.9 \| A \| / \| / \| \| 347 \| B348 \| 14.3 \| 45 \| 0.64 \| 1.91 \| 1.68 \| 5.5 \| 1.1 \| B1 \| / \| / \| \| 348 \| B349 \| 22.0 \| 45 \| 0.99 \| 1.99 \| 1.45 \| 6.3 \| 1.0 \| A1 \| / \| / \| \| 349 \| B350 \| 4.6 \| 45 \| 0.21 \| 2.38 \| 0.81 \| 7.5 \| 1.0 \| A \| / \| / \| \| 350 \| B351 \| 27.4 \| 45 \| 1.23 \| 1.91 \| 1.71 \| 3.4 \| 0.8 \| C \| / \| / \| \| 351 \| B352 \| 28.4 \| 45 \| 1.28 \| 2.02 \| 2.11 \| 4.9 \| 0.8 \| C \| / \| / \| \| 352 \| B353 \| 14.4 \| 45 \| 0.65 \| 1.93 \| 1.30 \| 5.3 \| 0.8 \| B1 \| / \| / \| \| 353 \| B354 \| 48.3 \| 45 \| 2.17 \| 2.00 \| 1.83 \| 2.8 \| 1.3 \| C \| / \| / \| \| 354 \| B355 \| 21.6 \| 45 \| 0.97 \| 1.99 \| 1.80 \| 2.1 \| 2.5 \| C \| / \| / \| \| 355 \| B356 \| 6.4 \| 45 \| 0.29 \| 1.96 \| 0.68 \| 6.5 \| 1.4 \| A1 \| / \| / \| \| 356 \| B357 \| 25.7 \| 45 \| 1.15 \| 2.04 \| 1.93 \| 6.9 \| 1.0 \| A1 \| / \| / \| \| 357 \| B358 \| 11.9 \| 45 \| 0.54 \| 1.97 \| 1.28 \| 7.6 \| 1.0 \| A \| / \| / \| \| 358 \| B359 \| 24.2 \| 45 \| 1.09 \| 1.95 \| 1.89 \| 7.0 \| 0.9 \| A \| / \| / \| \| 359 \| B360 \| 15.5 \| 45 \| 0.70 \| 2.01 \| 1.23 \| 6.1 \| 1.1 \| A1 \| / \| / \| \| 360 \| B361 \| 19.0 \| 45 \| 0.85 \| 2.02 \| 1.70 \| 5.6 \| 1.1 \| B1 \| / \| / \| \| 361 \| B362 \| 37.4 \| 45 \| 1.68 \| 1.85 \| 1.57 \| 5.0 \| 0.8 \| B1 \| / \| / \| \| 362 \| B363 \| 56.5 \| 45 \| 2.54 \| 1.94 \| 1.97 \| 2.8 \| 0.8 \| C \| / \| / \| \| 363 \| B364 \| 57.2 \| 45 \| 2.57 \| 1.71 \| 0.99 \| 2.7 \| 0.0 \| C \| / \| / \| \| 364 \| B365 \| 11.0 \| 45 \| 0.50 \| 1.93 \| 1.52 \| 2.5 \| 0.0 \| C \| / \| / \| \| 365 \| B366 \| 32.3 \| 45 \| 1.45 \| 1.87 \| 0.82 \| 3.1 \| 0.7 \| C \| / \| / \| \| 366 \| B367 \| 7.6 \| 45 \| 0.34 \| 1.88 \| 0.72 \| 1.2 \| 0.0 \| C \| / \| / \| \| 367 \| B368 \| 11.3 \| 45 \| 0.51 \| 2.02 \| 0.93 \| 6.2 \| 1.4 \| A1 \| / \| / \| \| 368 \| B369 \| 17.9 \| 45 \| 0.81 \| 1.93 \| 1.69 \| 4.4 \| 0.7 \| C \| / \| / \| \| 369 \| B370 \| 21.2 \| 45 \| 0.95 \| 1.97 \| 1.67 \| 3.6 \| 0.0 \| C \| / \| / \| \| 370 \| B371 \| 28.9 \| 45 \| 1.30 \| 2.03 \| 1.87 \| 3.2 \| 0.7 \| C \| / \| / \| \| 371 \| B372 \| 16.4 \| 45 \| 0.74 \| 1.92 \| 1.67 \| 5.1 \| 0.9 \| B1 \| / \| / \| \| 372 \| B373 \| 24.4 \| 45 \| 1.10 \| 1.90 \| 1.72 \| 4.3 \| 0.7 \| C \| / \| / \| \| 373 \| B374 \| 25.9 \| 45 \| 1.17 \| 1.92 \| 1.67 \| 3.5 \| 1.0 \| C \| / \| / \| \| 374 \| B375 \| 9.1 \| 45 \| 0.41 \| 1.84 \| 1.35 \| 6.2 \| 1.4 \| A1 \| / \| / \| \| 375 \| B376 \| 21.2 \| 45 \| 0.95 \| 1.92 \| 1.58 \| 3.4 \| 0.7 \| C \| / \| / \| \| 376 \| B377 \| 40.9 \| 45 \| 1.84 \| 1.93 \| 1.86 \| 2.6 \| 0.2 \| C \| / \| / \| \| 377 \| B378 \| 42.7 \| 45 \| 1.92 \| 1.93 \| 1.67 \| 3.9 \| 1.1 \| C \| / \| / \| \| 378 \| B379 \| 27.2 \| 45 \| 1.22 \| 1.88 \| 1.81 \| 4.6 \| 0.9 \| C \| / \| / \| \| 379 \| B380 \| 16.2 \| 45 \| 0.73 \| 1.81 \| 1.49 \| 2.8 \| 0.7 \| C \| / \| / \| \| 380 \| B381 \| 24.5 \| 45 \| 1.10 \| 1.95 \| 1.41 \| 3.8 \| 0.8 \| C \| / \| / \| \| 381 \| B382 \| 31.3 \| 45 \| 1.41 \| 1.92 \| 1.71 \| 5.1 \| 0.8 \| B1 \| / \| / \| \| 382 \| B383 \| 23.7 \| 45 \| 1.07 \| 1.98 \| 1.68 \| 3.8 \| 0.6 \| C \| / \| / \| \| 383 \| B384 \| 38.1 \| 45 \| 1.71 \| 1.94 \| 1.09 \| 5.3 \| 1.3 \| B1 \| / \| / \| \| 384 \| B385 \| 32.8 \| 45 \| 1.48 \| 1.95 \| 1.71 \| 5.6 \| 1.0 \| B1 \| / \| / \| \| 385 \| B386 \| 29.4 \| 45 \| 1.32 \| 1.96 \| 1.59 \| 6.3 \| 1.2 \| A1 \| / \| / \| \| 386 \| B387 \| 56.1 \| 45 \| 2.52 \| 1.98 \| 1.07 \| 5.6 \| 1.0 \| B1 \| / \| / \| \| 387 \| B388 \| 29.8 \| 45 \| 1.34 \| 1.77 \| 1.03 \| 5.3 \| 1.0 \| B1 \| / \| / \| \| 388 \| B389 \| 26.2 \| 45 \| 1.18 \| 1.88 \| 1.42 \| 2.9 \| 0.0 \| C \| / \| / \| \| 389 \| B390 \| 27.3 \| 45 \| 1.23 \| 1.96 \| 1.74 \| 6.3 \| 0.9 \| A1 \| / \| / \| \| 390 \| B391 \| 24.6 \| 45 \| 1.11 \| 1.89 \| 0.46 \| 6.7 \| 1.0 \| A1 \| / \| / \| \| 391 \| B392 \| 8.7 \| 45 \| 0.39 \| 1.80 \| 1.19 \| 5.3 \| 0.9 \| B1 \| / \| / \| \| 392 \| B393 \| 23.7 \| 45 \| 1.07 \| 1.91 \| 1.52 \| 6.7 \| 1.3 \| A1 \| / \| / \| \| 393 \| B394 \| 18.1 \| 45 \| 0.81 \| 1.87 \| 1.30 \| 5.4 \| 1.1 \| B1 \| / \| / \| \| 394 \| B395 \| 23.2 \| 45 \| 1.05 \| 1.93 \| 1.51 \| 6.1 \| 1.4 \| A1 \| / \| / \| \| 395 \| B396 \| 24.3 \| 45 \| 1.09 \| 1.92 \| 1.68 \| 5.3 \| 0.9 \| B1 \| / \| / \| \| 396 \| B397 \| 28.0 \| 45 \| 1.26 \| 1.95 \| 1.68 \| 4.3 \| 0.7 \| C \| / \| / \| \| 397 \| B398 \| 18.2 \| 45 \| 0.82 \| 1.88 \| 1.30 \| 6.3 \| 0.7 \| A1 \| / \| / \| \| 398 \| B399 \| 15.2 \| 45 \| 0.69 \| 1.91 \| 1.45 \| 5.9 \| 1.0 \| B1 \| / \| / \| \| 399 \| B400 \| 15.1 \| 45 \| 0.68 \| 1.94 \| 1.19 \| 5.4 \| 0.0 \| B1 \| / \| / \| \| 400 \| B401 \| 5.8 \| 45 \| 0.26 \| 1.85 \| 0.82 \| 5.9 \| 0.7 \| B1 \| / \| / \| \| 401 \| B402 \| 22.2 \| 45 \| 1.00 \| 1.90 \| 1.31 \| 5.0 \| 0.7 \| B1 \| / \| / \| \| 402 \| B403 \| 10.0 \| 45 \| 0.45 \| 1.57 \| 0.67 \| 3.7 \| 0.9 \| C \| / \| / \| \| 403 \| B404 \| 9.7 \| 45 \| 0.43 \| 1.77 \| 0.69 \| 6.3 \| 0.9 \| A1 \| / \| / \| \| 404 \| B405 \| 3.0 \| 45 \| 0.13 \| 1.76 \| 0.47 \| 2.8 \| 0.0 \| C \| / \| / \| \| 405 \| B406 \| 2.9 \| 45 \| 0.13 \| 1.94 \| 0.27 \| 7.1 \| 1.1 \| A \| / \| / \| \| 406 \| B407 \| 10.6 \| 45 \| 0.48 \| 1.92 \| 1.15 \| 6.1 \| 0.9 \| A1 \| / \| / \| \| 407 \| B408 \| 17.3 \| 45 \| 0.78 \| 1.92 \| 1.15 \| 5.6 \| 1.2 \| B1 \| / \| / \| \| 408 \| B409 \| 24.3 \| 45 \| 1.09 \| 1.93 \| 0.55 \| 6.6 \| 1.0 \| A1 \| / \| / \| \| 409 \| B410 \| 27.0 \| 45 \| 1.21 \| 1.94 \| 1.31 \| 5.7 \| 0.8 \| B1 \| / \| / \| \| 410 \| B411 \| 13.3 \| 45 \| 0.60 \| 1.78 \| 0.53 \| 7.0 \| 0.7 \| A \| / \| / \| \| 411 \| B412 \| 41.8 \| 45 \| 1.88 \| 1.79 \| 0.62 \| 6.5 \| 0.9 \| A1 \| / \| / \| \| 412 \| B413 \| 17.7 \| 45 \| 0.80 \| 1.81 \| 0.92 \| 6.6 \| 0.9 \| A1 \| / \| / \| \| 413 \| B414 \| 6.7 \| 45 \| 0.30 \| 2.17 \| 0.58 \| 6.6 \| 0.8 \| A1 \| / \| / \| \| 414 \| B415 \| 5.8 \| 45 \| 0.26 \| 2.32 \| 0.81 \| 7.3 \| 0.6 \| A \| / \| / \| \| 415 \| B416 \| 15.5 \| 45 \| 0.70 \| 2.04 \| 0.85 \| 6.3 \| 0.7 \| A1 \| / \| / \| \| 416 \| B417 \| 15.2 \| 45 \| 0.68 \| 1.85 \| 0.32 \| 6.8 \| 0.8 \| A1 \| / \| / \| \| 417 \| B418 \| 13.3 \| 45 \| 0.60 \| 2.01 \| 1.16 \| 6.2 \| 1.0 \| A1 \| / \| / \| \| 418 \| B419 \| 14.0 \| 45 \| 0.63 \| 2.01 \| 1.43 \| 6.0 \| 1.2 \| A1 \| / \| / \| \| 419 \| B420 \| 7.4 \| 45 \| 0.33 \| 2.14 \| 0.85 \| 6.8 \| 0.7 \| A1 \| / \| / \| \| 420 \| B421 \| 4.3 \| 45 \| 0.20 \| 1.86 \| 0.72 \| 6.9 \| 0.8 \| A1 \| / \| / \| \| 421 \| B422 \| 26.7 \| 45 \| 1.20 \| 1.88 \| 1.27 \| 5.5 \| 0.7 \| B1 \| / \| / \| \| 422 \| B423 \| 18.9 \| 45 \| 0.85 \| 1.67 \| 0.80 \| 4.1 \| 1.0 \| C \| / \| / \| \| 423 \| B424 \| 5.6 \| 45 \| 0.25 \| 1.55 \| 0.68 \| 6.7 \| 0.6 \| A1 \| / \| / \| \| 424 \| B425 \| 3.8 \| 45 \| 0.17 \| 2.31 \| 0.60 \| 2.9 \| 0.0 \| C \| / \| / \| \| 425 \| B426 \| 11.4 \| 45 \| 0.51 \| 2.03 \| 1.00 \| 5.9 \| 0.9 \| B1 \| / \| / \| \| 426 \| B427 \| 14.6 \| 45 \| 0.66 \| 2.00 \| 1.33 \| 6.8 \| 1.0 \| A1 \| / \| / \| \| 427 \| B428 \| 14.1 \| 45 \| 0.63 \| 1.82 \| 0.72 \| 6.1 \| 0.7 \| A1 \| / \| / \| \| 428 \| B429 \| 11.0 \| 45 \| 0.49 \| 2.03 \| 0.22 \| 6.0 \| 0.8 \| A1 \| / \| / \| \| 429 \| B430 \| 5.3 \| 45 \| 0.24 \| 1.94 \| 0.33 \| 6.9 \| 0.7 \| A1 \| / \| / \| \| 430 \| B431 \| 3.0 \| 45 \| 0.14 \| 2.44 \| 0.39 \| 7.3 \| 1.0 \| A \| / \| / \| \| 431 \| B432 \| 3.0 \| 45 \| 0.13 \| 1.90 \| 0.48 \| 6.4 \| 0.3 \| A1 \| / \| / \| \| 432 \| B433 \| 5.7 \| 45 \| 0.26 \| 2.00 \| 0.59 \| 5.8 \| 0.7 \| B1 \| / \| / \| \| 433 \| B434 \| 2.2 \| 45 \| 0.10 \| 2.59 \| 0.61 \| 6.8 \| 0.7 \| A1 \| / \| / \| \| 434 \| B435 \| 1.3 \| 45 \| 0.06 \| 1.67 \| 0.40 \| 1.0 \| 0.0 \| C \| / \| / \| \| 435 \| B436 \| 14.0 \| 45 \| 0.63 \| 1.65 \| 0.65 \| 5.6 \| 0.9 \| B1 \| / \| / \| \| 436 \| B437 \| 7.0 \| 45 \| 0.31 \| 1.94 \| 1.62 \| 7.0 \| 1.0 \| A \| / \| / \| \| 437 \| B438 \| 2.6 \| 45 \| 0.12 \| 1.84 \| 0.75 \| 7.0 \| 0.9 \| A \| / \| / \| \| 438 \| B439 \| 4.6 \| 45 \| 0.21 \| 1.81 \| 0.73 \| 6.5 \| 1.2 \| A1 \| / \| / \| \| 439 \| B440 \| 16.5 \| 45 \| 0.74 \| 2.04 \| 1.69 \| 6.7 \| 1.2 \| A1 \| / \| / \| \| 440 \| B441 \| 6.2 \| 45 \| 0.28 \| 1.79 \| 1.00 \| 6.9 \| 0.9 \| A1 \| / \| / \| \| 441 \| B442 \| 41.8 \| 45 \| 1.88 \| 1.52 \| 0.60 \| 7.4 \| 0.8 \| A \| / \| / \| \| 442 \| B443 \| 2.8 \| 45 \| 0.13 \| 2.21 \| 0.56 \| 7.0 \| 0.5 \| A \| / \| / \| \| 443 \| B444 \| 33.9 \| 45 \| 1.53 \| 1.59 \| 0.73 \| 5.4 \| 0.8 \| B1 \| / \| / \| \| 444 \| B445 \| 31.8 \| 45 \| 1.43 \| 1.75 \| 1.16 \| 5.5 \| 1.0 \| B1 \| / \| / \| \| 445 \| B446 \| 17.6 \| 45 \| 0.79 \| 1.80 \| 0.78 \| 7.0 \| 1.2 \| A \| / \| / \| \| 446 \| B447 \| 9.4 \| 45 \| 0.42 \| 2.15 \| 0.84 \| 7.0 \| 0.9 \| A \| / \| / \| \| 447 \| B448 \| 3.4 \| 45 \| 0.15 \| 2.22 \| 0.77 \| 6.2 \| 0.7 \| A1 \| / \| / \| \| 448 \| B449 \| 28.9 \| 45 \| 1.30 \| 1.86 \| 1.52 \| 5.6 \| 0.9 \| B1 \| / \| / \| \| 449 \| B450 \| 7.2 \| 45 \| 0.32 \| 1.56 \| 0.63 \| 6.0 \| 0.7 \| A1 \| / \| / \| \| 450 \| B451 \| 27.1 \| 45 \| 1.22 \| 1.81 \| 1.12 \| 4.3 \| 1.0 \| C \| / \| / \| \| 451 \| B452 \| 26.5 \| 45 \| 1.19 \| 1.97 \| 1.27 \| 4.8 \| 1.1 \| C \| / \| / \| \| 452 \| B453 \| 104.9 \| 45 \| 4.72 \| 1.94 \| 1.76 \| 2.3 \| 5.0 \| C \| / \| / \| \| 453 \| B454 \| 71.8 \| 45 \| 3.23 \| 1.96 \| 1.93 \| 3.5 \| 1.2 \| C \| / \| / \| \| 454 \| B455 \| 56.5 \| 45 \| 2.54 \| 1.93 \| 1.67 \| 3.3 \| 1.0 \| C \| / \| / \| \| 455 \| B456 \| 71.1 \| 45 \| 3.20 \| 1.91 \| 1.71 \| 3.3 \| 1.1 \| C \| / \| / \| \| 456 \| B457 \| 43.6 \| 45 \| 1.96 \| 1.97 \| 1.73 \| 3.7 \| 0.7 \| C \| / \| / \| \| 457 \| B458 \| 67.5 \| 45 \| 3.04 \| 1.95 \| 1.80 \| 4.0 \| 0.8 \| C \| / \| / \| \| 458 \| B459 \| 33.2 \| 45 \| 1.49 \| 1.93 \| 1.77 \| 4.9 \| 0.0 \| C \| / \| / \| \| 459 \| B460 \| 49.5 \| 45 \| 2.23 \| 1.95 \| 1.81 \| 5.4 \| 1.0 \| B1 \| / \| / \| \| 460 \| B461 \| 27.7 \| 45 \| 1.25 \| 1.88 \| 1.63 \| 5.1 \| 0.9 \| B1 \| / \| / \| \| 461 \| B462 \| 36.4 \| 45 \| 1.64 \| 1.93 \| 1.84 \| 3.0 \| 0.9 \| C \| / \| / \| \| 462 \| B463 \| 77.3 \| 45 \| 3.48 \| 1.94 \| 1.87 \| 5.5 \| 0.9 \| B1 \| / \| / \| \| 463 \| B464 \| 30.8 \| 45 \| 1.39 \| 1.95 \| 1.04 \| 3.6 \| 0.7 \| C \| / \| / \| \| 464 \| B465 \| 40.6 \| 45 \| 1.83 \| 1.94 \| 1.80 \| 4.0 \| 0.9 \| C \| / \| / \| \| 465 \| B466 \| 72.6 \| 45 \| 3.27 \| 1.94 \| 1.80 \| 2.5 \| 0.4 \| C \| / \| / \| \| 466 \| B467 \| 32.3 \| 45 \| 1.46 \| 1.99 \| 1.80 \| 3.6 \| 0.6 \| C \| / \| / \| \| 467 \| B468 \| 27.0 \| 45 \| 1.22 \| 1.96 \| 1.45 \| 3.8 \| 1.4 \| C \| / \| / \| \| 468 \| B469 \| 52.7 \| 45 \| 2.37 \| 1.97 \| 0.93 \| 5.9 \| 1.1 \| B1 \| / \| / \| \| 469 \| B470 \| 47.7 \| 45 \| 2.15 \| 1.91 \| 1.79 \| 4.9 \| 0.9 \| C \| / \| / \| \| 470 \| B471 \| 40.5 \| 45 \| 1.82 \| 1.91 \| 1.74 \| 3.2 \| 0.7 \| C \| / \| / \| \| 471 \| B472 \| 32.2 \| 45 \| 1.45 \| 1.97 \| 1.76 \| 5.7 \| 1.1 \| C \| / \| / \| \| 472 \| B473 \| 19.0 \| 45 \| 0.86 \| 2.00 \| 0.25 \| 5.2 \| 1.5 \| B1 \| / \| / \| \| 473 \| B474 \| 71.3 \| 45 \| 3.21 \| 1.96 \| 1.92 \| 2.7 \| 0.4 \| C \| / \| / \| \| 474 \| B475 \| 26.7 \| 45 \| 1.20 \| 1.88 \| 1.76 \| 6.0 \| 1.2 \| A1 \| / \| / \| \| 475 \| B476 \| 14.3 \| 45 \| 0.64 \| 1.94 \| 0.85 \| 6.0 \| 1.1 \| A1 \| / \| / \| \| 476 \| B477 \| 54.3 \| 45 \| 2.44 \| 1.84 \| 1.26 \| 3.5 \| 0.8 \| C \| / \| / \| \| 477 \| B478 \| 32.9 \| 45 \| 1.48 \| 1.97 \| 1.64 \| 5.5 \| 1.0 \| B1 \| / \| / \| \| 478 \| B479 \| 66.3 \| 45 \| 2.98 \| 1.97 \| 1.54 \| 3.1 \| 0.5 \| C \| / \| / \| \| 479 \| B480 \| 18.3 \| 45 \| 0.83 \| 2.07 \| 1.55 \| 4.5 \| 0.0 \| C \| / \| / \| \| 480 \| B481 \| 49.1 \| 45 \| 2.21 \| 1.92 \| 1.83 \| 2.5 \| 0.1 \| C \| / \| / \| \| 481 \| B482 \| 83.6 \| 45 \| 3.76 \| 2.00 \| 2.06 \| 2.3 \| 0.1 \| C \| / \| / \| \| 482 \| B483 \| 107.8 \| 45 \| 4.85 \| 1.92 \| 1.83 \| 3.3 \| 1.1 \| C \| / \| / \| \| 483 \| B484 \| 67.6 \| 45 \| 3.04 \| 1.95 \| 1.99 \| 4.4 \| 1.1 \| C \| / \| / \| \| 484 \| B485 \| 82.7 \| 45 \| 3.72 \| 1.97 \| 1.87 \| 3.0 \| 0.7 \| C \| / \| / \| \| 485 \| B486 \| 41.6 \| 45 \| 1.87 \| 1.89 \| 0.16 \| 3.4 \| 0.7 \| C \| / \| / \| \| 486 \| B487 \| 71.9 \| 45 \| 3.24 \| 1.97 \| 1.94 \| 3.0 \| 0.4 \| C \| / \| / \| \| 487 \| B488 \| 85.2 \| 45 \| 3.83 \| 1.93 \| 1.84 \| 2.4 \| 0.4 \| C \| / \| / \| \| 488 \| B489 \| 147.2 \| 45 \| 6.63 \| 1.93 \| 1.68 \| 2.6 \| 0.5 \| C \| / \| / \| \| 489 \| B490 \| 124.0 \| 45 \| 5.58 \| 1.91 \| 1.98 \| 2.7 \| 0.7 \| C \| / \| / \| \| 490 \| B491 \| 64.1 \| 45 \| 2.88 \| 1.96 \| 1.61 \| 3.1 \| 0.6 \| C \| / \| / \| \| 491 \| B492 \| 55.4 \| 45 \| 2.49 \| 1.92 \| 1.45 \| 2.9 \| 0.4 \| C \| / \| / \| \| 492 \| B493 \| 63.2 \| 45 \| 2.85 \| 1.94 \| 1.89 \| 3.1 \| 0.6 \| C \| / \| / \| \| 493 \| B494 \| 46.5 \| 45 \| 2.09 \| 1.90 \| 1.20 \| 3.0 \| 0.4 \| C \| / \| / \| \| 494 \| B495 \| 73.8 \| 45 \| 3.32 \| 1.97 \| 1.84 \| 3.3 \| 0.8 \| C \| / \| / \| \| 495 \| B496 \| 51.6 \| 45 \| 2.32 \| 1.93 \| 1.51 \| 2.9 \| 0.5 \| C \| / \| / \| \| 496 \| B497 \| 75.1 \| 45 \| 3.38 \| 1.95 \| 1.96 \| 2.3 \| 0.4 \| C \| / \| / \| \| 497 \| B498 \| 33.7 \| 45 \| 1.52 \| 1.95 \| 1.58 \| 3.0 \| 0.8 \| C \| / \| / \| \| 498 \| B499 \| 53.1 \| 45 \| 2.39 \| 1.95 \| 1.84 \| 2.9 \| 0.6 \| C \| / \| / \| \| 499 \| B500 \| 67.9 \| 45 \| 3.06 \| 2.00 \| 1.88 \| 3.2 \| 0.8 \| C \| / \| / \| \| 500 \| B501 \| 7.4 \| 45 \| 0.33 \| 2.40 \| 1.66 \| 6.6 \| 0.8 \| A1 \| / \| / \| \| 501 \| B502 \| 5.1 \| 45 \| 0.23 \| 2.53 \| 1.70 \| 5.7 \| 0.8 \| B1 \| / \| / \| \| 502 \| B503 \| 20.1 \| 45 \| 0.91 \| 2.12 \| 1.95 \| 6.9 \| 1.3 \| A1 \| / \| / \| \| 503 \| B504 \| 12.9 \| 45 \| 0.58 \| 2.08 \| 1.28 \| 6.0 \| 0.9 \| A1 \| / \| / \| \| 504 \| B505 \| 15.0 \| 45 \| 0.68 \| 2.26 \| 1.58 \| 6.5 \| 1.0 \| A1 \| / \| / \| \| 505 \| B506 \| 7.3 \| 45 \| 0.33 \| 1.83 \| 1.60 \| 6.6 \| 0.9 \| A1 \| / \| / \| \| 506 \| B507 \| 27.1 \| 45 \| 1.22 \| 1.90 \| 1.73 \| 7.7 \| 1.2 \| A \| / \| / \| \| 507 \| B508 \| 4.4 \| 45 \| 0.20 \| 1.62 \| 0.96 \| 5.2 \| 1.0 \| B1 \| / \| / \| \| 508 \| B509 \| 14.0 \| 45 \| 0.63 \| 1.87 \| 1.57 \| 6.9 \| 1.1 \| A1 \| / \| / \| \| 509 \| B510 \| 6.2 \| 45 \| 0.28 \| 1.71 \| 1.47 \| 6.2 \| 0.8 \| A1 \| / \| / \| \| 510 \| B511 \| 21.4 \| 45 \| 0.96 \| 1.87 \| 1.57 \| 6.3 \| 1.1 \| A1 \| / \| / \| \| 511 \| B512 \| 13.6 \| 45 \| 0.61 \| 1.86 \| 1.82 \| 5.9 \| 0.7 \| B1 \| / \| / \| \| 512 \| B513 \| 7.6 \| 45 \| 0.34 \| 1.60 \| 1.21 \| 6.4 \| 0.8 \| A1 \| / \| / \| \| 513 \| B514 \| 16.4 \| 45 \| 0.74 \| 1.92 \| 1.71 \| 5.6 \| 1.0 \| B1 \| / \| / \| \| 514 \| B515 \| 6.6 \| 45 \| 0.30 \| 1.66 \| 1.46 \| 6.0 \| 0.7 \| A1 \| / \| / \| \| 515 \| B516 \| 15.7 \| 45 \| 0.70 \| 1.81 \| 1.66 \| N/A \| 0.0 \| A1 \| / \| / \| \| 516 \| B517 \| 17.1 \| 45 \| 0.77 \| 1.88 \| 1.33 \| 4.3 \| 1.1 \| C \| / \| / \| \| 517 \| B518 \| 22.3 \| 45 \| 1.00 \| 1.88 \| 1.54 \| 6.6 \| 0.7 \| A1 \| / \| / \| \| 518 \| B519 \| 10.9 \| 45 \| 0.49 \| 1.62 \| 0.94 \| 6.1 \| 0.7 \| A1 \| / \| / \| \| 519 \| B520 \| 4.9 \| 45 \| 0.22 \| 1.70 \| 1.11 \| 6.8 \| 0.8 \| A1 \| / \| / \| \| 520 \| B521 \| 39.1 \| 45 \| 1.76 \| 1.60 \| 0.68 \| 6.5 \| 0.7 \| A1 \| / \| / \| \| 521 \| B522 \| 6.6 \| 45 \| 0.30 \| 1.78 \| 0.42 \| 6.6 \| 1.0 \| A1 \| / \| / \| \| 522 \| B523 \| 39.6 \| 45 \| 1.78 \| 1.78 \| 1.15 \| 5.7 \| 1.0 \| B1 \| / \| / \| \| 523 \| B524 \| 25.1 \| 45 \| 1.13 \| 1.78 \| 1.30 \| 6.6 \| 0.9 \| A1 \| / \| / \| \| 524 \| B525 \| 11.1 \| 45 \| 0.50 \| 1.93 \| 1.53 \| 4.8 \| 0.9 \| C \| / \| / \| \| 525 \| B526 \| 127.0 \| 45 \| 5.71 \| 1.60 \| 0.77 \| 5.1 \| 0.7 \| B1 \| / \| / \| \| 526 \| B527 \| 12.5 \| 45 \| 0.56 \| 2.02 \| 1.89 \| 5.8 \| 1.1 \| B1 \| / \| / \| \| 527 \| B528 \| 32.7 \| 45 \| 1.47 \| 1.64 \| 0.78 \| 5.5 \| 0.8 \| B1 \| / \| / \| \| 528 \| B529 \| 19.1 \| 45 \| 0.86 \| 1.86 \| 1.70 \| 6.5 \| 0.8 \| A1 \| / \| / \| \| 529 \| B530 \| 9.7 \| 45 \| 0.44 \| 1.80 \| 1.15 \| 6.8 \| 1.0 \| A1 \| / \| / \| \| 530 \| B531 \| 10.7 \| 45 \| 0.48 \| 1.65 \| 0.83 \| 7.2 \| 1.5 \| A \| / \| / \| \| 531 \| B532 \| 15.9 \| 45 \| 0.72 \| 1.68 \| 0.82 \| 6.5 \| 0.9 \| A1 \| / \| / \| \| 532 \| B533 \| 5.4 \| 45 \| 0.24 \| 1.66 \| 0.80 \| 7.0 \| 1.0 \| A \| / \| / \| \| 533 \| B534 \| 7.3 \| 45 \| 0.33 \| 1.82 \| 1.13 \| 6.9 \| 0.9 \| A1 \| / \| / \| \| 534 \| B535 \| 6.0 \| 45 \| 0.27 \| 1.61 \| 0.87 \| 7.1 \| 0.9 \| A \| / \| / \| \| 535 \| B536 \| 18.6 \| 45 \| 0.84 \| 1.87 \| 1.52 \| 6.9 \| 0.9 \| A1 \| / \| / \| \| 536 \| B537 \| 10.7 \| 45 \| 0.48 \| 1.68 \| 0.81 \| 3.6 \| 1.8 \| C \| / \| / \| \| 537 \| B538 \| 3.7 \| 45 \| 0.17 \| 1.69 \| 0.66 \| 6.7 \| 0.8 \| A1 \| / \| / \| \| 538 \| B539 \| 5.2 \| 45 \| 0.23 \| 1.70 \| 0.75 \| 7.4 \| 1.1 \| A \| / \| / \| \| 539 \| B540 \| 3.6 \| 45 \| 0.16 \| 1.71 \| 0.64 \| 7.3 \| 0.9 \| A \| / \| / \| \| 540 \| B541 \| 11.2 \| 45 \| 0.51 \| 1.81 \| 0.53 \| 6.9 \| 0.8 \| A1 \| / \| / \| \| 541 \| B542 \| 5.0 \| 45 \| 0.23 \| 1.45 \| 0.64 \| 5.4 \| 0.7 \| B1 \| / \| / \| \| 542 \| B543 \| 14.8 \| 45 \| 0.67 \| 1.79 \| 1.03 \| N/A \| 0.0 \| B1 \| / \| / \| \| 543 \| B544 \| 7.7 \| 45 \| 0.34 \| 1.73 \| 0.84 \| 7.2 \| 1.1 \| A \| / \| / \| \| 544 \| B545 \| 15.7 \| 45 \| 0.70 \| 1.91 \| 1.44 \| N/A \| 0.0 \| B1 \| / \| / \| \| 545 \| B546 \| 6.6 \| 45 \| 0.30 \| 1.74 \| 0.88 \| 6.8 \| 0.9 \| A1 \| / \| / \| \| 546 \| B547 \| 16.1 \| 45 \| 0.72 \| 1.58 \| 0.74 \| 6.5 \| 0.7 \| A1 \| / \| / \| \| 547 \| B548 \| 11.3 \| 45 \| 0.51 \| 1.87 \| 1.34 \| 6.6 \| 0.8 \| A1 \| / \| / \| \| 548 \| B549 \| 5.5 \| 45 \| 0.25 \| 1.81 \| 0.98 \| 6.1 \| 0.9 \| A1 \| / \| / \| \| 549 \| B550 \| 10.9 \| 45 \| 0.49 \| 1.89 \| 1.32 \| 6.4 \| 0.9 \| A1 \| / \| / \| \| \| --- \| --- \| --- \| --- \| --- \| --- \| --- \| --- \| --- \| --- \| --- \| --- \| --- \| --- \| --- \| --- \| --- \| --- \| --- \| --- \| --- \| --- \| --- \| --- \| --- \| --- \| --- \| --- \| --- \| --- \| --- \| --- \| --- \| --- \| --- \| --- \| --- \| --- \| --- \| --- \| --- \| --- \| --- \| --- \| --- \| --- \| --- \| --- \| --- \| --- \| --- \| --- \| --- \| --- \| --- \| --- \| --- \| --- \| --- \| --- \| --- \| --- \| --- \| --- \| --- \| --- \| --- \| --- \| --- \| --- \| --- \| --- \| --- \| --- \| --- \| --- \| --- \| --- \| --- \| --- \| --- \| --- \| --- \| --- \| --- \| --- \| --- \| --- \| --- \| --- \| --- \| --- \| --- \| --- \| --- \| --- \| --- \| --- \| --- \| --- \| --- \| --- \| --- \| --- \| --- \| --- \| --- \| --- \| --- \| --- \| --- \| --- \| --- \| --- \| --- \| --- \| --- \| --- \| --- \| --- \| --- \| --- \| --- \| --- \| --- \| --- \| --- \| --- \| --- \| --- \| --- \| --- \| --- \| --- \| --- \| --- \| --- \| --- \| --- \| --- \| --- \| --- \| --- \| --- \| --- \| --- \| --- \| --- \| --- \| --- \| --- \| --- \| --- \| --- \| --- \| --- \| --- \| --- \| --- \| --- \| --- \| --- \| --- \| --- \| --- \| --- \| --- \| --- \| --- \| --- \| --- \| --- \| --- \| --- \| --- \| --- \| --- \| --- \| --- \| --- \| --- \| --- \| --- \| --- \| --- \| --- \| --- \| --- \| --- \| --- \| --- \| --- \| --- \| --- \| --- \| --- \| --- \| --- \| --- \| --- \| --- \| --- \| --- \| --- \| --- \| --- \| --- \| --- \| --- \| --- \| --- \| --- \| --- \| --- \| --- \| --- \| --- \| --- \| --- \| --- \| --- \| --- \| --- \| --- \| --- \| --- \| --- \| --- \| --- \| --- \| --- \| --- \| --- \| --- \| --- \| --- \| --- \| --- \| --- \| --- \| --- \| --- \| --- \| --- \| --- \| --- \| --- \| --- \| --- \| --- \| --- \| --- \| --- \| --- \| --- \| --- \| --- \| --- \| --- \| --- \| --- \| --- \| --- \| --- \| --- \| --- \| --- \| --- \| --- \| --- \| --- \| --- \| --- \| --- \| --- \| --- \| --- \| --- \| --- \| --- \| --- \| --- \| --- \| --- \| --- \| --- \| --- \| --- \| --- \| --- \| --- \| --- \| --- \| --- \| --- \| --- \| --- \| --- \| --- \| --- \| --- \| --- \| --- \| --- \| --- \| --- \| --- \| --- \| --- \| --- \| --- \| --- \| --- \| --- \| --- \| --- \| --- \| --- \| --- \| --- \| --- \| --- \| --- \| --- \| --- \| --- \| --- \| --- \| --- \| --- \| --- \| --- \| --- \| --- \| --- \| --- \| --- \| --- \| --- \| --- \| --- \| --- \| --- \| --- \| --- \| --- \| --- \| --- \| --- \| --- \| --- \| --- \| --- \| --- \| --- \| --- \| --- \| --- \| --- \| --- \| --- \| --- \| --- \| --- \| --- \| --- \| --- \| --- \| --- \| --- \| --- \| --- \| --- \| --- \| --- \| --- \| --- \| --- \| --- \| --- \| --- \| --- \| --- \| --- \| --- \| --- \| --- \| --- \| --- \| --- \| --- \| --- \| --- \| --- \| --- \| --- \| --- \| --- \| --- \| --- \| --- \| --- \| --- \| --- \| --- \| --- \| --- \| --- \| --- \| --- \| --- \| --- \| --- \| --- \| --- \| --- \| --- \| --- \| --- \| --- \| --- \| --- \| --- \| --- \| --- \| --- \| --- \| --- \| --- \| --- \| --- \| --- \| --- \| --- \| --- \| --- \| --- \| --- \| --- \| --- \| --- \| --- \| --- \| --- \| --- \| --- \| --- \| --- \| --- \| --- \| --- \| --- \| --- \| --- \| --- \| --- \| --- \| --- \| --- \| --- \| --- \| --- \| --- \| --- \| --- \| --- \| --- \| --- \| --- \| --- \| --- \| --- \| --- \| --- \| --- \| --- \| --- \| --- \| --- \| --- \| --- \| --- \| --- \| --- \| --- \| --- \| --- \| --- \| --- \| --- \| --- \| --- \| --- \| --- \| --- \| --- \| --- \| --- \| --- \| --- \| --- \| --- \| --- \| --- \| --- \| --- \| --- \| --- \| --- \| --- \| --- \| --- \| --- \| --- \| --- \| --- \| --- \| --- \| --- \| --- \| --- \| --- \| --- \| --- \| --- \| --- \| --- \| --- \| --- \| --- \| --- \| --- \| --- \| --- \| --- \| --- \| --- \| --- \| --- \| --- \| --- \| --- \| --- \| --- \| --- \| --- \| --- \| --- \| --- \| --- \| --- \| --- \| --- \| --- \| --- \| --- \| --- \| --- \| --- \| --- \| --- \| --- \| --- \| --- \| --- \| --- \| --- \| --- \| --- \| --- \| --- \| --- \| --- \| --- \| --- \| --- \| --- \| --- \| --- \| --- \| --- \| --- \| --- \| --- \| --- \| --- \| --- \| --- \| --- \| --- \| --- \| --- \| --- \| --- \| --- \| --- \| --- \| --- \| --- \| --- \| --- \| --- \| --- \| --- \| --- \| --- \| --- \| --- \| --- \| --- \| --- \| --- \| --- \| --- \| --- \| --- \| --- \| --- \| --- \| --- \| --- \| --- \| --- \| --- \| --- \| --- \| --- \| --- \| --- \| --- \| --- \| --- \| --- \| --- \| --- \| --- \| --- \| --- \| --- \| --- \| --- \| --- \| --- \| --- \| --- \| --- \| --- \| --- \| --- \| --- \| --- \| --- \| --- \| --- \| --- \| --- \| --- \| --- \| --- \| --- \| --- \| --- \| --- \| --- \| --- \| --- \| --- \| --- \| --- \| --- \| --- \| --- \| --- \| --- \| --- \| --- \| --- \| --- \| --- \| --- \| --- \| --- \| --- \| --- \| --- \| --- \| --- \| --- \| --- \| --- \| --- \| --- \| --- \| --- \| --- \| --- \| --- \| --- \| --- \| --- \| --- \| --- \| --- \| --- \| --- \| --- \| --- \| --- \| --- \| --- \| --- \| --- \| --- \| --- \| --- \| --- \| --- \| --- \| --- \| --- \| --- \| --- \| --- \| --- \| --- \| --- \| --- \| --- \| --- \| --- \| --- \| --- \| --- \| --- \| --- \| --- \| --- \| --- \| --- \| --- \| --- \| --- \| --- \| --- \| --- \| --- \| --- \| --- \| --- \| --- \| --- \| --- \| --- \| --- \| --- \| --- \| --- \| --- \| --- \| --- \| --- \| --- \| --- \| --- \| --- \| --- \| --- \| --- \| --- \| --- \| --- \| --- \| --- \| --- \| --- \| --- \| --- \| --- \| --- \| --- \| --- \| --- \| --- \| --- \| --- \| --- \| --- \| --- \| --- \| --- \| --- \| --- \| --- \| --- \| --- \| --- \| --- \| --- \| --- \| --- \| --- \| --- \| --- \| --- \| --- \| --- \| --- \| --- \| --- \| --- \| --- \| --- \| --- \| --- \| --- \| --- \| --- \| --- \| --- \| --- \| --- \| --- \| --- \| --- \| --- \| --- \| --- \| --- \| --- \| --- \| --- \| --- \| --- \| --- \| --- \| --- \| --- \| --- \| --- \| --- \| --- \| --- \| --- \| --- \| --- \| --- \| --- \| --- \| --- \| --- \| --- \| --- \| --- \| --- \| --- \| --- \| --- \| --- \| --- \| --- \| --- \| --- \| --- \| --- \| --- \| --- \| --- \| --- \| --- \| --- \| --- \| --- \| --- \| --- \| --- \| --- \| --- \| --- \| --- \| --- \| --- \| --- \| --- \| --- \| --- \| --- \| --- \| --- \| --- \| --- \| --- \| --- \| --- \| --- \| --- \| --- \| --- \| --- \| --- \| --- \| --- \| --- \| --- \| --- \| --- \| --- \| --- \| --- \| --- \| --- \| --- \| --- \| --- \| --- \| --- \| --- \| --- \| --- \| --- \| --- \| --- \| --- \| --- \| --- \| --- \| --- \| --- \| --- \| --- \| --- \| --- \| --- \| --- \| --- \| --- \| --- \| --- \| --- \| --- \| --- \| --- \| --- \| --- \| --- \| --- \| --- \| --- \| --- \| --- \| --- \| --- \| --- \| --- \| --- \| --- \| --- \| --- \| --- \| --- \| --- \| --- \| --- \| --- \| --- \| --- \| --- \| --- \| --- \| --- \| --- \| --- \| --- \| --- \| --- \| --- \| --- \| --- \| --- \| --- \| --- \| --- \| --- \| --- \| --- \| --- \| --- \| --- \| --- \| --- \| --- \| --- \| --- \| --- \| --- \| --- \| --- \| --- \| --- \| --- \| --- \| --- \| --- \| --- \| --- \| --- \| --- \| --- \| --- \| --- \| --- \| --- \| --- \| --- \| --- \| --- \| --- \| --- \| --- \| --- \| --- \| --- \| --- \| --- \| --- \| --- \| --- \| --- \| --- \| --- \| --- \| --- \| --- \| --- \| --- \| --- \| --- \| --- \| --- \| --- \| --- \| --- \| --- \| --- \| --- \| --- \| --- \| --- \| --- \| --- \| --- \| --- \| --- \| --- \| --- \| --- \| --- \| --- \| --- \| --- \| --- \| --- \| --- \| --- \| --- \| --- \| --- \| --- \| --- \| --- \| --- \| --- \| --- \| --- \| --- \| --- \| --- \| --- \| --- \| --- \| --- \| --- \| --- \| --- \| --- \| --- \| --- \| --- \| --- \| --- \| --- \| --- \| --- \| --- \| --- \| --- \| --- \| --- \| --- \| --- \| --- \| --- \| --- \| --- \| --- \| --- \| --- \| --- \| --- \| --- \| --- \| --- \| --- \| --- \| --- \| --- \| --- \| --- \| --- \| --- \| --- \| --- \| --- \| --- \| --- \| --- \| --- \| --- \| --- \| --- \| --- \| --- \| --- \| --- \| --- \| --- \| --- \| --- \| --- \| --- \| --- \| --- \| --- \| --- \| --- \| --- \| --- \| --- \| --- \| --- \| --- \| --- \| --- \| --- \| --- \| --- \| --- \| --- \| --- \| --- \| --- \| --- \| --- \| --- \| --- \| --- \| --- \| --- \| --- \| --- \| --- \| --- \| --- \| --- \| --- \| --- \| --- \| --- \| --- \| --- \| --- \| --- \| --- \| --- \| --- \| --- \| --- \| --- \| --- \| --- \| --- \| --- \| --- \| --- \| --- \| --- \| --- \| --- \| --- \| --- \| --- \| --- \| --- \| --- \| --- \| --- \| --- \| --- \| --- \| --- \| --- \| --- \| --- \| --- \| --- \| --- \| --- \| --- \| --- \| --- \| --- \| --- \| --- \| --- \| --- \| --- \| --- \| --- \| --- \| --- \| --- \| --- \| --- \| --- \| --- \| --- \| --- \| --- \| --- \| --- \| --- \| --- \| --- \| --- \| --- \| --- \| --- \| --- \| --- \| --- \| --- \| --- \| --- \| --- \| --- \| --- \| --- \| --- \| --- \| --- \| --- \| --- \| --- \| --- \| --- \| --- \| --- \| --- \| --- \| --- \| --- \| --- \| --- \| --- \| --- \| --- \| --- \| --- \| --- \| --- \| --- \| --- \| --- \| --- \| --- \| --- \| --- \| --- \| --- \| --- \| --- \| --- \| --- \| --- \| --- \| --- \| --- \| --- \| --- \| --- \| --- \| --- \| --- \| --- \| --- \| --- \| --- \| --- \| --- \| --- \| --- \| --- \| --- \| --- \| --- \| --- \| --- \| --- \| --- \| --- \| --- \| --- \| --- \| --- \| --- \| --- \| --- \| --- \| --- \| --- \| --- \| --- \| --- \| --- \| --- \| --- \| --- \| --- \| --- \| --- \| --- \| --- \| --- \| --- \| --- \| --- \| --- \| --- \| --- \| --- \| --- \| --- \| --- \| --- \| --- \| --- \| --- \| --- \| --- \| --- \| --- \| --- \| --- \| --- \| --- \| --- \| --- \| --- \| --- \| --- \| --- \| --- \| --- \| --- \| --- \| --- \| --- \| --- \| --- \| --- \| --- \| --- \| --- \| --- \| --- \| --- \| --- \| --- \| --- \| --- \| --- \| --- \| --- \| --- \| --- \| --- \| --- \| --- \| --- \| --- \| --- \| --- \| --- \| --- \| --- \| --- \| --- \| --- \| --- \| --- \| --- \| --- \| --- \| --- \| --- \| --- \| --- \| --- \| --- \| --- \| --- \| --- \| --- \| --- \| --- \| --- \| --- \| --- \| --- \| --- \| --- \| --- \| --- \| --- \| --- \| --- \| --- \| --- \| --- \| --- \| --- \| --- \| --- \| --- \| --- \| --- \| --- \| --- \| --- \| --- \| --- \| --- \| --- \| --- \| --- \| --- \| --- \| --- \| --- \| --- \| --- \| --- \| --- \| --- \| --- \| --- \| --- \| --- \| --- \| --- \| --- \| --- \| --- \| --- \| --- \| --- \| --- \| --- \| --- \| --- \| --- \| --- \| --- \| --- \| --- \| --- \| --- \| --- \| --- \| --- \| --- \| --- \| --- \| --- \| --- \| --- \| --- \| --- \| --- \| --- \| --- \| --- \| --- \| --- \| --- \| --- \| --- \| --- \| --- \| --- \| --- \| --- \| --- \| --- \| --- \| --- \| --- \| --- \| --- \| --- \| --- \| --- \| --- \| --- \| --- \| --- \| --- \| --- \| --- \| --- \| --- \| --- \| --- \| --- \| --- \| --- \| --- \| --- \| --- \| --- \| --- \| --- \| --- \| --- \| --- \| --- \| --- \| --- \| --- \| --- \| --- \| --- \| --- \| --- \| --- \| --- \| --- \| --- \| --- \| --- \| --- \| --- \| --- \| --- \| --- \| --- \| --- \| --- \| --- \| --- \| --- \| --- \| --- \| --- \| --- \| --- \| --- \| --- \| --- \| --- \| --- \| --- \| --- \| --- \| --- \| --- \| --- \| --- \| --- \| --- \| --- \| --- \| --- \| --- \| --- \| --- \| --- \| --- \| --- \| --- \| --- \| --- \| --- \| --- \| --- \| --- \| --- \| --- \| --- \| --- \| --- \| --- \| --- \| --- \| --- \| --- \| --- \| --- \| --- \| --- \| --- \| --- \| --- \| --- \| --- \| --- \| --- \| --- \| --- \| --- \| --- \| --- \| --- \| --- \| --- \| --- \| --- \| --- \| --- \| --- \| --- \| --- \| --- \| --- \| --- \| --- \| --- \| --- \| --- \| --- \| --- \| --- \| --- \| --- \| --- \| --- \| --- \| --- \| --- \| --- \| --- \| --- \| --- \| --- \| --- \| --- \| --- \| --- \| --- \| --- \| --- \| --- \| --- \| --- \| --- \| --- \| --- \| --- \| --- \| --- \| --- \| --- \| --- \| --- \| --- \| --- \| --- \| --- \| --- \| --- \| --- \| --- \| --- \| --- \| --- \| --- \| --- \| --- \| --- \| --- \| --- \| --- \| --- \| --- \| --- \| --- \| --- \| --- \| --- \| --- \| --- \| --- \| --- \| --- \| --- \| --- \| --- \| --- \| --- \| --- \| --- \| --- \| --- \| --- \| --- \| --- \| --- \| --- \| --- \| --- \| --- \| --- \| --- \| --- \| --- \| --- \| --- \| --- \| --- \| --- \| --- \| --- \| --- \| --- \| --- \| --- \| --- \| --- \| --- \| --- \| --- \| --- \| --- \| --- \| --- \| --- \| --- \| --- \| --- \| --- \| --- \| --- \| --- \| --- \| --- \| --- \| --- \| --- \| --- \| --- \| --- \| --- \| --- \| --- \| --- \| --- \| --- \| --- \| --- \| --- \| --- \| --- \| --- \| --- \| --- \| --- \| --- \| --- \| --- \| --- \| --- \| --- \| --- \| --- \| --- \| --- \| --- \| --- \| --- \| --- \| --- \| --- \| --- \| --- \| --- \| --- \| --- \| --- \| --- \| --- \| --- \| --- \| --- \| --- \| --- \| --- \| --- \| --- \| --- \| --- \| --- \| --- \| --- \| --- \| --- \| --- \| --- \| --- \| --- \| --- \| --- \| --- \| --- \| --- \| --- \| --- \| --- \| --- \| --- \| --- \| --- \| --- \| --- \| --- \| --- \| --- \| --- \| --- \| --- \| --- \| --- \| --- \| --- \| --- \| --- \| --- \| --- \| --- \| --- \| --- \| --- \| --- \| --- \| --- \| --- \| --- \| --- \| --- \| --- \| --- \| --- \| --- \| --- \| --- \| --- \| --- \| --- \| --- \| --- \| --- \| --- \| --- \| --- \| --- \| --- \| --- \| --- \| --- \| --- \| --- \| --- \| --- \| --- \| --- \| --- \| --- \| --- \| --- \| --- \| --- \| --- \| --- \| --- \| --- \| --- \| --- \| --- \| --- \| --- \| --- \| --- \| --- \| --- \| --- \| --- \| --- \| --- \| --- \| --- \| --- \| --- \| --- \| --- \| --- \| --- \| --- \| --- \| --- \| --- \| --- \| --- \| --- \| --- \| --- \| --- \| --- \| --- \| --- \| --- \| --- \| --- \| --- \| --- \| --- \| --- \| --- \| --- \| --- \| --- \| --- \| --- \| --- \| --- \| --- \| --- \| --- \| --- \| --- \| --- \| --- \| --- \| --- \| --- \| --- \| --- \| --- \| --- \| --- \| --- \| --- \| --- \| --- \| --- \| --- \| --- \| --- \| --- \| --- \| --- \| --- \| --- \| --- \| --- \| --- \| --- \| --- \| --- \| --- \| --- \| --- \| --- \| --- \| --- \| --- \| --- \| --- \| --- \| --- \| --- \| --- \| --- \| --- \| --- \| --- \| --- \| --- \| --- \| --- \| --- \| --- \| --- \| --- \| --- \| --- \| --- \| --- \| --- \| --- \| --- \| --- \| --- \| --- \| --- \| --- \| --- \| --- \| --- \| --- \| --- \| --- \| --- \| --- \| --- \| --- \| --- \| --- \| --- \| --- \| --- \| --- \| --- \| --- \| --- \| --- \| --- \| --- \| --- \| --- \| --- \| --- \| --- \| --- \| --- \| --- \| --- \| --- \| --- \| --- \| --- \| --- \| --- \| --- \| --- \| --- \| --- \| --- \| --- \| --- \| --- \| --- \| --- \| --- \| --- \| --- \| --- \| --- \| --- \| --- \| --- \| --- \| --- \| --- \| --- \| --- \| --- \| --- \| --- \| --- \| --- \| --- \| --- \| --- \| --- \| --- \| --- \| --- \| --- \| --- \| --- \| --- \| --- \| --- \| --- \| --- \| --- \| --- \| --- \| --- \| --- \| --- \| --- \| --- \| --- \| --- \| --- \| --- \| --- \| --- \| --- \| --- \| --- \| --- \| --- \| --- \| --- \| --- \| --- \| --- \| --- \| --- \| --- \| --- \| --- \| --- \| --- \| --- \| --- \| --- \| --- \| --- \| --- \| --- \| --- \| --- \| --- \| --- \| --- \| --- \| --- \| --- \| --- \| --- \| --- \| --- \| --- \| --- \| --- \| --- \| --- \| --- \| --- \| --- \| --- \| --- \| --- \| --- \| --- \| --- \| --- \| --- \| --- \| --- \| --- \| --- \| --- \| --- \| --- \| --- \| --- \| --- \| --- \| --- \| --- \| --- \| --- \| --- \| --- \| --- \| --- \| --- \| --- \| --- \| --- \| --- \| --- \| --- \| --- \| --- \| --- \| --- \| --- \| --- \| --- \| --- \| --- \| --- \| --- \| --- \| --- \| --- \| --- \| --- \| --- \| --- \| --- \| --- \| --- \| --- \| --- \| --- \| --- \| --- \| --- \| --- \| --- \| --- \| --- \| --- \| --- \| --- \| --- \| --- \| --- \| --- \| --- \| --- \| --- \| --- \| --- \| --- \| --- \| --- \| --- \| --- \| --- \| --- \| --- \| --- \| --- \| --- \| --- \| --- \| --- \| --- \| --- \| --- \| --- \| --- \| --- \| --- \| --- \| --- \| --- \| --- \| --- \| --- \| --- \| --- \| --- \| --- \| --- \| --- \| --- \| --- \| --- \| --- \| --- \| --- \| --- \| --- \| --- \| --- \| --- \| --- \| --- \| --- \| --- \| --- \| --- \| --- \| --- \| --- \| --- \| --- \| --- \| --- \| --- \| --- \| --- \| --- \| --- \| --- \| --- \| --- \| --- \| --- \| --- \| --- \| --- \| --- \| --- \| --- \| --- \| --- \| --- \| --- \| --- \| --- \| --- \| --- \| --- \| --- \| --- \| --- \| --- \| --- \| --- \| --- \| --- \| --- \| --- \| --- \| --- \| --- \| --- \| --- \| --- \| --- \| --- \| --- \| --- \| --- \| --- \| --- \| --- \| --- \| --- \| --- \| --- \| --- \| --- \| --- \| --- \| --- \| --- \| --- \| --- \| --- \| --- \| --- \| --- \| --- \| --- \| --- \| --- \| --- \| --- \| --- \| --- \| --- \| --- \| --- \| --- \| --- \| --- \| --- \| --- \| --- \| --- \| --- \| --- \| --- \| --- \| --- \| --- \| --- \| --- \| --- \| --- \| --- \| --- \| --- \| --- \| --- \| --- \| --- \| --- \| --- \| --- \| --- \| --- \| --- \| --- \| --- \| --- \| --- \| --- \| --- \| --- \| --- \| --- \| --- \| --- \| --- \| --- \| --- \| --- \| --- \| --- \| --- \| --- \| --- \| --- \| --- \| --- \| --- \| --- \| --- \| --- \| --- \| --- \| --- \| --- \| --- \| --- \| --- \| --- \| --- \| --- \| --- \| --- \| --- \| --- \| --- \| --- \| --- \| --- \| --- \| --- \| --- \| --- \| --- \| --- \| --- \| --- \| --- \| --- \| --- \| --- \| --- \| --- \| --- \| --- \| --- \| --- \| --- \| --- \| --- \| --- \| --- \| --- \| --- \| --- \| --- \| --- \| --- \| --- \| --- \| --- \| --- \| --- \| --- \| --- \| --- \| --- \| --- \| --- \| --- \| --- \| --- \| --- \| --- \| --- \| --- \| --- \| --- \| --- \| --- \| --- \| --- \| --- \| --- \| --- \| --- \| --- \| --- \| --- \| --- \| --- \| --- \| --- \| --- \| --- \| --- \| --- \| --- \| --- \| --- \| --- \| --- \| --- \| --- \| --- \| --- \| --- \| --- \| --- \| --- \| --- \| --- \| --- \| --- \| --- \| --- \| --- \| --- \| --- \| --- \| --- \| --- \| --- \| --- \| --- \| --- \| --- \| --- \| --- \| --- \| --- \| --- \| --- \| --- \| --- \| --- \| --- \| --- \| --- \| --- \| --- \| --- \| --- \| --- \| --- \| --- \| --- \| --- \| --- \| --- \| --- \| --- \| --- \| --- \| --- \| --- \| --- \| --- \| --- \| --- \| --- \| --- \| --- \| --- \| --- \| --- \| --- \| --- \| --- \| --- \| --- \| --- \| --- \| --- \| --- \| --- \| --- \| --- \| --- \| --- \| --- \| --- \| --- \| --- \| --- \| --- \| --- \| --- \| --- \| --- \| --- \| --- \| --- \| --- \| --- \| --- \| --- \| --- \| --- \| --- \| --- \| --- \| --- \| --- \| --- \| --- \| --- \| --- \| --- \| --- \| --- \| --- \| --- \| --- \| --- \| --- \| --- \| --- \| --- \| --- \| --- \| --- \| --- \| --- \| --- \| --- \| --- \| --- \| --- \| --- \| --- \| --- \| --- \| --- \| --- \| --- \| --- \| --- \| --- \| --- \| --- \| --- \| --- \| --- \| --- \| --- \| --- \| --- \| --- \| --- \| --- \| --- \| --- \| --- \| --- \| --- \| --- \| --- \| --- \| --- \| --- \| --- \| --- \| --- \| --- \| --- \| --- \| --- \| --- \| --- \| --- \| --- \| --- \| --- \| --- \| --- \| --- \| --- \| --- \| --- \| --- \| --- \| --- \| --- \| --- \| --- \| --- \| --- \| --- \| --- \| --- \| --- \| --- \| --- \| --- \| --- \| --- \| --- \| --- \| --- \| --- \| --- \| --- \| --- \| --- \| --- \| --- \| --- \| --- \| --- \| --- \| --- \| --- \| --- \| --- \| --- \| --- \| --- \| --- \| --- \| --- \| --- \| --- \| --- \| --- \| --- \| --- \| --- \| --- \| --- \| --- \| --- \| --- \| --- \| --- \| --- \| --- \| --- \| --- \| --- \| --- \| --- \| --- \| --- \| --- \| --- \| --- \| --- \| --- \| --- \| --- \| --- \| --- \| --- \| --- \| --- \| --- \| --- \| --- \| --- \| --- \| --- \| --- \| --- \| --- \| --- \| --- \| --- \| --- \| --- \| --- \| --- \| --- \| --- \| --- \| --- \| --- \| --- \| --- \| --- \| --- \| --- \| --- \| --- \| --- \| --- \| --- \| --- \| --- \| --- \| --- \| --- \| --- \| --- \| --- \| --- \| --- \| --- \| --- \| --- \| --- \| --- \| --- \| --- \| --- \| --- \| --- \| --- \| --- \| --- \| --- \| --- \| --- \| --- \| --- \| --- \| --- \| --- \| --- \| --- \| --- \| --- \| --- \| --- \| --- \| --- \| --- \| --- \| --- \| --- \| --- \| --- \| --- \| --- \| --- \| --- \| --- \| --- \| --- \| --- \| --- \| --- \| --- \| --- \| --- \| --- \| --- \| --- \| --- \| --- \| --- \| --- \| --- \| --- \| --- \| --- \| --- \| --- \| --- \| --- \| --- \| --- \| --- \| --- \| --- \| --- \| --- \| --- \| --- \| --- \| --- \| --- \| --- \| --- \| --- \| --- \| --- \| --- \| --- \| --- \| --- \| --- \| --- \| --- \| --- \| --- \| --- \| --- \| --- \| --- \| --- \| --- \| --- \| --- \| --- \| --- \| --- \| --- \| --- \| --- \| --- \| --- \| --- \| --- \| --- \| --- \| --- \| --- \| --- \| --- \| --- \| --- \| --- \| --- \| --- \| --- \| --- \| --- \| --- \| --- \| --- \| --- \| --- \| --- \| --- \| --- \| --- \| --- \| --- \| --- \| --- \| --- \| --- \| --- \| --- \| --- \| --- \| --- \| --- \| --- \| --- \| --- \| --- \| --- \| --- \| --- \| --- \| --- \| --- \| --- \| --- \| --- \| --- \| --- \| --- \| --- \| --- \| --- \| --- \| --- \| --- \| --- \| --- \| --- \| --- \| --- \| --- \| --- \| --- \| --- \| --- \| --- \| --- \| --- \| --- \| --- \| --- \| --- \| --- \| --- \| --- \| --- \| --- \| --- \| --- \| --- \| --- \| --- \| --- \| --- \| --- \| --- \| --- \| --- \| --- \| --- \| --- \| --- \| --- \| --- \| --- \| --- \| --- \| --- \| --- \| --- \| --- \| --- \| --- \| --- \| --- \| --- \| --- \| --- \| --- \| --- \| --- \| --- \| --- \| --- \| --- \| --- \| --- \| --- \| --- \| --- \| --- \| --- \| --- \| --- \| --- \| --- \| --- \| --- \| --- \| --- \| --- \| --- \| --- \| --- \| --- \| --- \| --- \| --- \| --- \| --- \| --- \| --- \| --- \| --- \| --- \| --- \| --- \| --- \| --- \| --- \| --- \| --- \| --- \| --- \| --- \| --- \| --- \| --- \| --- \| --- \| --- \| --- \| --- \| --- \| --- \| --- \| --- \| --- \| --- \| --- \| --- \| --- \| --- \| --- \| --- \| --- \| --- \| --- \| --- \| --- \| --- \| --- \| --- \| --- \| --- \| --- \| --- \| --- \| --- \| --- \| --- \| --- \| --- \| --- \| --- \| --- \| --- \| --- \| --- \| --- \| --- \| --- \| --- \| --- \| --- \| --- \| --- \| --- \| --- \| --- \| --- \| --- \| --- \| --- \| --- \| --- \| --- \| --- \| --- \| --- \| --- \| --- \| --- \| --- \| --- \| --- \| --- \| --- \| --- \| --- \| --- \| --- \| --- \| --- \| --- \| --- \| --- \| --- \| --- \| --- \| --- \| --- \| --- \| --- \| --- \| --- \| --- \| --- \| --- \| --- \| --- \| --- \| --- \| --- \| --- \| --- \| --- \| --- \| --- \| --- \| --- \| --- \| --- \| --- \| --- \| --- \| --- \| --- \| --- \| --- \| --- \| --- \| --- \| --- \| --- \| --- \| --- \| --- \| --- \| --- \| --- \| --- \| --- \| --- \| --- \| --- \| --- \| --- \| --- \| --- \| --- \| --- \| --- \| --- \| --- \| --- \| --- \| --- \| --- \| --- \| --- \| --- \| --- \| --- \| --- \| --- \| --- \| --- \| --- \| --- \| --- \| --- \| --- \| --- \| --- \| --- \| --- \| --- \| --- \| --- \| --- \| --- \| --- \| --- \| --- \| --- \| --- \| --- \| --- \| --- \| --- \| --- \| --- \| --- \| --- \| --- \| --- \| --- \| --- \| --- \| --- \| --- \| --- \| --- \| --- \| --- \| --- \| --- \| --- \| --- \| --- \| --- \| --- \| --- \| --- \| --- \| --- \| --- \| --- \| --- \| --- \| --- \| --- \| --- \| --- \| --- \| --- \| --- \| --- \| --- \| --- \| --- \| --- \| --- \| --- \| --- \| --- \| --- \| --- \| --- \| --- \| --- \| --- \| --- \| --- \| --- \| --- \| --- \| --- \| --- \| --- \| --- \| --- \| --- \| --- \| --- \| --- \| --- \| --- \| --- \| --- \| --- \| --- \| --- \| --- \| --- \| --- \| --- \| --- \| --- \| --- \| --- \| --- \| --- \| --- \| --- \| --- \| --- \| --- \| --- \| --- \| --- \| --- \| --- \| --- \| --- \| --- \| --- \| --- \| --- \| --- \| --- \| --- \| --- \| --- \| --- \| --- \| --- \| --- \| --- \| --- \| --- \| --- \| --- \| --- \| --- \| --- \| --- \| --- \| --- \| --- \| --- \| --- \| --- \| --- \| --- \| --- \| --- \| --- \| --- \| --- \| --- \| --- \| --- \| --- \| --- \| --- \| --- \| --- \| --- \| --- \| --- \| --- \| --- \| --- \| --- \| --- \| --- \| --- \| --- \| --- \| --- \| --- \| --- \| --- \| --- \| --- \| --- \| --- \| --- \| --- \| --- \| --- \| --- \| --- \| --- \| --- \| --- \| --- \| --- \| --- \| --- \| --- \| --- \| --- \| --- \| --- \| --- \| --- \| --- \| --- \| --- \| --- \| --- \| --- \| --- \| --- \| --- \| --- \| --- \| --- \| --- \| --- \| --- \| --- \| --- \| --- \| --- \| --- \| --- \| --- \| --- \| --- \| --- \| --- \| --- \| --- \| --- \| --- \| --- \| --- \| --- \| --- \| --- \| --- \| --- \| --- \| --- \| --- \| --- \| --- \| --- \| --- \| --- \| --- \| --- \| --- \| --- \| --- \| --- \| --- \| --- \| --- \| --- \| --- \| --- \| --- \| --- \| --- \| --- \| --- \| --- \| --- \| --- \| --- \| --- \| --- \| --- \| --- \| --- \| --- \| --- \| --- \| --- \| --- \| --- \| --- \| --- \| --- \| --- \| --- \| --- \| --- \| --- \| --- \| --- \| --- \| --- \| --- \| --- \| --- \| --- \| --- \| --- \| --- \| --- \| --- \| --- \| --- \| --- \| --- \| --- \| --- \| --- \| --- \| --- \| --- \| --- \| --- \| --- \| --- \| --- \| --- \| --- \| --- \| --- \| --- \| --- \| --- \| --- \| --- \| --- \| --- \| --- \| --- \| --- \| --- \| --- \| --- \| --- \| --- \| --- \| --- \| --- \| --- \| --- \| --- \| --- \| --- \| --- \| --- \| --- \| --- \| --- \| --- \| --- \| --- \| --- \| --- \| --- \| --- \| --- \| --- \| --- \| --- \| --- \| --- \| --- \| --- \| --- \| --- \| --- \| --- \| --- \| --- \| --- \| --- \| --- \| --- \| --- \| --- \| --- \| --- \| --- \| --- \| --- \| --- \| --- \| --- \| --- \| --- \| --- \| --- \| --- \| --- \| --- \| --- \| --- \| --- \| --- \| --- \| --- \| --- \| --- \| --- \| --- \| --- \| --- \| --- \| --- \| --- \| --- \| --- \| --- \| --- \| --- \| --- \| --- \| --- \| --- \| --- \| --- \| --- \| --- \| --- \| --- \| --- \| --- \| --- \| --- \| --- \| --- \| --- \| --- \| --- \| --- \| --- \| --- \| --- \| --- \| --- \| --- \| --- \| --- \| --- \| --- \| --- \| --- \| --- \| --- \| --- \| --- \| --- \| --- \| --- \| --- \| --- \| --- \| --- \| --- \| --- \| --- \| --- \| --- \| --- \| --- \| --- \| --- \| --- \| --- \| --- \| --- \| --- \| --- \| --- \| --- \| --- \| --- \| --- \| --- \| --- \| --- \| --- \| --- \| --- \| --- \| --- \| --- \| --- \| --- \| --- \| --- \| --- \| --- \| --- \| --- \| --- \| --- \| --- \| --- \| --- \| --- \| --- \| --- \| --- \| --- \| --- \| --- \| --- \| --- \| --- \| --- \| --- \| --- \| --- \| --- \| --- \| --- \| --- \| --- \| --- \| --- \| --- \| --- \| --- \| --- \| --- \| --- \| --- \| --- \| --- \| --- \| --- \| --- \| --- \| --- \| --- \| --- \| --- \| --- \| --- \| --- \| --- \| --- \| --- \| --- \| --- \| --- \| --- \| --- \| --- \| --- \| --- \| --- \| --- \| --- \| --- \| --- \| --- \| --- \| --- \| --- \| --- \| --- \| --- \| --- \| --- \| --- \| --- \| --- \| --- \| --- \| --- \| --- \| --- \| --- \| --- \| --- \| --- \| --- \| --- \| --- \| --- \| --- \| --- \| --- \| --- \| --- \| --- \| --- \| --- \| --- \| --- \| --- \| --- \| --- \| --- \| --- \| --- \| --- \| --- \| --- \| --- \| --- \| --- \| --- \| --- \| --- \| --- \| --- \| --- \| --- \| --- \| --- \| --- \| --- \| --- \| --- \| --- \| --- \| --- \| --- \| --- \| --- \| --- \| --- \| --- \| --- \| --- \| --- \| --- \| --- \| --- \| --- \| --- \| --- \| --- \| --- \| --- \| --- \| --- \| --- \| --- \| --- \| --- \| --- \| --- \| --- \| --- \| --- \| --- \| --- \| --- \| --- \| --- \| --- \| --- \| --- \| --- \| --- \| --- \| --- \| --- \| --- \| --- \| --- \| --- \| --- \| --- \| --- \| --- \| --- \| --- \| --- \| --- \| --- \| --- \| --- \| --- \| --- \| --- \| --- \| --- \| --- \| --- \| --- \| --- \| --- \| --- \| --- \| --- \| --- \| --- \| --- \| --- \| --- \| --- \| --- \| --- \| --- \| --- \| --- \| --- \| --- \| --- \| --- \| --- \| --- \| --- \| --- \| --- \| --- \| --- \| --- \| --- \| --- \| --- \| --- \| --- \| --- \| --- \| --- \| --- \| --- \| --- \| --- \| --- \| --- \| --- \| --- \| --- \| --- \| --- \| --- \| --- \| --- \| --- \| --- \| --- \| --- \| --- \| --- \| --- \| --- \| --- \| --- \| --- \| --- \| --- \| --- \| --- \| --- \| --- \| --- \| --- \| --- \| --- \| --- \| --- \| --- \| --- \| --- \| --- \| --- \| --- \| --- \| --- \| --- \| --- \| --- \| --- \| --- \| --- \| --- \| --- \| --- \| --- \| --- \| --- \| --- \| --- \| --- \| --- \| --- \| --- \| --- \| --- \| --- \| --- \| --- \| --- \| --- \| --- \| --- \| --- \| --- \| --- \| --- \| --- \| --- \| --- \| --- \| --- \| --- \| --- \| --- \| --- \| --- \| --- \| --- \| --- \| --- \| --- \| --- \| --- \| --- \| --- \| --- \| --- \| --- \| --- \| --- \| --- \| --- \| --- \| --- \| --- \| --- \| --- \| --- \| --- \| --- \| --- \| --- \| --- \| --- \| --- \| --- \| --- \| --- \| --- \| --- \| --- \| --- \| --- \| --- \| --- \| --- \| --- \| --- \| --- \| --- \| --- \| --- \| --- \| --- \| --- \| --- \| --- \| --- \| --- \| --- \| --- \| --- \| --- \| --- \| --- \| --- \| --- \| --- \| --- \| --- \| --- \| --- \| --- \| --- \| --- \| --- \| --- \| --- \| --- \| --- \| --- \| --- \| --- \| --- \| --- \| --- \| --- \| --- \| --- \| --- \| --- \| --- \| --- \| --- \| --- \| --- \| --- \| --- \| --- \| --- \| --- \| --- \| --- \| --- \| --- \| --- \| --- \| --- \| --- \| --- \| --- \| --- \| --- \| --- \| --- \| --- \| --- \| --- \| --- \| --- \| --- \| --- \| --- \| --- \| --- \| --- \| --- \| --- \| --- \| --- \| --- \| --- \| --- \| --- \| --- \| --- \| --- \| --- \| --- \| --- \| --- \| --- \| --- \| --- \| --- \| --- \| --- \| --- \| --- \| --- \| --- \| --- \| --- \| --- \| --- \| --- \| --- \| --- \| --- \| --- \| --- \| --- \| --- \| --- \| --- \| --- \| --- \| --- \| --- \| --- \| --- \| --- \| --- \| --- \| --- \| --- \| --- \| --- \| --- \| --- \| --- \| --- \| --- \| --- \| --- \| --- \| --- \| --- \| --- \| --- \| --- \| --- \| --- \| --- \| --- \| --- \| --- \| --- \| --- \| --- \| --- \| --- \| --- \| --- \| --- \| --- \| --- \| --- \| --- \| --- \| --- \| --- \| --- \| --- \| --- \| --- \| --- \| --- \| --- \| --- \| --- \| --- \| --- \| --- \| --- \| --- \| --- \| --- \| --- \| --- \| --- \| --- \| --- \| --- \| --- \| --- \| --- \| --- \| --- \| --- \| --- \| --- \| --- \| --- \| --- \| --- \| --- \| --- \| --- \| --- \| --- \| --- \| --- \| --- \| --- \| --- \| --- \| --- \| --- \| --- \| --- \| --- \| --- \| --- \| --- \| --- \| --- \| --- \| --- \| --- \| --- \| --- \| --- \| --- \| --- \| --- \| --- \| --- \| --- \| --- \| --- \| --- \| --- \| --- \| --- \| --- \| --- \| --- \| --- \| --- \| --- \| --- \| --- \| --- \| --- \| --- \| --- \| --- \| --- \| --- \| --- \| --- \| --- \| --- \| --- \| --- \| --- \| --- \| --- \| --- \| --- \| --- \| --- \| --- \| --- \| --- \| --- \| --- \| --- \| --- \| --- \| --- \| --- \| --- \| --- \| --- \| --- \| --- \| --- \| --- \| --- \| --- \| --- \| --- \| --- \| --- \| --- \| --- \| --- \| --- \| --- \| --- \| --- \| --- \| --- \| --- \| --- \| --- \| --- \| --- \| --- \| --- \| --- \| --- \| --- \| --- \| --- \| --- \| --- \| --- \| --- \| --- \| --- \| --- \| --- \| --- \| --- \| --- \| --- \| --- \| --- \| --- \| --- \| --- \| --- \| --- \| --- \| --- \| --- \| --- \| --- \| --- \| --- \| --- \| --- \| --- \| --- \| --- \| --- \| --- \| --- \| --- \| --- \| --- \| --- \| --- \| --- \| --- \| --- \| --- \| --- \| --- \| --- \| --- \| --- \| --- \| --- \| --- \| --- \| --- \| --- \| --- \| --- \| --- \| --- \| --- \| --- \| --- \| --- \| --- \| --- \| --- \| --- \| --- \| --- \| --- \| --- \| --- \| --- \| --- \| --- \| --- \| --- \| --- \| --- \| --- \| --- \| --- \| --- \| --- \| --- \| --- \| --- \| --- \| --- \| --- \| --- \| --- \| --- \| --- \| --- \| --- \| --- \| --- \| --- \| --- \| --- \| --- \| --- \| --- \| --- \| --- \| --- \| --- \| --- \| --- \| --- \| --- \| --- \| --- \| --- \| --- \| --- \| --- \| --- \| --- \| --- \| --- \| --- \| --- \| --- \| --- \| --- \| --- \| --- \| --- \| --- \| --- \| --- \| --- \| --- \| --- \| --- \| --- \| --- \| --- \| --- \| --- \| --- \| --- \| --- \| --- \| --- \| --- \| --- \| --- \| --- \| --- \| --- \| --- \| --- \| --- \| --- \| --- \| --- \| --- \| --- \| --- \| --- \| --- \| --- \| --- \| --- \| --- \| --- \| --- \| --- \| --- \| --- \| --- \| --- \| --- \| --- \| --- \| --- \| --- \| --- \| --- \| --- \| --- \| --- \| --- \| --- \| --- \| --- \| --- \| --- \| --- \| --- \| --- \| --- \| --- \| --- \| --- \| --- \| --- \| --- \| --- \| --- \| --- \| --- \| --- \| --- \| --- \| --- \| --- \| --- \| --- \| --- \| --- \| --- \| --- \| --- \| --- \| --- \| --- \| --- \| --- \| --- \| --- \| --- \| --- \| --- \| --- \| --- \| --- \| --- \| --- \| --- \| --- \| --- \| --- \| --- \| --- \| --- \| --- \| --- \| --- \| --- \| --- \| --- \| --- \| --- \| --- \| --- \| --- \| --- \| --- \| --- \| --- \| --- \| --- \| --- \| --- \| --- \| --- \| --- \| --- \| --- \| --- \| --- \| --- \| --- \| --- \| --- \| --- \| --- \| --- \| --- \| --- \| --- \| --- \| --- \| --- \| --- \| --- \| --- \| --- \| --- \| --- \| --- \| --- \| --- \| --- \| --- \| --- \| --- \| --- \| --- \| --- \| --- \| --- \| --- \| --- \| --- \| --- \| --- \| --- \| --- \| --- \| --- \| --- \| --- \| --- \| --- \| --- \| --- \| --- \| --- \| --- \| --- \| --- \| --- \| --- \| --- \| --- \| --- \| --- \| --- \| --- \| --- \| --- \| --- \| --- \| --- \| --- \| --- \| --- \| --- \| --- \| --- \| --- \| --- \| --- \| --- \| --- \| --- \| --- \| --- \| --- \| --- \| --- \| --- \| --- \| --- \| --- \| --- \| --- \| --- \| --- \| --- \| --- \| --- \| --- \| --- \| --- \| --- \| --- \| --- \| --- \| --- \| --- \| --- \| --- \| --- \| --- \| --- \| --- \| --- \| --- \| --- \| --- \| --- \| --- \| --- \| --- \| --- \| --- \| --- \| --- \| --- \| --- \| --- \| --- \| --- \| --- \| --- \| --- \| --- \| --- \| --- \| --- \| --- \| --- \| --- \| --- \| --- \| --- \| --- \| --- \| --- \| --- \| --- \| --- \| --- \| --- \| --- \| --- \| --- \| --- \| --- \| --- \| --- \| --- \| --- \| --- \| --- \| --- \| --- \| --- \| --- \| --- \| --- \| --- \| --- \| --- \| --- \| --- \| --- \| --- \| --- \| --- \| --- \| --- \| --- \| --- \| --- \| --- \| --- \| --- \| --- \| --- \| --- \| --- \| --- \| --- \| --- \| --- \| --- \| --- \| --- \| --- \| --- \| --- \| --- \| --- \| --- \| --- \| --- \| --- \| --- \| --- \| --- \| --- \| --- \| --- \| --- \| --- \| --- \| --- \| --- \| --- \| --- \| --- \| --- \| --- \| --- \| --- \| --- \| --- \| --- \| --- \| --- \| --- \| --- \| --- \| --- \| --- \| --- \| --- \| --- \| --- \| --- \| --- \| --- \| --- \| --- \| --- \| --- \| --- \| --- \| --- \| --- \| --- \| --- \| --- \| --- \| --- \| --- \| --- \| --- \| --- \| --- \| --- \| --- \| --- \| --- \| --- \| --- \| --- \| --- \| --- \| --- \| --- \| --- \| --- \| --- \| --- \| --- \| --- \| --- \| --- \| --- \| --- \| --- \| --- \| --- \| --- \| --- \| --- \| --- \| --- \| --- \| --- \| --- \| --- \| --- \| --- \| --- \| --- \| --- \| --- \| --- \| --- \| --- \| --- \| --- \| --- \| --- \| --- \| --- \| --- \| --- \| --- \| --- \| --- \| --- \| --- \| --- \| --- \| --- \| --- \| --- \| --- \| --- \| --- \| --- \| --- \| --- \| --- \| --- \| --- \| --- \| --- \| --- \| --- \| --- \| --- \| --- \| --- \| --- \| --- \| --- \| --- \| --- \| --- \| --- \| --- \| --- \| --- \| --- \| --- \| --- \| --- \| --- \| --- \| --- \| --- \| --- \| --- \| --- \| --- \| --- \| --- \| --- \| --- \| --- \| --- \| --- \| --- \| --- \| --- \| --- \| --- \| --- \| --- \| --- \| --- \| --- \| --- \| --- \| --- \| --- \| --- \| --- \| --- \| --- \| --- \| --- \| --- \| --- \| --- \| --- \| --- \| --- \| --- \| --- \| --- \| --- \| --- \| --- \| --- \| --- \| --- \| --- \| --- \| --- \| --- \| --- \| --- \| --- \| --- \| --- \| --- \| --- \| --- \| --- \| --- \| --- \| --- \| --- \| --- \| --- \| --- \| --- \| --- \| --- \| --- \| --- \| --- \| --- \| --- \| --- \| --- \| --- \| --- \| --- \| --- \| --- \| --- \| --- \| --- \| --- \| --- \| --- \| --- \| --- \| --- \| --- \| --- \| --- \| --- \| --- \| --- \| --- \| --- \| --- \| --- \| --- \| --- \| --- \| --- \| --- \| --- \| --- \| --- \| --- \| --- \| --- \| --- \| --- \| --- \| --- \| --- \| --- \| --- \| --- \| --- \| --- \| --- \| --- \| --- \| --- \| --- \| --- \| --- \| --- \| --- \| --- \| --- \| --- \| --- \| --- \| --- \| --- \| --- \| --- \| --- \| --- \| --- \| --- \| --- \| --- \| --- \| --- \| --- \| --- \| --- \| --- \| --- \| --- \| --- \| --- \| --- \| --- \| --- \| --- \| --- \| --- \| --- \| --- \| --- \| --- \| --- \| --- \| --- \| --- \| --- \| --- \| --- \| --- \| --- \| --- \| --- \| --- \| --- \| --- \| --- \| --- \| --- \| --- \| --- \| --- \| --- \| --- \| --- \| --- \| --- \| --- \| --- \| --- \| --- \| --- \| --- \| --- \| --- \| --- \| --- \| --- \| --- \| --- \| --- \| --- \| --- \| --- \| --- \| --- \| --- \| --- \| --- \| --- \| --- \| --- \| --- \| --- \| --- \| --- \| --- \| --- \| --- \| --- \| --- \| --- \| --- \| --- \| --- \| --- \| --- \| --- \| --- \| --- \| --- \| --- \| --- \| --- \| --- \| --- \| --- \| --- \| --- \| --- \| --- \| --- \| --- \| --- \| --- \| --- \| --- \| --- \| --- \| --- \| --- \| --- \| --- \| --- \| --- \| --- \| --- \| --- \| --- \| --- \| --- \| --- \| --- \| --- \| --- \| --- \| --- \| --- \| --- \| --- \| --- \| --- \| --- \| --- \| --- \| --- \| --- \| --- \| --- \| --- \| --- \| --- \| --- \| --- \| --- \| --- \| --- \| --- \| --- \| --- \| --- \| --- \| --- \| --- \| --- \| --- \| --- \| --- \| --- \| --- \| --- \| --- \| --- \| --- \| --- \| --- \| --- \| --- \| --- \| --- \| --- \| --- \| --- \| --- \| --- \| --- \| --- \| --- \| --- \| --- \| --- \| --- \| --- \| --- \| --- \| --- \| --- \| --- \| --- \| --- \| --- \| --- \| --- \| --- \| --- \| --- \| --- \| --- \| --- \| --- \| --- \| --- \| --- \| --- \| --- \| --- \| --- \| --- \| --- \| --- \| --- \| --- \| --- \| --- \| --- \| --- \| --- \| --- \| --- \| --- \| --- \| --- \| --- \| --- \| --- \| --- \| --- \| --- \| --- \| --- \| --- \| --- \| --- \| --- \| --- \| --- \| --- \| --- \| --- \| --- \| --- \| --- \| --- \| --- \| --- \| --- \| --- \| --- \| --- \| --- \| --- \| --- \| --- \| --- \| --- \| --- \| --- \| --- \| --- \| --- \| --- \| --- \| --- \| --- \| --- \| --- \| --- \| --- \| --- \| --- \| --- \| --- \| --- \| --- \| --- \| --- \| --- \| --- \| --- \| --- \| --- \| --- \| --- \| --- \| --- \| --- \| --- \| --- \| --- \| --- \| --- \| --- \| --- \| --- \| --- \| --- \| --- \| --- \| --- \| --- \| --- \| --- \| --- \| --- \| --- \| --- \| --- \| --- \| --- \| --- \| --- \| --- \| --- \| --- \| --- \| --- \| --- \| --- \| --- \| --- \| --- \| --- \| --- \| --- \| --- \| --- \| --- \| --- \| --- \| --- \| --- \| --- \| --- \| --- \| --- \| --- \| --- \| --- \| --- \| --- \| --- \| --- \| --- \| --- \| --- \| --- \| --- \| --- \| --- \| --- \| --- \| --- \| --- \| --- \| --- \| --- \| --- \| --- \| --- \| --- \| --- \| --- \| --- \| --- \| --- \| --- \| --- \| --- \| --- \| --- \| --- \| --- \| --- \| --- \| --- \| --- \| --- \| --- \| --- \| --- \| --- \| --- \| --- \| --- \| --- \| --- \| --- \| --- \| --- \| --- \| --- \| --- \| --- \| --- \| --- \| --- \| --- \| --- \| --- \| --- \| --- \| --- \| --- \| --- \| --- \| --- \| --- \| --- \| --- \| --- \| --- \| --- \| --- \| --- \| --- \| --- \| --- \| --- \| --- \| --- \| --- \| --- \| --- \| --- \| --- \| --- \| --- \| --- \| --- \| --- \| --- \| --- \| --- \| --- \| --- \| --- \| --- \| --- \| --- \| --- \| --- \| --- \| --- \| --- \| --- \| --- \| --- \| --- \| --- \| --- \| --- \| --- \| --- \| --- \| --- \| --- \| --- \| --- \| --- \| --- \| --- \| --- \| --- \| --- \| --- \| --- \| --- \| --- \| --- \| --- \| --- \| --- \| --- \| --- \| --- \| --- \| --- \| --- \| --- \| --- \| --- \| --- \| --- \| --- \| --- \| --- \| --- \| --- \| --- \| --- \| --- \| --- \| --- \| --- \| --- \| --- \| --- \| --- \| --- \| --- \| --- \| --- \| --- \| --- \| --- \| --- \| --- \| --- \| --- \| --- \| --- \| --- \| --- \| --- \| --- \| --- \| --- \| --- \| --- \| --- \| --- \| --- \| --- \| --- \| --- \| --- \| --- \| --- \| --- \| --- \| --- \| --- \| --- \| --- \| --- \| --- \| --- \| --- \| --- \| --- \| --- \| --- \| --- \| --- \| --- \| --- \| --- \| --- \| --- \| --- \| --- \| --- \| --- \| --- \| --- \| --- \| --- \| --- \| --- \| --- \| --- \| --- \| --- \| --- \| --- \| --- \| --- \| --- \| --- \| --- \| --- \| --- \| --- \| --- \| --- \| --- \| --- \| --- \| --- \| --- \| --- \| --- \| --- \| --- \| --- \| --- \| --- \| --- \| --- \| --- \| --- \| --- \| --- \| --- \| --- \| --- \| --- \| --- \| --- \| --- \| --- \| --- \| --- \| --- \| --- \| --- \| --- \| --- \| --- \| --- \| --- \| --- \| --- \| --- \| --- \| --- \| --- \| --- \| --- \| --- \| --- \| --- \| --- \| --- \| --- \| --- \| --- \| --- \| --- \| --- \| --- \| --- \| --- \| --- \| --- \| --- \| --- \| --- \| --- \| --- \| --- \| --- \| --- \| --- \| --- \| --- \| --- \| --- \| --- \| --- \| --- \| --- \| --- \| --- \| --- \| --- \| --- \| --- \| --- \| --- \| --- \| --- \| --- \| --- \| --- \| --- \| --- \| --- \| --- \| --- \| --- \| --- \| --- \| --- \| --- \| --- \| --- \| --- \| --- \| --- \| --- \| --- \| --- \| --- \| --- \| --- \| --- \| --- \| --- \| --- \| --- \| --- \| --- \| --- \| --- \| --- \| --- \| --- \| --- \| --- \| --- \| --- \| --- \| --- \| --- \| --- \| --- \| --- \| --- \| --- \| --- \| --- \| --- \| --- \| --- \| --- \| --- \| --- \| --- \| --- \| --- \| --- \| --- \| --- \| --- \| --- \| --- \| --- \| --- \| --- \| --- \| --- \| --- \| --- \| --- \| --- \| --- \| --- \| --- \| --- \| --- \| --- \| --- \| --- \| --- \| --- \| --- \| --- \| --- \| --- \| --- \| --- \| --- \| --- \| --- \| --- \| --- \| --- \| --- \| --- \| --- \| --- \| --- \| --- \| --- \| --- \| --- \| --- \| --- \| --- \| --- \| --- \| --- \| --- \| --- \| --- \| --- \| --- \| --- \| --- \| --- \| --- \| --- \| --- \| --- \| --- \| --- \| --- \| --- \| --- \| --- \| --- \| --- \| --- \| --- \| --- \| --- \| --- \| --- \| --- \| --- \| --- \| --- \| --- \| --- \| --- \| --- \| --- \| --- \| --- \| --- \| --- \| --- \| --- \| --- \| --- \| --- \| --- \| --- \| --- \| --- \| --- \| --- \| --- \| --- \| --- \| --- \| --- \| --- \| --- \| --- \| --- \| --- \| --- \| --- \| --- \| --- \| --- \| --- \| --- \| --- \| --- \| --- \| --- \| --- \| --- \| --- \| --- \| --- \| --- \| --- \| --- \| --- \| --- \| --- \| --- \| --- \| --- \| --- \| --- \| --- \| --- \| --- \| --- \| --- \| --- \| --- \| --- \| --- \| --- \| --- \| --- \| --- \| --- \| --- \| --- \| --- \| --- \| --- \| --- \| --- \| --- \| --- \| --- \| --- \| --- \| --- \| --- \| --- \| --- \| --- \| --- \| --- \| --- \| --- \| --- \| --- \| --- \| --- \| --- \| --- \| --- \| --- \| --- \| --- \| --- \| --- \| --- \| --- \| --- \| --- \| --- \| --- \| --- \| --- \| --- \| --- \| --- \| --- \| --- \| --- \| --- \| --- \| --- \| --- \| --- \| --- \| --- \| --- \| --- \| --- \| --- \| --- \| --- \| --- \| --- \| --- \| --- \| --- \| --- \| --- \| --- \| --- \| --- \| --- \| --- \| --- \| --- \| --- \| --- \| --- \| --- \| --- \| --- \| --- \| --- \| --- \| --- \| --- \| --- \| --- \| --- \| --- \| --- \| --- \| --- \| --- \| --- \| --- \| --- \| --- \| --- \| --- \| --- \| --- \| --- \| --- \| --- \| --- \| --- \| --- \| --- \| --- \| --- \| --- \| --- \| --- \| --- \| --- \| --- \| --- \| --- \| --- \| --- \| --- \| --- \| --- \| --- \| --- \| --- \| --- \| --- \| --- \| --- \| --- \| --- \| --- \| --- \| --- \| --- \| --- \| --- \| --- \| --- \| --- \| --- \| --- \| --- \| --- \| --- \| --- \| --- \| --- \| --- \| --- \| --- \| --- \| --- \| --- \| --- \| --- \| --- \| --- \| --- \| --- \| --- \| --- \| --- \| --- \| --- \| --- \| --- \| --- \| --- \| --- \| --- \| --- \| --- \| --- \| --- \| --- \| --- \| --- \| --- \| --- \| --- \| --- \| --- \| --- \| --- \| --- \| --- \| --- \| --- \| --- \| --- \| --- \| --- \| --- \| --- \| --- \| --- \| --- \| --- \| --- \| --- \| --- \| --- \| --- \| --- \| --- \| --- \| --- \| --- \| --- \| --- \| --- \| --- \| --- \| --- \| --- \| --- \| --- \| --- \| --- \| --- \| --- \| --- \| --- \| --- \| --- \| --- \| --- \| --- \| --- \| --- \| --- \| --- \| --- \| --- \| --- \| --- \| --- \| --- \| --- \| --- \| --- \| --- \| --- \| --- \| --- \| --- \| --- \| --- \| --- \| --- \| --- \| --- \| --- \| --- \| --- \| --- \| --- \| --- \| --- \| --- \| --- \| --- \| --- \| --- \| --- \| --- \| --- \| --- \| --- \| --- \| --- \| --- \| --- \| --- \| --- \| --- \| --- \| --- \| --- \| --- \| --- \| --- \| --- \| --- \| --- \| --- \| --- \| --- \| --- \| --- \| --- \| --- \| --- \| --- \| --- \| --- \| --- \| --- \| --- \| --- \| --- \| --- \| --- \| --- \| --- \| --- \| --- \| --- \| --- \| --- \| --- \| --- \| --- \| --- \| --- \| --- \| --- \| --- \| --- \| --- \| --- \| --- \| --- \| --- \| --- \| --- \| --- \| --- \| --- \| --- \| --- \| --- \| --- \| --- \| --- \| --- \| --- \| --- \| --- \| --- \| --- \| --- \| --- \| --- \| --- \| --- \| --- \| --- \| --- \| --- \| --- \| --- \| --- \| --- \| --- \| --- \| --- \| --- \| --- \| --- \| --- \| --- \| --- \| --- \| --- \| --- \| --- \| --- \| --- \| --- \| --- \| --- \| --- \| --- \| --- \| --- \| --- \| --- \| --- \| --- \| --- \| --- \| --- \| --- \| --- \| --- \| --- \| --- \| --- \| --- \| --- \| --- \| --- \| --- \| --- \| --- \| --- \| --- \| --- \| --- \| --- \| --- \| --- \| --- \| --- \| --- \| --- \| --- \| --- \| --- \| --- \| --- \| --- \| --- \| --- \| --- \| --- \| --- \| --- \| --- \| --- \| --- \| --- \| --- \| --- \| --- \| --- \| --- \| --- \| --- \| --- \| --- \| --- \| --- \| --- \| --- \| --- \| --- \| --- \| --- \| --- \| --- \| --- \| --- \| --- \| --- \| --- \| --- \| --- \| --- \| --- \| --- \| --- \| --- \| --- \| --- \| --- \| --- \| --- \| --- \| --- \| --- \| --- \| --- \| --- \| --- \| --- \| --- \| --- \| --- \| --- \| --- \| --- \| --- \| --- \| --- \| --- \| --- \| --- \| --- \| --- \| --- \| --- \| --- \| --- \| --- \| --- \| --- \| --- \| --- \| --- \| --- \| --- \| --- \| --- \| --- \| --- \| --- \| --- \| --- \| --- \| --- \| --- \| --- \| --- \| --- \| --- \| --- \| --- \| --- \| --- \| --- \| --- \| --- \| --- \| --- \| --- \| --- \| --- \| --- \| --- \| --- \| --- \| --- \| --- \| --- \| --- \| --- \| --- \| --- \| --- \| --- \| --- \| --- \| --- \| --- \| --- \| --- \| --- \| --- \| --- \| --- \| --- \| --- \| --- \| --- \| --- \| --- \| --- \| --- \| --- \| --- \| --- \| --- \| --- \| --- \| --- \| --- \| --- \| --- \| --- \| --- \| --- \| --- \| --- \| --- \| --- \| --- \| --- \| --- \| --- \| --- \| --- \| --- \| --- \| --- \| --- \| --- \| --- \| --- \| --- \| --- \| --- \| --- \| --- \| --- \| --- \| --- \| --- \| --- \| --- \| --- \| --- \| | | | |
|  | | | |
| \| **Electrophoretogram** \| \| --- \|   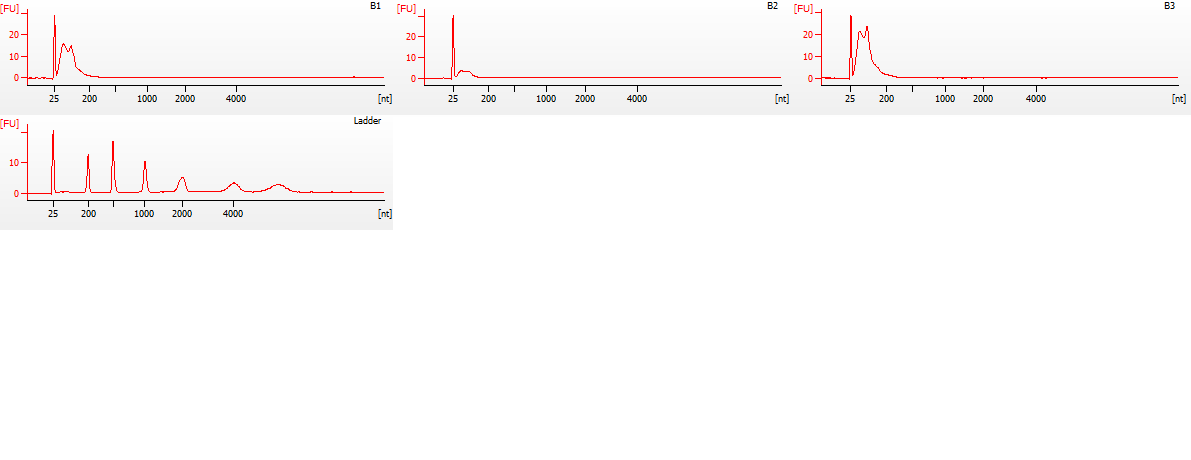  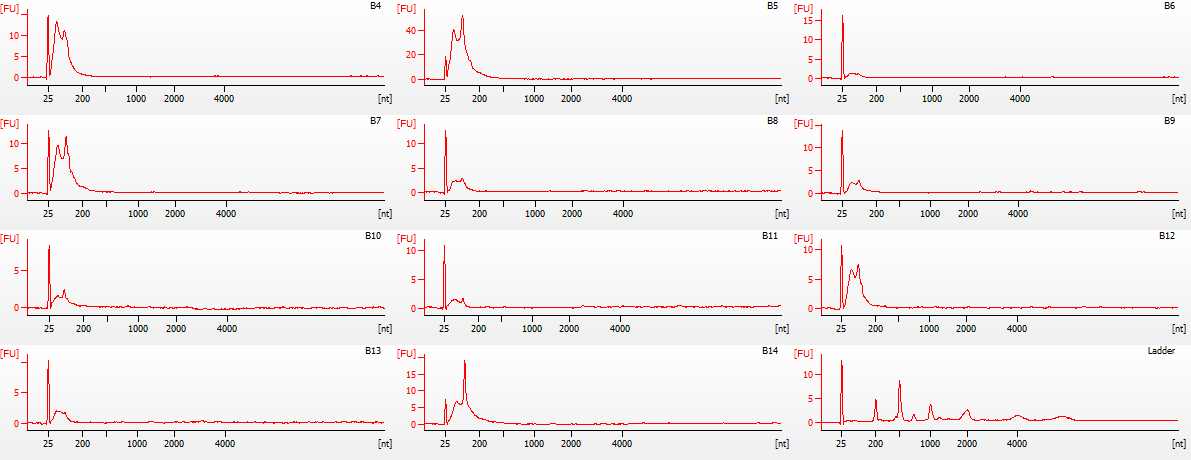  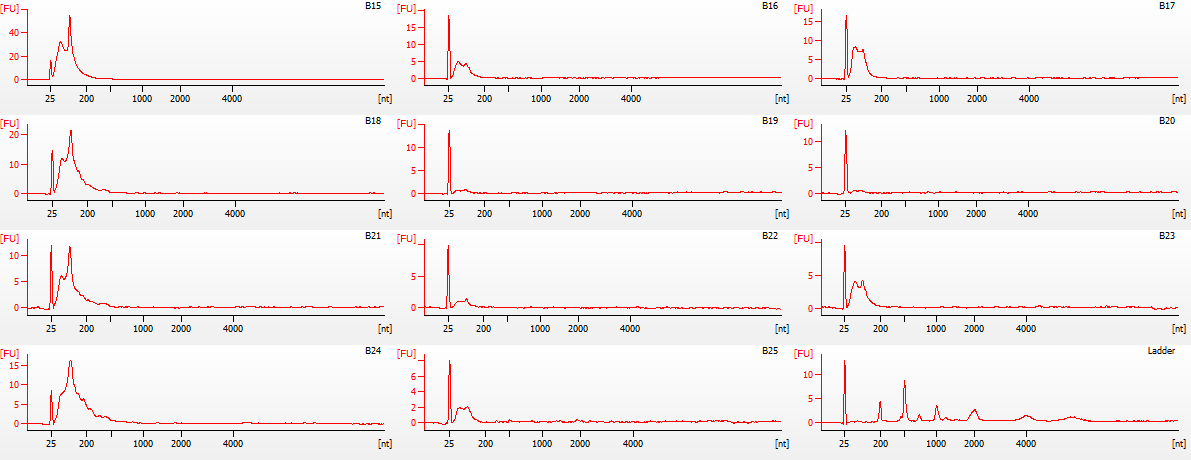  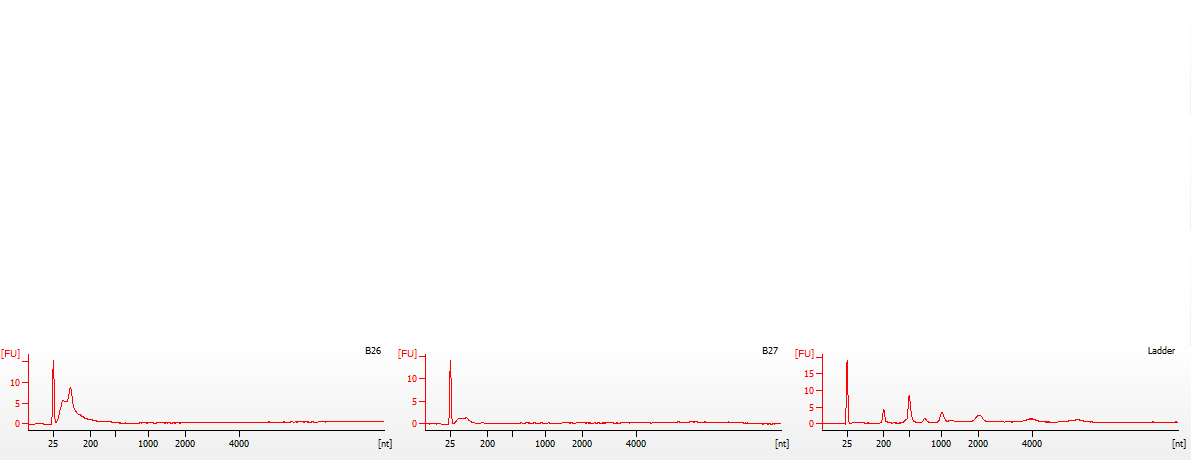  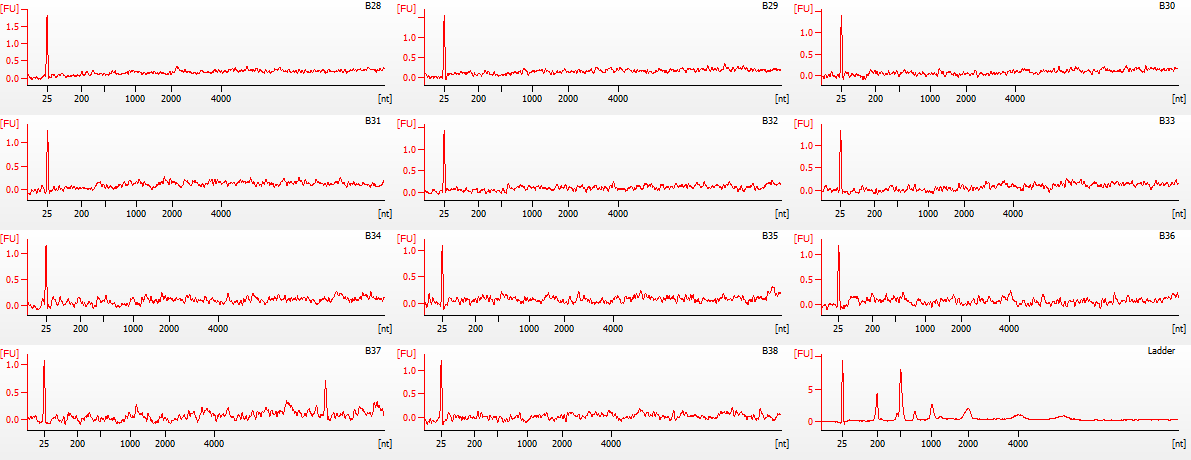  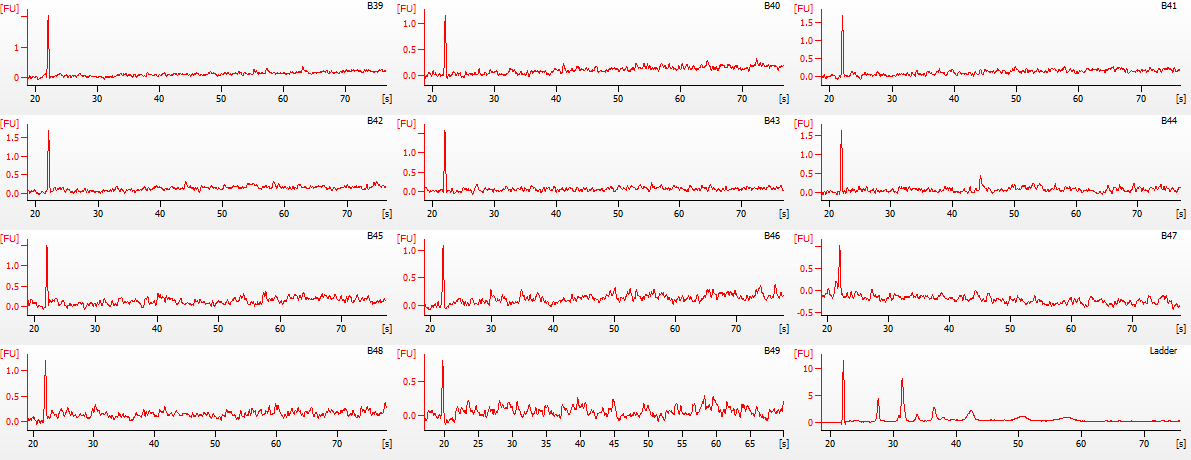  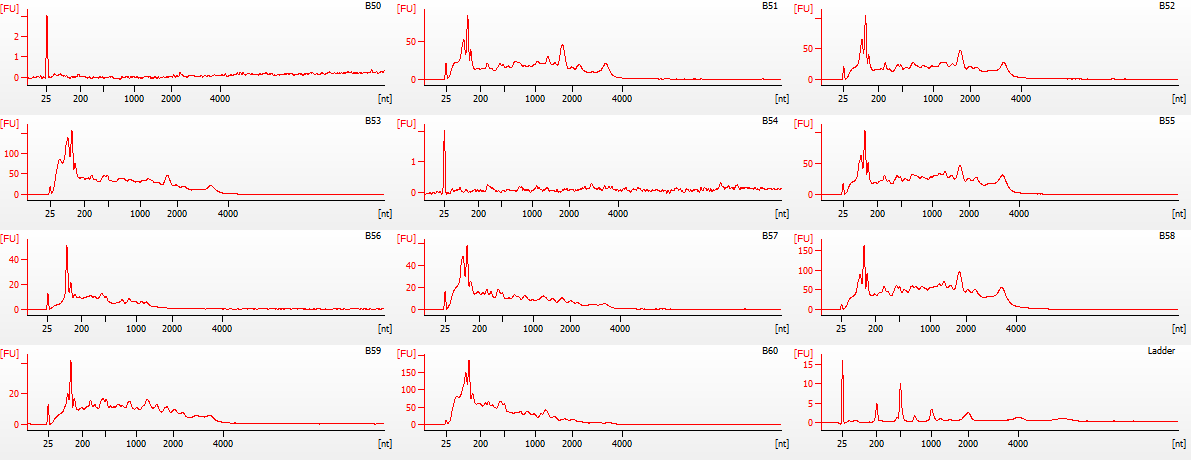  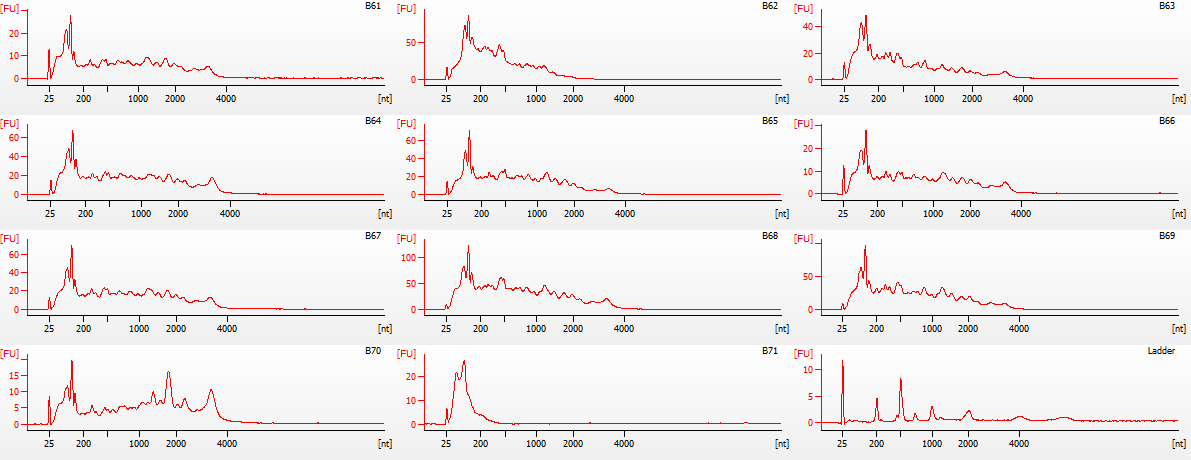  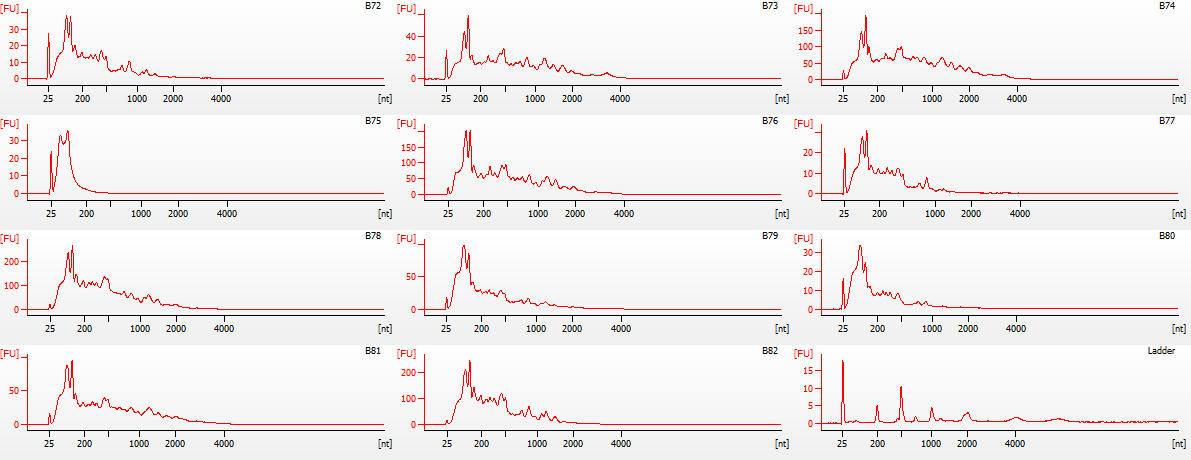  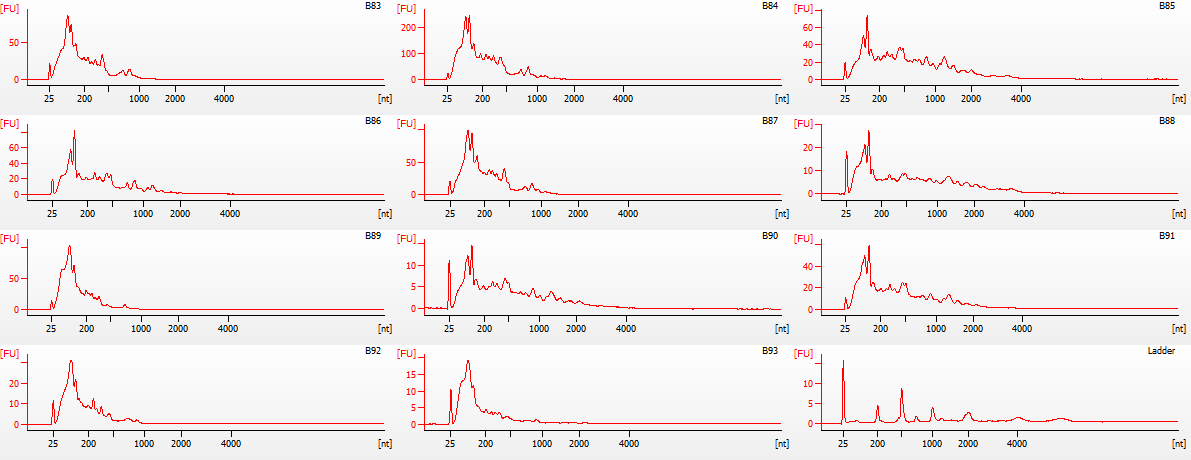  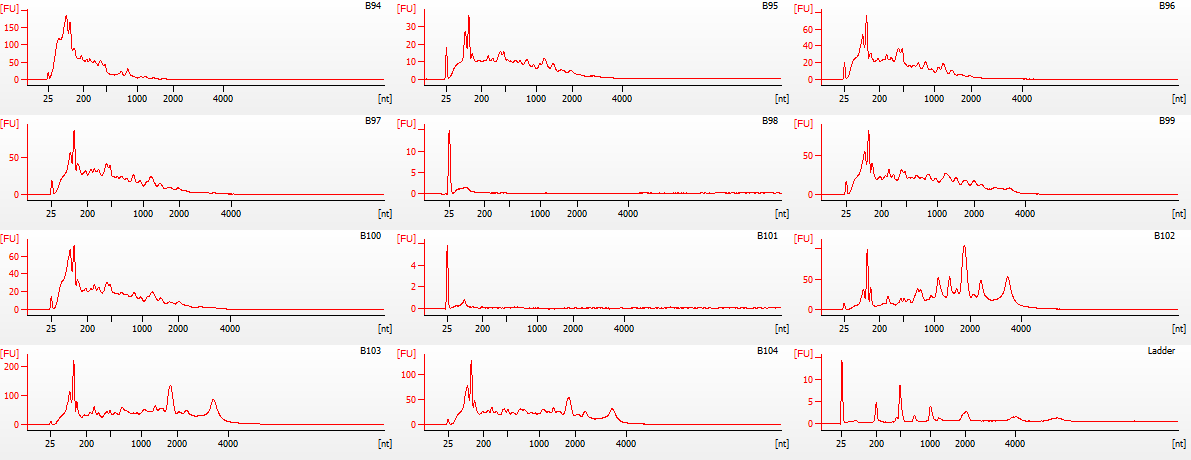  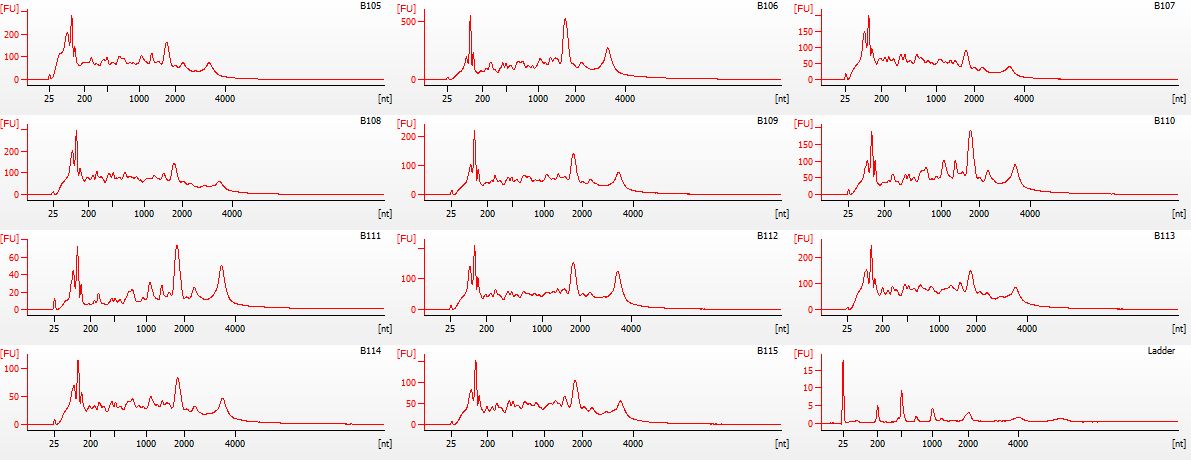  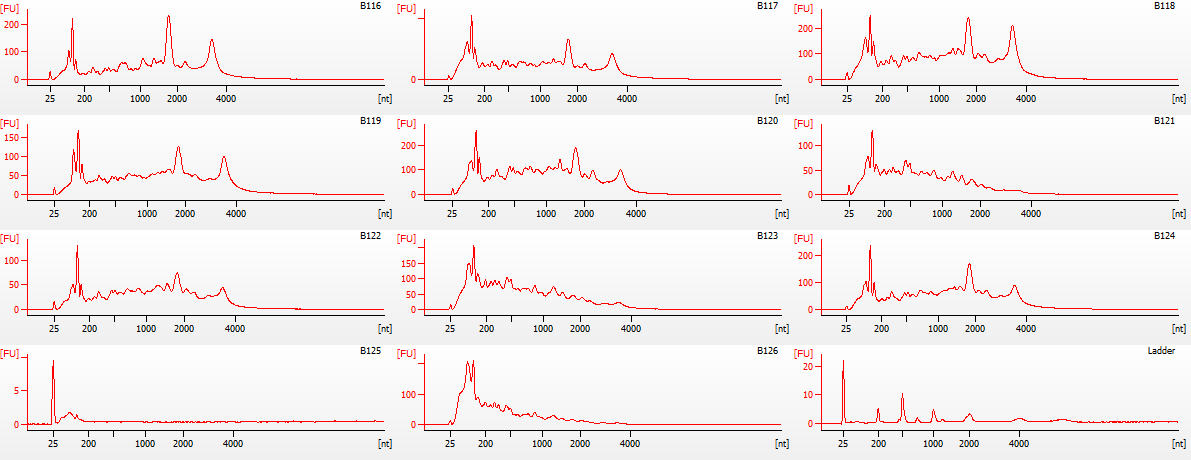  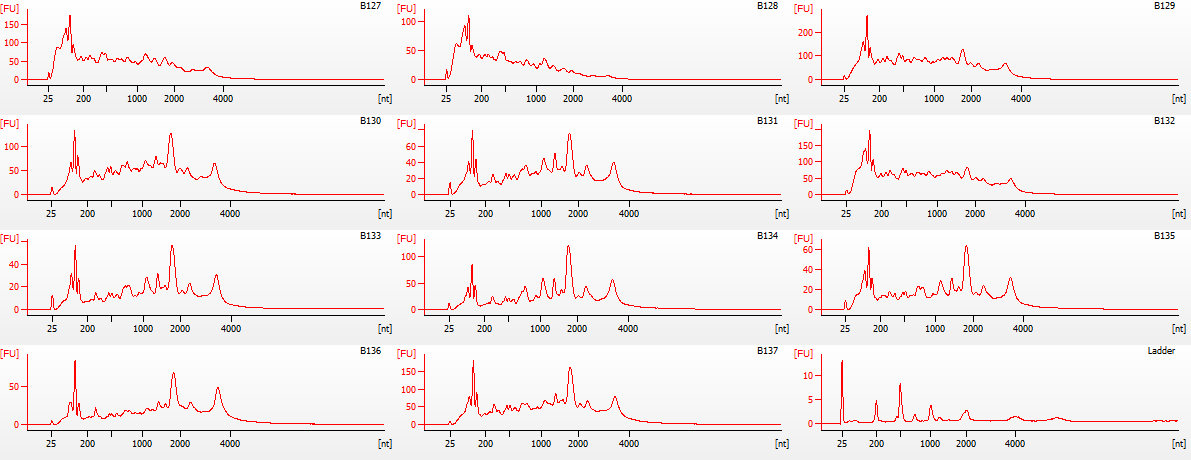  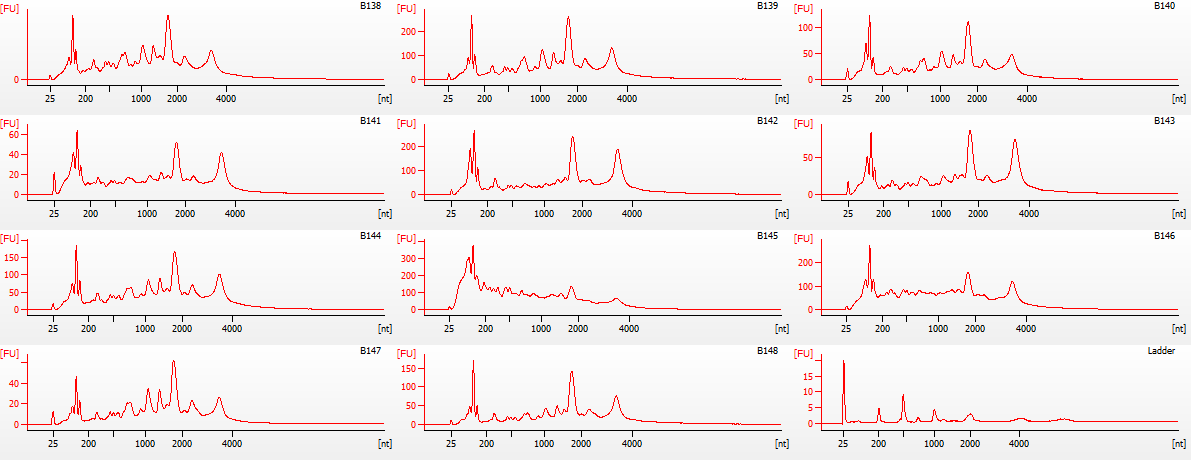  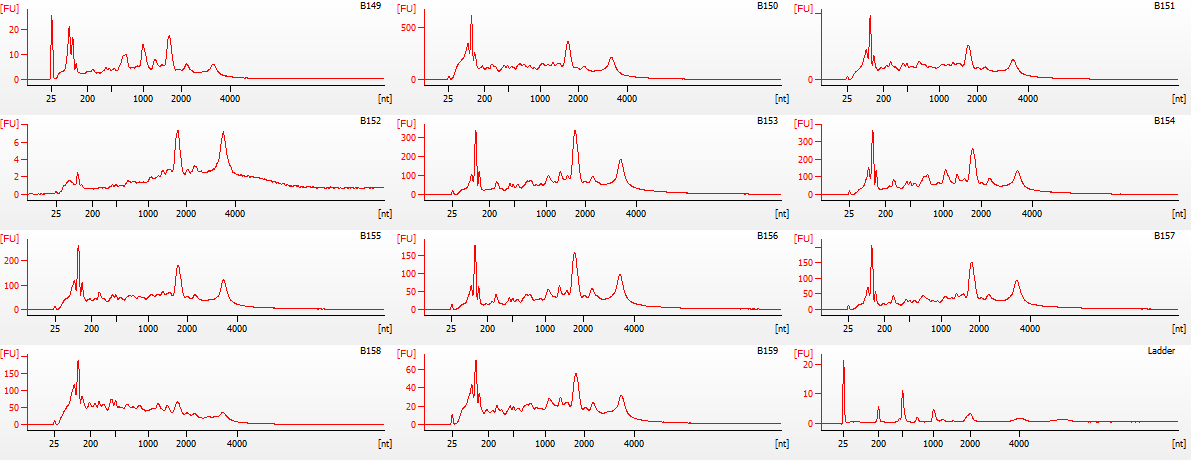  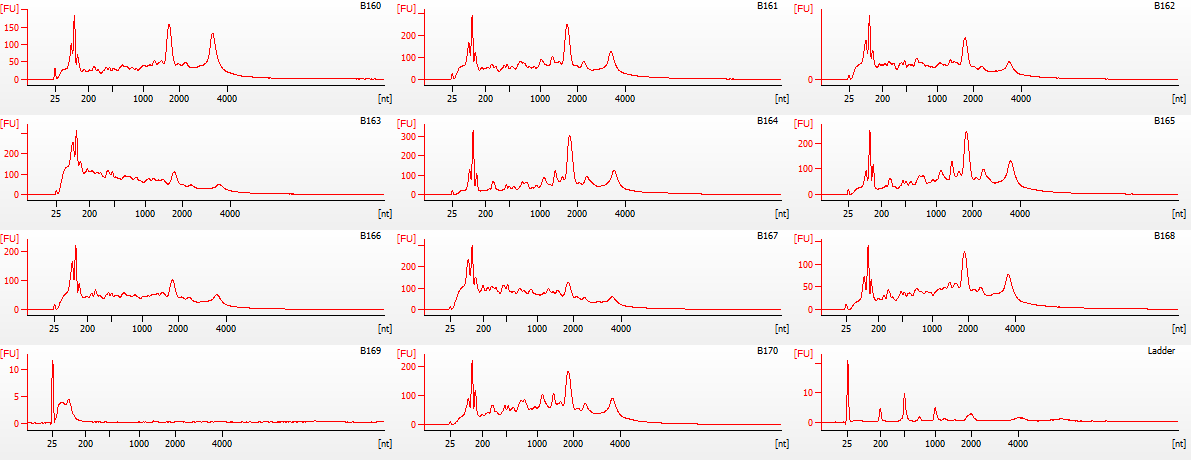  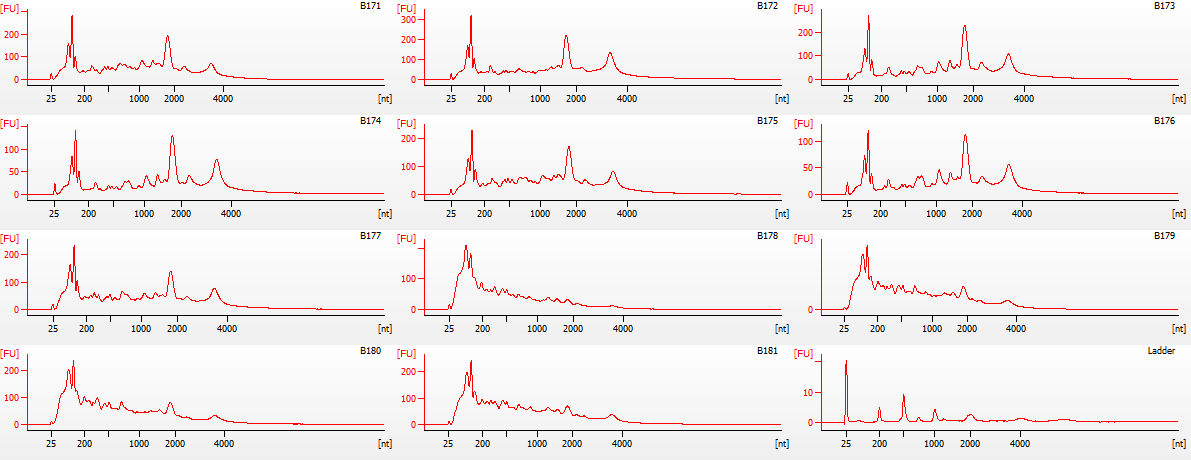  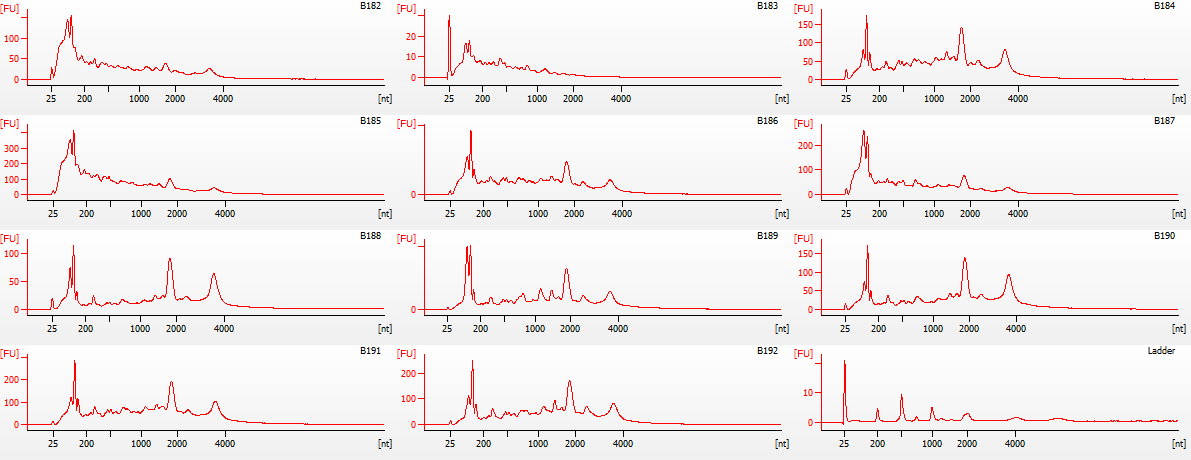  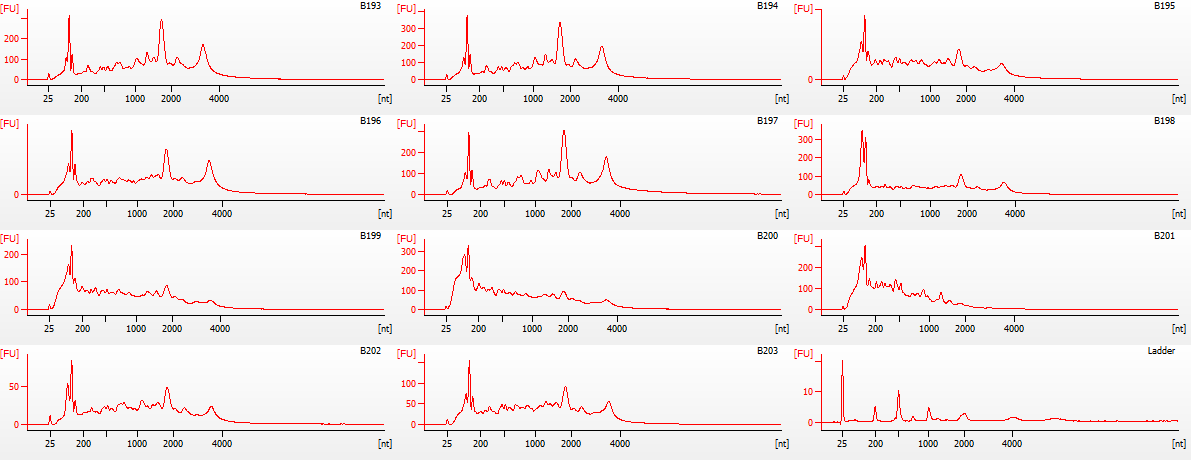  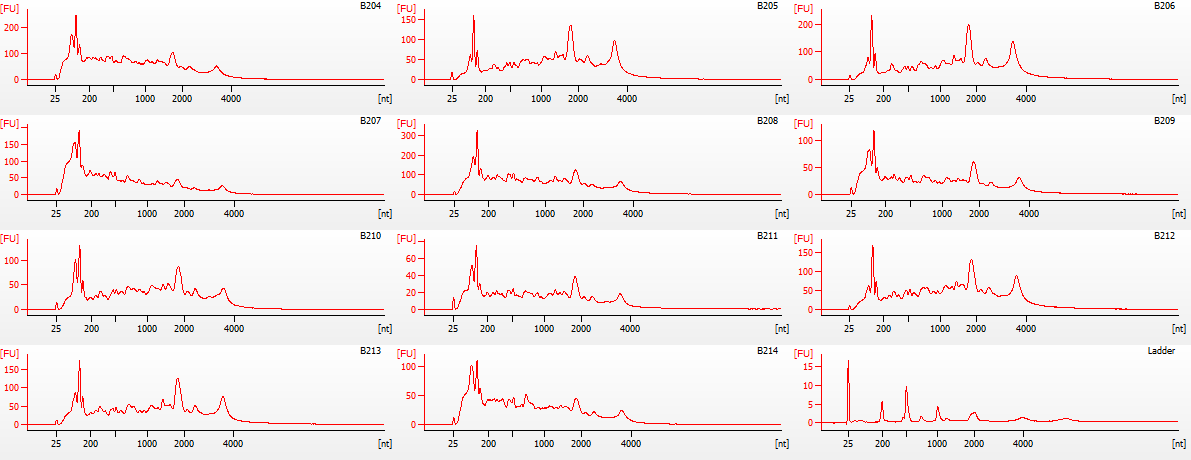  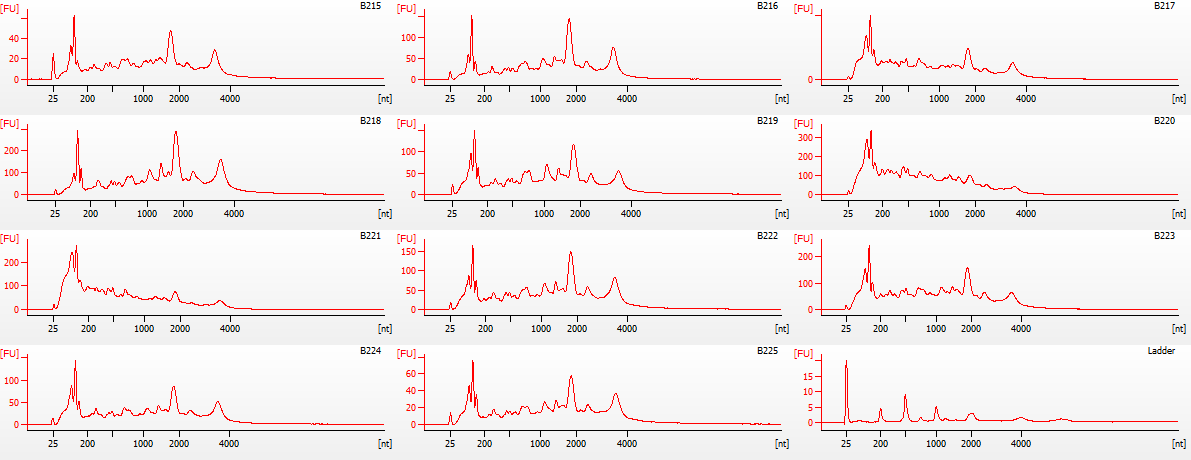  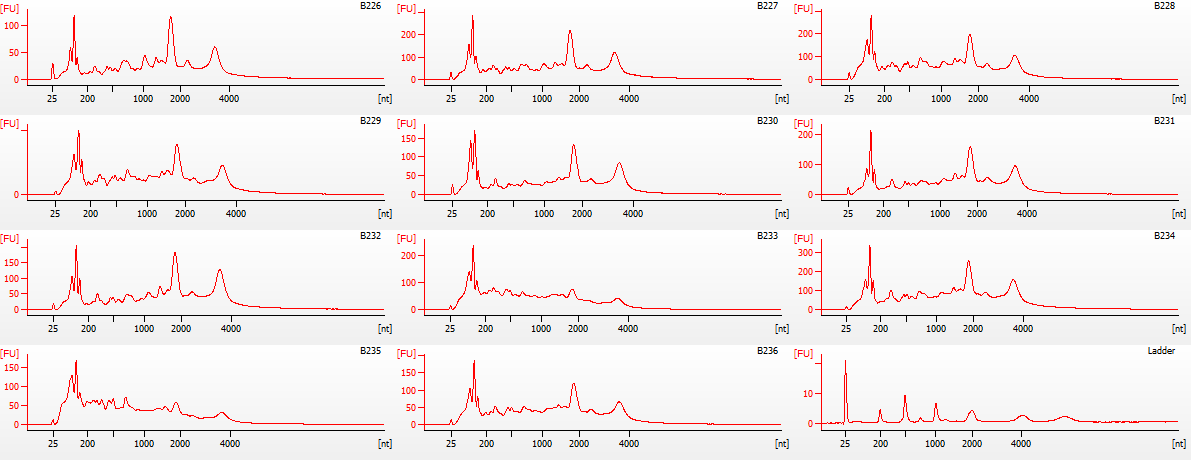  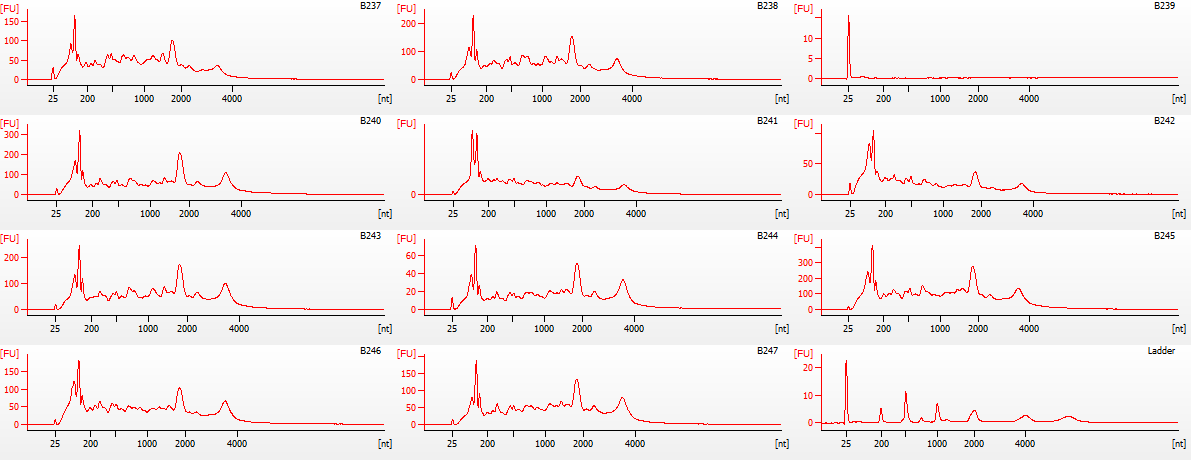  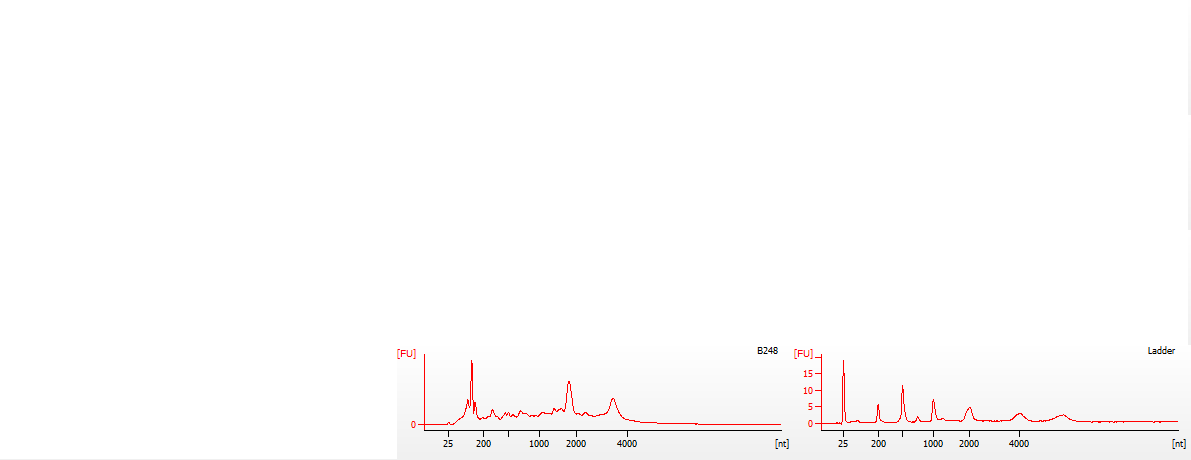  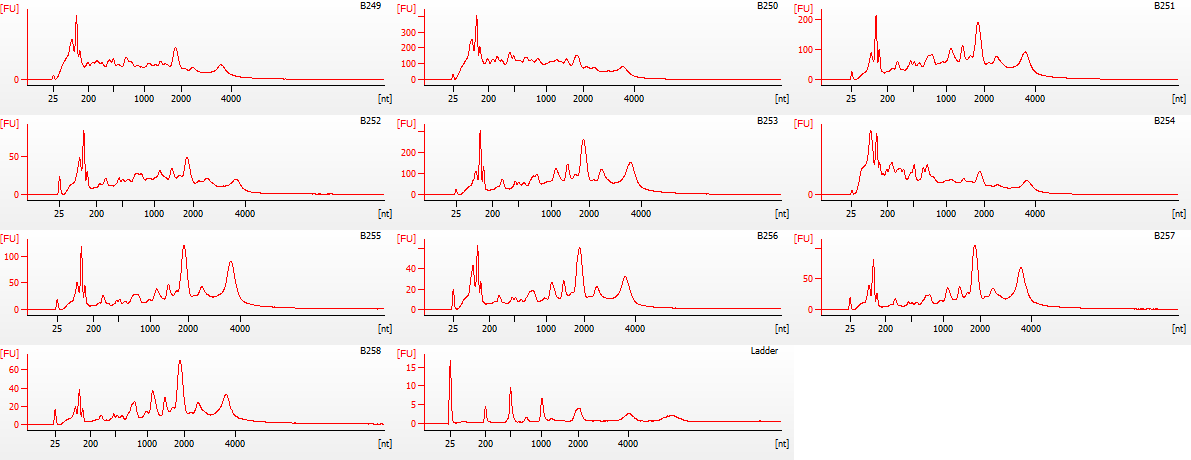  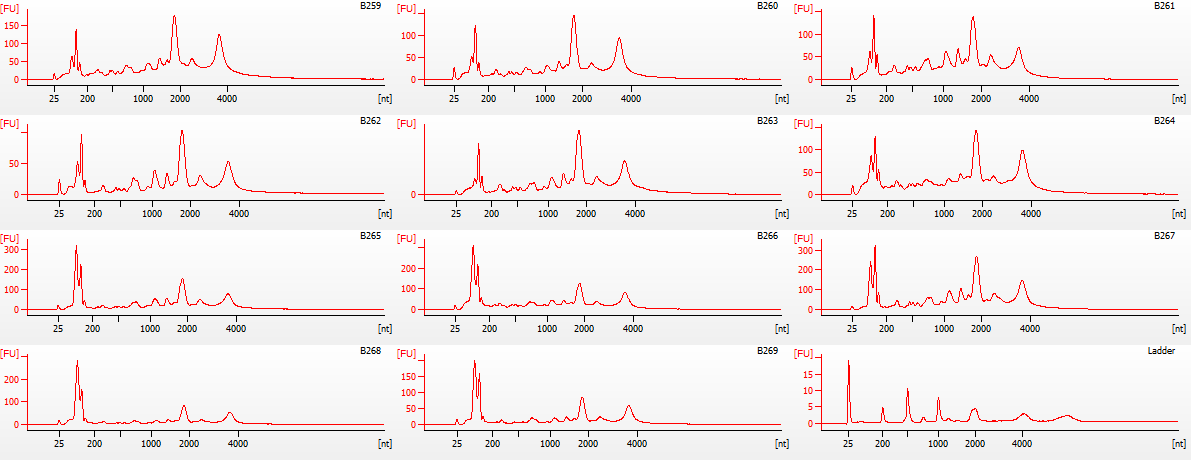  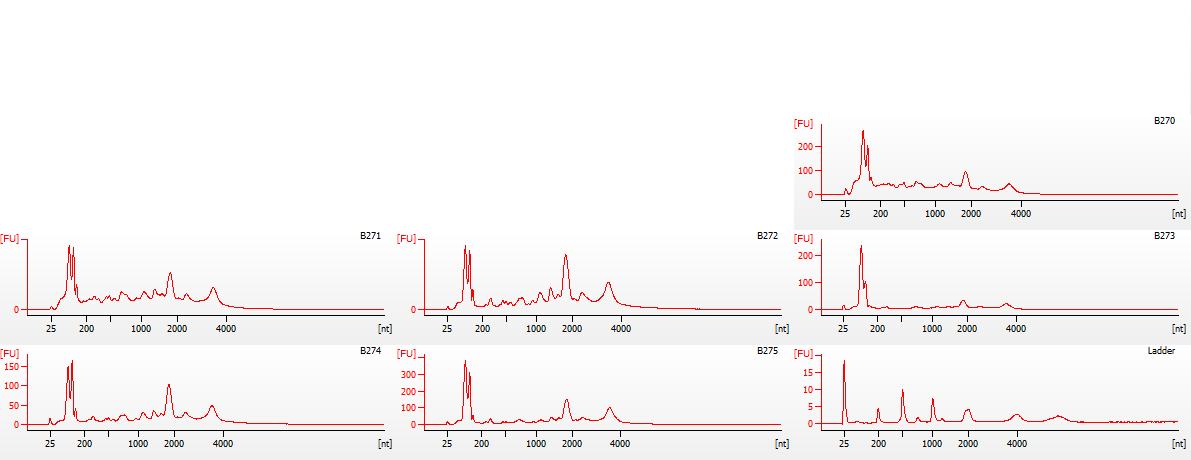  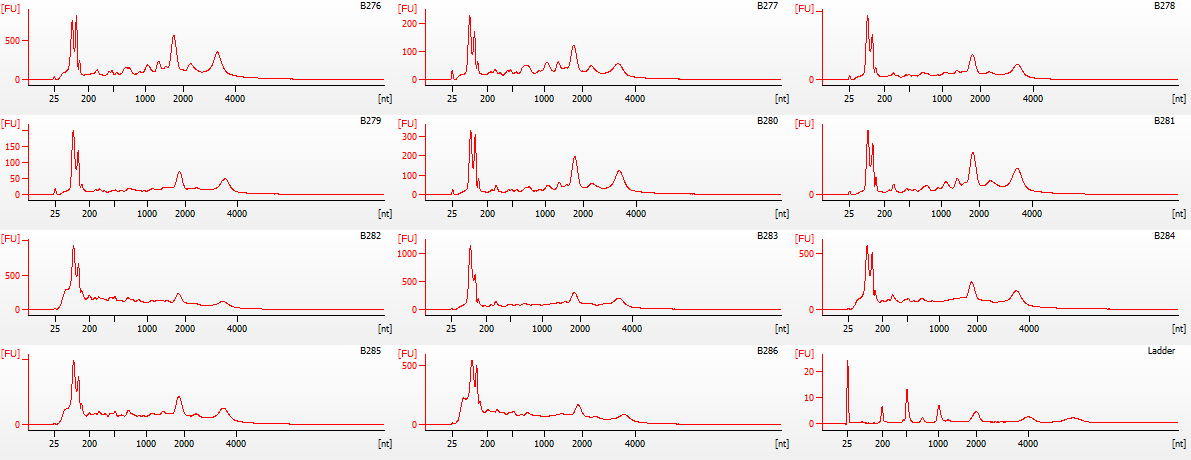  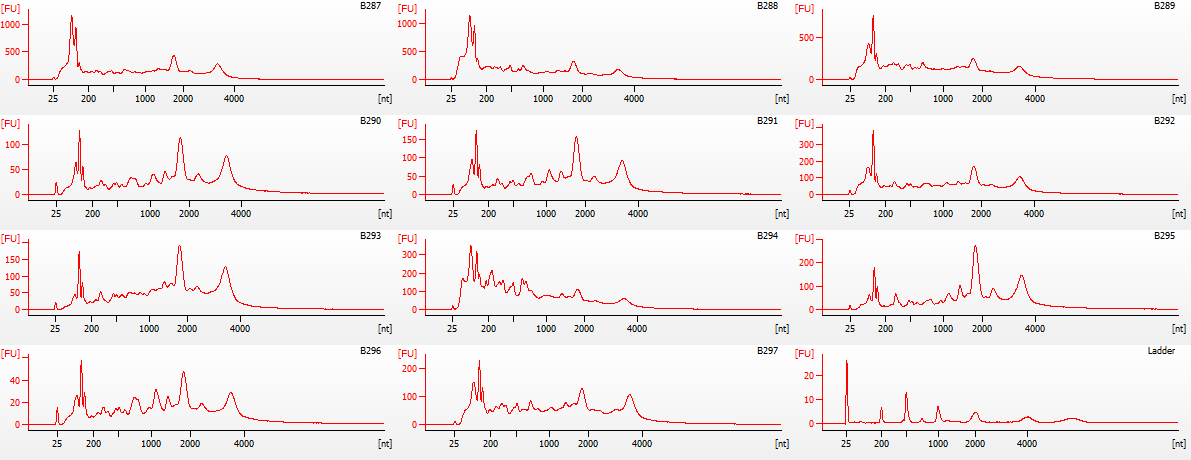  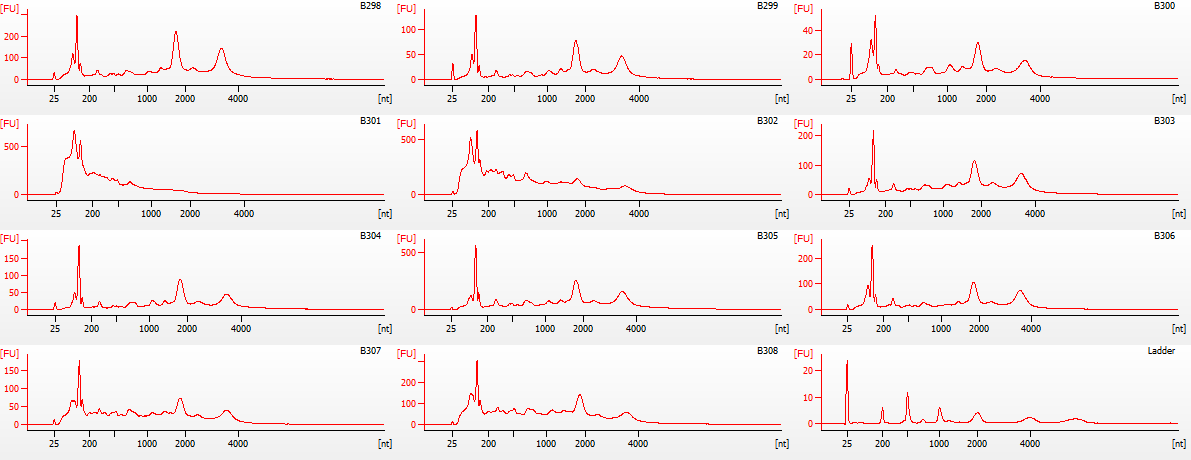  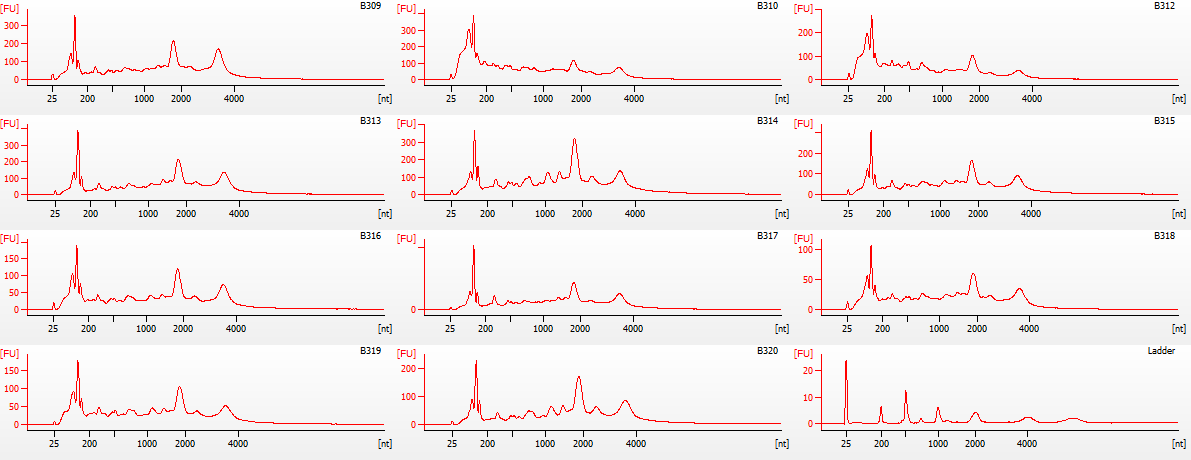  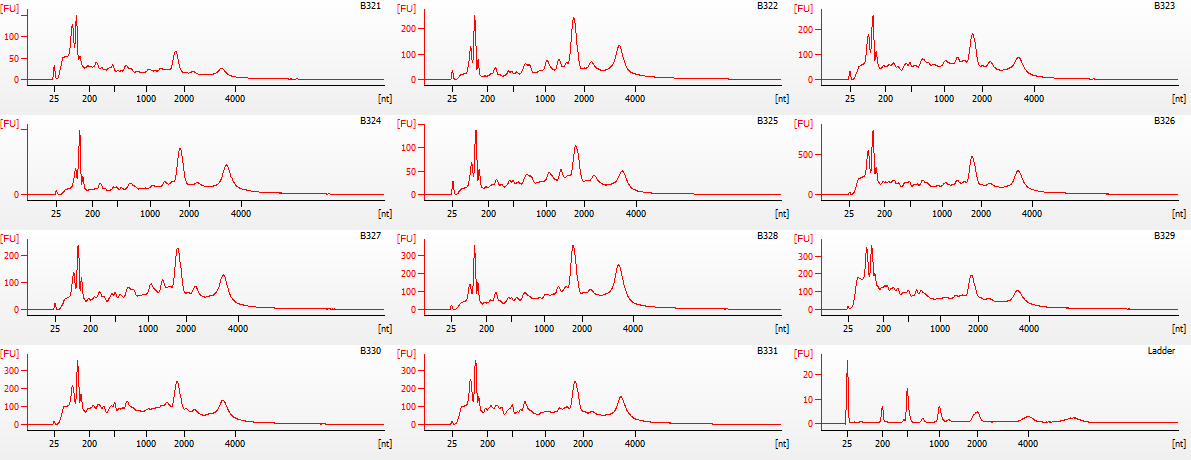  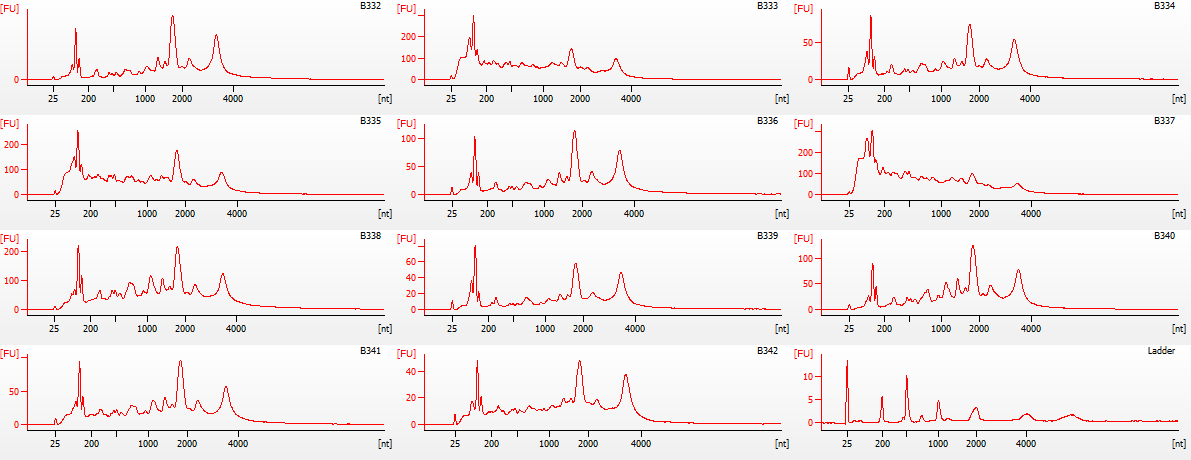  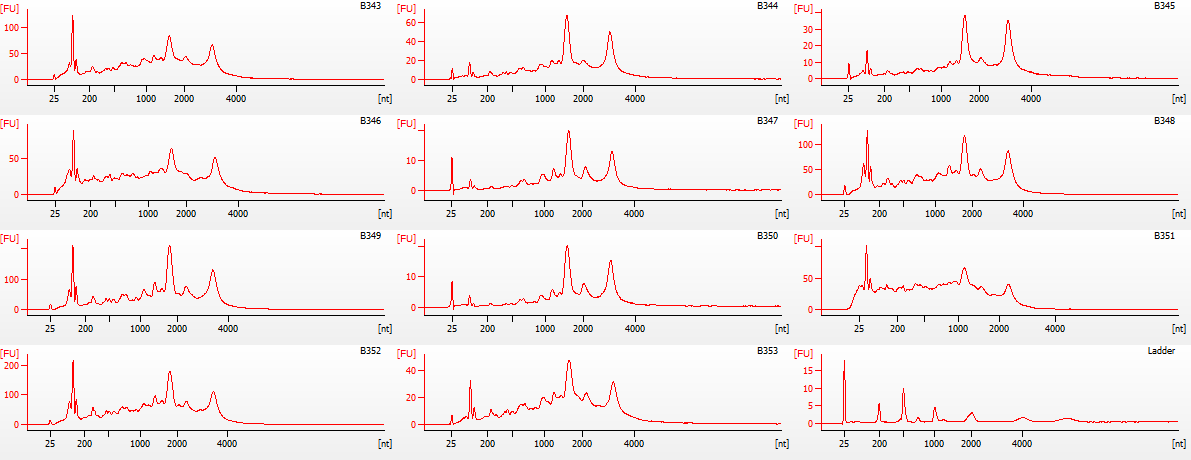  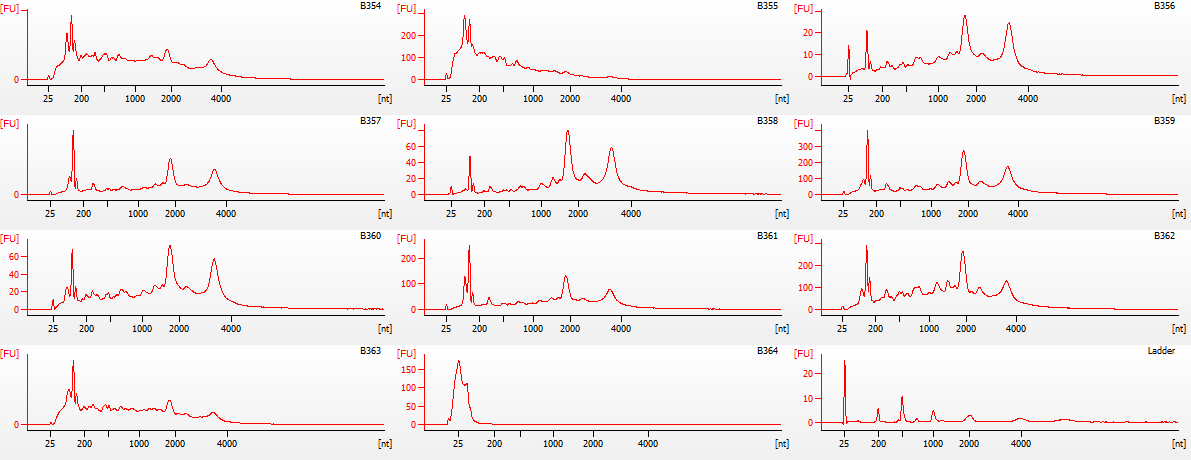  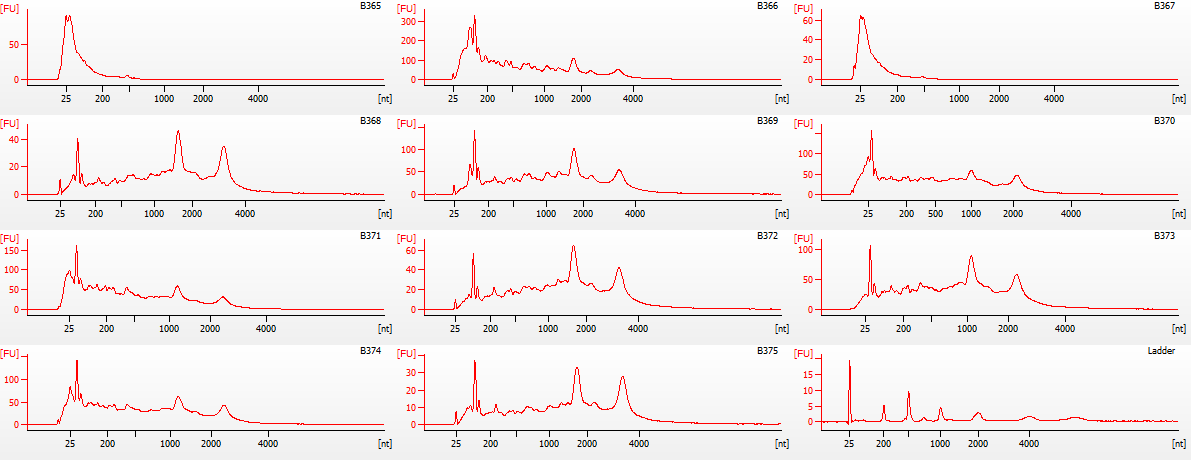  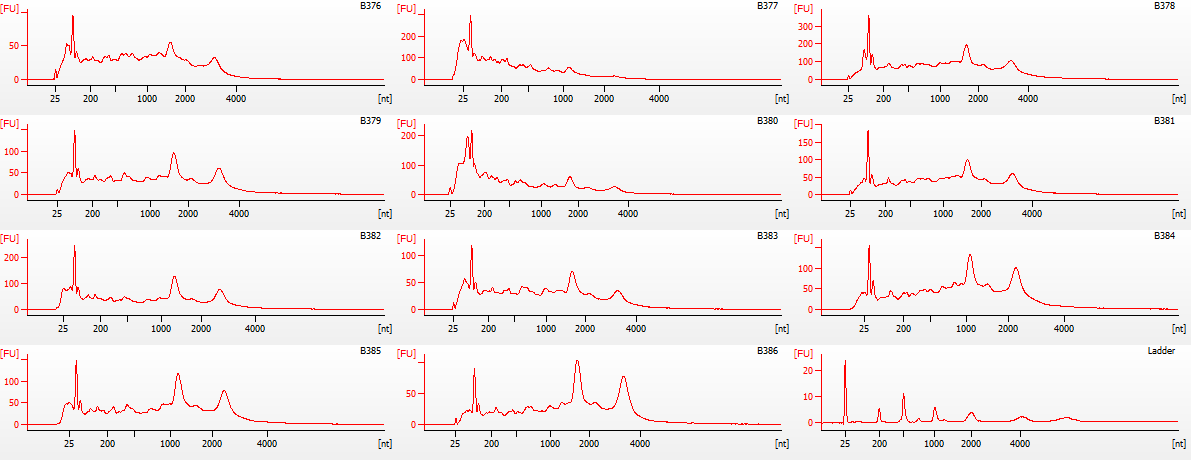  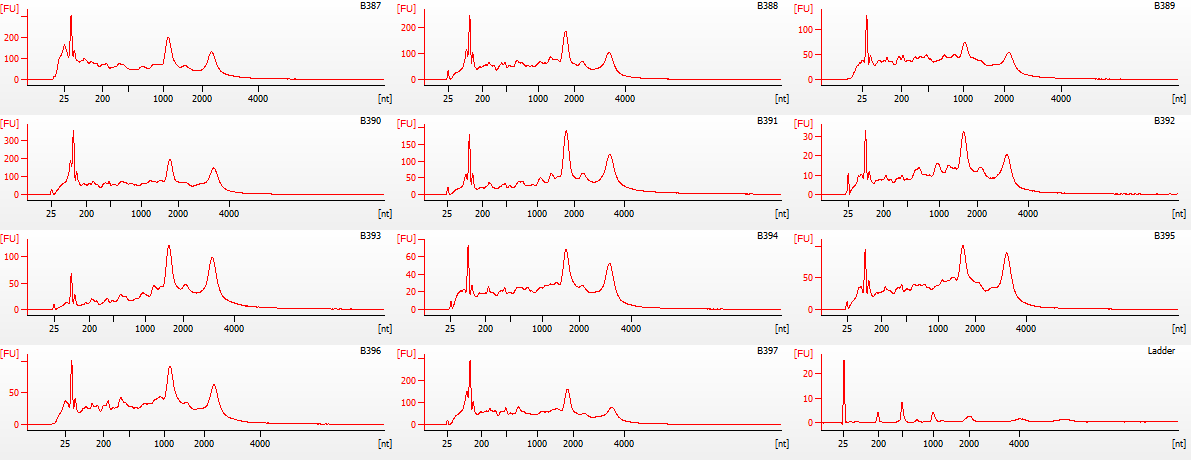  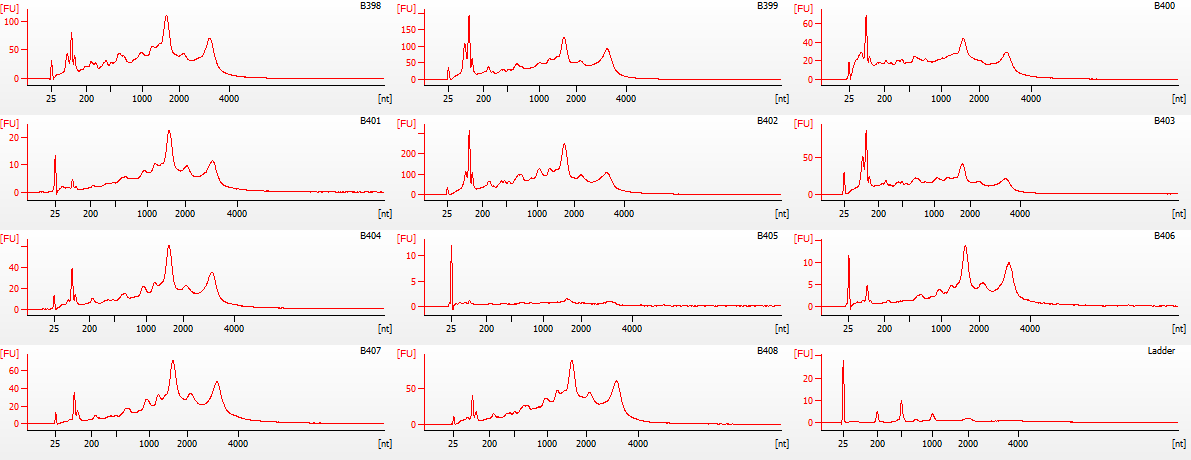  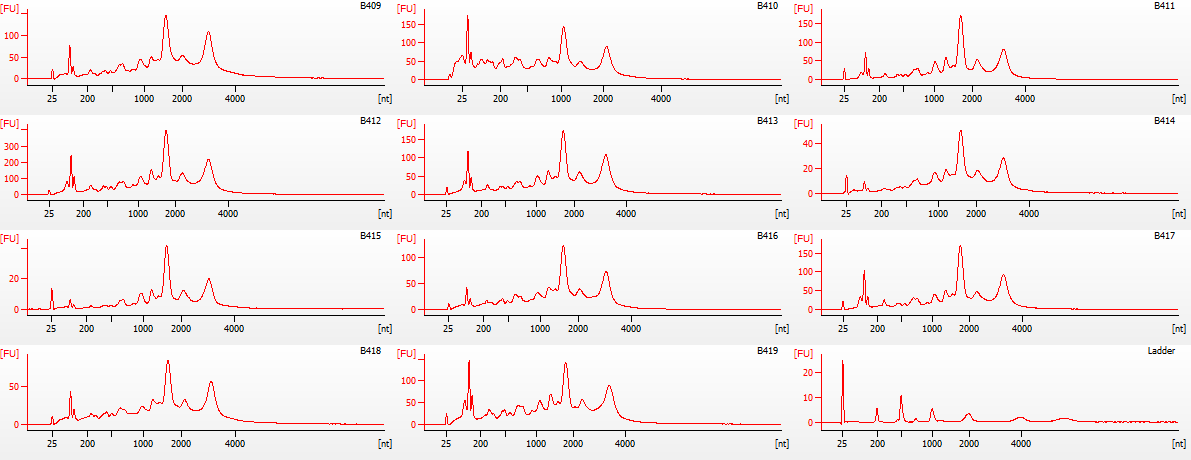  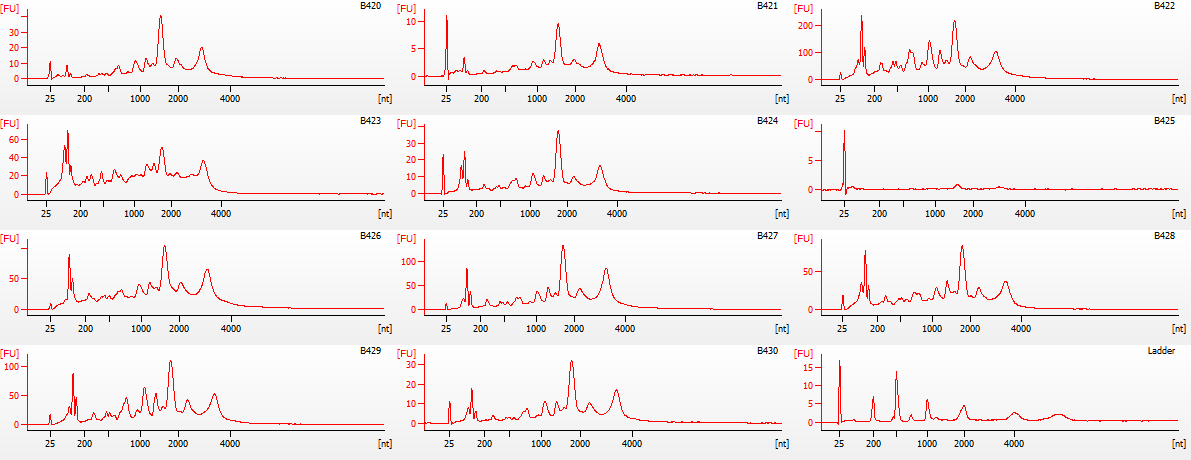  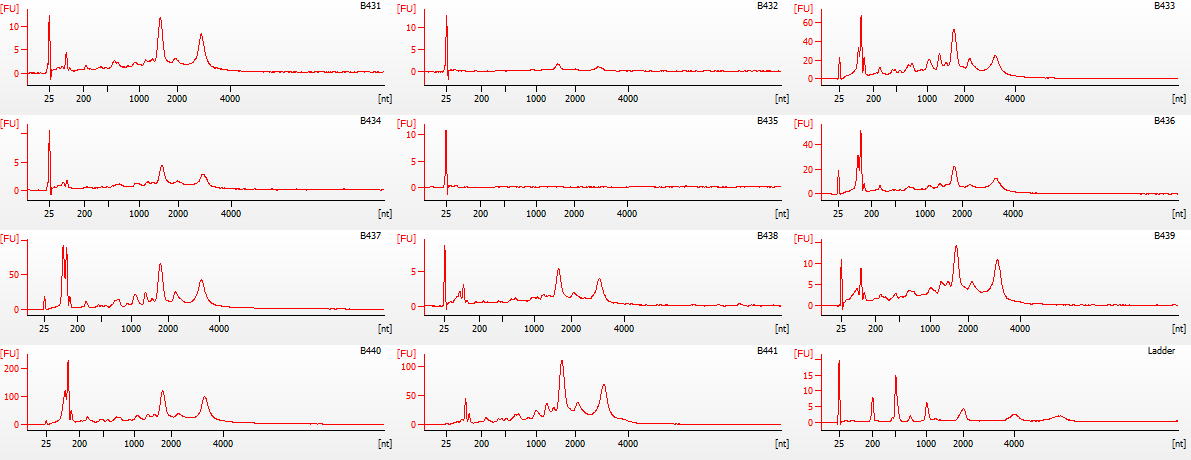  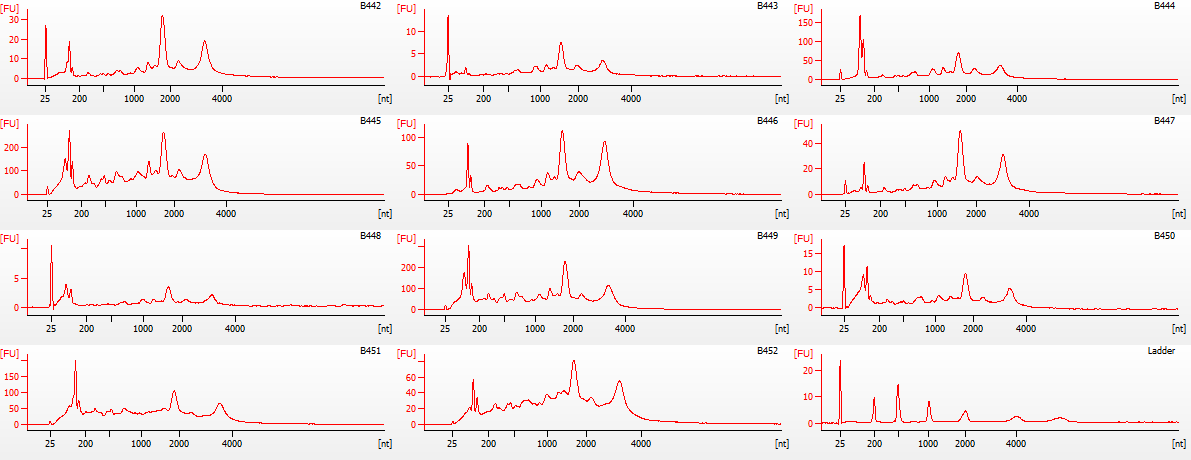  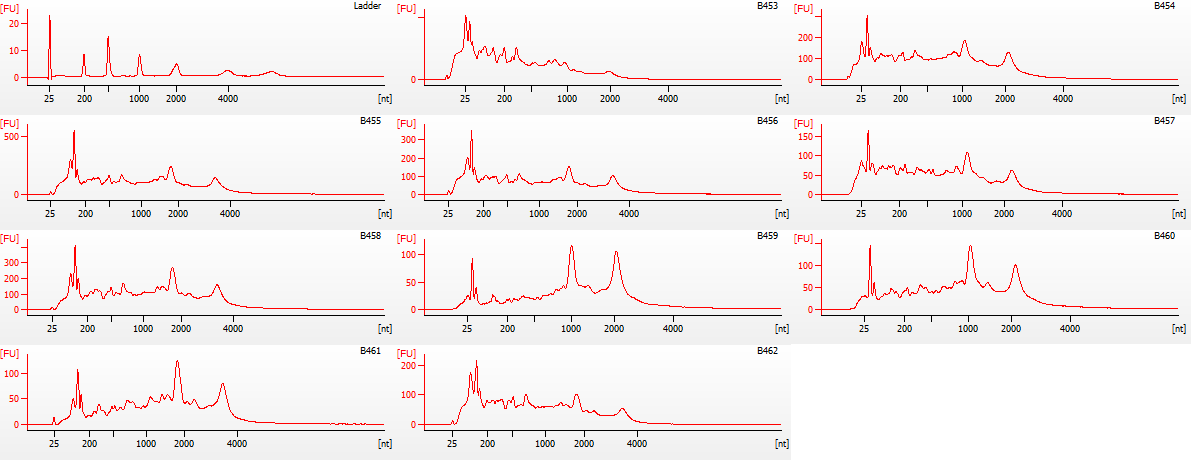  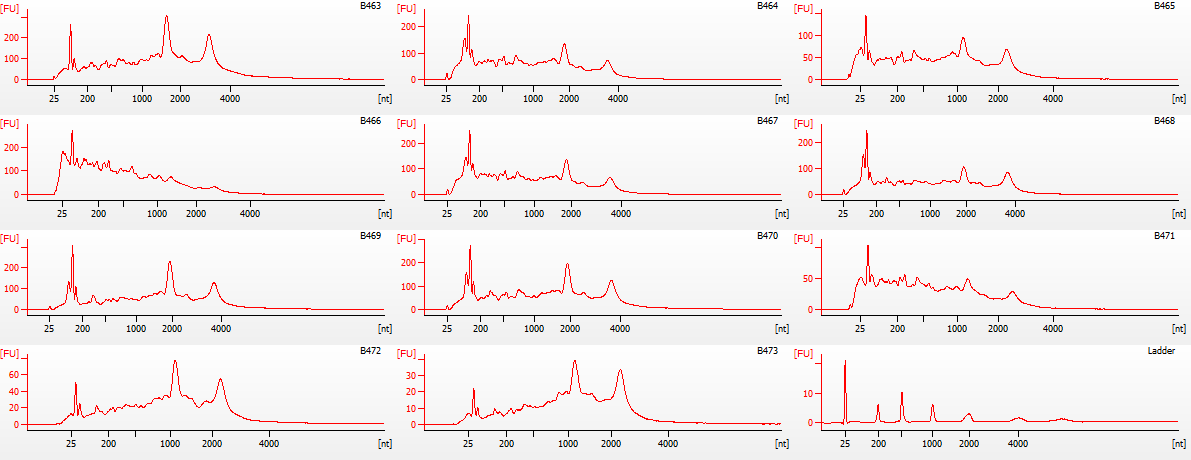  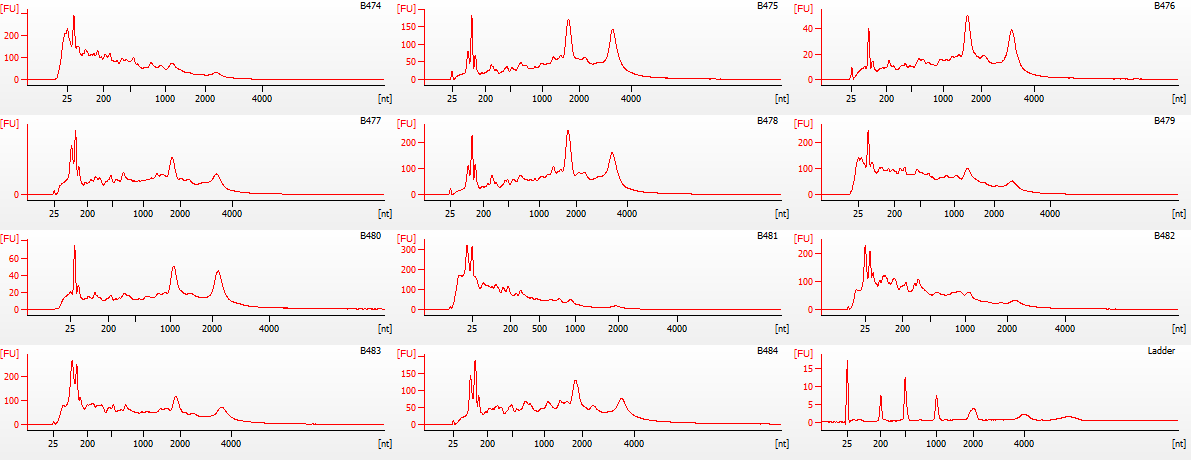  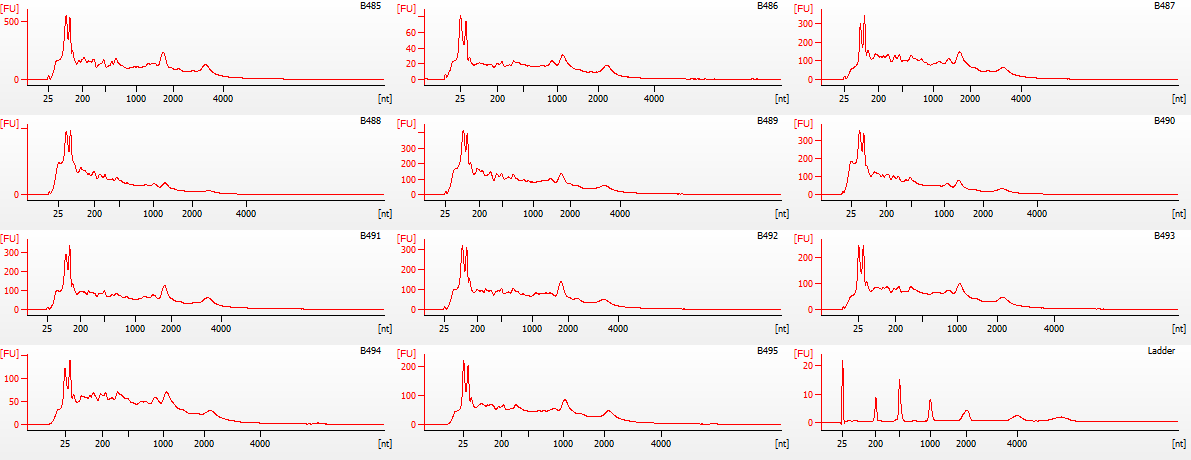  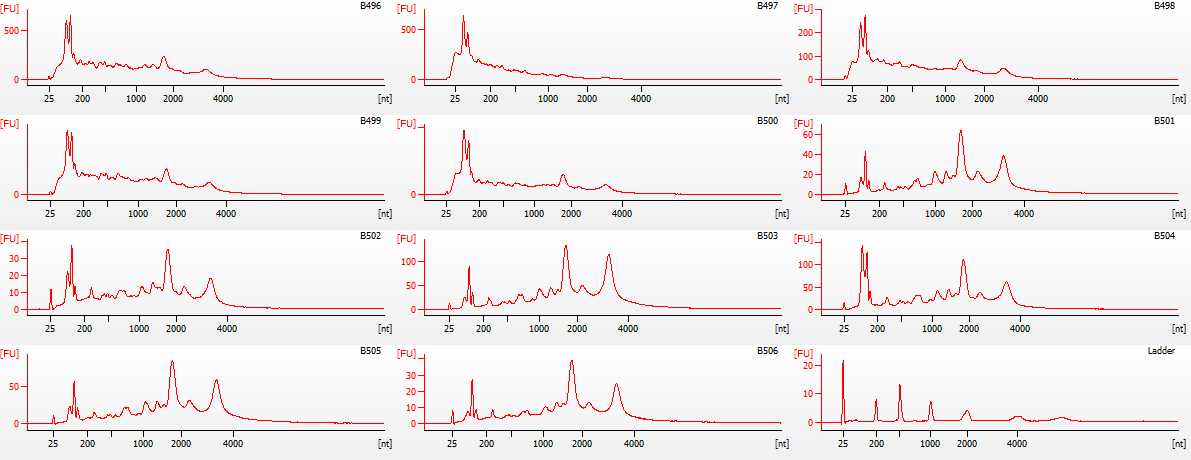  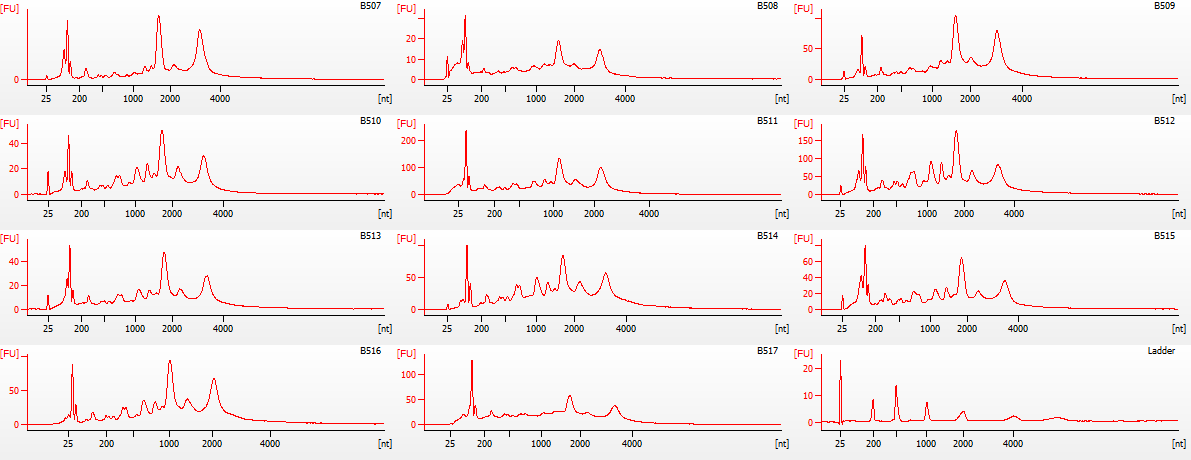  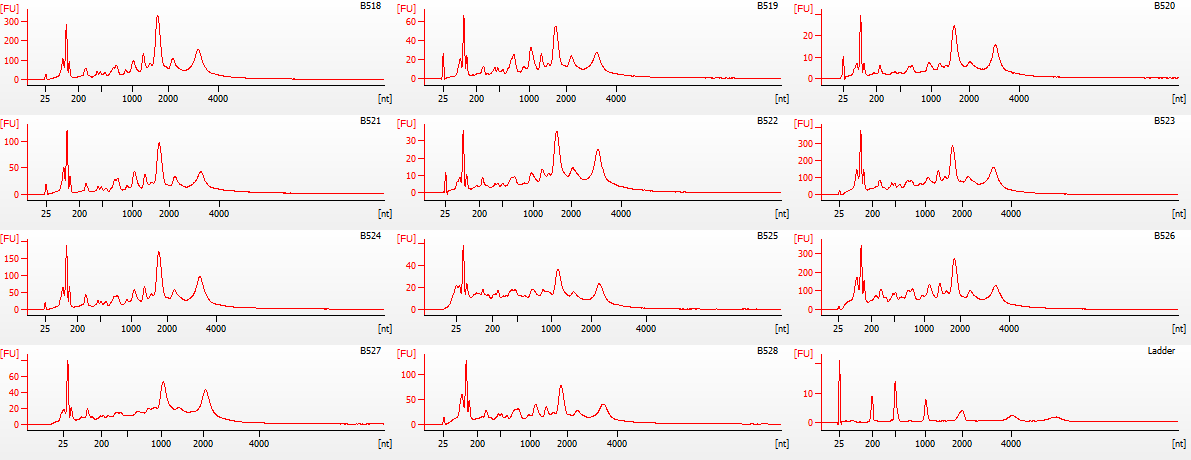  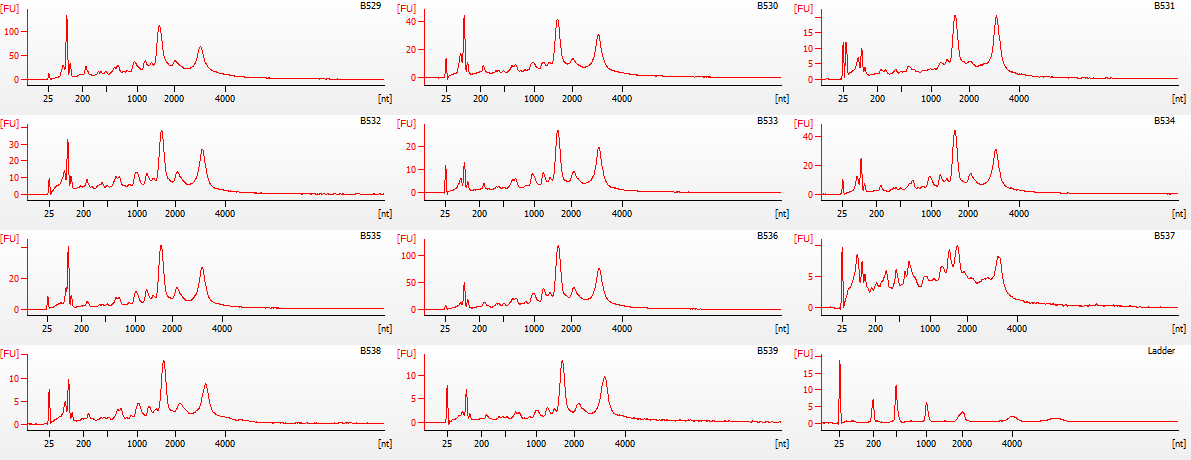  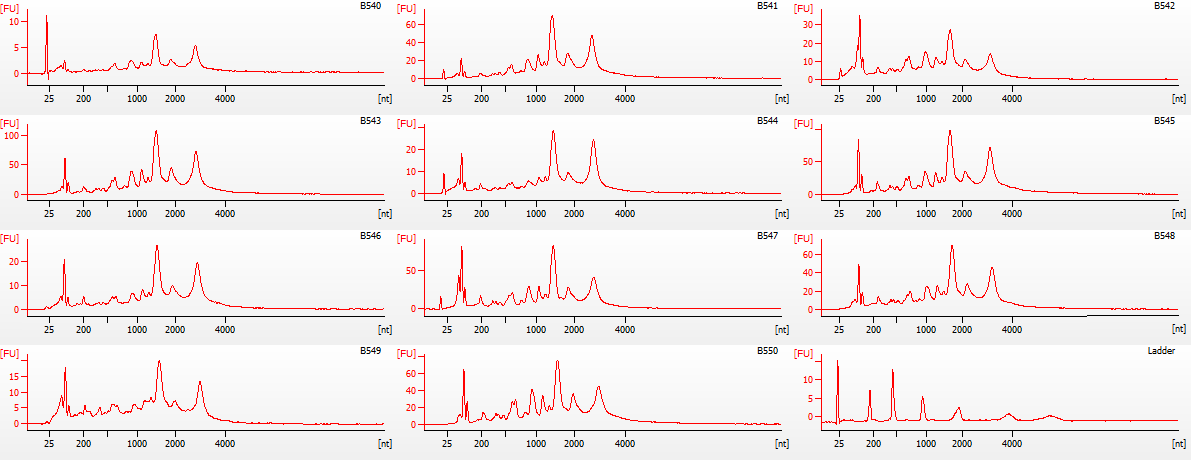  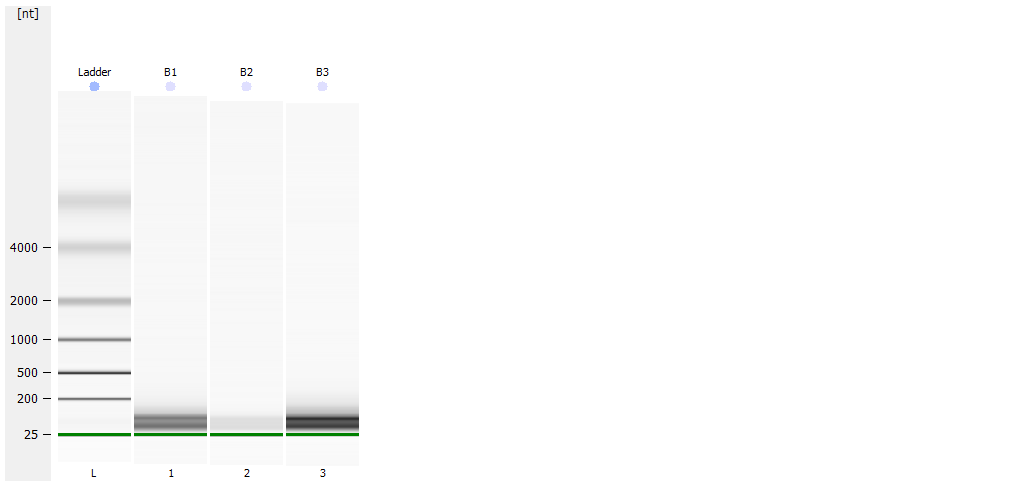  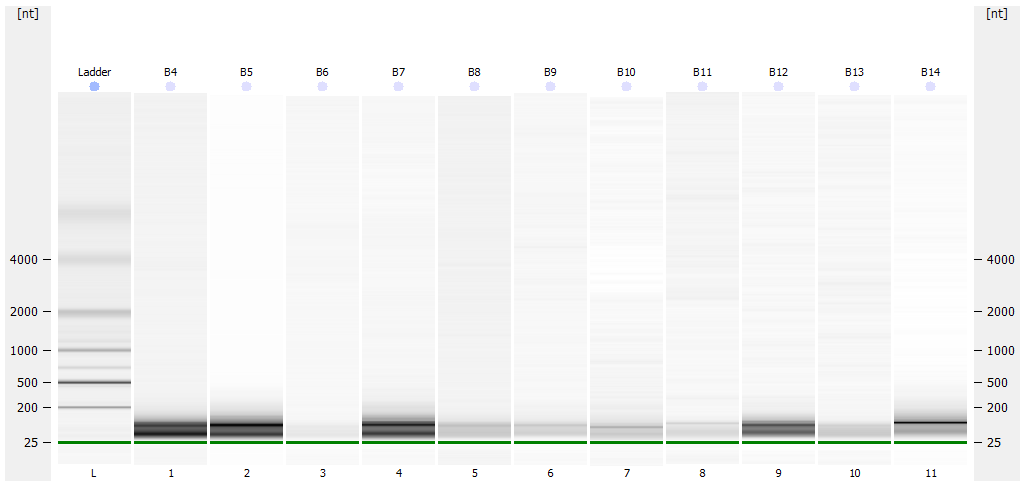  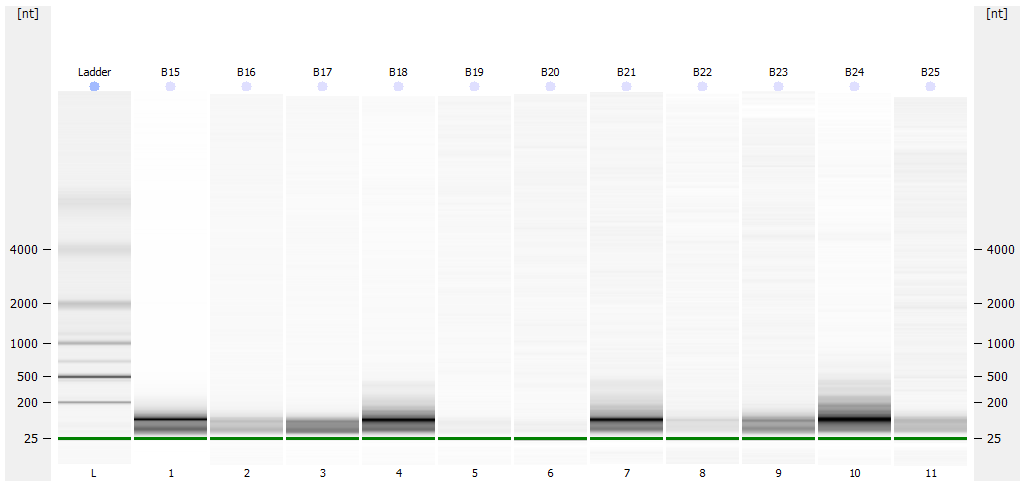  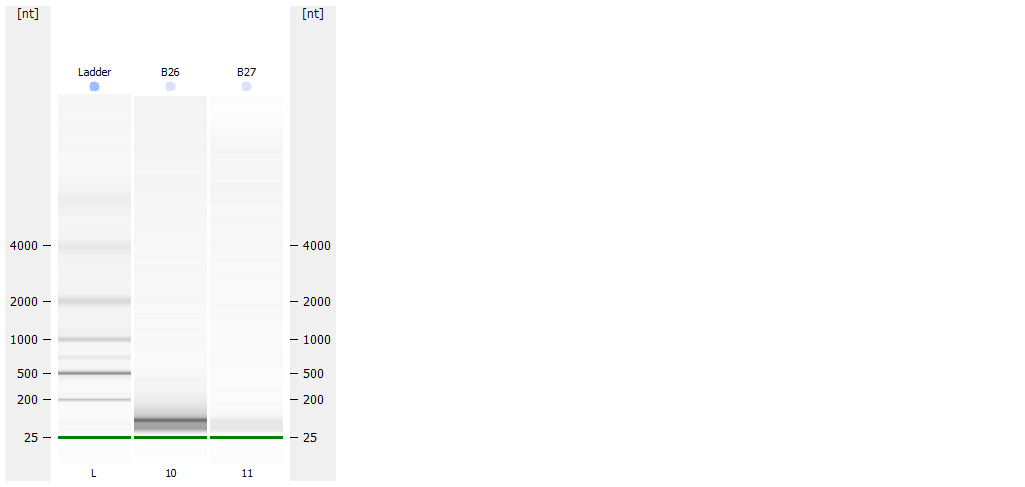  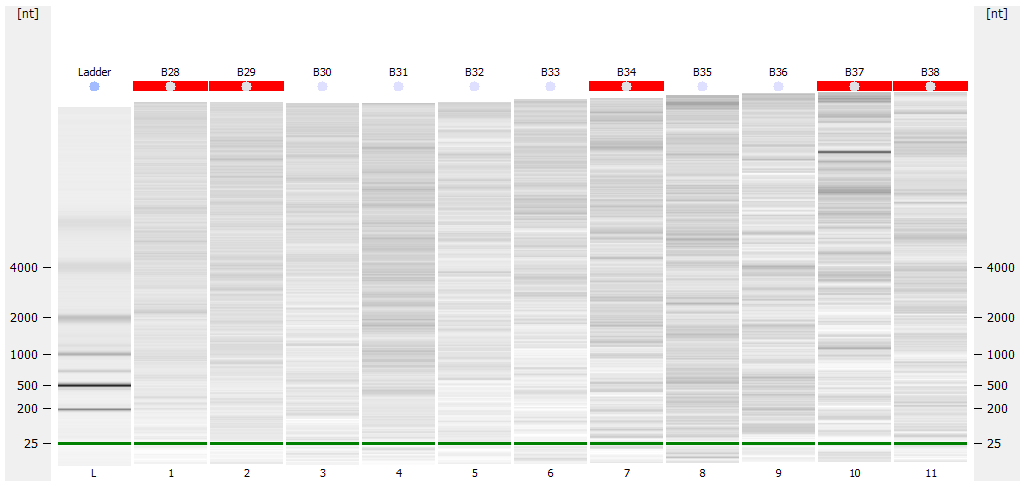  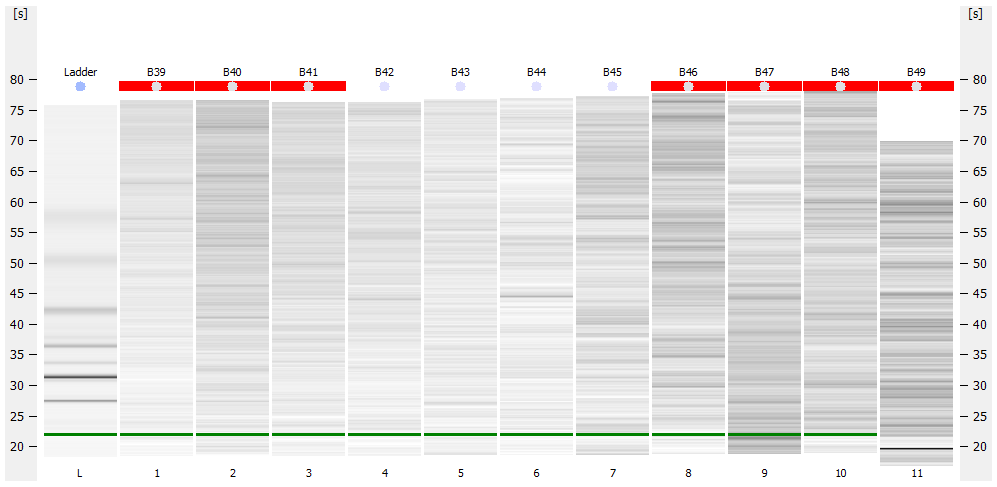  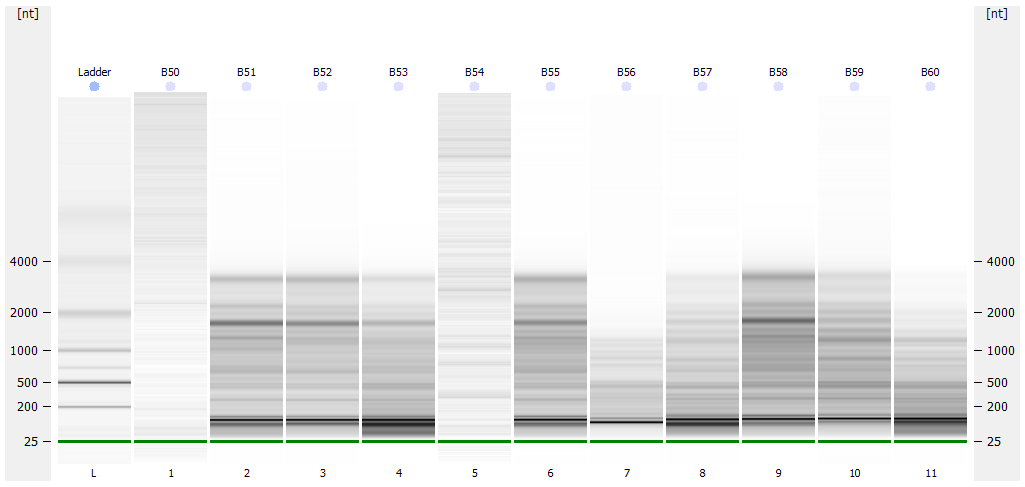  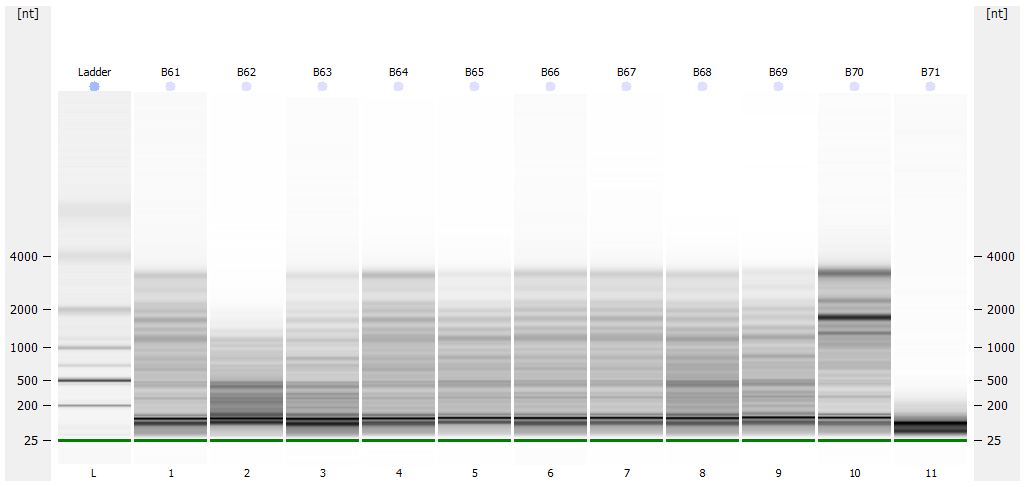  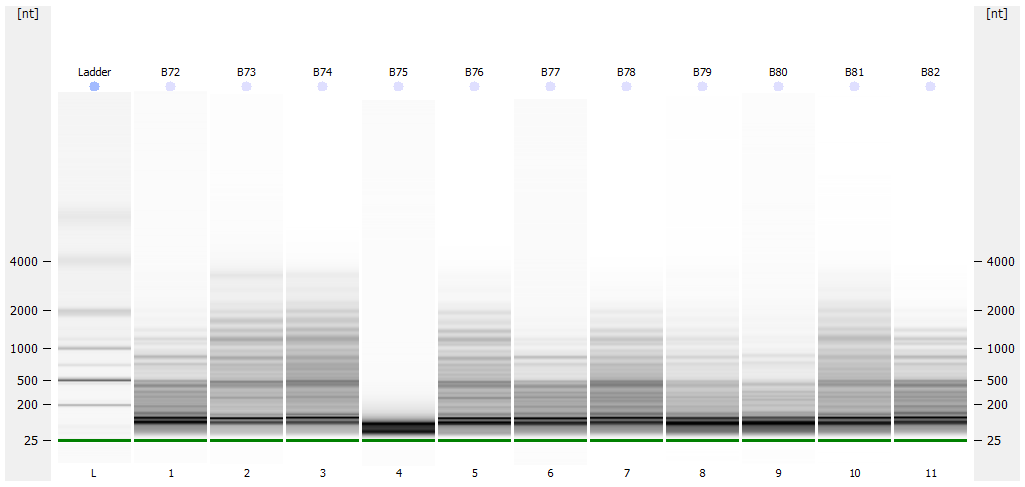  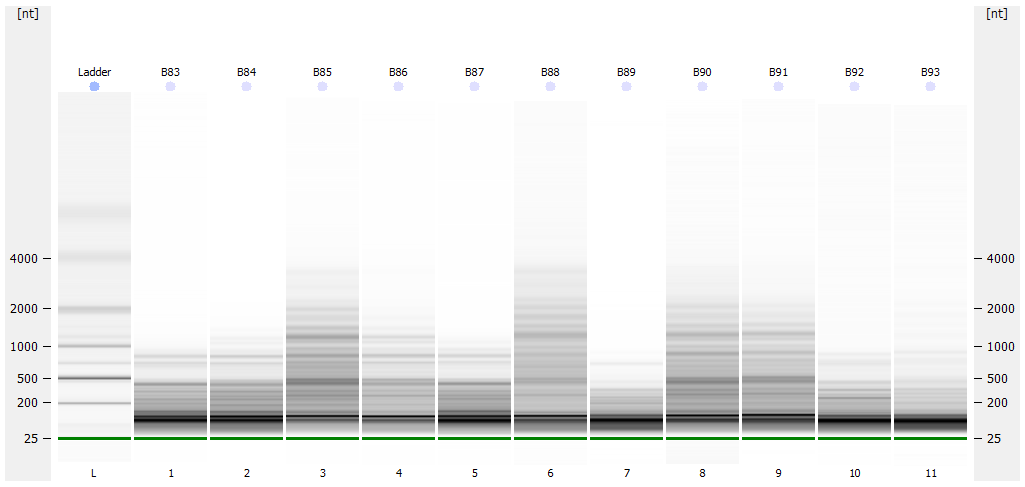  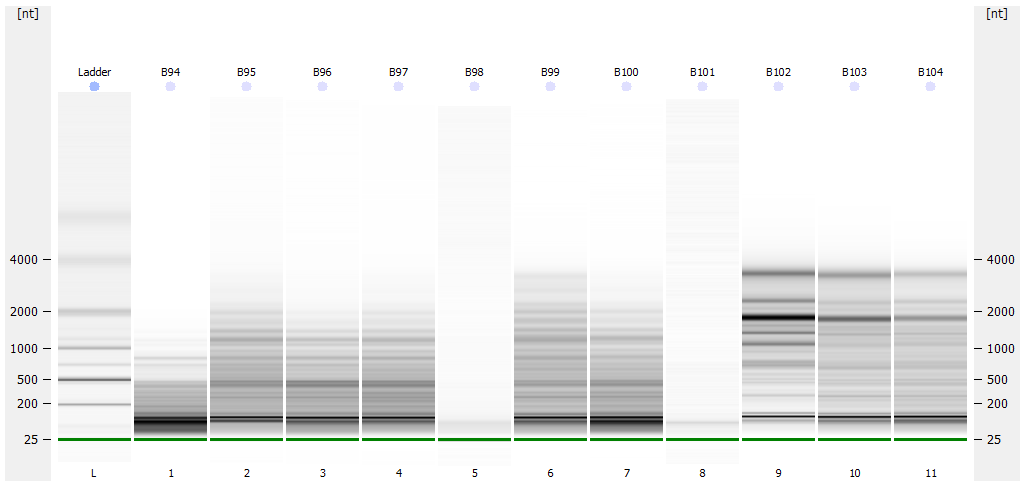  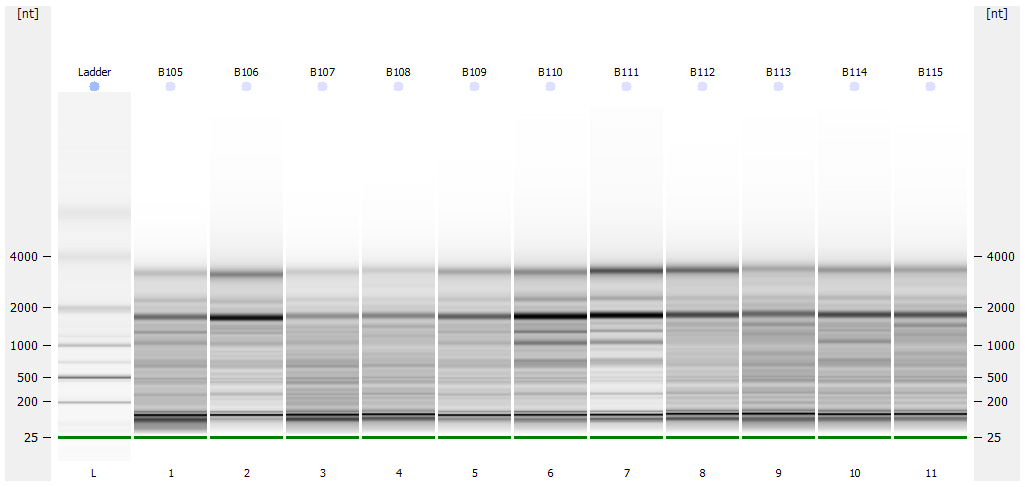  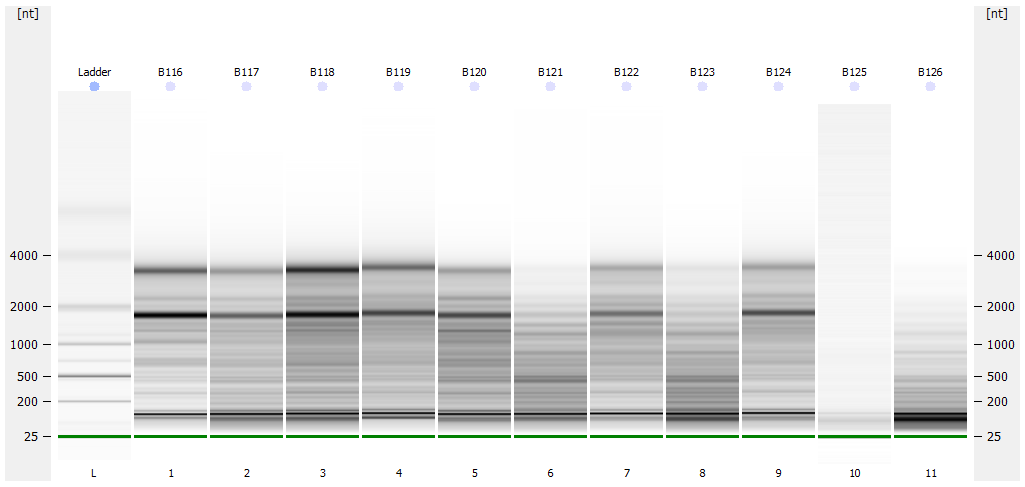  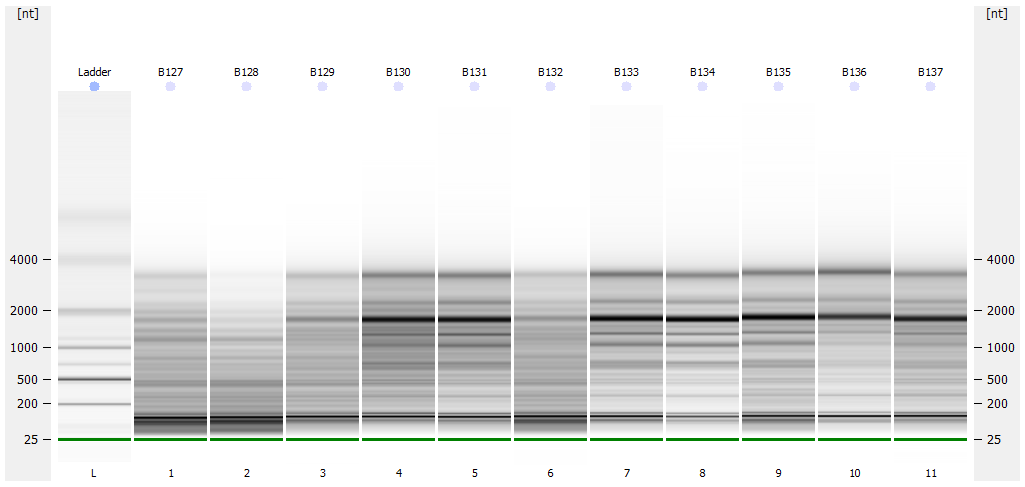  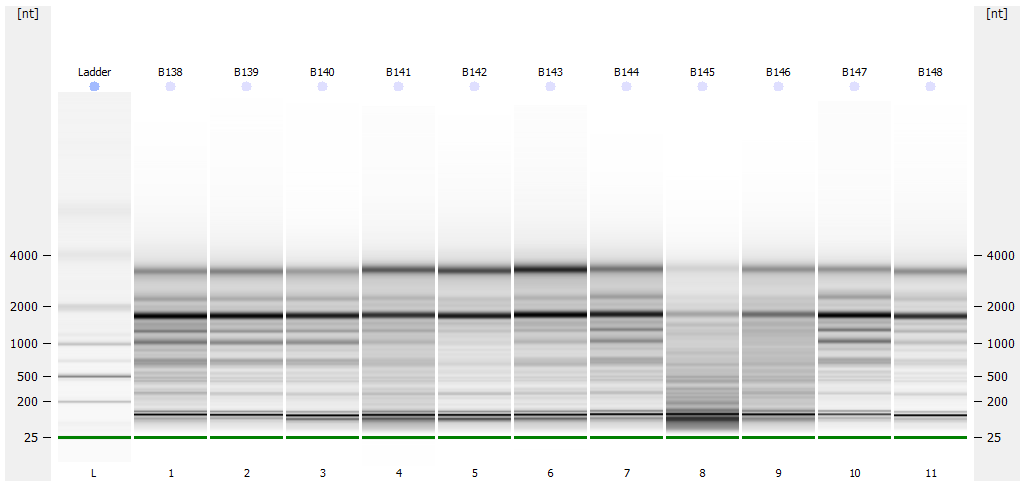  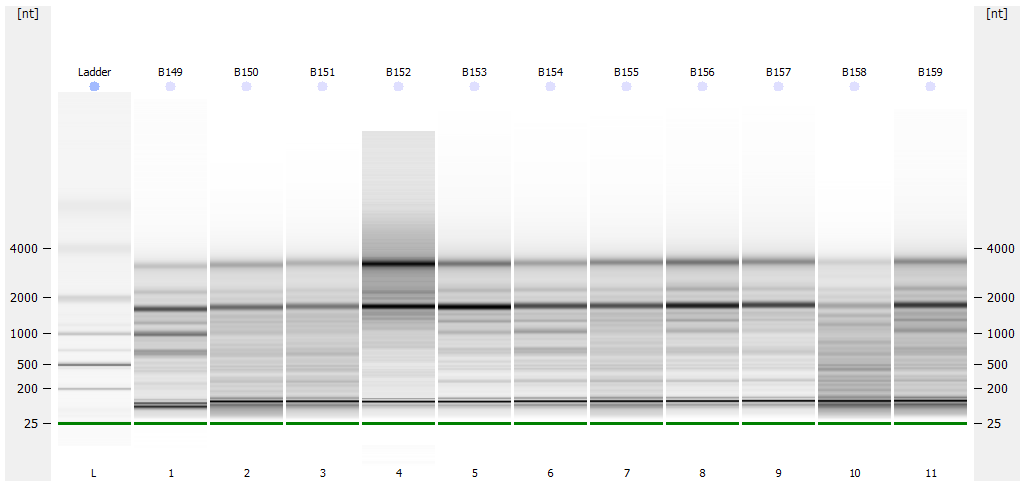  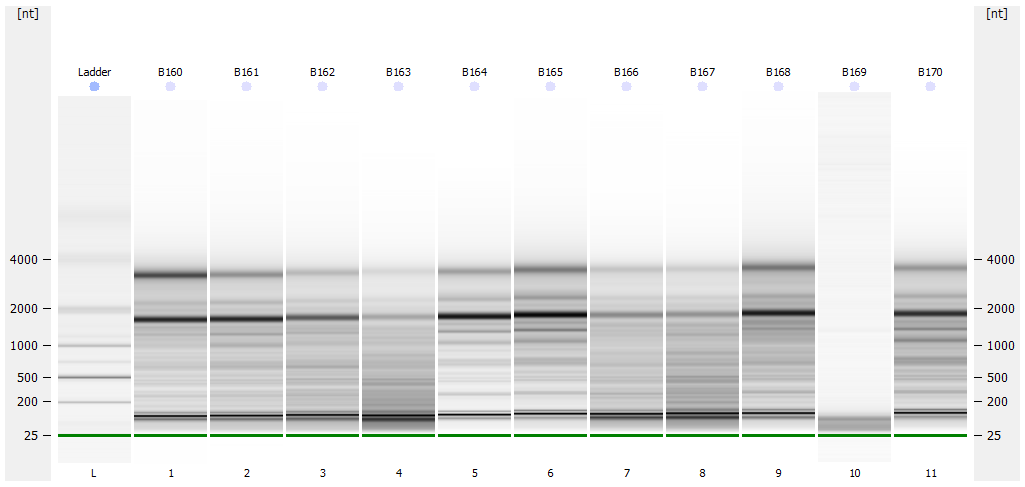  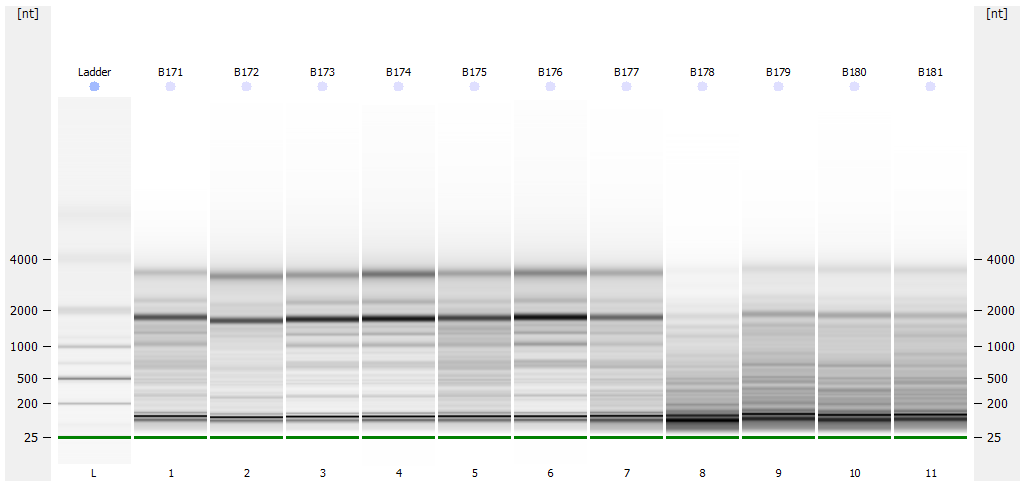  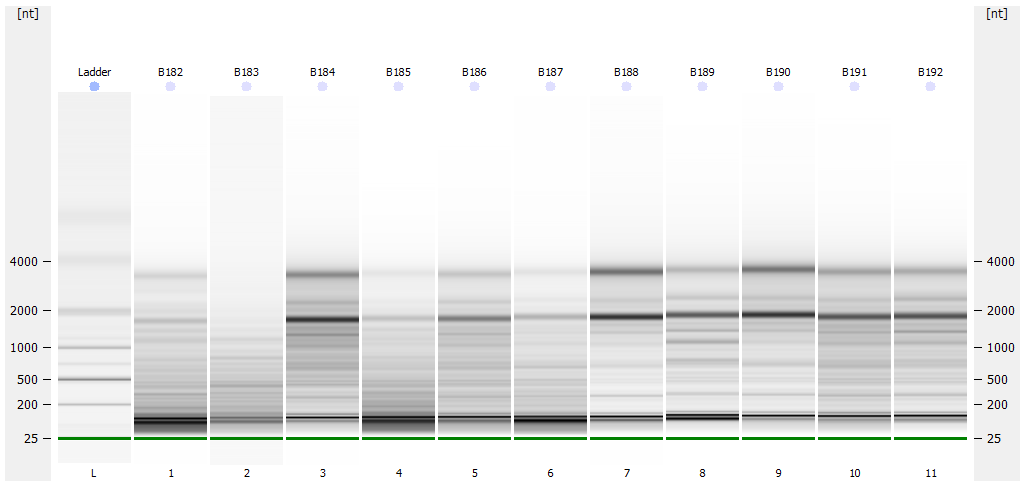  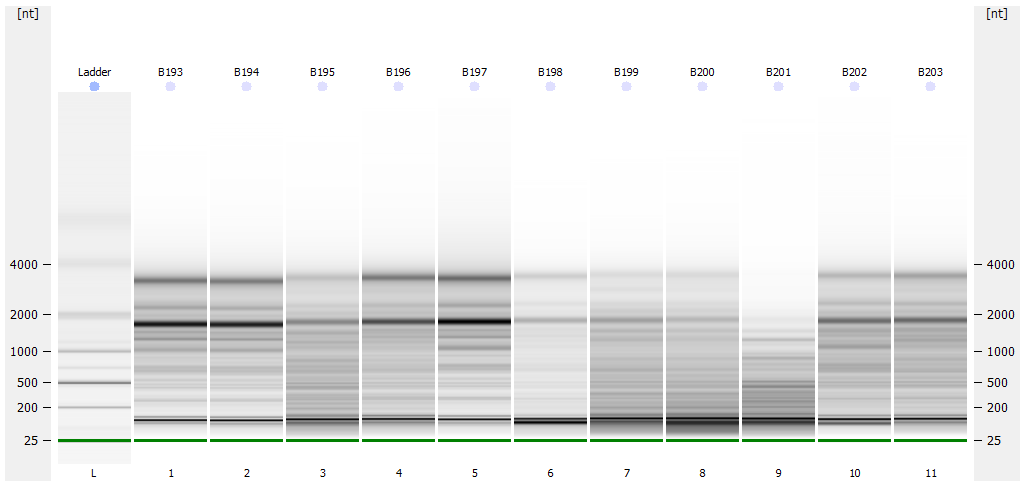  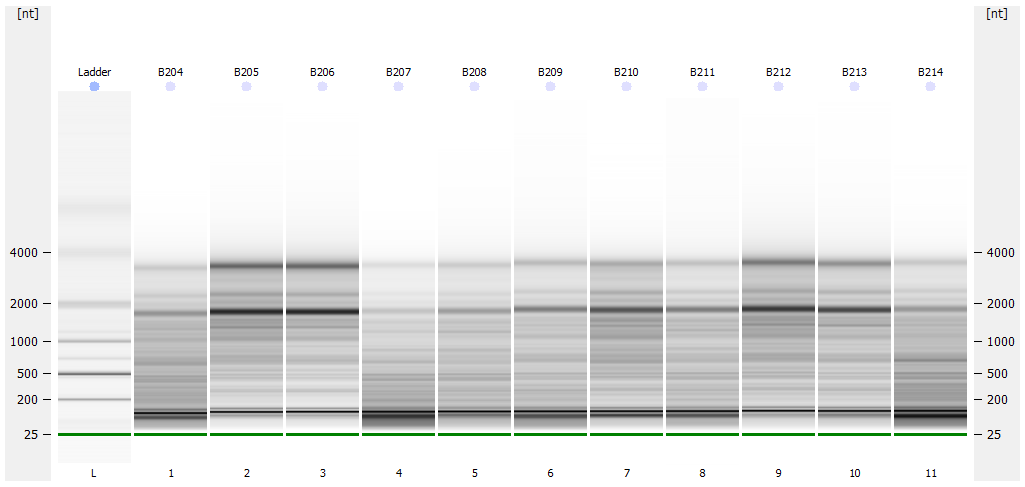  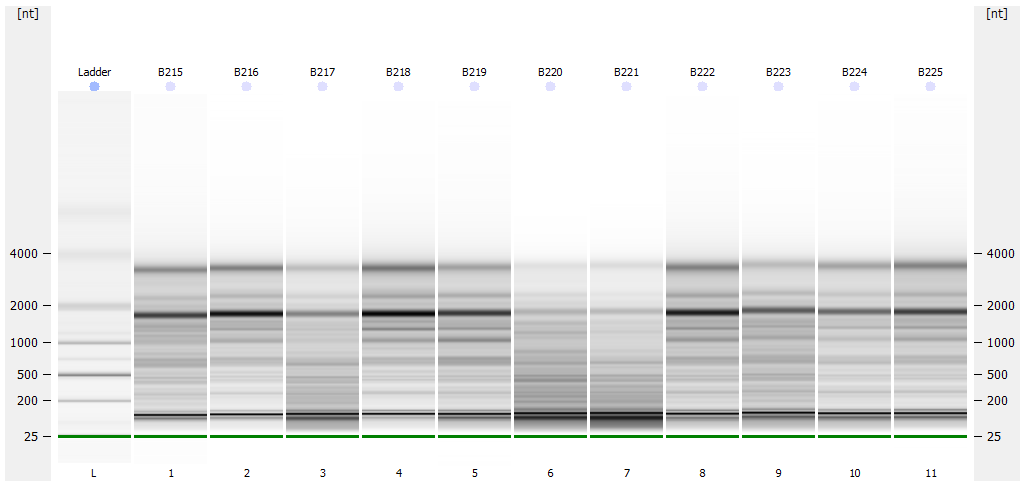  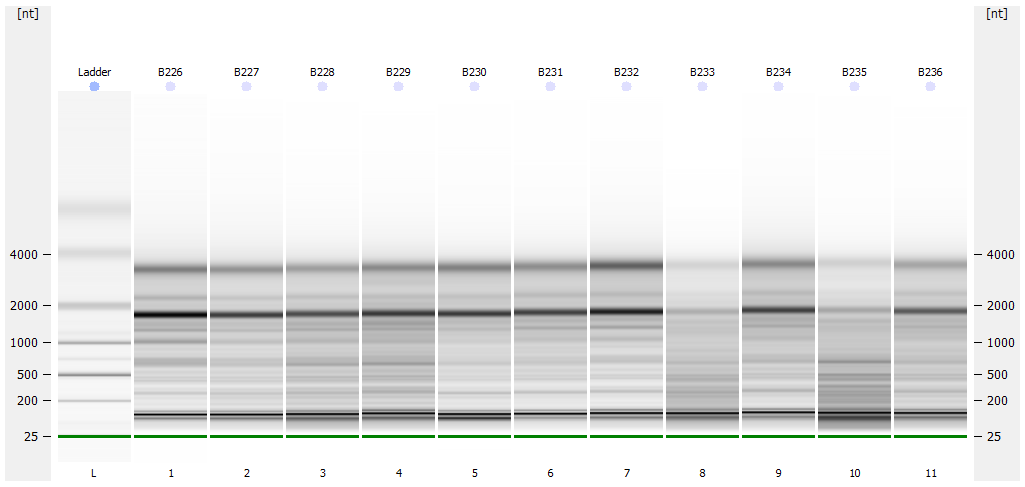  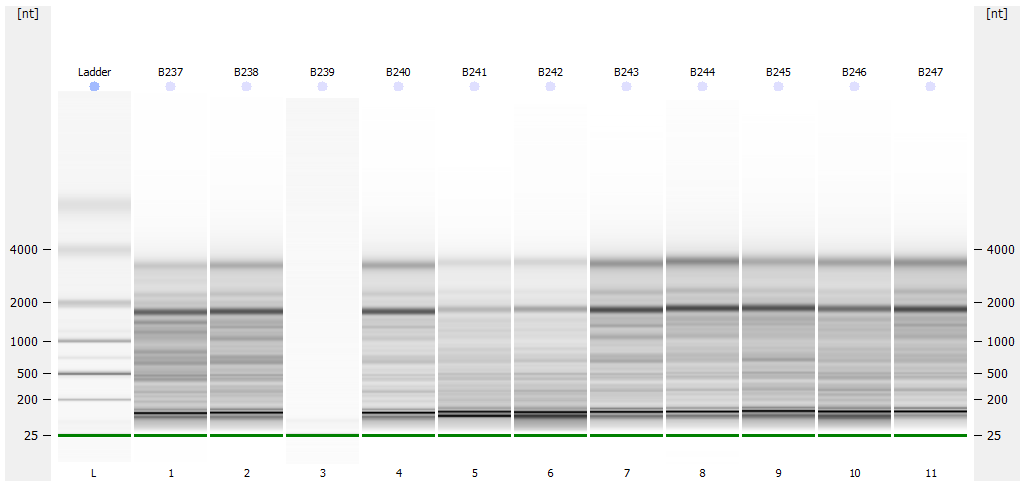  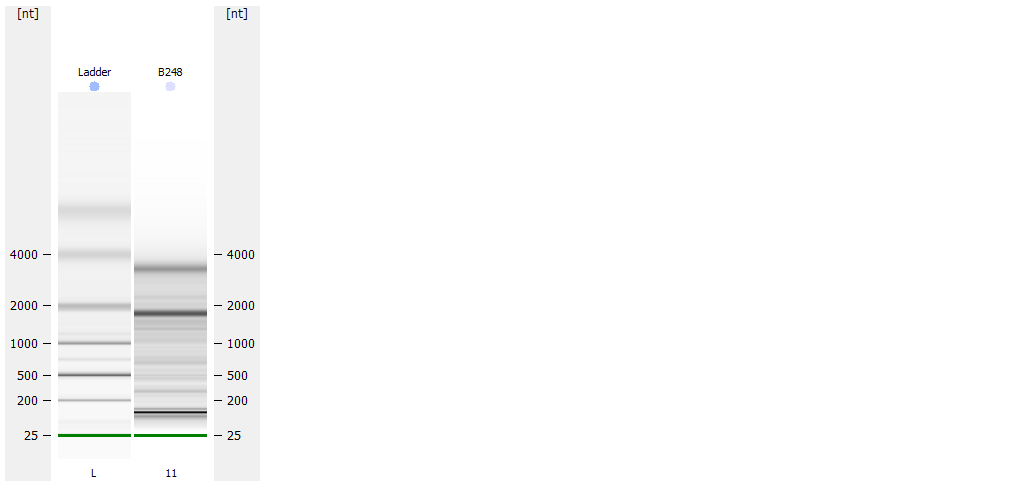  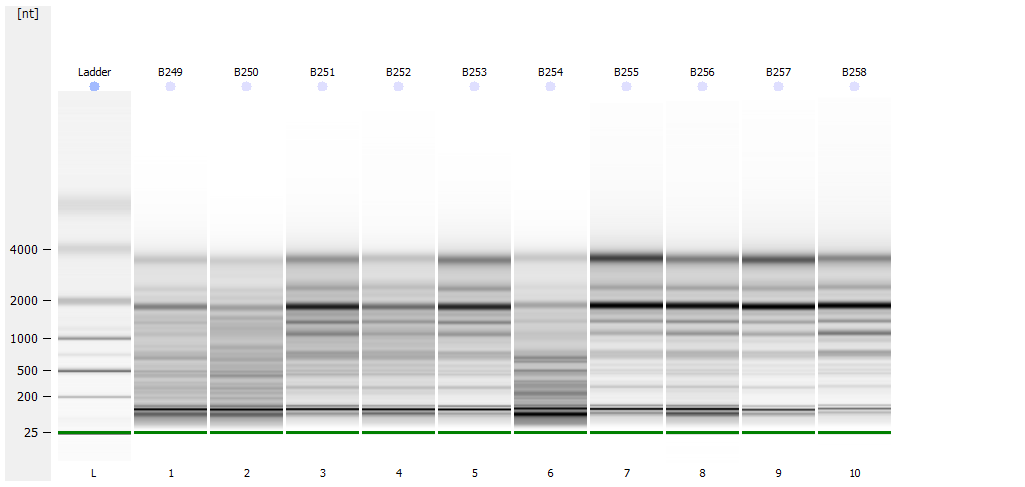  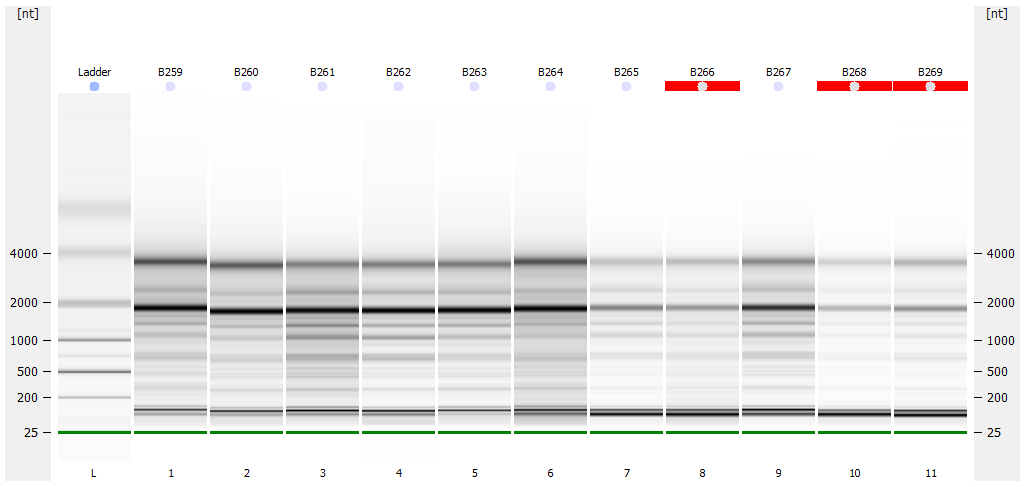  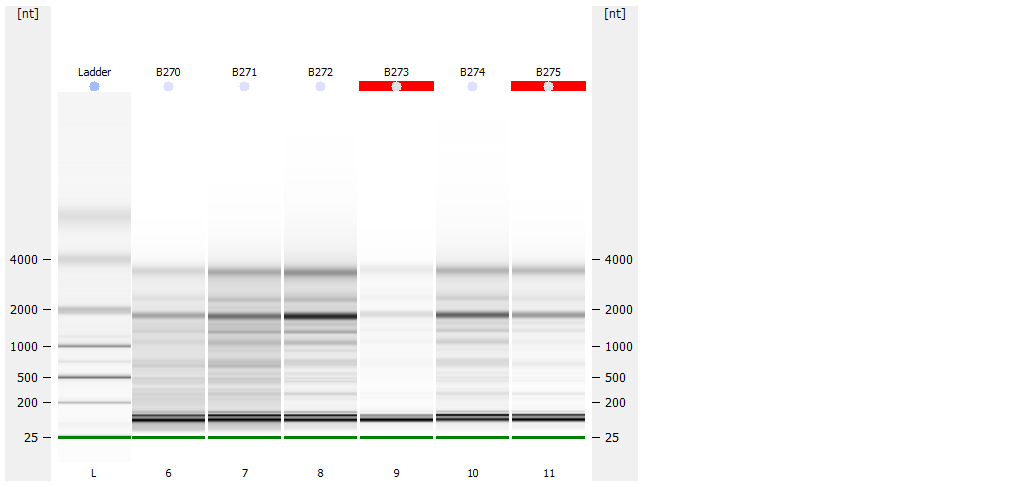  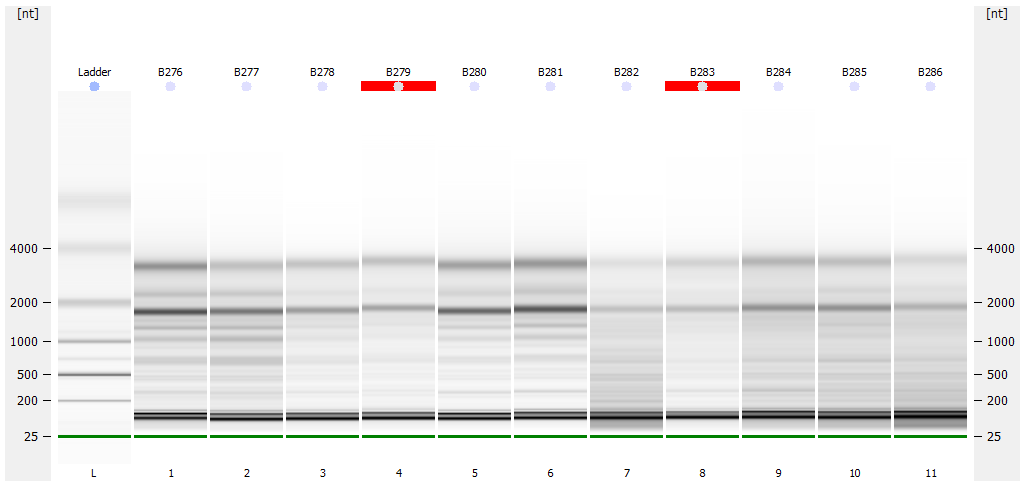  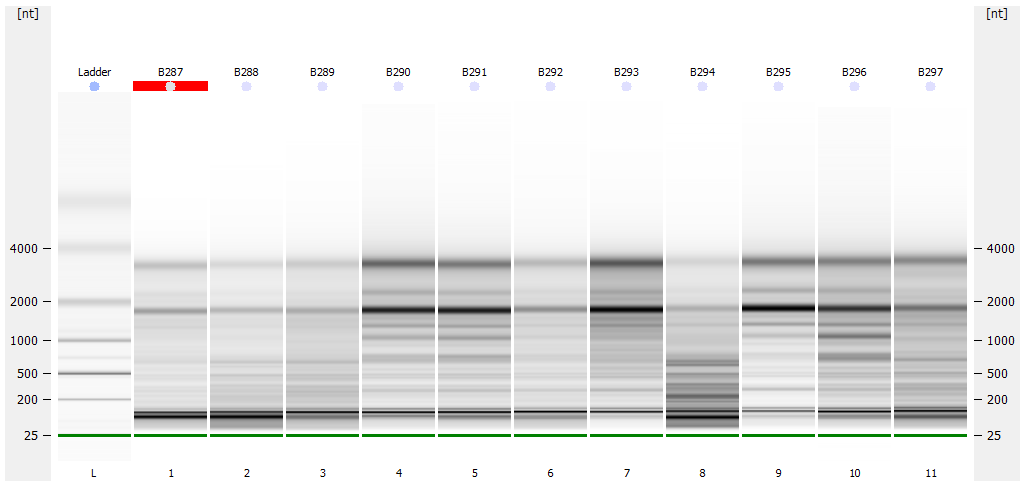  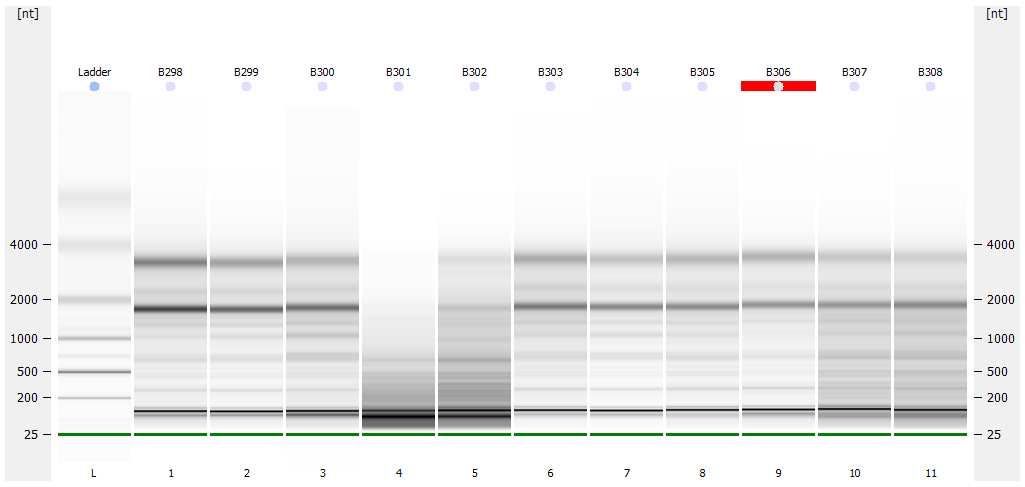  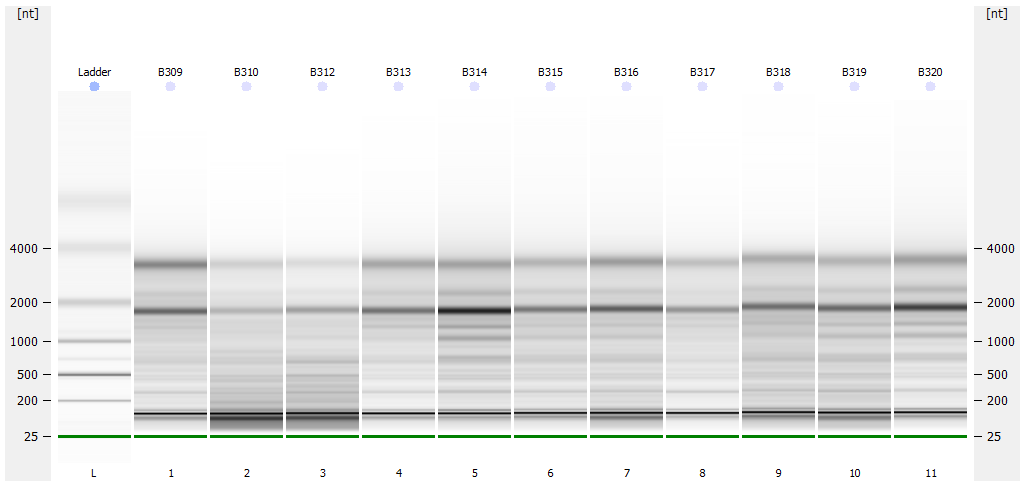  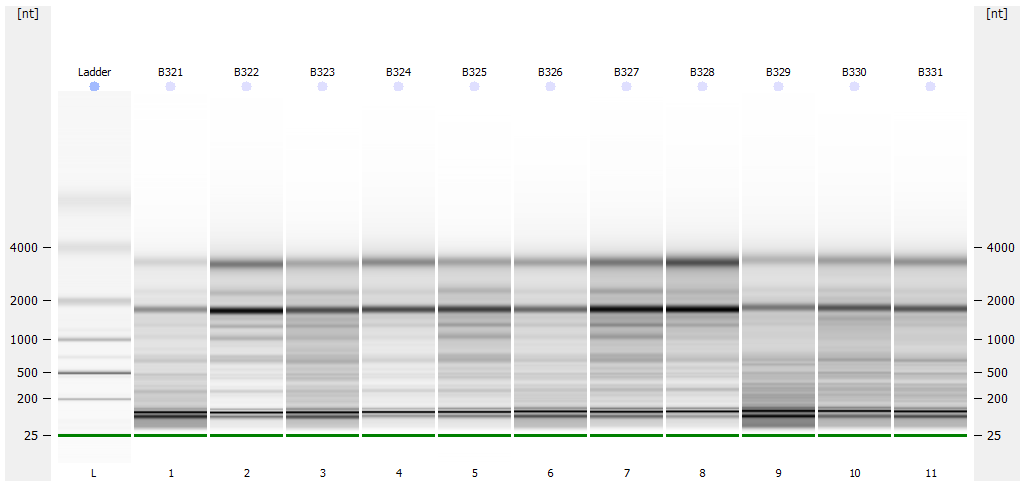  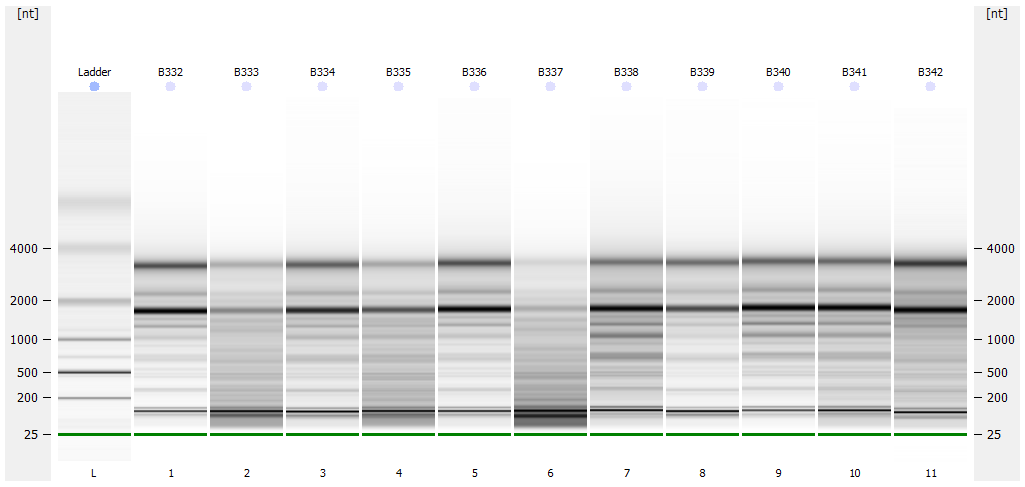  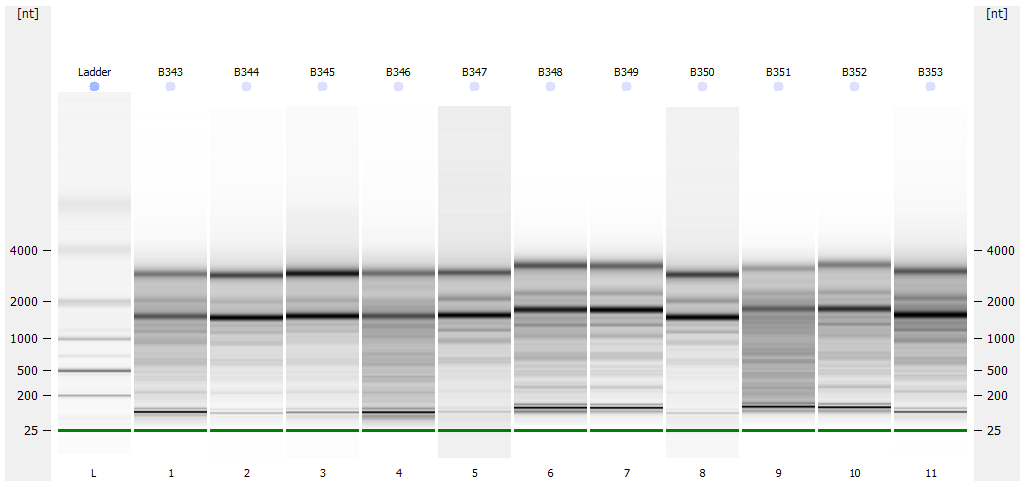  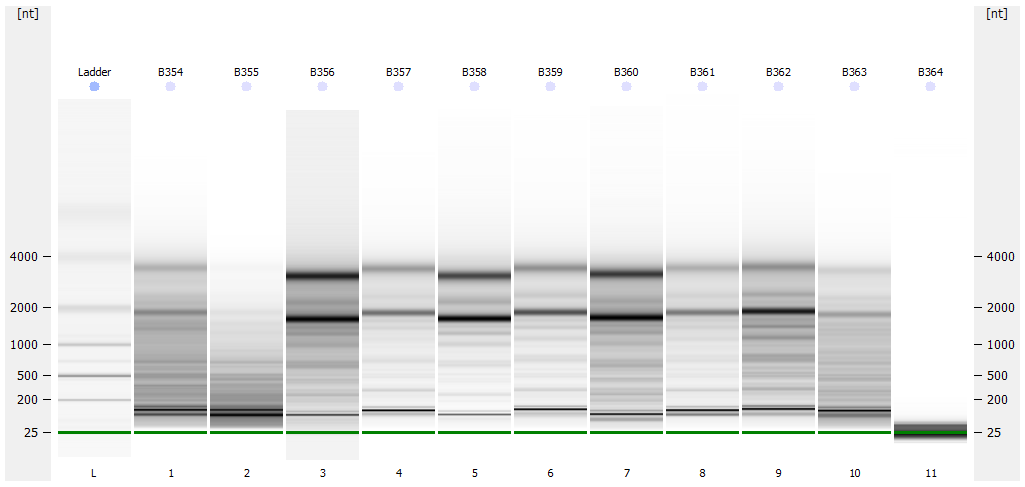  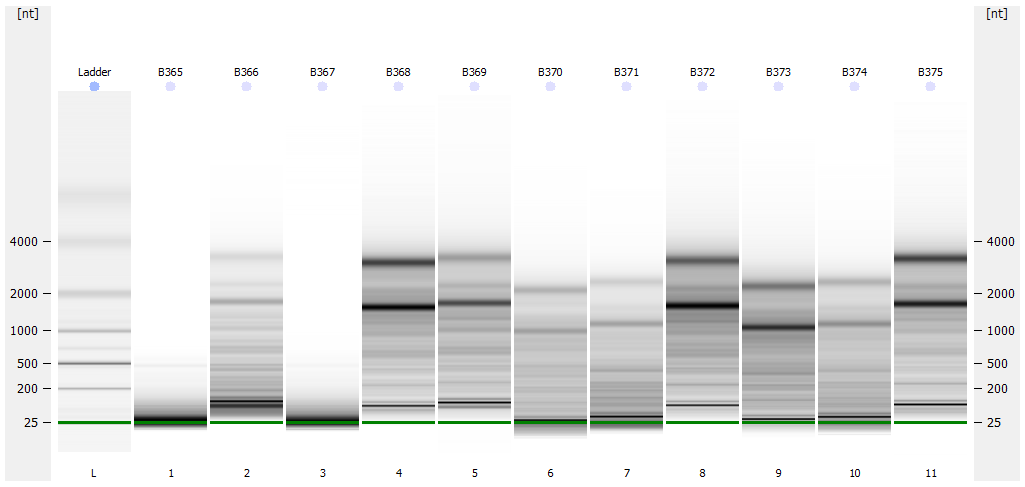  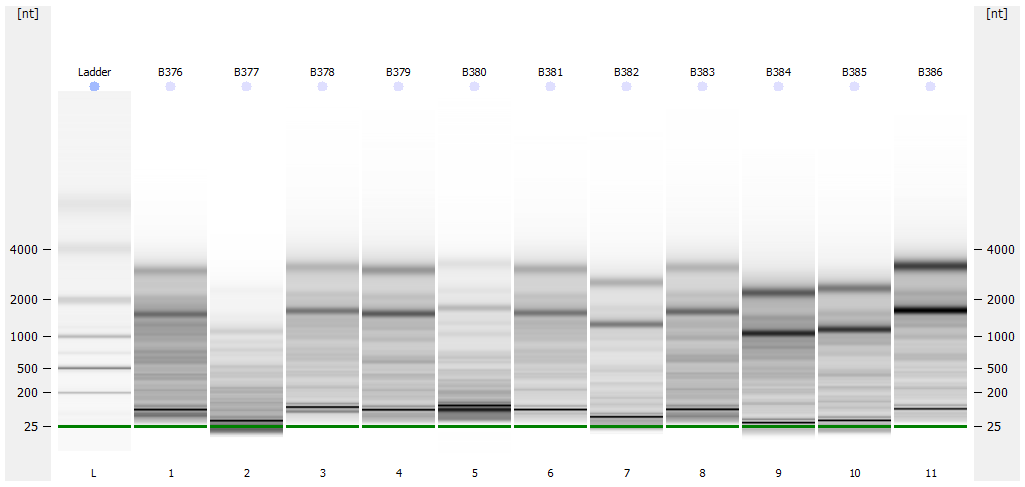  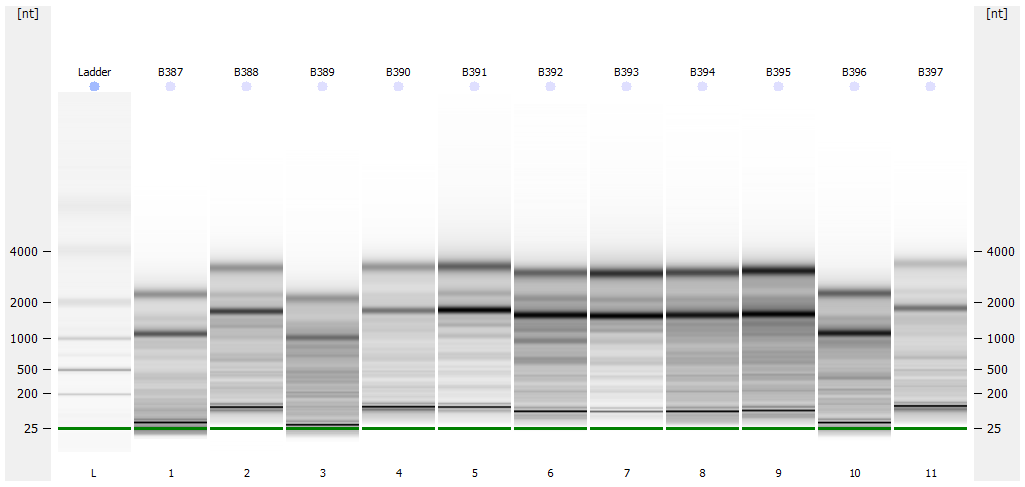  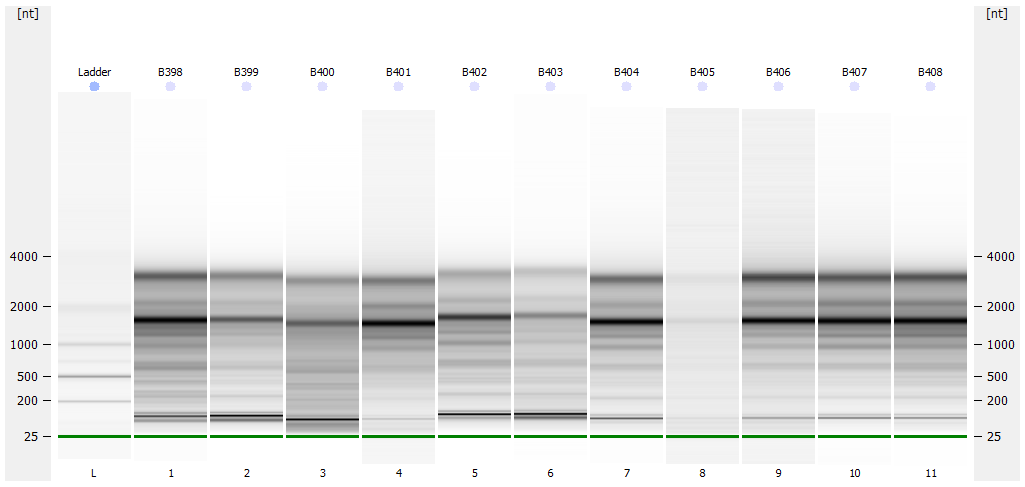  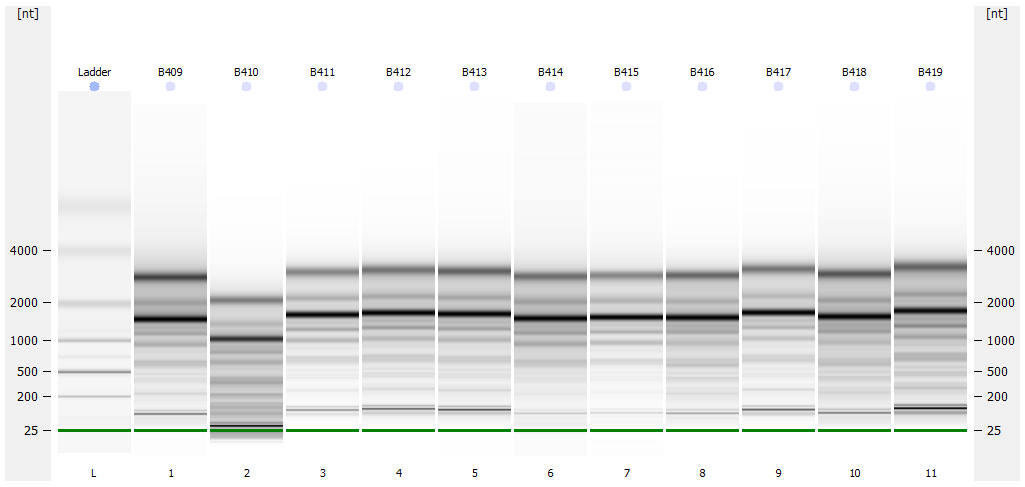  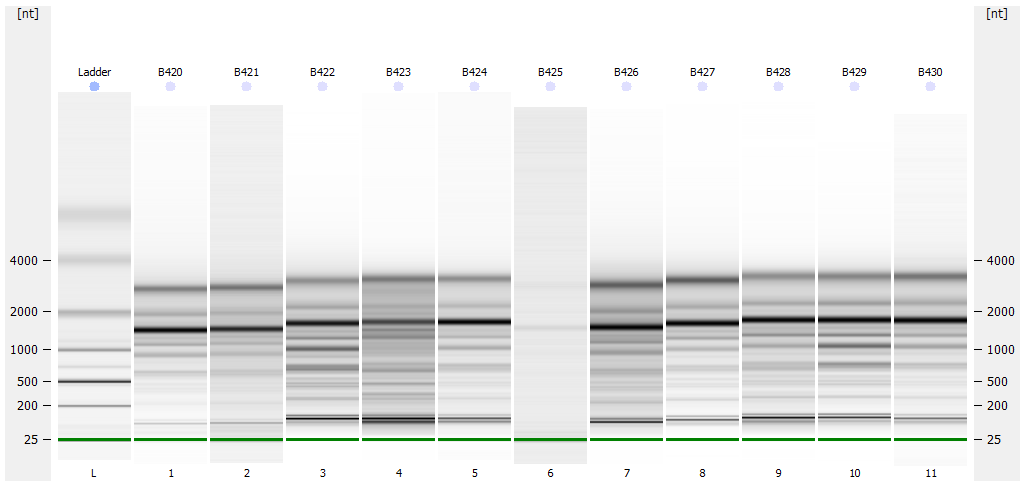  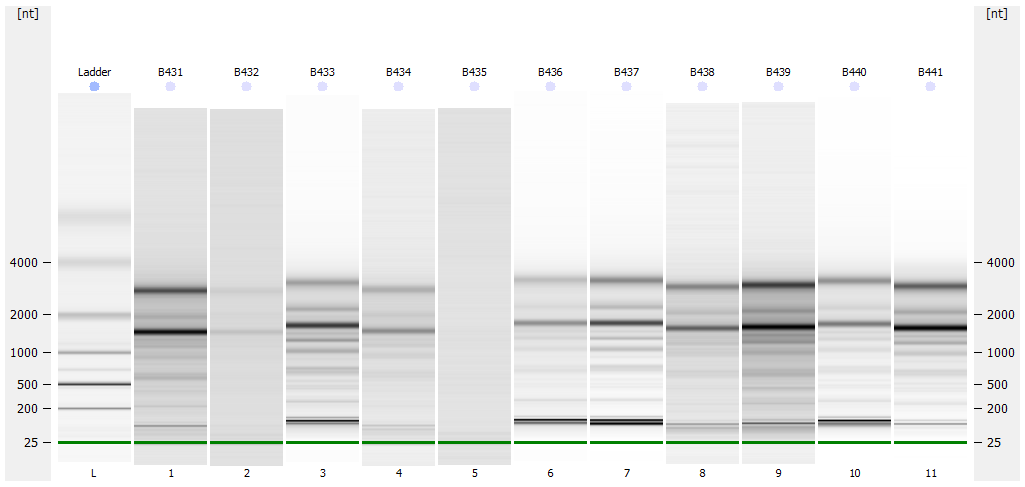  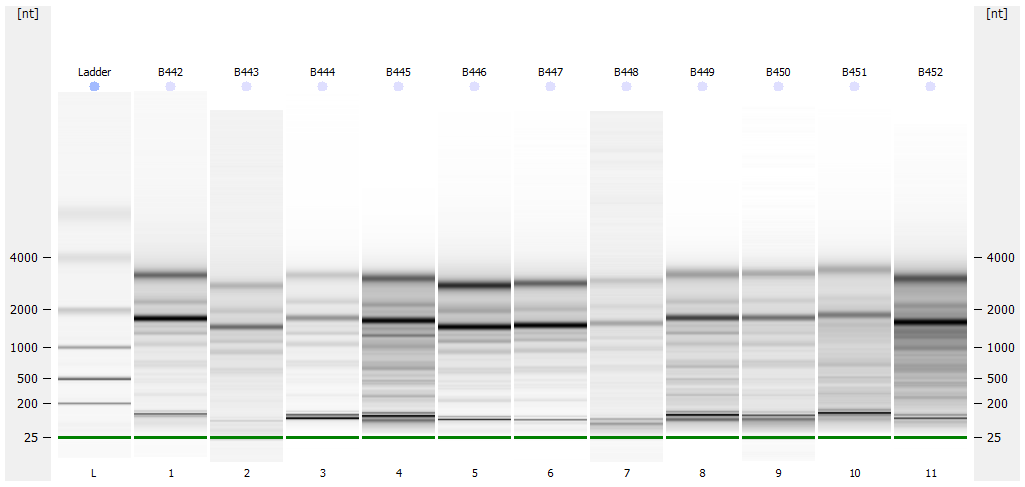  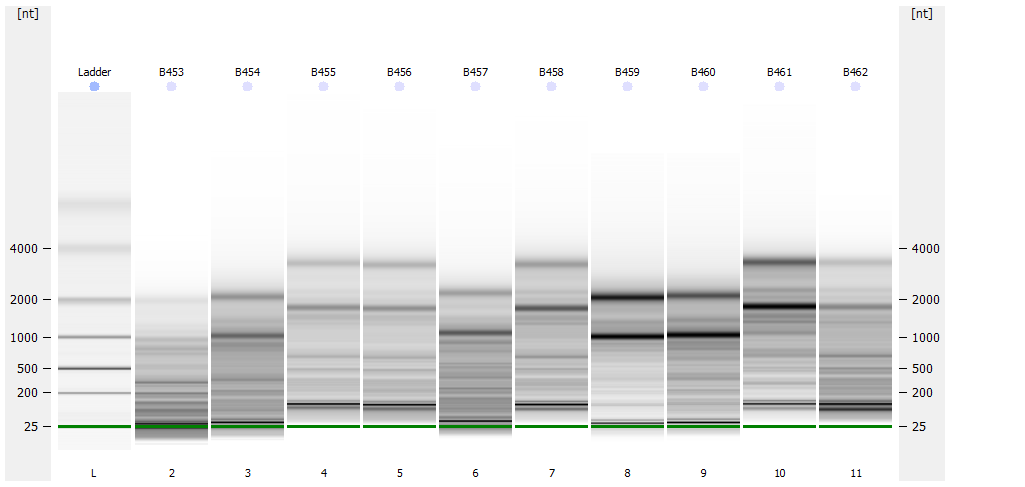  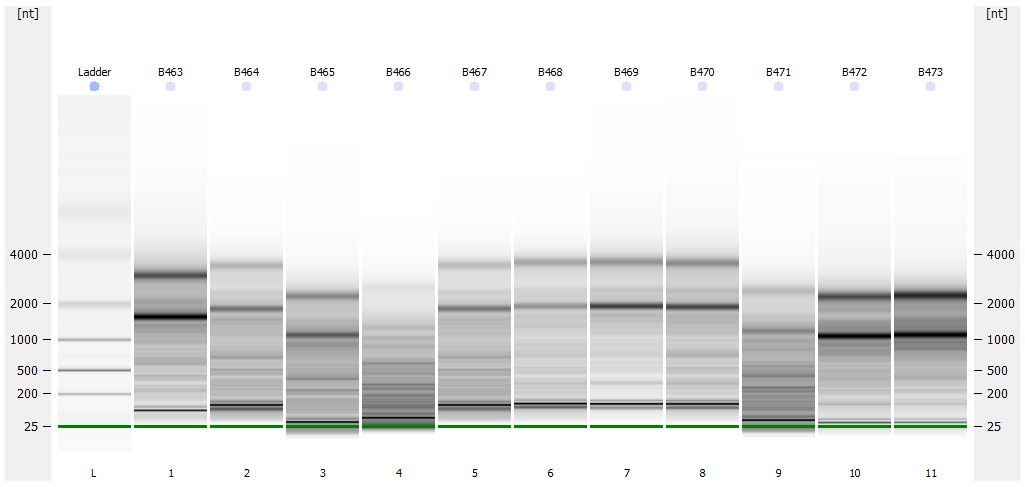  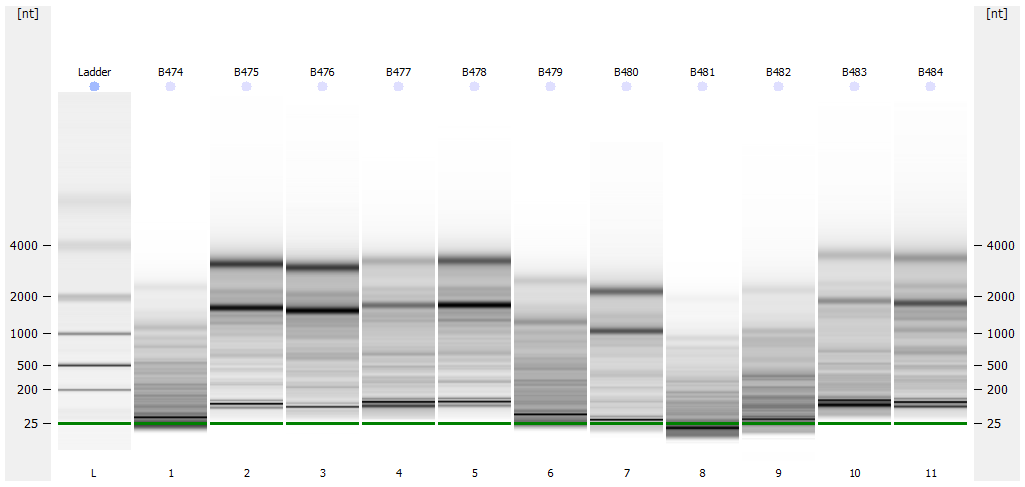               \|  \| \| --- \| \|  \| | | | |
